# scDenorm: a denormalization tool for integrating single-cell transcriptomics data

**DOI:** 10.1093/gigascience/giag032

**Published:** 2026-03-31

**Authors:** Yin Huang, Anna Vathrakokoili Pournara, Ying Ao, Ziliang Huang, Hui Zhang, Yongjian Zhang, Sheng Liu, Alvis Brazma, Irene Papatheodorou, Xinlu Yang, Ming Shi, Zhichao Miao

**Affiliations:** Translational Research Institute of Brain and Brain-Like Intelligence and Department of Anesthesiology, Shanghai Fourth People’s Hospital Affiliated to Tongji University School of Medicine, Shanghai 200434, China; Guangzhou National Laboratory, Guangzhou International Bio Island, Guangzhou 510005, China; European Molecular Biology Laboratory, European Bioinformatics Institute, EMBL-EBI, Wellcome Genome Campus, Cambridge CB10 1SD, UK; GMU-GIBH Joint School of Life Sciences, Guangzhou Medical University, Guangzhou 511436, China; Guangzhou National Laboratory, Guangzhou International Bio Island, Guangzhou 510005, China; Department of Obstetrics and Gynaecology, Harbin Red Cross Central Hospital, Harbin 150001, China; Department of Surgery Oncology, Harbin Medical University Cancer Hospital, Harbin 150001, China; State Key Laboratory of Ophthalmology, Zhongshan Ophthalmic Center, Sun Yat-sen University, Guangdong Provincial Key Laboratory of Ophthalmology and Visual Science, Guangzhou 510623, China; Guangdong Province Key Laboratory of Brain Function and Disease, Guangzhou 510623, China; European Molecular Biology Laboratory, European Bioinformatics Institute, EMBL-EBI, Wellcome Genome Campus, Cambridge CB10 1SD, UK; European Molecular Biology Laboratory, European Bioinformatics Institute, EMBL-EBI, Wellcome Genome Campus, Cambridge CB10 1SD, UK; Department of Obstetrics and Gynaecology, Harbin Red Cross Central Hospital, Harbin 150001, China; School of Life Science and Technology, Harbin Institute of Technology, Harbin 150001, China; Guangzhou National Laboratory, Guangzhou International Bio Island, Guangzhou 510005, China; GMU-GIBH Joint School of Life Sciences, Guangzhou Medical University, Guangzhou 511436, China

## Abstract

Integrating single-cell omics data at an atlas scale enhances our understanding of cell types and disease mechanisms. However, the integration of data processed by different normalization methods can lead to biases, such as unexpected batch effects and gene expression distortion, leading to misinterpretations in downstream analysis. To address these challenges, we present scDenorm, an algorithm that reverts delta-method normalized single-cell omics data to raw counts, preserving the integrity of the original measurements and ensuring consistent data processing during integration. We evaluated scDenorm’s performance on large-scale datasets and benchmarked its impact on data integration and downstream analysis across 3 datasets.

## Background

Single-cell RNA sequencing (scRNA-seq) is a powerful high-throughput technology for measuring gene expression in individual cells. Integration of atlas-level single-cell transcriptomics data has exerted great potential for understanding how cells orchestrate in the human body, as well as complex molecular mechanisms in various diseases [1, 2]. With the progress of the Human Cell Atlas (HCA) [3], an increasing number of reference atlases are available for comparison and integration [4–6]. Numerous integration methods have been developed, and several studies have been performed to benchmark their performance and explore their limitations [7–9]. To achieve effective large-scale data integration, it is crucial to account for assumptions about data distribution and noise levels. For instance, scVI [10] and scANVI [11] integration methods model single-cell data using a negative binomial distribution (also known as the Gamma–Poisson distribution), and thus both require raw counts as input. Even though some other integration methods (e.g., Seurat integration methods [12] [RPCA, CCA], Harmony [13], Liger [14]) do not directly rely on raw counts, they inherently make assumptions about the data distribution. As a result, for most existing integration methods, it is key to ensure the consistency of the input datasets.

To address technical variations (e.g., sequencing depth) and biases inherent in scRNA-seq, scaling and transformation methods are often employed to ensure comparability across cells [15, 16]. Normally, scaling is used to account for sequencing depth, while transformation is used to stabilize the variance of the data. The differences between variance-stabilizing transformations have been benchmarked by Constantin Ahlmann-Eltze and Wolfgang Huber [17], demonstrating the effectiveness of the delta method for comparing cells with varying gene expression levels. In a delta normalization, raw counts are scaled by total counts and target sum, followed by log-transformation with an added pseudo-count (see Methods). It has been adopted in well-established analysis workflows (e.g., Seurat [18] and SCANPY [19]), assuming that droplet-based scRNA-seq data follow a negative binomial distribution [20–23]. In some large-scale data resources, such as the UCSC Cell Browser [24], delta method–normalized matrices are deposited instead of the raw counts to facilitate reproducibility of analysis results. Thus, many datasets are available only as processed matrices rather than as raw counts, hindering atlas-level data integration.

The best way to guarantee consistent data processing in large-scale data integration is to use raw counts as input. If we integrate normalized data with raw counts, processed data can be renormalized again while the raw counts are being normalized, thus introducing unnecessary biases. Some downstream analysis steps [25–27] (e.g., multinomial model-based highly variable gene selection [28], differential gene expression analysis by statistical modeling of read counts [29]) also assume raw counts as input. When raw counts are not available, researchers often seek to obtain the raw sequencing data and reanalyze them, including secondary analysis of reads mapping, demultiplexing, and quantification analysis [30], to obtain the raw count matrix. However, a count matrix from the reanalysis may deviate from the original published analysis in terms of reference genome and cell barcodes. Thus, the cell-type annotation or other metadata reported in the raw publication cannot be used, rendering difficulties in reproducing the analysis results. Besides, this secondary analysis can be both computationally expensive and time-consuming. Therefore, reliable conversion of normalized matrices back to raw counts can benefit large-scale data integration tasks as well as wider use of publicly deposited data. Yet, there is no tool available to meet this urgent need.

In this study, we propose scDenorm, an algorithm that converts delta method–normalized gene expression data back to the raw counts. It effectively explores key implicit features of the data distribution in scRNA-seq and recovers raw count matrices. Based on benchmarking across large-scale datasets, as well as application studies of downstream analysis, we demonstrate the capability, accuracy, scalability, and efficiency of this method. Moreover, scDenorm can deal with different normalization parameters, thereby facilitating data integration, consistent downstream analyses, and the construction of atlases.

## Results

### Inconsistent data normalization may generate biases in data integration

**Figure 1:**
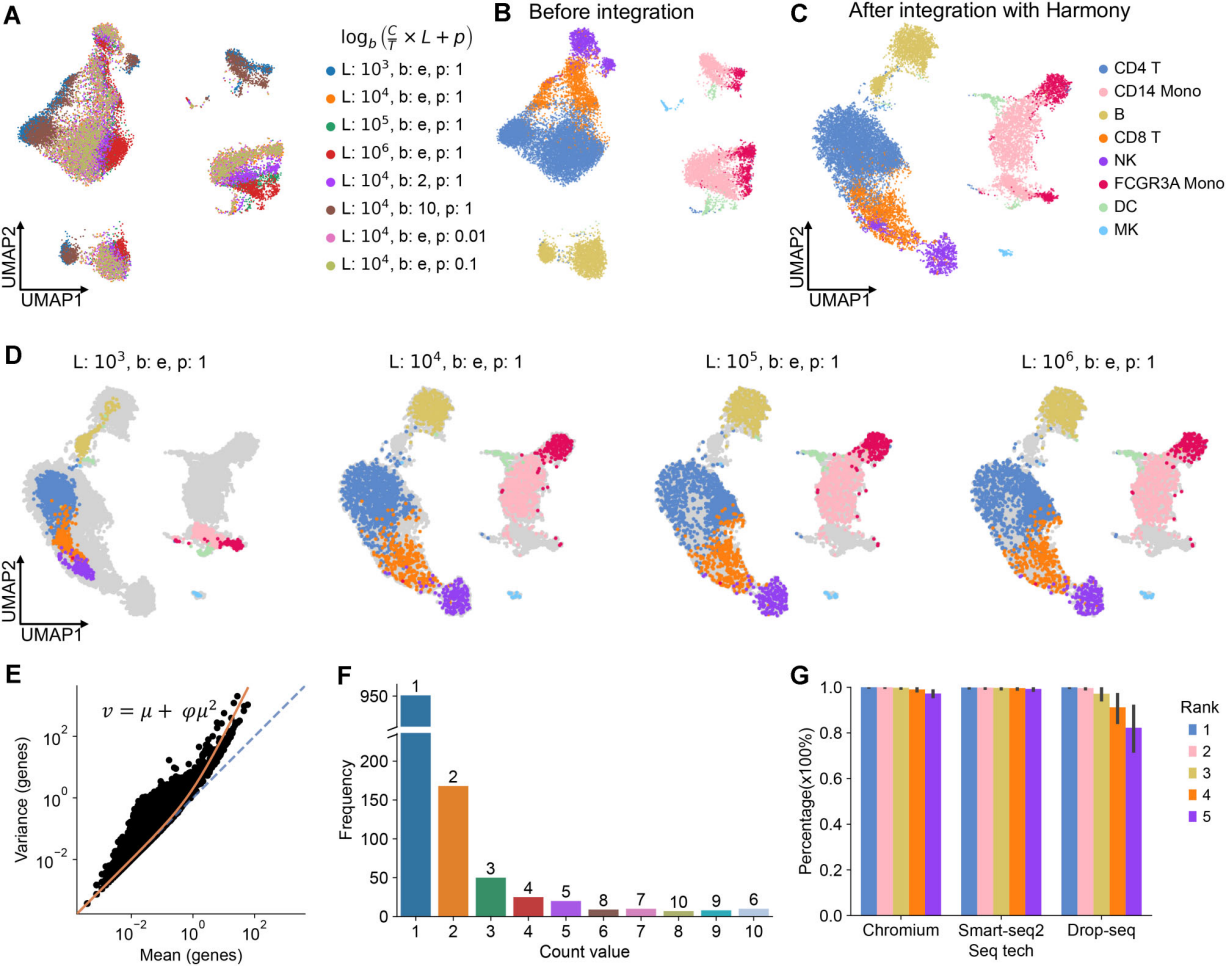
The data distribution of droplet-based single-cell data. (A) UMAP plot of PBMC 3k datasets, without data integration, normalized with different delta normalization parameters, including target sum (L), logarithmic base (b), and pseudo-count (p). The plot is colored by different parameter sets. (B) The same UMAP plot as panel (A) colored according to cell-type annotation. (C) UMAP plot after Harmony integration of data, normalized by different parameters, colored by cell types. (D) The UMAP plots are the same as panel (C) (after data integration by Harmony), displaying 4 different normalization parameter sets. (E) Scatterplot demonstrating the mean (x-axis) against the variance (y-axis) for each gene in the count matrix of the PBMC 3k datasets. Each dot shows the mean and variance value of a gene. The diagonal line is shown in blue. The orange curve is the fitted curve of the negative binomial distribution with variance ν, mean μ, and dispersion φ. (F) Histogram depicting the frequencies of count values and their ranks in a single cell, showing the “count-rank” distribution in a cell selected from the count matrix of the PBMC 3k datasets. (G) The percentage of cells that follow the “count-rank” distribution (the value of count equal to its rank from 1 to 5) in 3 scRNA-seq technologies (Chromium, Smart-seq2, and Drop-seq).

Using the 10×3k peripheral blood mononuclear cell (PBMC) data, which include the example data used in the well-established SCANPY [19] and Seurat [18] single-cell tutorials, as an example, we investigated the impact of normalization parameters in the delta method. These parameters are the scaling factor, logarithmic transformation base, and pseudo-counts. Matrices normalized by different parameters go through the same downstream analysis of highly variable gene selection, dimensionality reduction (e.g., principal component analysis), clustering, and visualization. The Uniform Manifold Approximation and Projection (UMAP) plot shows deviations between datasets processed with different normalization parameters, for example, the deviations between B cells in L = 10^3^ and the same B cells in other normalizations, indicating the potential bias introduced by inconsistent data normalization (Fig. 1A, B, Supplementary Fig. S1a). Furthermore, such a data normalization effect cannot be removed through data integration by Harmony [13], scanorama [31], or BBKNN [32] (Fig. 1, Supplementary Fig. S1); for example, the B-cell populations in Fig. 1C, D cluster separately. Therefore, we suggest converting the normalized matrices back to raw counts for consistent data integration and downstream analysis.

### The denormalization process in scDenorm

We term the recovery of normalized data to raw counts as “denormalization.” Denormalizing delta method normalized data requires the determination of 3 parameters: scaling factors, the logarithmic transformation (log-transformation) base, and the pseudo-count. The first step for denormalization is to determine if a log-transformation has been applied to the whole expression matrix. It is well established that droplet-based scRNA-seq data follow a negative binomial distribution [20–23], where the variance exceeds the mean (Fig. 1E). Thus, the variance versus mean distribution effectively indicates whether the data have been log-transformed or not (Supplementary Fig. S2a). The second key step in denormalization is to determine the scaling factor for each cell. This needs to exploit the implicit data distribution feature of scRNA-seq. Droplet-based scRNA-seq mainly probes the highly expressed genes, rendering a high dropout rate. In a sparse matrix where zeros have been removed, the frequency of counts can be ranked, with the most frequent count number being 1, followed by 2, and so on (Fig. 1F, Supplementary Fig. S2b, c). Using such a “count-rank” distribution, scaling factors for cells can be measured by establishing the relationship between the top 2 most frequent numbers in the normalized data and numbers 1 and 2. After exploring 105 datasets from the Brain Cell Atlas [33], we found that over 99% of the cells follow this “count-rank” distribution for the top 3 most frequent count numbers (1, 2, and 3), while >95% cells in Chromium and >80% cells in Drop-seq follow the distribution for the top 5 count numbers. Notably, >99% cells in Smart-seq2 data follow this distribution for the top 10 numbers (Fig. 1G, Supplementary Fig. S2d). Following this count-rank distribution, the top most frequent count numbers can be used to determine the 3 parameters in delta method normalization (Supplementary Fig. S3a).

The denormalization procedure in scDenorm involves 2 steps: detransformation and unscaling (Supplementary Fig. S3b). In the detransformation step, a subset matrix (100 cells) is used to determine the same log-transformation base and pseudo-count among cells since these 2 parameters keep the same for the whole expression matrix. Using a subset of data effectively accelerates the calculation. First, empirical values (e.g., 2, e Euler’s number, 10 for the log-transformation base, 0.01, 0.1, 1 for pseudo-count), which are used in standard analysis workflows, are tried. If not successful, these 2 parameters can be determined by solving equations between the top 2 most frequent numbers (Supplementary Fig. S3c). In the unscaling step, each cell has a different scaling factor, which is a ratio between the total counts of the cell and the target sum (e.g., 10,000). To measure the scaling factor of a cell, we implement 2 methods: (i) a regression-based method (see Methods, equation (4) in Supplementary Fig. S3d) and (ii) solving equations between the top 2 most frequent numbers (see Methods, equation (5) in Supplementary Fig. S3d), while the latter method offers the advantages of fast speed and good robustness (Supplementary Fig. S4a, b). As the expression matrix is processed from raw counts, which consist of integers only, a successful denormalization should result in a small mean square error between denormalized values and their nearest integers (see Methods).

**Figure 2:**
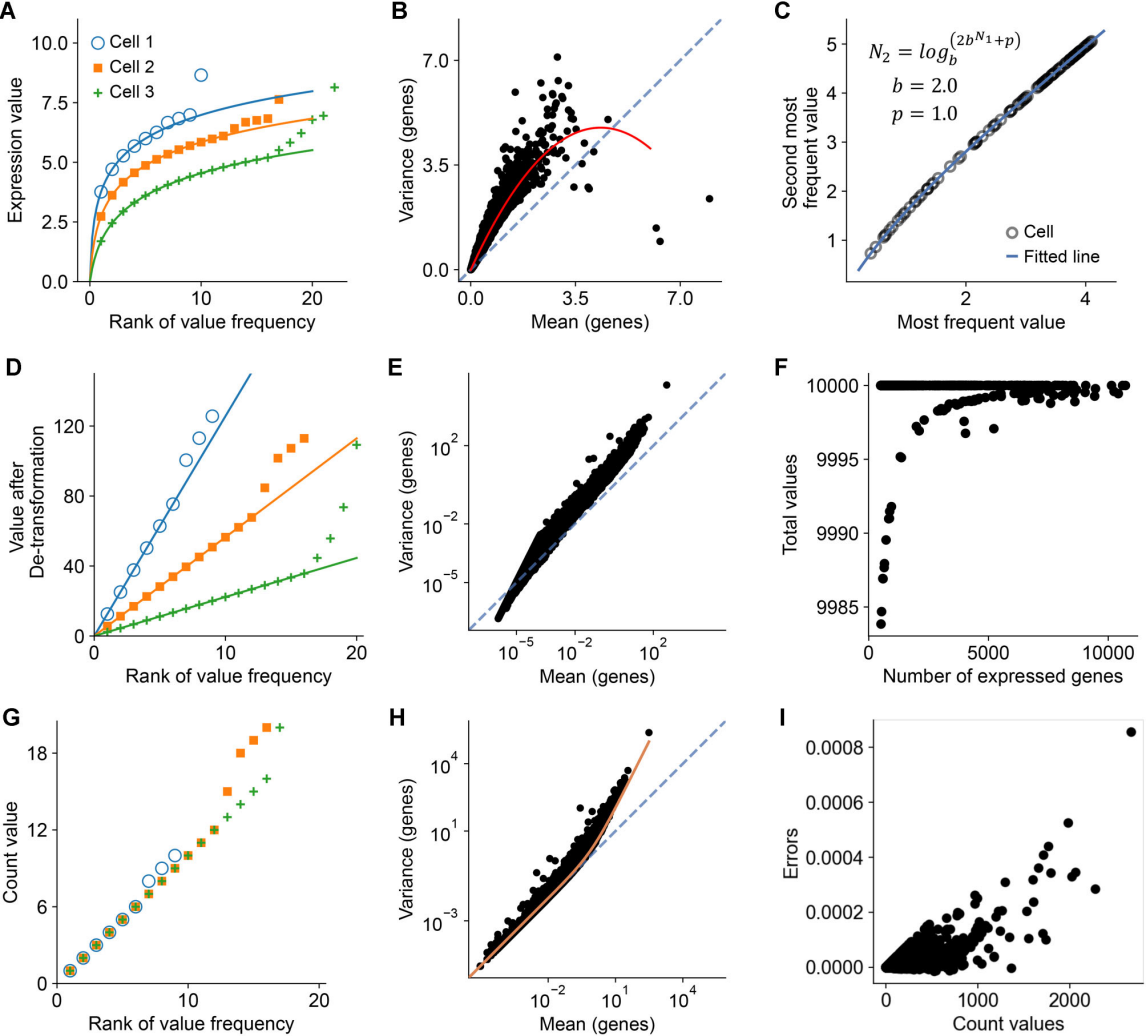
Evaluation of scDenorm on normalized scRNA-seq data with known raw counts. (A) The scatterplot shows the distribution between expression values and their ranks of frequencies in 3 example cells. Each dot is an expression value and its rank of frequency in the cell; different cells are shown in different shapes and colors. (B) The scatterplot shows the distribution between the log-transformed mean expression (x-axis) and the log-transformed variance (y-axis) for each gene in the gene expression matrix from the Velmeshev et al. dataset. The diagonal line (x = y) is shown in blue. (C) The scatterplot shows the distribution between the most and second most frequent values in different cells, displaying each cell as a dot. The blue curve shows the fitted equation derived from equation (4) in the Methods, with base value (b) equal to 2 and pseudo-count (p) equal to 1. N_1_ and N_2_ are the most and second most frequent values in cells, respectively. (D) The scatterplot shows the distribution between expression values and their ranks of frequencies in the 3 example cells after detransformation, colored in the same manner as panel (A). (E) The scatterplot shows the relationship between the mean expression (x-axis) and variance (y-axis) for each gene after detransformation. (F) The scatterplot shows the distribution between the number of genes and the target sum (sum of all expression values) in the cell after detransformation. (G) The scatterplot shows the distribution between expression values and their ranks of frequencies in the 3 example cells after unscaling, displaying the count-rank distribution. (H) The dot plot shows the distribution between the mean expression (x-axis) and the variance (y-axis) for each gene in the count matrix. (I) The scatterplot shows the distribution between the count values and the errors between the denormalized matrix and the raw count matrix after denormalization. Each dot represents a count value in a cell and its rounding error.

To elaborate on the denormalization process, we used an example dataset [34] of single-nucleus RNA sequencing (snRNA-seq) data of autism spectrum disorder, with both the normalized data and raw count matrix available in the autism database (see Data Availability). According to the respective publication [34], the data were normalized with the delta method. The relationship between the top 10 most frequent gene expression values and their respective frequencies in 3 cells in the processed data (Fig. 2A) suggests a logarithmic distribution, while the less frequent values after them do not follow such a distribution due to dropouts. If any of the cells in the dataset were to follow this pattern, the mean versus variance distribution would support a logarithmic transformation (Fig. 2B). The log-transformation base and the pseudo-count are determined as 2 and 1, respectively, by solving equation (3) in Supplementary Fig. S3c. These parameters show a good fit according to the top 2 most frequent values (Fig. 2C). The normalized matrix is detransformed by taking the exponential of the log-transformation base and subtracting the pseudo-count, resulting in a “scaled matrix.” In the scaled matrix, the top 5 most frequent values show a linear “count-rank” distribution in each cell (Fig. 2D). The slope of the line is the reciprocal of the scaling factor. This linear distribution indicates the success of detransformation. Additionally, the mean versus variance distribution (Fig. 2E) confirms this success. The summed expression values for most cells are approximately 10,000, indicating that the target sum is 10,000. Some genes may have been removed after normalization, leading to a reduction in the summed expression values (Fig. 2F). In the unscaling step, scaling factors are determined by solving equation (5) in Supplementary Fig. S3d. Each cell is multiplied by its scaling factor, resulting in a “denormalized matrix,” which is supposed to be similar to the raw count matrix of integers. As in a sparse matrix, the top 2 most frequent numbers should be 1 and 2 (Fig. 2G). The mean versus variance distribution of the denormalized matrix conforms to a negative binomial distribution (Fig. 2H), which is expected for the raw counts of droplet-based scRNA-seq. Comparing the denormalized matrix with the raw count matrix, the maximum error for each value was less than 0.001 (Fig. 2I), which may result from the digital float calculation. After taking round values, the denormalized matrix is identical to the raw count matrix, suggesting a successful denormalization.

### scDenorm recovers raw count matrices for a large-scale database

**Figure 3:**
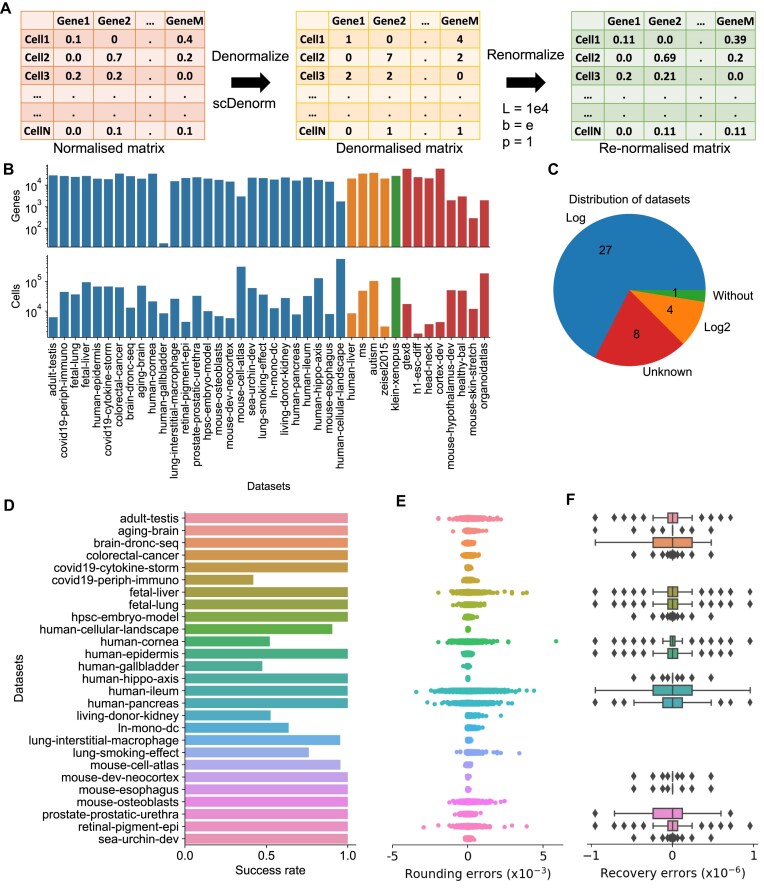
Performance of scDenorm on normalized scRNA-seq data from the UCSC Cell Browser. (A) The diagram illustrates the workflow of scDenorm to evaluate denormalization using the rounding and recovery error. The normalized matrix (left) deposited in the UCSC database is first denormalized (middle) with scDenorm to calculate the rounding error (see Methods). Subsequently, the denormalized matrix (middle) is renormalized (right) to measure the recovery error (see Methods). These matrices are used to calculate rounding errors (the difference between the denormalized matrix and its rounding matrix) and recovery errors (the difference between the normalized matrix and the renormalized matrix). The values of target sum and pseudo-count normalization parameters are 1e4 and 1, respectively. (B) Two barplots show the number of genes (top) and the number of cells (bottom) for the collected UCSC datasets. The x-axis shows the datasets by name, while the y-axis shows the log-scaled number of genes (top) and the log-scaled number of cells (bottom). The colors represent the different parameters of the delta normalization. Blue and orange are natural base(e) and base = 2, respectively; green represents data without log-transformation. Red shows non–delta method normalization cases, which could not be denormalized by scDenorm. (C) The pie chart shows the distribution of the number of datasets classified by different base values, detected by scDenorm. The colors are the same as shown in panel (B). “Unknown” represents unsuccessful cases that were not normalized with the delta method, while all other 32 cases were successful. (D) The bar plot shows the distribution of the success rate (see Methods) across the 27 datasets that were normalized with natural logarithmic transformation. (E) The jitter plot shows the distribution of rounding errors observed in the denormalized datasets. The x-axis is the rounding error, while the y-axis shows the same datasets as panel (D). (F) The boxplot shows the distribution of recovery errors after renormalization with the parameters of target sum, pseudo-count, and logarithm base as 1e4, 1, and natural base(e), respectively. The x-axis is the recovery error, while the y-axis shows the datasets in the same order as in panel (D).

To evaluate the performance of scDenorm in realistic scenarios, 40 processed datasets (Supplementary Table S1) from the UCSC Cell Browser [24] were used as test data, covering a good variety of species, tissues, and sequencing techniques (Fig. 3B). Denormalization performance was evaluated by 2 metrics: (i) rounding error, defined as the difference between a value in the denormalized matrix and its nearest integer (round value), and (ii) recovery error, defined as the difference between a value in the normalized matrix and its corresponding value in the denormalized matrix after renormalization (Fig. 3A; see Methods). Thirty-two of the 40 test sets were successfully denormalized (Fig. 3C, Supplementary Table S1), while the 8 unsuccessful cases were normalized as transcripts per million (TPM), log2FPKM (Fragments Per Kilobase Million)), or by the scTransform [35] method rather than the delta method (Supplementary Table S1). The mean versus variance distribution confirms a negative binomial distribution after denormalization, indicating the successful denormalization (Supplementary Fig. S5). To further assess the robustness of scDenorm across diverse datasets, we evaluated its performance on 27 datasets normalized using a natural logarithmic transformation. We present the distribution of success rates (Fig. 3D), defined as the proportion of cells successfully denormalized (equation (11)). This metric accounts for cases where poor sequencing quality or a low number of expressed genes may result in cell-wise deviations from the expected negative binomial distribution, thereby preventing accurate recovery during denormalization. The results demonstrate that scDenorm performs robustly across diverse datasets, even when some cells cannot be fully recovered. The rounding errors, which are positively correlated to the expression value (Supplementary Fig. S6), are consistently below 0.005 (Fig. 3E). For recovery error, the absolute values are below 10^−6^ in 27 datasets normalized with natural logarithmic transformation (Supplementary Table S1), indicating a good accuracy of scDenorm (Fig. 3F). Further benchmarking of the denormalization on 60 datasets (Supplementary Table S2) from the Brain Cell Atlas [33] shows similar results (Supplementary Fig. S7). In addition, scDenorm shows a linear computational time complexity and memory usage with increasing numbers of cells and genes, demonstrating a high computational efficiency and scalability (Supplementary Fig. S4c, d).

### scDenorm accurately recovers raw counts in different scenarios

**Figure 4:**
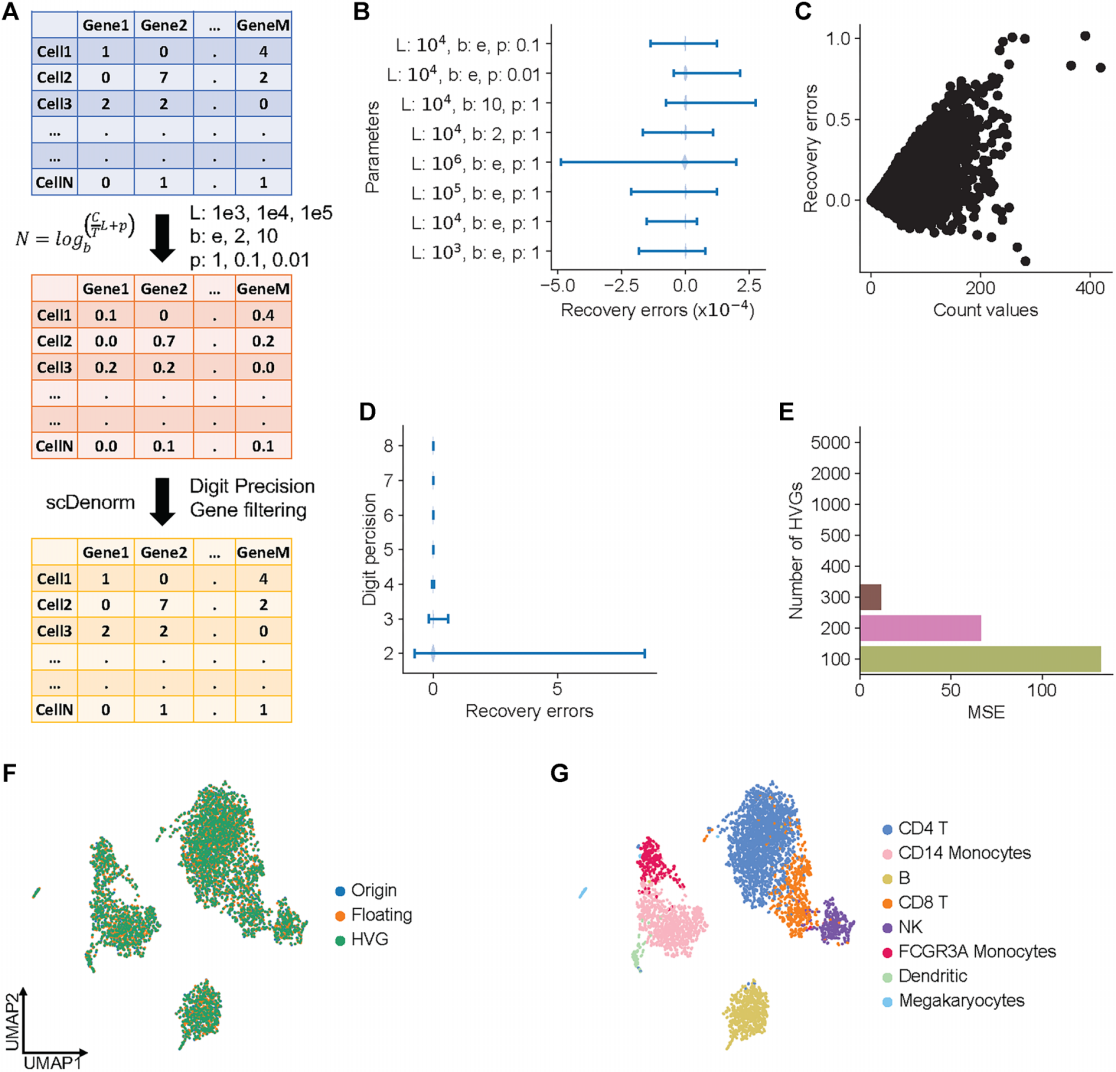
Benchmark of scDenorm on different normalization parameters, digital precision, and gene filtering. (A) The diagram shows the workflow of evaluating the recovery errors of denormalization in 3 scenarios (different normalization parameters, different digit precision, and gene filtering) on the PBMC 3k dataset. The raw count matrix (top) is first normalized with different parameter sets (middle) and then denormalized (bottom) with scDenorm, giving different digit precisions and filtered genes. In delta method normalization, C is the count value, T is the total count value of a cell, L is the target sum, p is the pseudo-count, b is the base of the logarithmic function, and N is the normalized gene expression value. (B) The line plot shows the distribution of recovery errors from using different parameter sets in delta normalization. (C) The dot plot shows the distribution between raw count values and their recovery errors after the conversion of normalized data from float32 to float16. (D) The line plot shows the distribution of recovery errors from normalized float32 data while preserving different levels of digit precision (2- to 8-digit precisions). (E) The histogram shows the mean square error of regression loss of equation (4) (see Methods) from normalized data with different numbers of highly variable genes (from 100 to 5,000). (F) The UMAP plot shows the distribution of data processed using different approaches, including original processed data (blue), denormalized data after converting to float16 (orange), and denormalized data after selecting 2,000 highly variable genes (green). Both the original processed data and the denormalized data show similar UMAP visualizations. (G) The UMAP plot shows the cell-type distribution of panel (F), color-coded by cell type. Both the original processed data and the denormalized data capture the same cell types.

In realistic scenarios, denormalizing the normalized matrix deposited in the database can be affected by several aspects, including (i) the parameters used in delta normalization method, (ii) the digital precision kept in the deposited data, and (iii) the genes filtered after data normalization (Fig. 4A), (e.g., some lowly expressed genes could be removed). Using the 10×3k PBMC single-cell dataset as a showcase, we benchmark these aspects.

We examined the effect of normalization parameters (target sum, log-transformation base, and pseudo-count) by simulating the normalization process with 8 sets of hierarchical parameters. The dataset was normalized using these parameters and denormalized by scDenorm. As shown in Fig. 4B, the errors between the denormalized value and its raw count in all denormalized matrices are consistently low, as <5×10^−4^, indicating a minimal impact from normalization parameters.

The digital precision of the normalized data, which can vary depending on the data-processing tools and the saved file format, can also affect computational memory consumption. By default, the normalized data are saved as float32 (single-precision floating-point) format, with a precision of 6 to 9 decimal digits [36]. We simulated data with lower precision and denormalized them with scDenorm. The recovery error was less than 0.5 for count values less than 100 and less than 1 for count values greater than 100 (Fig. 4C). The errors are less than 0.01 when the digit precisions are more than 4 digits. The precision achieved with 3 to 4 decimal digits was consistent with the results of float16 conversion (Fig. 4D). Yet, 2-decimal precision shows larger errors in highly expressed genes but keeps the cell identities (Supplementary Fig. S8).

In scRNA-seq data analysis, some genes expressed in few cells need to be removed, or only selected genes may be kept in the normalized matrix. We simulated a gradient of the number of selected genes and tested the impact on denormalization. No detectable error was found when more than 300 genes were kept in the normalized matrix, with the error increasing as the number of genes decreased (Fig. 4E, Supplementary Fig. S9). However, downstream data visualization demonstrates that the denormalized matrices from float16 precision and a selection of 2,000 highly variable genes successfully recovered the UMAP representation derived from raw counts (Fig. 4F, G), despite minor differences in the values.

### scDenorm facilitates downstream analysis

We further evaluated the impact of denormalization on downstream analysis tasks, including data integration, cell-type annotation, differential expression (DE) analysis, and Gene Ontology (GO) analysis. As data from different batches may go through different normalizations, 3 datasets were prepared to cover different batch types. The batch in the COVID-19 PBMC [37] dataset includes samples from 2 patients; in the human prefrontal cortex (PFC) [34, 38] dataset, samples are from 2 different studies; and the batch in the human skin [39] dataset includes groups of samples from young and old donors.

**Figure 5:**
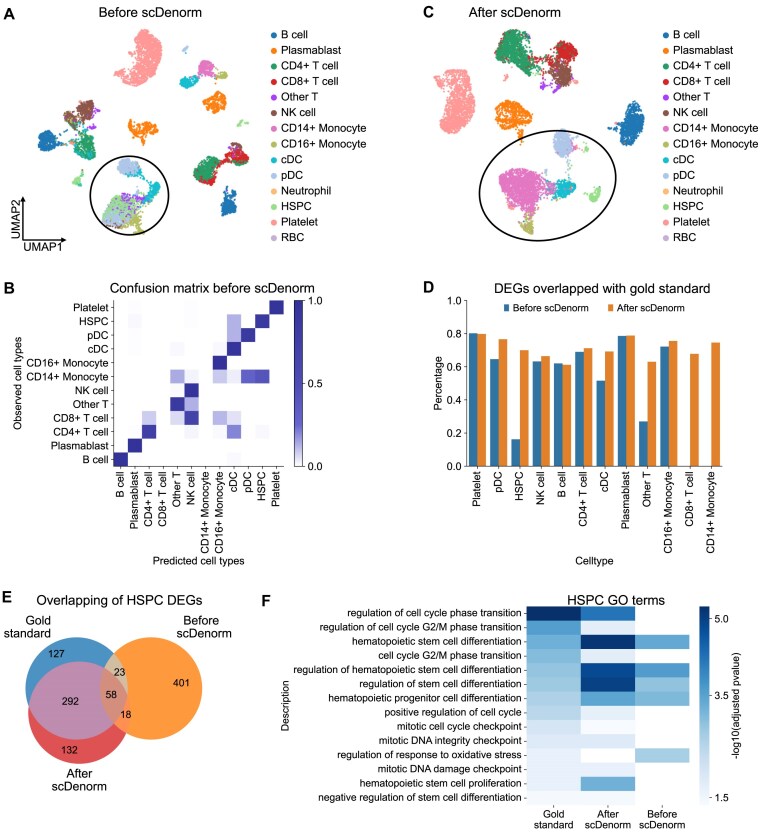
Different normalizations impact cell-type annotation of COVID-19 PBMCs. (A) The UMAP plot shows the distribution of cells before denormalization by scDenorm, colored by predicted cell-type annotation (see Methods). The 2 patient samples were normalized to different target sums (1,000 and 10,000). The UMAP shows that some cells of the same type exist in multiple clusters and mix with other cell types, as highlighted by the black circle. (B) The heatmap shows the confusion matrix between the published cell-type labels and the predicted cell types based on the Harmony-integrated latent space before scDenorm denormalization. The x-axis represents predicted cell types, while the y-axis denotes the original cell-type annotation published in the study. The confusion matrix was derived from SCCAF, based on logistic regression learning of the data. (C) The UMAP plot shows the distribution of cells after Harmony data integration followed by scDenorm. Cells are colored by predicted cell-type annotation. The UMAP shows that cells of the same type are clustered together. (D) The histogram shows the percentage of DEG overlap between the gold standard (DEGs derived according to the original published cell-type labels) and the ones derived from reanalysis before (blue) and after (orange) scDenorm across cell types. The DEGs are calculated with a 2-sided Wilcoxon test using the predicted cell types as clusters. (E) The Venn diagram shows the overlap of the top 500 DEGs for HSPCs derived from the gold standard (blue) from the original study, as well as before (orange) and after (red) scDenorm. (F) The heatmap shows the enriched GO terms of HSPCs derived from DEGs of the gold standard (blue) from the original study, as well as before (orange) and after (red) scDenorm.

The 2 patient samples in the COVID-19 PBMC [37] dataset were first normalized with different target sums (1,000 and 10,000) before going through downstream analysis (Supplementary Fig. S10). First, without denormalization, the UMAP visualization after harmony integration showed cells of the same cell type were in multiple clusters (e.g., plasmablast) (Fig. 5A). Subsequently, SCCAF [40], a well-established reference-based machine learning algorithm, was used to annotate the cell types. The first sample was used as a reference to annotate the cell types in the second sample, resulting in an accuracy of 66% (the consistency between the original cell-type labels and those assigned by SCCAF). Notably, CD14^+^ monocytes were misclassified as plasmacytoid dendritic cells (pDCs) and hematopoietic stem and progenitor cells (HSPCs), while CD8^+^ T cells were misclassified as natural killer (NK) cells and CD4^+^ T cells (Fig. 5B). Fortunately, with the help of scDenorm denormalization, the 2 patient samples could be integrated, with each cell-type cluster forming distinct clusters (Fig. 5C). The accuracy of cell-type annotation using SCCAF increased to 92%, indicating effective correction of denormalization. Furthermore, misannotated cell-type labels may result in biases in differential gene expression analysis (Fig. 5D, Supplementary Fig. S10d) and GO analysis (Supplementary Fig. S10e). For instance, 350 differential genes in HSPCs matched the “gold standard” after scDenorm, compared to only 81 before scDenorm (Fig. 5E). Gene enrichment analysis of the differential genes in HSPCs indicated that the GO terms enriched after scDenorm closely aligned with those of the gold standard, whereas the enrichment before scDenorm showed minimal overlap (Supplementary Fig. S10f). The enriched GO terms are relevant functions associated with HSPC cells, such as hematopoietic stem cell proliferation and hematopoietic progenitor cell differentiation (Fig. 5F).

**Figure 6:**
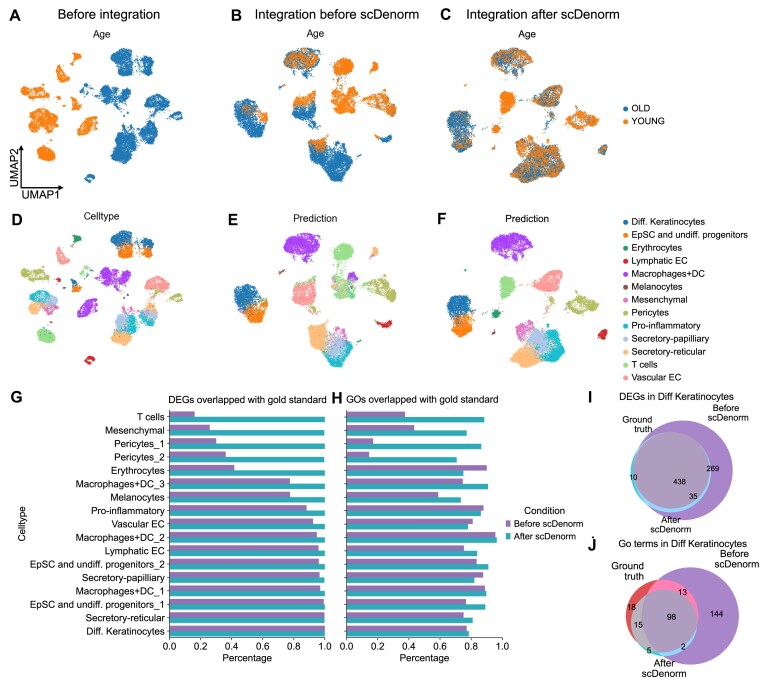
Different normalizations impact differential expression analysis. (A) The UMAP plot shows the cell distribution of the 2 age groups of the human skin dataset (Solé-Boldo et al.) before data integration without the scDenorm denormalization, colored by age group. (B) The UMAP plot shows the Harmony-integrated result without the scDenorm denormalization, colored by age group. (C) The UMAP plot shows the Harmony-integrated result after running scDenorm, colored by age group. (D) The UMAP plots show the same distribution as panel (A), colored by original cell-type labels from Solé-Boldo et al. (E, F) The UMAP plots show the same distribution as panels (B) and (C), but colored by predicted cell-type labels before and after denormalization by scDenorm. (G, H) The histograms show the percentages of DEGs (G) and GO terms (H) that overlap between the gold standard (DEGs extracted from the data in the original study, GO terms derived from these DEGs) and data before (purple) and after (blue) scDenorm across cell types, using the gold standard cell-type labels reported in the original publication. The DEGs are calculated with a 2-sided Wilcoxon test based on the original cell type from the human skin dataset (Solé-Boldo et al.), while the GO analysis shows a Benjamini–Hochberg–adjusted *P* value <0.05. (I, J) The Venn diagrams show the overlaps of the DEGs (I) (Benjamini–Hochberg–adjusted *P* value <0.05 and logFC >0.25) and GO terms (J) for differentiated keratinocytes derived from data of the gold standard (red), as well as before (purle) and after (blue) scDenorm denormalization.

**Figure 7:**
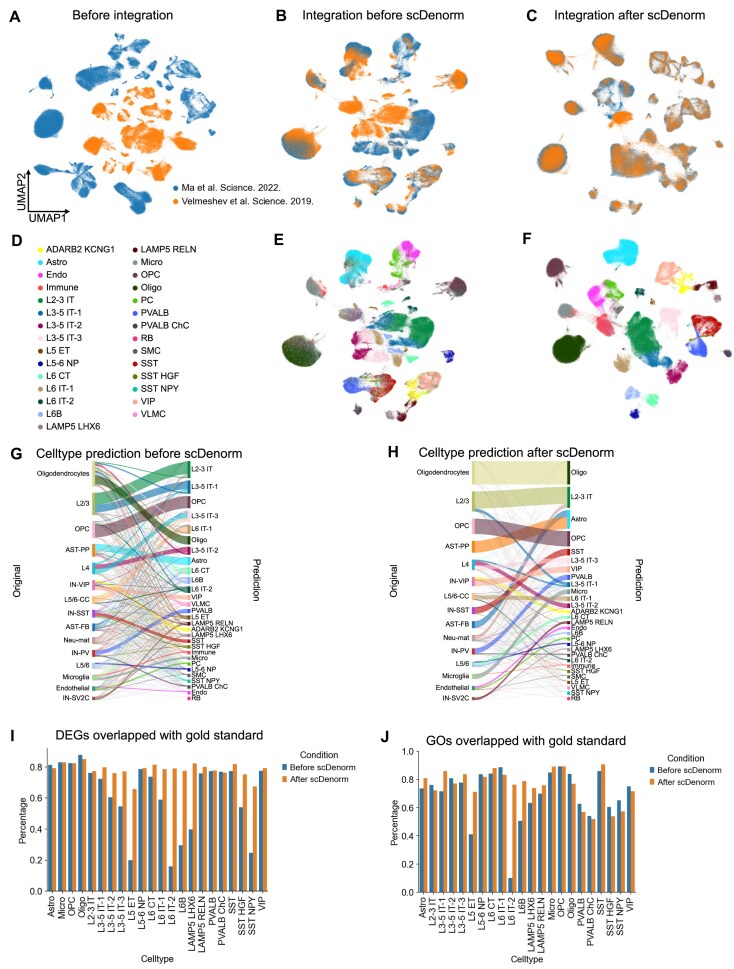
scDenorm helps in cell-type annotation and differential expression analysis on prefrontal cortex datasets. (A) The UMAP plot shows the distribution of cells of the 2 prefrontal cortex datasets before integration. (B) The UMAP plot shows Harmony-integrated results without the scDenorm denormalization, colored by studies. (C) The UMAP plot shows the Harmony-integrated result after running scDenorm, colored by studies. (D) The figure legend of cell-type annotation for panels (E) and (F). (E) The UMAP plot is the same as panel (B), colored by predicted cell-type annotation. (F) The UMAP plot is the same as panel (C), colored by predicted cell-type annotation. (G, H) River plot illustrates the transition between original and predicted cell types before scDenorm (G) and after scDenorm (H). The left side represents the original cell types from Velmeshev et al., while the right side displays the predicted cell types. (I) Bar plot showing the overlapping percentage of DEGs between the gold standard and before and after scDenorm across cell types. The DEGs are calculated with a 2-sided Wilcoxon test based on the predicted cell types. (J) Bar plot showing the overlapping percentage of GO terms between the gold standard and before and after scDenorm across cell types.

Similarly, the same analysis of data integration and cell-type annotation was performed on 2 other datasets, the human prefrontal cortex dataset and the human skin dataset. Both study-wise batch (the former dataset) and condition-wise batch (the latter dataset) demonstrated that data processed by scDenorm yielded superior integration results (Figs. 6, 7) and improved annotation results from SCCAF (Fig. 7G, H, Supplementary Fig. S11d, e). Yet, the mislabeled cell types led to biased differentially expressed genes (DEGs) (Fig. 7I) and GO terms (Fig. 7J).

Furthermore, we evaluate the impact of normalization parameters on downstream DE and GO analysis. Taking the human skin data [39] as an example, differential gene expression analysis was performed before and after scDenorm using the cell-type labels derived from the original publication, while the published differential expression genes (see Methods) and their GO results were taken as the gold standard. The DE and GO results after scDenorm show higher consistency with the gold standard than the results before scDenorm (Fig. 6G, H). In addition, the DEGs identified before scDenorm include more false-positive genes (Fig. 6I), resulting in the enrichment of unrelated GO terms (Fig. 6J), such as the nuclear transport function for keratinocyte cells (Supplementary Fig. S11f).

## Discussion

In our survey of 133 well-established single-cell studies, delta method normalization takes up >83% (110) of the datasets (Supplementary Table S3), since it is implemented in widely used SCANPY and Seurat analysis workflows. We demonstrate the capability of scDenorm on an example dataset and large-scale test sets from the UCSC database [24] and the Brain Cell Atlas [33]. Different parameter sets in the delta method normalization, as well as the digital precision kept in the normalized data, have a minimal effect on denormalization. Moreover, the number of genes kept after normalization does not significantly affect denormalization, unless the number of genes used is too small (fewer than 300) (Fig. 4E). In the 40 datasets from the UCSC database (Supplementary Table S1) and 60 datasets from Brain Cell Atlas (Supplementary Table S2), scDenorm successfully restored count values in most (88%) cases, with minimal rounding errors and recovery errors. Therefore, scDenorm may robustly recover matrices for the majority (estimated to be 80-90%) of the datasets, which are delta method normalized, while maintaining efficient computational speed (Supplementary Fig. S4c).

The limitations of scDenorm rely on specific prerequisites of the delta method normalization. Datasets normalized using alternative methods may not be compatible with scDenorm. For example, GLM residual methods (such as SCTransfrom [35]) and latent expression (such as Sanity [41] and Dino [42]) cannot be denormalized by scDenorm. Fortunately, other denormalization methods besides the delta method only constitute 10% to 20% of the datasets, and the raw counts of these datasets can be obtained from read mapping. Additionally, the performance of scDenorm may be influenced by the choice of normalization parameters and the quality of the input data. Cells whose gene expression distribution deviates from the assumptions of the negative binomial distribution may lead to the failure of the denormalization process.

Several case studies show that different normalizations can result in unnecessary deviations in downstream analysis, including data integration, cell-type annotation, differential gene expression, GO, and pathway analysis. In particular, biased differential expression or GO results can be generated due to different normalization parameters when the cell-type annotation is correct. Therefore, denormalizing the expression matrix to raw counts can be a good choice to mitigate biases in downstream analysis. It could be a key question for large-scale data integration, where study-wise batch effects need to be minimized while biology should be kept. Batch correction and data integration methods have already been extensively discussed and benchmarked [8]. Here we highlight the consistency in data processing, which is nontrivial when data from tens or hundreds of studies need to be combined. Consistent single-cell data analysis workflows that preserve the raw conclusions from publications and integrate with data from other studies would greatly help. Therefore, the availability and reproducibility of the raw published analysis code would be important.

## Conclusions

Here, we demonstrate that inconsistent data normalization can generate unexpected bias in data integration, potentially obstructing atlas-level single-cell data integration. Fortunately, denormalizing processed data back to raw counts could standardize analysis, thereby facilitating the creation of comprehensive cell atlases. We present scDenorm, a tool designed to denormalize data from the delta method normalization, which is widely used by 80% of the 40 datasets in the UCSC database and 93% of the 60 datasets in the Brain Cell Atlas. It employs both equation solving and regression methods to determine the parameters in the delta method. Benchmarks on 32 UCSC cell browser datasets and 56 Brain Cell Atlas datasets demonstrate the efficacy of scDenorm for delta method normalization data, with further applications on COVID-19 PBMCs, prefrontal cortex, and human skin datasets revealing its ability to mitigate biases in downstream analysis. scDenorm can be a useful tool in atlas-level single-cell data processing and integration, such as the Human Cell Atlas [43], the Human Developmental Cell Atlas [44], the Brain Cell Atlas [33], and HuBMAP [45].

## Methods

### Assumption and algorithm design

In scRNA-seq, the data are in the form of a count matrix, where most entries are zeros due to the sparsity of gene expression. Our assumption is that the scRNA-seq data follow a negative binomial distribution, which is theoretically and empirically well supported for the unique molecular identifier data [17]. This means that probabilistically speaking, in the count matrix, zero is the most frequently observed count, followed by 1, 2, and so on. The sequential pattern of these values has a probabilistic one-to-one correspondence with the rank of their frequency by descending order (Fig. 1F). The smaller the values, the higher the probability of the correspondence (Fig. 1G). For example, without considering 0, the probability that the values 1 and 2 equal the rank of their frequencies is almost 100%. Based on this assumption, we designed an algorithm to normalize scRNA-seq data that has been normalized by the most commonly used delta methods, which scale the raw counts by the total number of counts (library size) and target sum (the summed value of the cell after scaling), and then log-transformed after adding a pseudo-count (Supplementary Fig. S3a). Specifically, we consider a scaled expression matrix from a count matrix that has been transformed to adjust for differences in the scale of the features (e.g., genes) in the data. In scRNA-seq data, a scaled expression matrix typically refers to a count matrix that has been normalized and transformed to have a similar distribution of gene expression values across cells. For example, scRNA-seq data can be normalized to account for differences in sequencing depth and other technical factors that can affect the distribution of counts across cells and genes, such as total count normalization. It can also be transformed to adjust for the distribution of gene expression values across cells, such as log-transformation and variance-stabilizing transformation. The normalized gene expression matrix is derived from the count matrix to adjust for differences in gene expression across cells, which usually involves scaling and transformation techniques such as total count scaling and log-transformation. This normalization process does not change the one-to-one correspondence between the gene expression value and its rank of the value’s frequency in a cell.

Using the probabilistic one-to-one correspondence property, we can extract a cell vector from a normalized expression matrix and sort the values based on their frequency in the vector. This allows us to establish that the most frequently occurring nonzero value corresponds to 1, the second most frequent represents 2, and so forth, which means the rank number and the count number are theoretically the same, and this is normally true for the top ranks. By following this procedure, we were able to obtain the rank and normalized value pairs (C, N) (where C is the rank and N is the normalized count) for the equation $N\ = \ log_b^{( {\frac{C}{s} + p} )}$ (s is the scaling factor, b is the base of log-transformation, and p is the pseudo-count). First, we try reversing the log-transformation of natural base(e), base 2, and base 10 and solve the equation for the pairs of values (1, N1) and (2, N2), where N1 and N2 are the values of the 2 most frequent numbers. Normally, we think the pseudo-count C is given as 1. Otherwise, we need to check whether the variance of the solved C from different cells is sufficiently small, since each vector from the gene expression matrix has been augmented with the same pseudo-count. If the unscaling process is unsuccessful for all of the above cases, we conclude that the matrix has not been preprocessed according to the workflow. The following shows the complete workflow of the scDenorm algorithm.

The denormalization algorithm can be divided into 2 steps: detransformation and unscaling.

In detransformation, there are 2 sequential steps. First, (a) we search for empirical values for the log-transformation bases and the pseudo-count. It searches for empirical bases such as 2, e (natural base), and 10, as well as common pseudo-counts like 0, 0.01, 0.1, and 1. If the pseudo-count is 0, it indicates that the normalization process has not added the pseudo-count. A fraction of cells is used to evaluate if any of these bases or pseudo-counts meet the criteria in step (c). If passing the criteria, skip to step 2. Otherwise, it goes to step (b) to determine the parameters. Step (b) uses the equation-solving method to determine the parameters: this method uses the 2 values (${{N}_1}$, ${{N}_2}$) occurring most frequently in a cell to construct the following equation. For each cell i:


(1) \begin{eqnarray*}
\frac{1}{{{{s}_i}}}\ + \ p\ = \ {{b}^{N_1^i}}
\end{eqnarray*}

(2) \begin{eqnarray*}
\frac{2}{{{{s}_i}}}\ + \ p\ = \ {{b}^{N_2^i}}
\end{eqnarray*}

${{s}_i}$ is the scaling factor for cell i. The p and b are pseudo-count and base, respectively. From equations (1) and (2), we can get equation (3).


(3) \begin{eqnarray*}
p\ = \ 2 \times {{b}^{N_1^i}}\ - \ {{b}^{N_2^i}}
\end{eqnarray*}

Randomly select a group of cells (e.g., *n* = 100) to generate a corresponding set of data points (${{N}_1}$, ${{N}_2}$), and solve p and b by equation (4) with optimization methods.


(4) \begin{eqnarray*}
\arg \min \sum\nolimits_{i = 1}^n {} {{(p - 2 \times {{b}^{N_1^i}} + {{b}^{N_2^i}})}^2}
\end{eqnarray*}

The L-BFGS-B method from the sklearn [46] package is used to find the best base (b) and pseudo-count (p). This method is based on the limited-memory Broyden–Fletcher–Goldfarb–Shanno (BFGS) algorithm, which is capable of large-scale optimization. L-BFGS-B allows for box constraints, ensuring that the parameters stay within specified bounds during optimization.

After the detransformation, the sum of each cell should be the same or very similar. Step (c) checks if the sum of each cell is the same. For example, let X be the vector of the sums, and x is a number in it. If abs(x-mean(X)) is always smaller than the small number (e.g., mean(X) = 10,000, x = 9,999.7, small number is 0.5), then the detransformation is successful. However, this is an ideal situation. Often, we encounter that after normalization, the data filters out some genes for quality control in downstream analysis. In addition, some normalization methods do not scale total expression values equally across all cells. To address these complex cases, we also added the following criteria. If it is the automatic detection method, we only need to make sure that the mean square error (MSE) is small enough, such as ${{10}^{ - 5}}$. In general, we just need to unscale a cell to see if it is successful.

In unscaling, we have 2 approaches implemented in the same function, while a parameter can be used to select the option. The first approach (a) is based on regression to determine the scaling factors for all cells. The scaling factor is derived from fitting a regression model to the relationship between the detransformed values and their ranks, providing an estimate of the scaling factor for each cell. For each cell:


(5) \begin{eqnarray*}
{\mathrm{arg}}\,\,\mathop {{\mathrm{min}}}\limits_S \sum\limits_{(i = 1)}^n {{{{\left( {\frac{{{{c}_i}}}{S} + p - {{x}_i}} \right)}}^2}}
\end{eqnarray*}

${{c}_i}$ is the rank, and ${{x}_i}$ is the detransformed value.

To ensure a more accurate one-to-one correspondence, only the first 5 pairs of values (${{c}_i}$, ${{x}_i}$) are used. We can get the scaling factor s by optimizing equation (5) using the same L-BFGS-B method as in solving equation (4).

The second approach (b) involves solving equations for the top 2 most frequent values; this method uses only the first 2 pairs of values (${{c}_i}$, ${{x}_i}$). We can get a closed form of the solution by solving the following equation. For each cell:


(6) \begin{eqnarray*}
\frac{{{{c}_1}}}{s}{\mathrm{\ }} + {\mathrm{\ }}p{\mathrm{\ }} = {\mathrm{\ }}{{x}_1}
\end{eqnarray*}

(7) \begin{eqnarray*}
\frac{{{{c}_2}}}{s}{\mathrm{\ }} + {\mathrm{\ }}p{\mathrm{\ }} = {\mathrm{\ }}{{x}_2}
\end{eqnarray*}

From equations (6) and (7), we can get equation (8).


(8) \begin{eqnarray*}
s\ = \ \frac{{{{c}_2}\ - \ {{c}_1}}}{{{{x}_2} - \ {{x}_1}}}
\end{eqnarray*}

To evaluate the success of the denormalization process, we quantify the error between the denormalized values and their rounded counterparts. Ideally, denormalized values should closely approximate integers. We therefore compute the mean absolute error (MAE) between the denormalized matrix and its rounded count matrix and assess whether the MAE falls below a predefined threshold (default cutoff: 0.05). If the MAE exceeds this cutoff, the denormalization is considered unsuccessful. Of note, in some cases, the same top value (e.g., 1) can be normalized into more than 1 different value due to some improper data processing, and the ranks of these numbers are thus lower than expected. These numbers with tiny differences are merged as 1 value by decreasing their digital precision.

scDenorm is publicly available as an open-source Python package and provides a user-friendly Python function interface, which can be combined with the use of SCANPY analysis. It can be used both at the command line and interactively in a Jupyter notebook. A description of the function details is provided in the Supplementary Materials. Considering that different samples in a dataset may be normalized with different parameter sets, scDenorm also implements a per-sample denormalization function, overloading the original “scdenorm” function with a “by=sample” parameter as input.

### Integration of scRNA-seq data from different normalization parameters

We downloaded PBMC scRNA-seq data from the 10x Genomics datasets and preprocessed and annotated the data according to the pbmc3k Scanpy tutorial. Then, we used different parameter combinations (including 1e3, 1e4, 1e5, and 1e6 as target sums; 2, e, and 10 as base; and 1, 0.1, and 0.001 as pseudo-counts) to normalize the data separately and merge all the data together. Principal component analysis (PCA) of 50 components was derived from the expression matrix. Three single-cell data integration tools (Harmony, BBKNN, and scanorama) were tested to integrate the combined data with the normalization parameters as the batch key. For data visualization, UMAP [47] was calculated in the integrated latent space or the PCA space.

### Consistency of count–rank relationship across sequencing platforms

The consistency of the count–rankrelationship refers to the percentage of the correct one-to-one correspondence between the gene expression value and its rank of the value’s frequency in a cell. For example, given 100 cells, we first calculate the frequency of the raw count values (the raw count value is called count) in each cell and order the frequencies from highest to lowest. The order is called rank, which ranges from 1,2, …, n. If count is the same as rank, we consider this to be a correct one-to-one correspondence. Finally, for counts from 1 to 10, we respectively calculate what percentage of cells have the correct one-to-one correspondence as the consistency of the count–rank relationship. To compare different sequencing platforms, we calculated the consistency of the count–rank relationship in 105 datasets obtained from the Brain Cell Atlas. Among these datasets, 81 are from Chromium, 15 from Drop-seq, and 9 from Smart-seq2.

### Evaluation metrics

When benchmarking denormalization for scRNA-seq data, 2 measures can be used: rounding error and recovery error. Rounding error measures the discrepancy between the denormalized values and their rounded counterparts. After denormalization, the expected outcome is that the denormalized values approximate integers. Rounding error quantifies the extent to which the denormalized values deviate from integers. To calculate the rounding error, the difference between each denormalized value and its rounded value is computed; see equation (9). Recovery error evaluates the difference before denormalization and after renormalizing the denormalized values (values after scDenorm; Fig. 3A). To calculate recovery error, the difference between each normalized value and its renormalized value is computed; see equation (10).

Specifically, we assume x is the normalized value (a single value for 1 gene in 1 cell), y is the denormalized value after scDenorm, and z is the renormalized value from the denormalized value (y). The rounding error is calculated as the difference between the denormalized value (y) and its rounded value; see equation (9):


(9) \begin{eqnarray*}
{\mathrm{rounding}}\_{\mathrm{error}} = {\mathrm{round}}\left( {\mathrm{y}} \right) - {\mathrm{y}}
\end{eqnarray*}

The recovery error is calculated as the difference between the normalized value (x) and the renormalized value (z); see equation (10):


(10) \begin{eqnarray*}
{\mathrm{recovery}}\_{\mathrm{error}} = {\mathrm{x}} - {\mathrm{z}}
\end{eqnarray*}

In certain cases, not all cells can be successfully denormalized due to poor sequencing quality or a low number of expressed genes. To evaluate denormalization in such situations, we define success rate as the percentage of successfully denormalized cells; see equation (11).


(11) \begin{eqnarray*}
{\mathrm{success rate}} = {\mathrm{Nsuccess}}/{\mathrm{Ntotal}}
\end{eqnarray*}

Nsuccess is the number of successfully denormalized cells, while Ntotal is the total number of cells.

### Benchmark scDenorm based on digital precision and gene filtering

To assess the impact of different digital precision of normalized data on the denormalization process, we performed the following steps on the PBMC data. First, we applied total-count normalization (the normalize_total function in SCANPY [19]) to the data matrix with a target sum of 10,000 and log-transformed (natural base, e) the data with 1 as a pseudo-count. Next, we used the round function to retain the data at different levels of precision, ranging from 2 to 8. Float16 corresponds to 3 to 4 decimal places of precision, while float32 corresponds to 6 to 9 decimal places of precision. Finally, we denormalized the data separately for each precision level and compared the results with rounding errors to evaluate their effects.

To test our algorithm for gene filtering on extreme cases, we selected a series of highly variable genes, including 100, 200, 300, 400, 500, 1,000, 2,000, and 5000. Specifically, first, we normalized the data by sc.pp.normalize_total with target_sum as 10,000 and logarithmized the data with sc.pp.log1p. The high-variable genes were then selected using sc.pp.highly_variable_genes with layer as “count” and flavor as “seruat_v3.” Finally, we used scDenorm to denormalize the data and calculate the recovery errors.

### Benchmark on large-scale datasets

To evaluate our tool on atlas data, we downloaded 40 datasets from the UCSC Cell Browser and 60 datasets from the Brain Cell Atlas, ensuring that they encompass a diverse range of species, sequencing platforms, and normalization methods. First, we used scDenorm to denormalize each dataset. If successful, we calculated the rounding errors for the dataset, which quantifies the difference between the denormalized values before and after rounding. In addition, when the total expression values (i.e., the sum of all denormalized values within each cell) were close to a fixed target sum (e.g., 10,000) after detransformation, we further calculated the recovery error. Specifically, the datasets were renormalized using a target sum of 1e4, a pseudo-count of 1, and the natural logarithm base(e). The recovery error was calculated as the difference between the original normalized matrix and the renormalized matrix obtained after denormalization and renormalization.

### Dataset processing for data integration and downstream analysis

The COVID-19 PBMC dataset from Arunachalam et al. [37] (Fig. 5) was downloaded from GEO [48] under accession code GSE155673. Two samples, Arunachalam_cov11 (S1) and Arunachalam_cov11 (S2), were processed with different delta normalization parameters: S1 was normalized by target sum 1e3, while S2 was normalized by target sum 1e4. Both were log-transformed. For data visualization, we performed Harmony [13] data integration of these 2 samples after PCA of 50 components. For cell-type annotation, SCCAF [40] was used. S2 was used as the reference for annotating S1.

The human skin dataset from Solé-Boldo et al. [39] was downloaded from GEO under accession code GSE130973 (Fig. 6), including 2 young (25 and 27 years old) and 3 old (53, 69, and 70 years old) donors. The young samples were normalized to a target sum of 1e3, while the old samples were normalized to a target sum of 1e4. Both samples were logarithmically transformed after normalization. For cell-type annotation, the old samples were used as the reference for annotating the young sample.

The human prefrontal cortex data includes datasets from 2 studies, Ma et al. [38] (170,000 cells) and Velmeshev et al. [34] (100,000 cells) (Fig. 7).  Velmeshev’s dataset was normalized to a target sum of 1e3 and logarithmic transformation, while Ma’s dataset was not normalized. Harmony was used for data integration. For cell-type annotation, Ma’s dataset was used as the reference for annotating Velmeshev’s dataset.

For data processing after denormalization with scDenorm, we follow a standard workflow of data normalization and dimension reduction. Specifically, the expression matrix was normalized to a target sum of 10,000 and log-transformed. The default dimension reduction process in the SCANPY workflow was also used, including PCA, Harmony integration, and UMAP visualization. SCCAF was used to predict the cell types as described above.

Downstream analysis after cell-type annotation includes DE analysis and GO pathway analysis. Differential gene expression analysis was conducted for each cell type (one against the rest) using the Wilcoxon test implemented in SCANPY [19]. As part of the dataset is used as the reference dataset, the DEGs derived from the reference dataset were used as the gold standard in our evaluation. The same approach was used to calculate the DEGs before and after scDenorm. The top differentially expressed genes were compared across different thresholds (top 50, 100, 200, 500, and 1,000). For GO pathway analysis, the enrichGO program was used on the top 500 differentially expressed genes.

DE and GO analyses with the correct labels from Solé-Boldo et al. [39] were conducted for each cell type (one against the rest) using the Wilcoxon test implemented in Seurat (V3.1.1), the same version described in Solé-Boldo et al. [39]. These analyses were conducted before scDenorm and after scDenorm. The differentially expressed genes were obtained from the study’s supplementary materials as the gold standard.

## Availability of Source Code and Requirements

Project name: scDenorm

Project homepage: https://github.com/rnacentre/scDenorm

License: Apache-2.0 license

Operating system: Linux

Programming language: Python

Package management: pip-https://pypi.org/project/scDenorm/; anaconda-https://anaconda.org/changebio/scdenorm

Hardware requirements: No requirements

biotools: scdenorm

RRID:SCR_027574

Codes for reproducing this work: https://github.com/rnacentre/scDenorm_reproducibility

## Supplementary Material

giag032_Supplemental_File

giag032_Authors_Response_To_Reviewer_Comments_original_submission

giag032_Authors_Response_To_Reviewer_Comments_revision_1

giag032_GIGA-D-25-00209_original_submission

giag032_GIGA-D-25-00209_Revision_1

giag032_GIGA-D-25-00209_Revision_2

giag032_Reviewer_1_Report_original_submission Reviewer 1 -- 7/18/2025

giag032_Reviewer_1_Report_revision_1 Reviewer 1 -- 1/22/2026

giag032_Reviewer_2_Report_original_submission Reviewer 2 -- 7/19/2025

giag032_Reviewer_2_Report_revision_1 Reviewer 2 -- 11/18/2025

giag032_Reviewer_3_Report_original_submission Reviewer 3 -- 8/7/2025

giag032_Reviewer_3_Report_revision_1 Reviewer 3 -- 11/18/2025

giag032_Reviewer_3_Report_revision_2 Reviewer 3 -- 3/9/2026

## Data Availability

The 10×3k PBMC data were downloaded from the 10x Genomics website [49]. The dataset with both the normalized expression and the raw count matrix was downloaded from the UCSC Cell Browser autism dataset [50]. Forty processed datasets (Supplementary Table S1) were downloaded from the UCSC Cell Browser [51]. Sixty processed datasets (Supplementary Table S2) were downloaded from the Brain Cell Atlas [52]. Datasets used in the article have been deposited at Zenodo [53].

## Additional Files

**Supplementary Fig. S1**. Inconsistent data normalization generates bias in data integration. (a–h) Each plot shows the UMAP based on PCA space from data obtained with different normalization methods, including different combinations of library size (L), base (b), and pseudo-count (p). (i, j) UMAP plots the BBKNN-integrated result on the data obtained from different normalization methods after BBKNN integration, colored by different cell types (i) and different normalization methods (j). (k, l) UMAP plots show the Scanorama-integrated result on the data obtained from different normalization methods after Scanorama integration, colored by different cell types (k) and different normalization methods (l).

**Supplementary Fig. S2**. Features of droplet-based single-cell data. (a) The relationship between the log-transformed mean expression (x-axis) and the log-transformed variance (y-axis) for each gene in the count matrix from the study (Satija et al., 2015). (b) The relationship between the rank of the count frequency (x-axis) and the count value (y-axis) in cells. (c) The zoom-in view of panel (b). (d) The change of the percentage of cells with the value of count equal to its rank in 3 scRNA-seq technologies (Chromium, Smart-seq2, and Drop-seq).

**Supplementary Fig. S3**. Schematic representation of the scDenorm algorithm. (a) Standard preprocessing: routinely, droplet-based single-cell data are first normalized by a size factor (s, usually total count with a library size factor) with a pseudo-count (p) and then transformed by a logarithmic function with a base (b) for downstream analysis. (b) Denormalization: scDenorm performs the reverse of the task above. First, it reverses the logarithmic transformation by searching for common bases (e.g., 2, e, 10) and common pseudo-counts (e.g., 0.01, 0.1, 1) or auto-detecting the base and pseudo-count. Then, it reverses the scaling by determining the scaling factor for each cell. (c) The formulas and derivations of the automatic detection method for base and pseudo-count during the detransformation process. b is the base, p is the pseudo-count, s is the scaling factor, and N_1_ and N_2_ are the most and second frequency values for each cell, respectively. (d) The formulas for solving the scaling factor for each cell during the unscaling process. Equation (4) is the formula of the regression-based method, and equation (5) is the formula of the top 2 rank equation method. c_i_ are the ranks of the frequency of normalized values (c_1_ is 1, c_2_ is 2, etc). x_i_ are the values sorted by frequency in decreasing order after detransformation.

**Supplementary Fig. S4**. The computational efficiency of scDenorm.(a) The boxplot shows the time distribution to calculate the scaling factors for cells by 2 unscaling methods: the top 2 rank equation method and the regression-based method. (b) The histogram shows the percentage of cells that were successfully denormalized by the 2 methods. (c) The distribution of execution times for denormalization (y-axis, the unit is seconds) across cells with genes ranging from 2,000 to 10,000 (x-axis). (d) The distribution of memory usage during denormalization (y-axis, the unit is GB) across cells ranging from 10,000 to 100,000 (x-axis).

**Supplementary Fig. S5**. The relationship between the mean and the variance of genes after denormalization. Scatterplots show the relationship between log-transformed mean expression (x-axis) and log-transformed variance (y-axis) for each gene after denormalization, across different scRNA-seq datasets from UCSC Cell Browser. Each dot represents a gene, with titles indicating the dataset names.

**Supplementary Fig. S6**. The relationship between the rounding errors and values after denormalization. Scatterplots illustrate the relationship between count values (x-axis) and rounding errors (y-axis) after denormalization for different scRNA-seq datasets from the UCSC Cell Browser. Each dot represents a cell, with titles indicating dataset names.

**Supplementary Fig. S7**. Benchmark analysis on the datasets from the Brain Cell Atlas. (a) The bar plot shows the number of genes in each dataset. The x-axis is the name of datasets (same as d), and the y-axis is the log-scaled number of genes. (b) The bar plot shows the number of cells in each dataset. The x-axis is the name of datasets (same as d), and the y-axis is the log-scaled number of cells. (c) The bar plot shows the success rate for each dataset. The x-axis is the name of datasets (same as d), and the y-axis is the success rate (see Method). (d) The jitter plot shows the distribution of rounding errors observed in the denormalized datasets from the Brain Cell Atlas. The x-axis is the name of the datasets, and the y-axis is the rounding error.

**Supplementary Fig. S8**. The robustness of scDenorm recovering raw counts in different scenarios. (a) The relationship between the count values and the rounding errors after denormalization on 2-digit precision data. (b) The mean square error of recovery error after denormalization on the digit precisions from 2 to 8. (c) UMAP plot with different colors to represent different digit precisions, including original data, 2-digit precision data, and 3-digit precision data. (d) UMAP plot (same as c) with different colors to represent different cell types.

**Supplementary Fig. S9**. The relationship between the rounding errors and count values in different numbers of the top highly variable genes. The scatterplots show the relationship between count values (x-axis) and rounding errors (y-axis) after denormalization for different numbers of the top highly variable genes. Each dot is a cell. The titles are the number of the top highly variable genes.

**Supplementary Fig. S10**. The integration and downstream analyses on COVID-19 PBMC datasets.(a) The UMAP plots show the distribution of cells of the COVID-19 PBMC datasets before integration without the scDenorm denormalization, colored by sample (top), and original cell type from Arunachalam et al. (bottom). (b) The UMAP plots show the Harmony-integrated results without the scDenorm denormalization, colored by sample (top), and original cell type from Arunachalam et al. (bottom). (c) The UMAP plots show the Harmony-integrated results after running scDenorm, colored by sample (top), and original cell type from Arunachalam et al. (bottom). (d) Bar plot shows the percentage difference of DEGs before and after scDenorm across cell types. The top DEGs were compared across different thresholds (top 50, 100, 200, 500, and 1,000). (e) Bar plot shows the overlapping percentage of GO terms between the gold standard and before and after scDenorm across cell types. (f) Venn diagram shows the overlap of GO terms for HSPC between the gold standard and before and after scDenorm.

**Supplementary Fig. S11**. scDenorm facilitates the downstream analyses on young and old human skin datasets.(a) The UMAP plot shows the cell distribution of the human skin datasets before integration without the scDenorm denormalization, colored by sample. (b) The UMAP plot shows the Harmony-integrated result without the scDenorm denormalization, colored by sample. (c) The UMAP plot shows the Harmony-integrated result after running scDenorm, colored by sample. (d) River plot illustrates the transition between the original and predicted cell types before scDenrom. The left side represents the original cell types from Solé-Boldo et al., while the right side displays the predicted cell types. (e) River plot illustrates the transition between original and predicted cell types after scDenrom. The left side represents the original cell types from Solé-Boldo et al., while the right side displays the predicted cell types. (f) Heatmap shows the enriched Gene Ontology (GO) terms for the keratinocyte’s DEGs identified between the gold standard and before and after scDenorm.

**Supplementary Table S1**. Description of the datasets from the UCSC Cell Browser.

**Supplementary Table S2**. Description of the datasets from the Brain Cell Atlas.

**Supplementary Table S3**. Summary of the normalization methods on 133 well-established single-cell studies.

## Abbreviations

BFGS: Broyden–Fletcher–Goldfarb–Shanno; DE: differential expression; DEG: differentially expressed gene; GO: Gene Ontology; HCA: Human Cell Atlas; HSPC: hematopoietic stem and progenitor cell; MAE: mean absolute error; MSE: mean square error; NK: natural killer; PBMC: peripheral blood mononuclear cell; PCA: principal component analysis; pDC: plasmacytoid dendritic cell; PFC: prefrontal cortex; scRNA-seq: single-cell RNA sequencing; snRNA-seq: single-nucleus RNA sequencing; TPM: transcripts per million; UMAP: Uniform Manifold Approximation and Projection.
