# Supplementary material for: scDenorm: a denormalization tool for integrating single-cell transcriptomics data
Source: Gigascience. 2026 Mar 31;15:giag032. doi: 10.1093/gigascience/giag032 (PMC13142155; doi:10.1093/gigascience/giag032)
Supplement: giag032_Supplemental_File [file giag032_supplemental_file.docx]

Supplementary Information

scDenorm: a denormalisation tool for integrating single-cell transcriptomics data

Yin Huang^1,2^, Anna Vathrakokili Pournara^4^, Ying Ao^3^, Ziliang Huang^2^, Hui Zhang^5^, Yongjian Zhang^6^, Sheng Liu^7,8^, Alvis Brazma^4^, Irene Papatheodorou^4^, Xinlu Yang^5,*^, Ming Shi^9,*^, Zhichao Miao^2,3,*^

^1^ Translational Research Institute of Brain and Brain-Like Intelligence and Department of Anesthesiology, Shanghai Fourth People’s Hospital Affiliated to Tongji University School of Medicine, Shanghai, China

^2^Guangzhou National Laboratory, Guangzhou International Bio Island, Guangzhou 510005, China

^3^ GMU-GIBH Joint School of Life Sciences, Guangzhou Medical University, Guangzhou, China

^4^ European Molecular Biology Laboratory, European Bioinformatics Institute, EMBL-EBI, Wellcome Genome Campus, Cambridge CB10 1SD, UK

^5^ Department of Obstetrics and Gynaecology, Harbin Red Cross Central Hospital, Harbin 150001, China.

^6^ Department of Surgery Oncology, Harbin Medical University Cancer Hospital, Harbin 150001, China

^7^ State Key Laboratory of Ophthalmology, Zhongshan Ophthalmic Center, Sun Yat-sen University, Guangdong Provincial Key Laboratory of Ophthalmology and Visual Science, Guangzhou, China

^8^ Guangdong Province Key Laboratory of Brain Function and Disease, Guangzhou, China

^9^ School of Life Science and Technology, Harbin Institute of Technology, Harbin 150001, China

* correspondence should be addressed to Zhichao Miao, [miao_zhichao@gzlab.ac.cn](mailto:miao_zhichao@gzlab.ac.cn), Ming Shi, [shiming@hit.edu.cn](mailto:shiming@hit.edu.cn), and Xinlu Yang, [519950370@qq.com](mailto:519950370@qq.com)

# Supplementary Figures

##
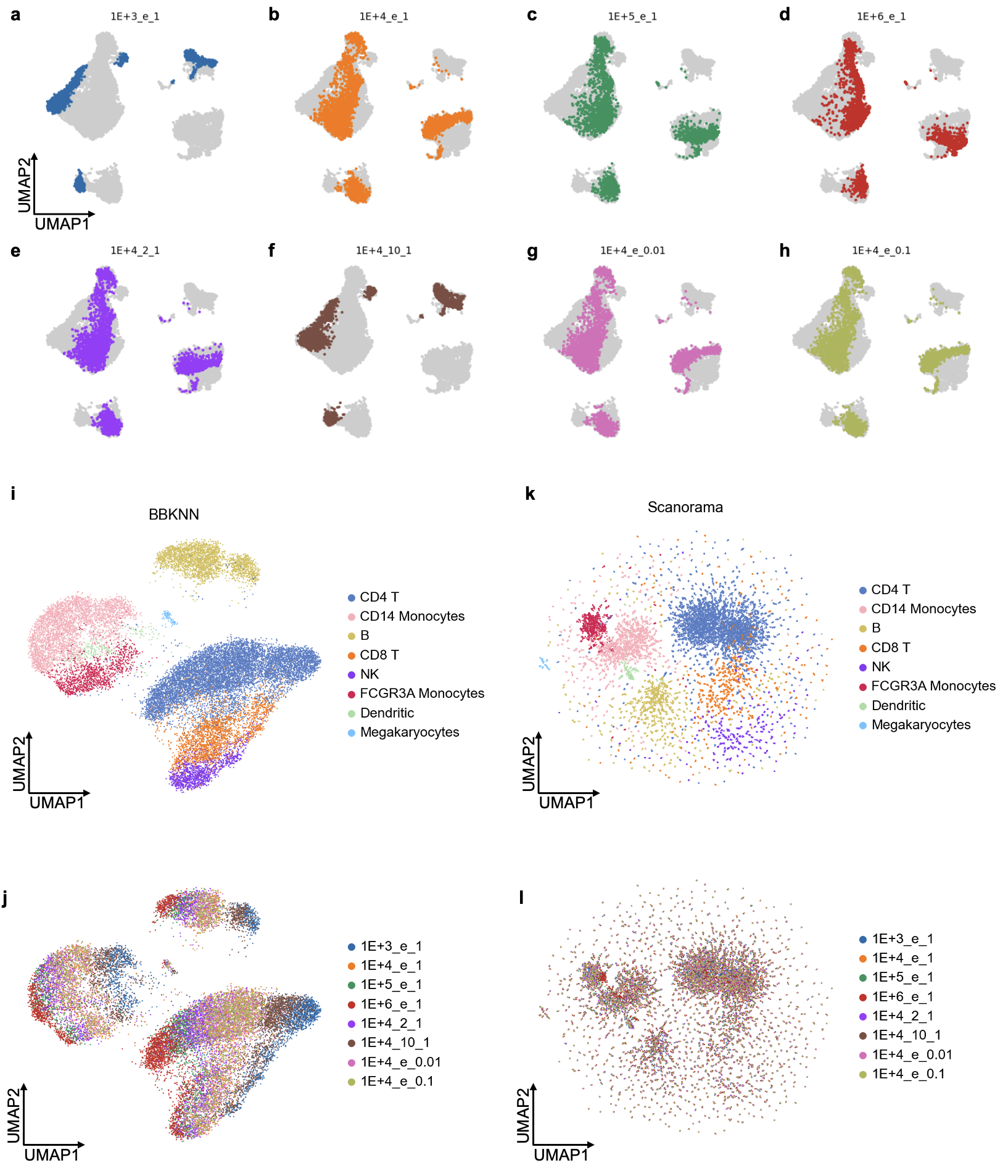


## Supplementary Fig. 1 Inconsistent data normalisation generates bias in data integration

**a-h**, Each plot shows the UMAP based on PCA space from data obtained with different normalization methods, including different combinations of library size (L), base (b) and pseudo count (p).

**i-j**, UMAP plots the BBKNN-integrated result on the data obtained from different normalization methods after BBKNN integration, coloured by different cell types (**i**) and different normalization methods (**j**).

**k-l**, UMAP plots show the scanorama-integrated result on the data obtained from different normalization methods after Scanorama integration, coloured by different cell types (**k**) and different normalization methods (**l**).


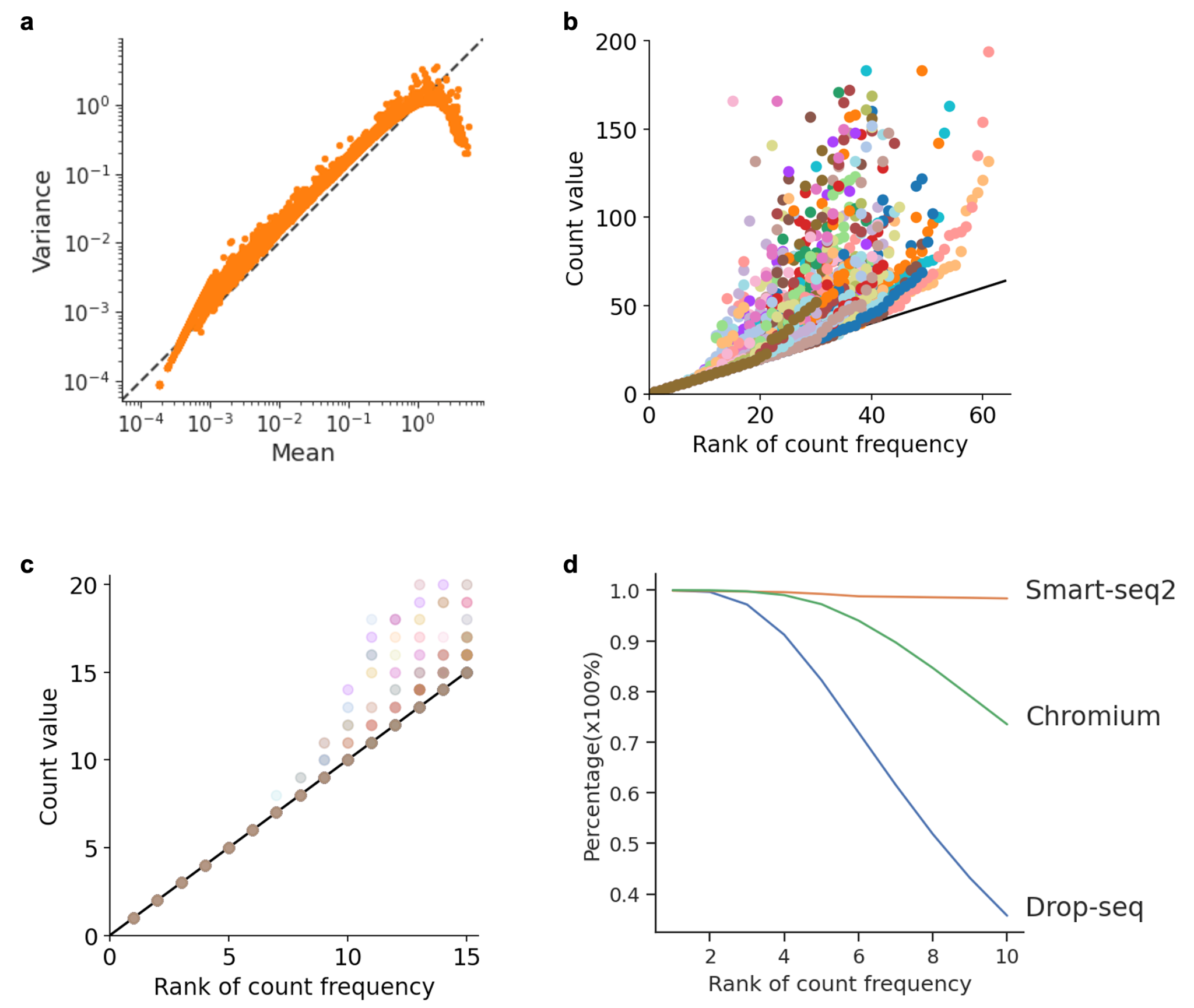


## Supplementary Fig. 2 Features of droplet-based single-cell data

**a**, The relationship between the log-transformed mean expression (x-axis) with the log-transformed variance (y-axis) for each gene in the count matrix from the study (Satija et al., 2015).

**b**, The relationship between the rank of the count frequency (x-axis) with the count value (y-axis) in cells.

**c**, the zoom-in view of panel (b).

**d**, The change of the percentage of cells with the value of count equal to its rank in three scRNA-seq technologies (Chromium, Smart-seq2, and Drop-seq).

##
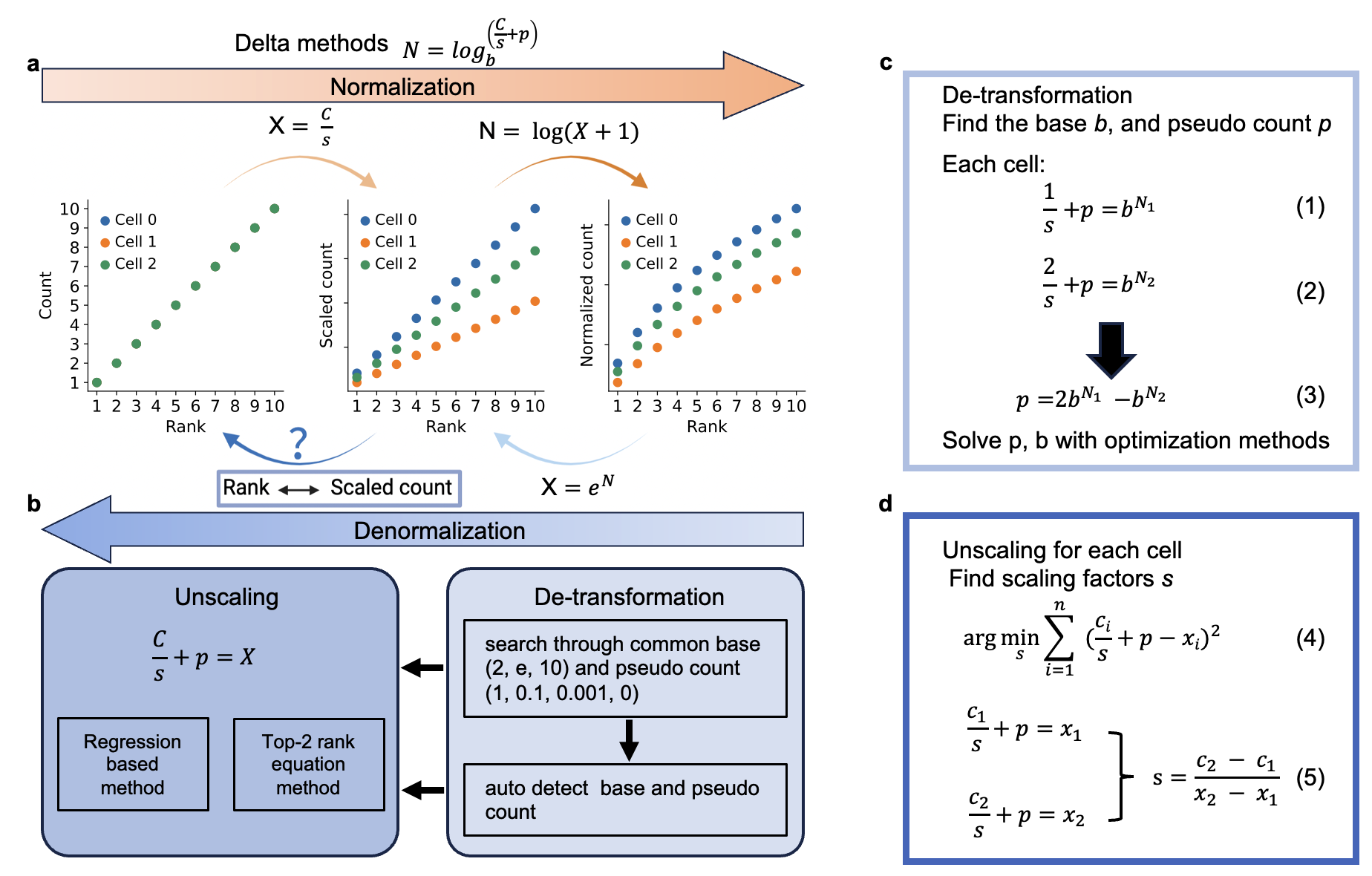


## Supplementary Fig. 3 Schematic representation of scDenorm algorithm

**a,** Standard pre-processing: routinely droplet-based single-cell data are first normalised by a size factor (s, usually total count with a library size factor) with and pseudo count (p) secondly transformed by a logarithmic function with a base (b) for downstream analysis.

**b,** Denormalisation: scDenorm performs the reverse of the task above. First it reverses the logarithmic transformation by searching for common bases (e.g., 2, e, 10) and common pseudo counts (e.g., 0.01, 0.1, 1), or auto detect base and pseudo count. Then, it reverses the scaling by determining the scaling factor for each cell.

**c**, The formulas and derivations of automatic detection method for base and pseudo count during the de-transformation process. b is the base, p is the pseudo count, s is the scaling factor, and N_1_ and N_2_ are the most and second frequency values for each cell.

**d**, The formulas of solving the scaling factor for each cell during the unscaling process. Equation (4) is the formula of the regression based method, and equation (5) is the formula of the top-2 rank equation method. c_i_ are the ranks of the frequency of normalised values ( c_1_ is 1, and c_2_ is 2, etc). x_i_ are the values sorted by frequency in a decreasing order after de-transformation.

##
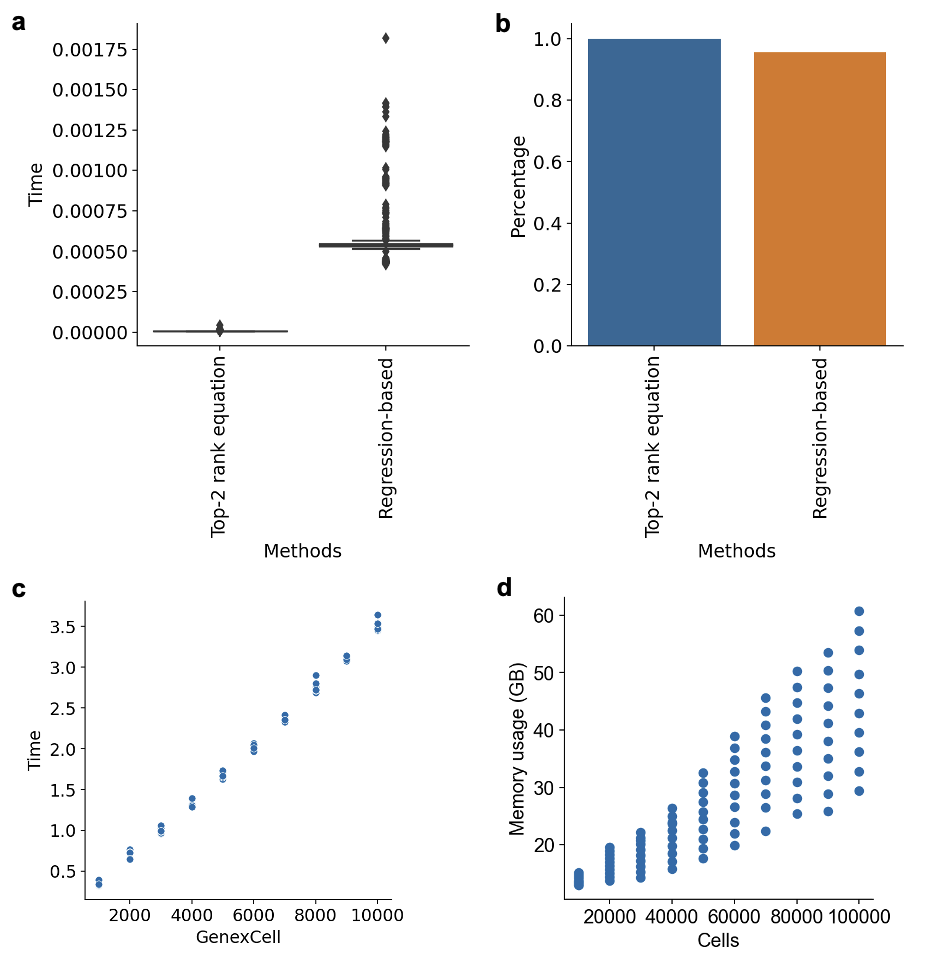


## Supplementary Fig. 4 The computational efficiency of scDenorm

**a**, The boxplot shows the time distribution to calculate the scaling factors for cells by two unscaling methods, the top-2 rank equation method and the regression based method.

**b**, The histogram shows the percentage of cells that were successfully denormalised by the two methods.

**c**, The distribution of execution times for denormalisation (y-axis, the unit is second) across cells with genes ranging from 2000 to 10000 (x-axis).

**d**, The distribution of memory usage during denormalisation (y-axis, the unit is GB) across cells ranging from 10000 to 100,000 (x-axis).

##
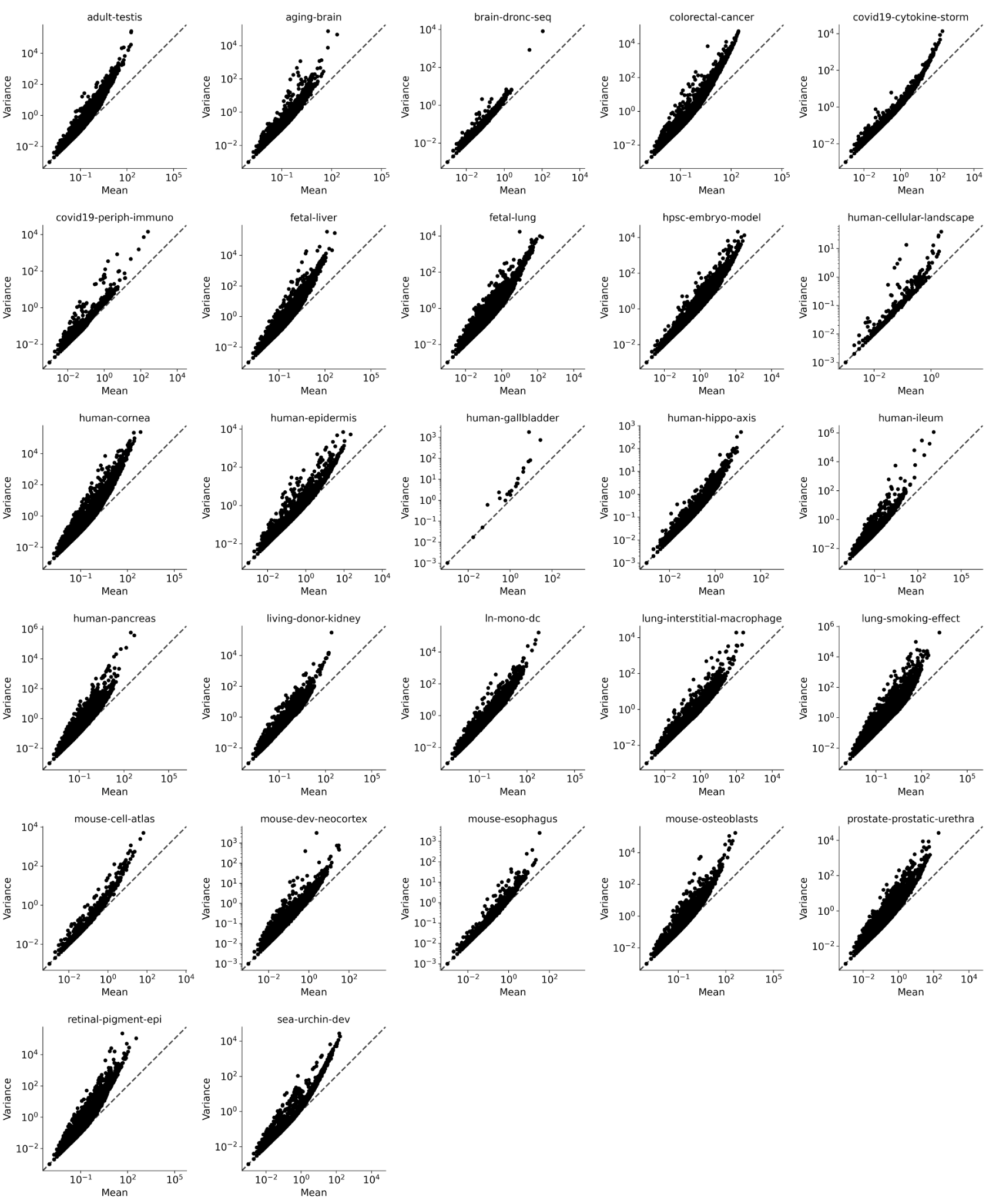
Supplementary Fig. 5 The relationship between the mean and the variance of genes after denormalisation

Scatter plots show the relationship between log-transformed mean expression (x-axis) with log-transformed variance (y-axis) for each gene after denormalisation, across different scRNA-seq datasets from UCSC Cell Browser. Each dot represents a gene, with titles indicating the dataset names.

##
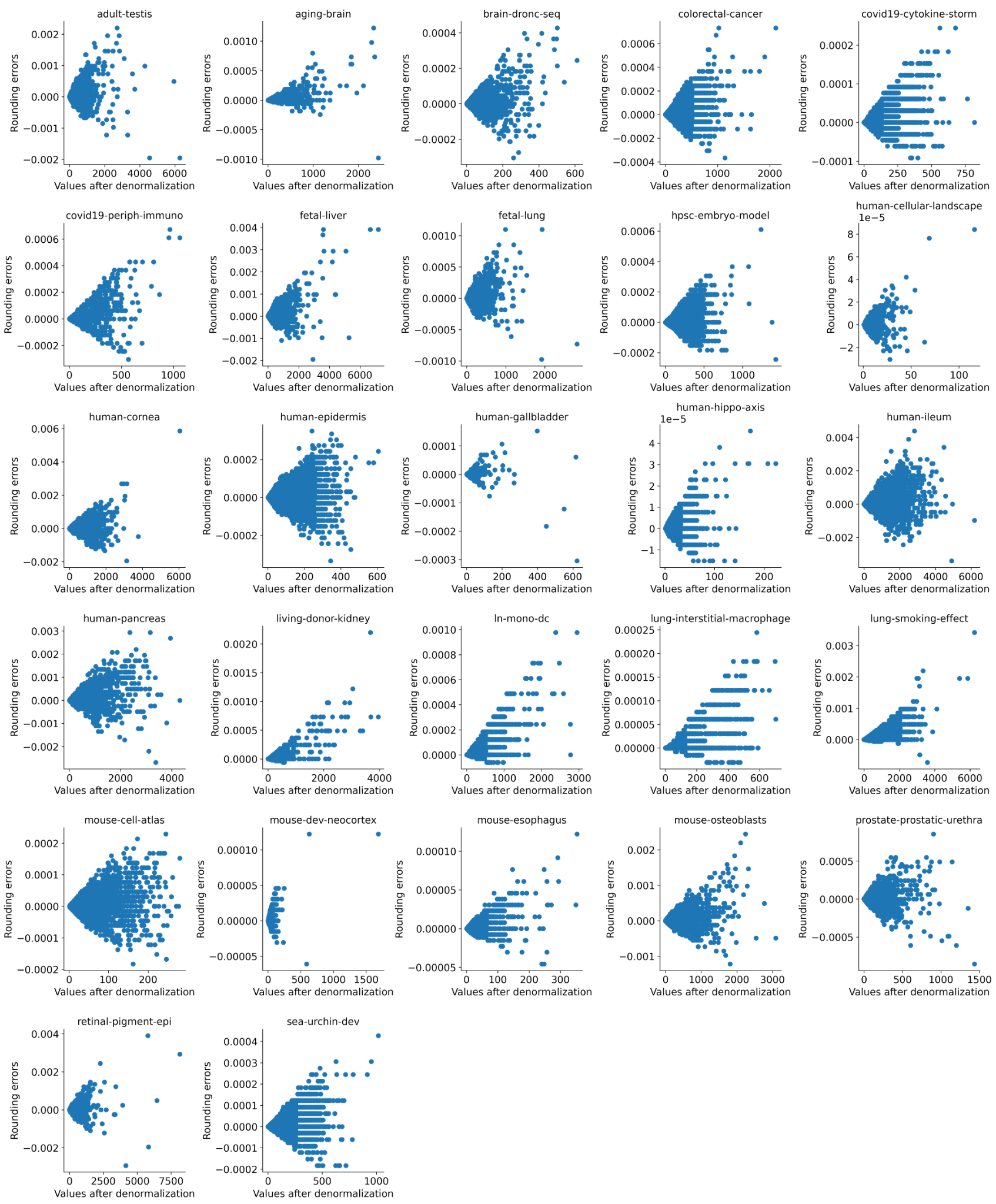
Supplementary Fig. 6 The relationship between the rounding errors and values after denormalisation

Scatter plots illustrate the relationship between count values (x-axis) and rounding errors (y-axis) after denormalisation for different scRNA-seq datasets from UCSC Cell Browser. Each dot represents a cell, with titles indicating dataset names.


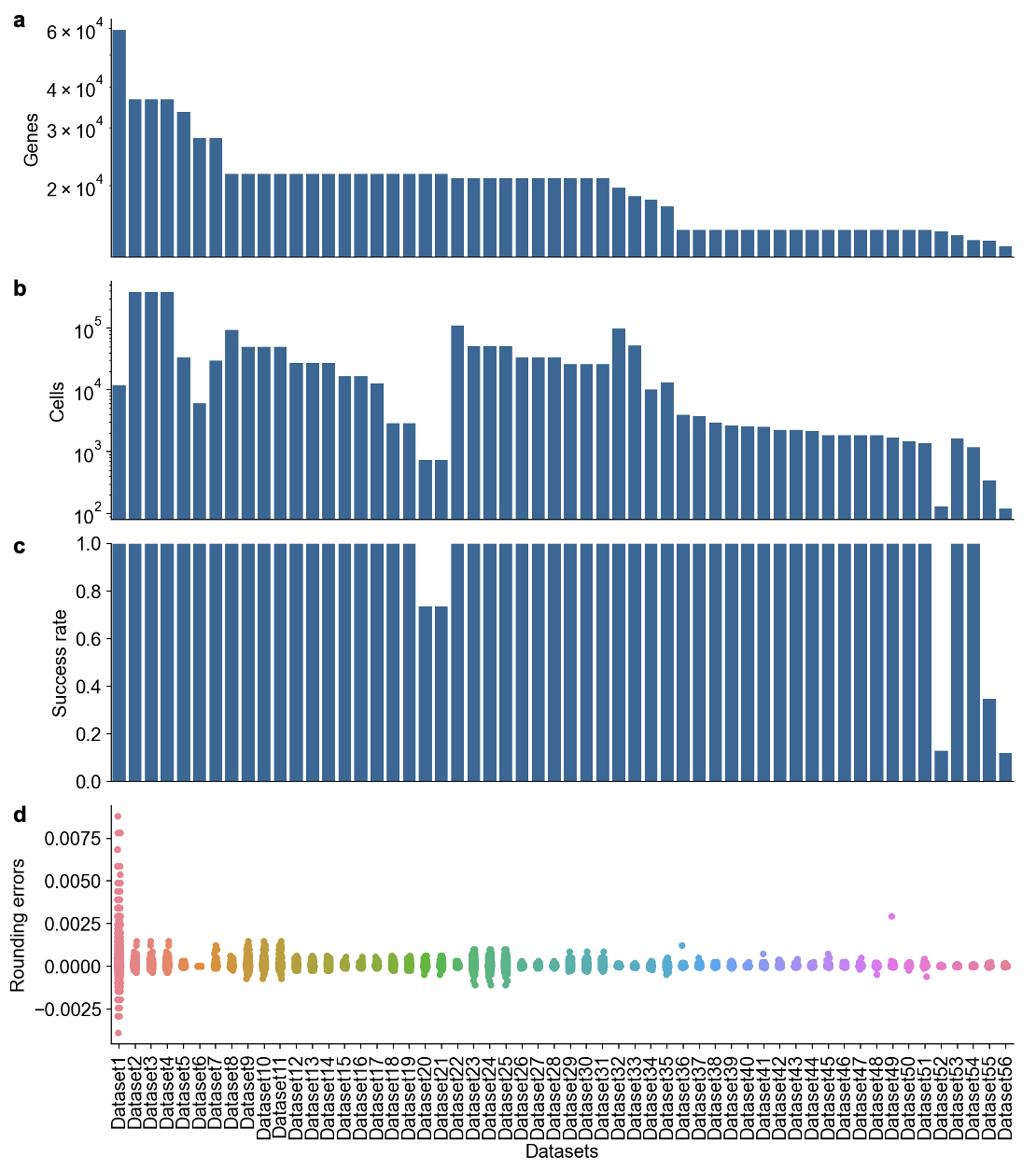


## Supplementary Fig. 7 Benchmark analysis on the datasets from Brain Cell Atlas

**a,** The bar plot shows the number of genes in each dataset. The x-axis is the name of datasets (same as **d**), and the y-axis is the log-scaled number of genes.

**b,** The bar plot shows the number of cells in each dataset. The x-axis is the name of datasets (same as **d**), and the y-axis is the log-scaled number of cells.

**c,** The bar plot shows the success rate for each dataset. The x-axis is the name of datasets (same as **d**), and the y-axis is the success rate (see **Method**).

**d,** The jitter plot shows the distribution of rounding errors observed in the denormalised datasets from the Brain Cell Atlas. The x-axis is the name of datasets, and the y-axis is the rounding error.


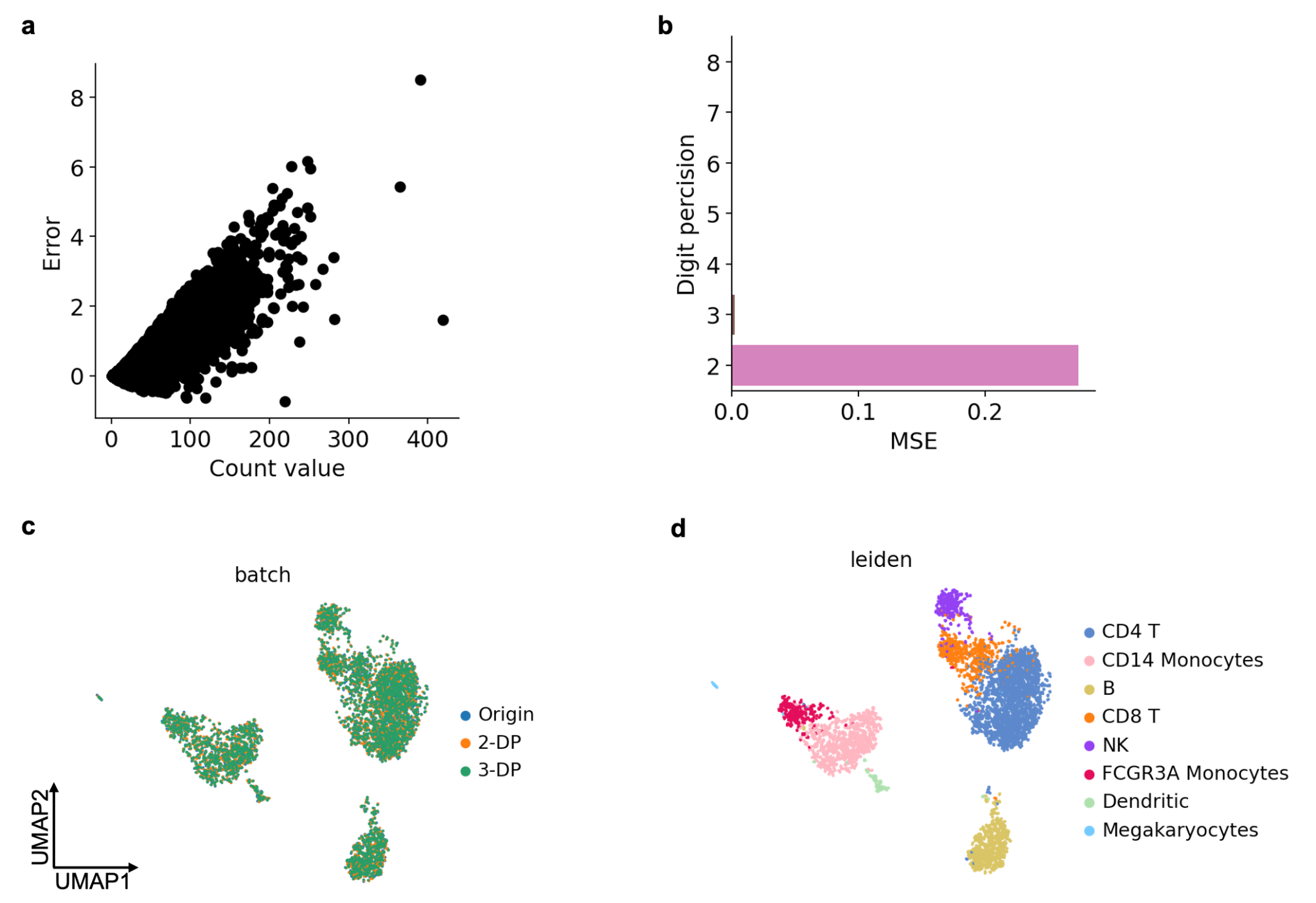


## Supplementary Fig. 8 The robustness of scDenorm recovering raw counts in different scenarios

**a**, The relationship between the count values and the rounding errors after denormalisation on 2 digit precision data.

**b**, The mean square error of recovery error after denormalisation on the digit precisions from 2 to 8.

**c**, UMAP plot with different colors to represent different digit precisions, including original data, 2 digital precision data and 3 digital precision data.

**d**, UMAP plot (same as **c**) with different colors to represent different cell types.

##
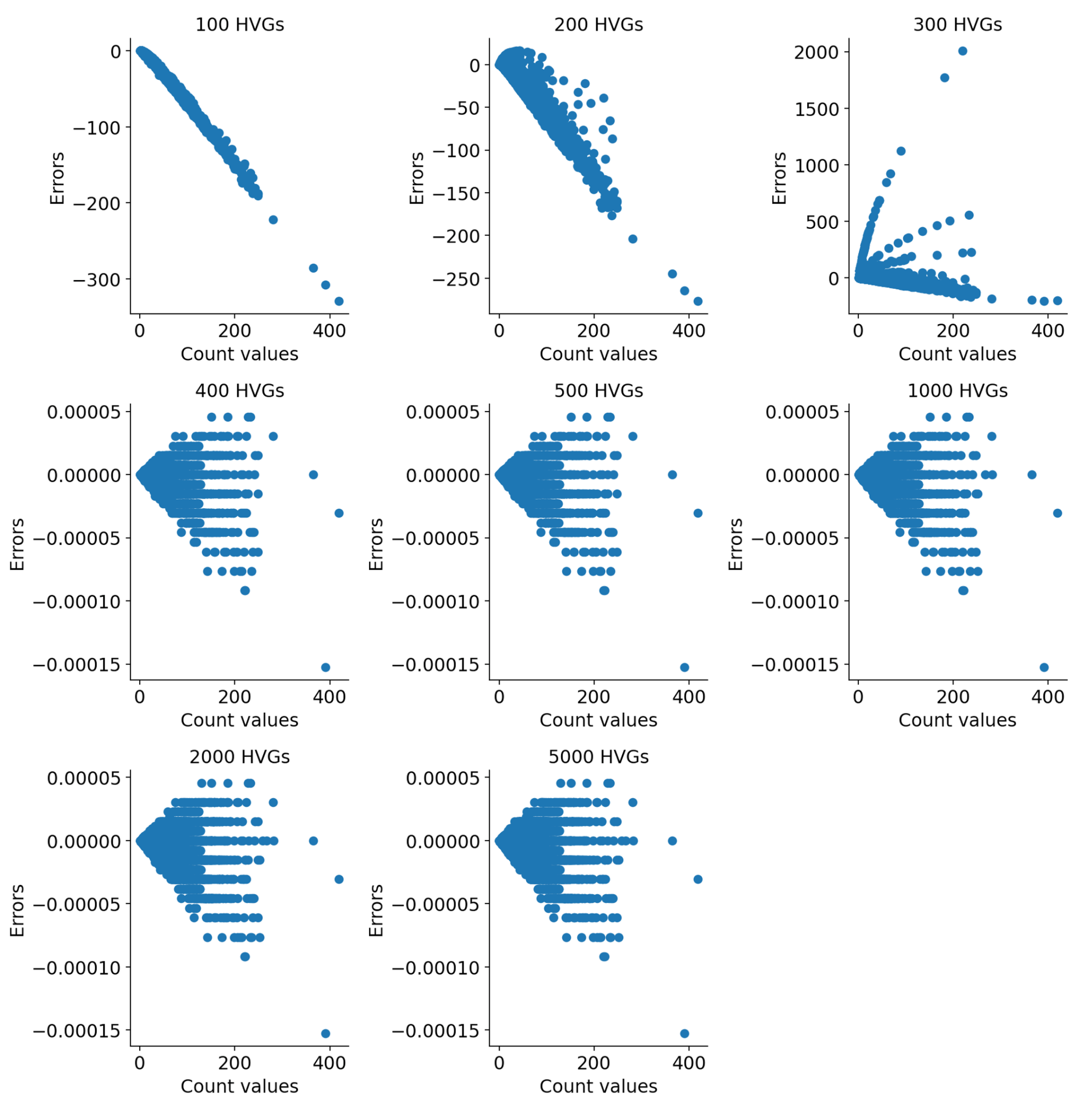
Supplementary Fig. 9 The relationship between the rounding errors and count values in different numbers of top highly variable genes

The scatter plots show that The relationship between count values (x-axis) and rounding errors (y-axis) after denormalisation, for different numbers of top highly variable genes. Each dot is a cell. The titles are the number of top highly variable genes.


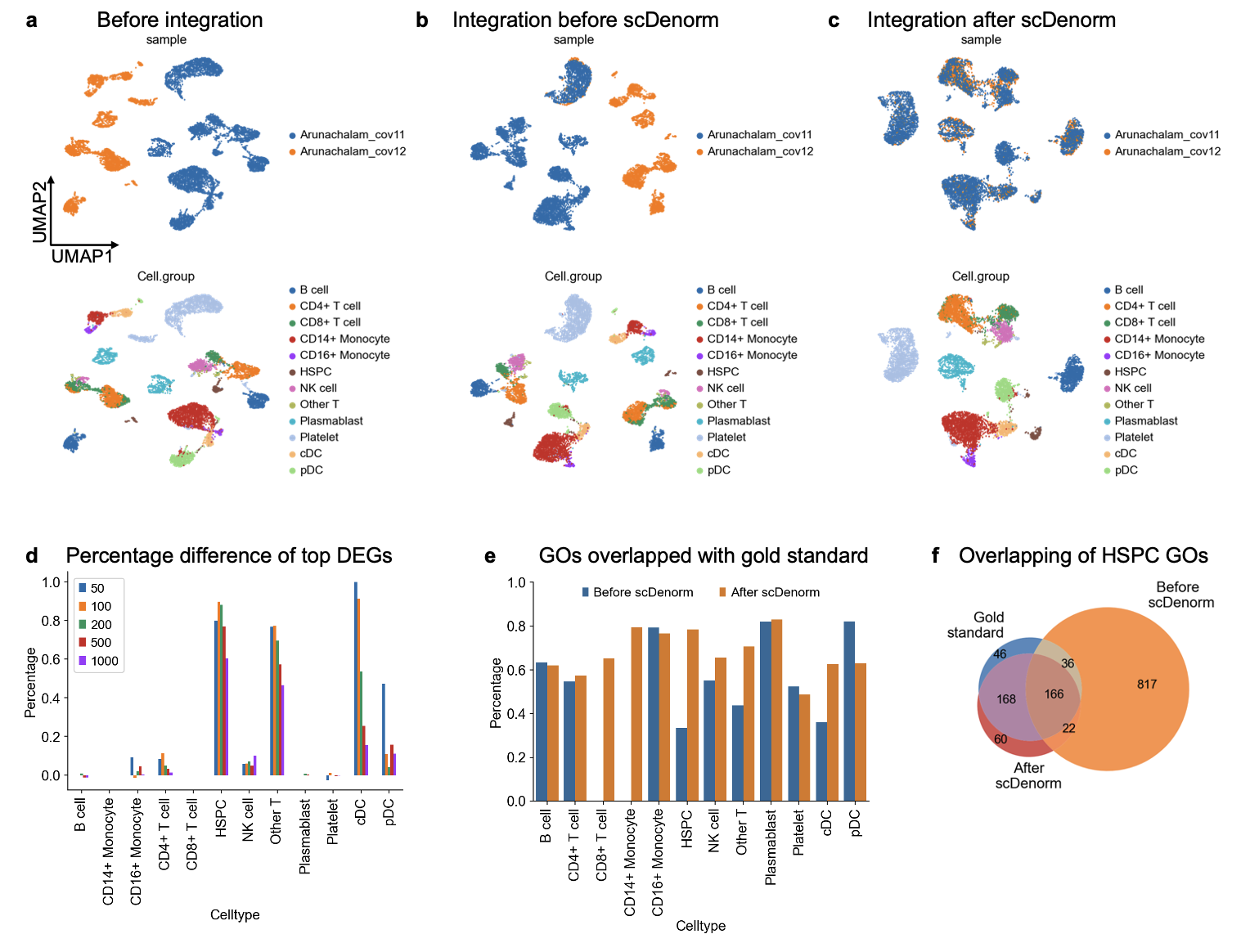


## Supplementary Fig. 10 The integration and downstream analyses on COVID-19 PBMC datasets

**a,** The UMAP plots show the distribution of cells of the COVID-19 PBMC datasets before integration without the scDenorm denormalisation, coloured by sample (top) , and original cell type from Arunachalam et al. (bottom).

**b,** The UMAP plots show the Harmony-integrated results without the scDenorm denormalisation, coloured by sample (top) , and original cell type from Arunachalam et al. (bottom).

**c,** The UMAP plots show the Harmony-integrated results after running scDenorm, coloured with sample (top) , and original cell type from Arunachalam et al. (bottom).

**d,** Bar plot shows the percentage difference of DEGs before and after scDenorm across cell types. The top DEGs were compared across different thresholds (top 50, 100, 200, 500, and 1000).

**e,** Bar plot shows the overlapping percentage of GO terms between the gold standard with before and after scDenorm across cell types.

**f,** Venn diagram shows the overlap of GO terms for HSPC among the gold standard, before scDenorm, and after scDenorm.

##


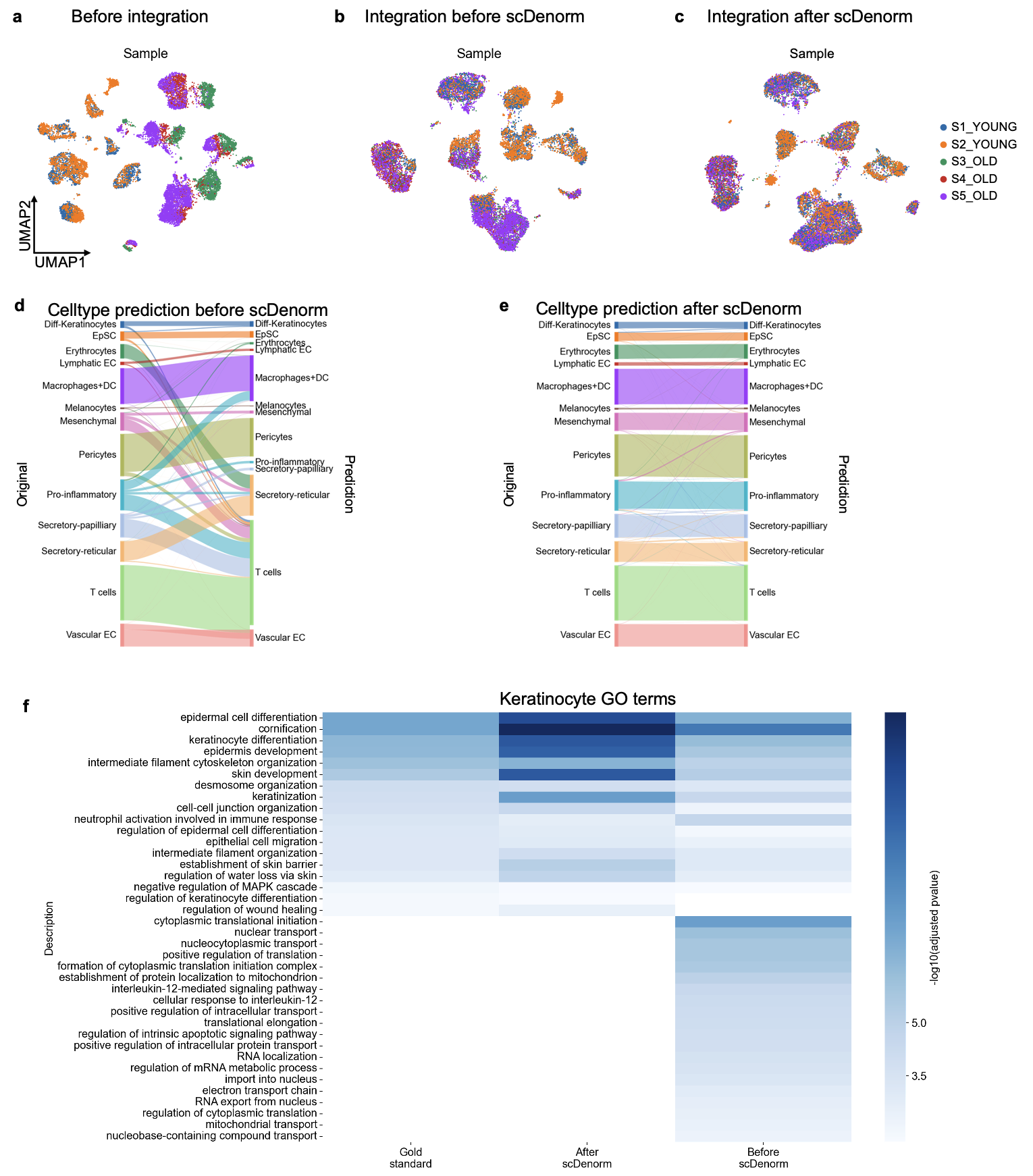


## Supplementary Fig. 11 scDenorm facilitates the downstream analyses on young and old human skin datasets

**a,** The UMAP plot shows the cell distribution of the human skin datasets before integration without the scDenorm denormalisation, coloured with samples.

**b,** The UMAP plot shows the Harmony-integrated result without the scDenorm denormalisation, coloured by samples.

**c,** The UMAP plot shows the Harmony-integrated result after running scDenorm, coloured by samples.

**d,** River plot illustrates the transition between original and predicted cell types before scDenrom. The left side represents the original cell types from Solé-Boldo et al., while the right side displays the predicted cell types.

**e,** River plot illustrates the transition between original and predicted cell types after scDenrom. The left side represents the original cell types from Solé-Boldo et al., while the right side displays the predicted cell types.

**f,** Heatmap shows the enriched Gene Ontology (GO) terms for the keratinocyte’s DEGs identified in the gold standard, before scDenorm as well as after scDenorm.

# Supplementary Tables

## Supplementary Table 1. Description of the datasets from UCSC Cell Browser.

## Supplementary Table 2. Description of the datasets from Brain Cell Atlas.

## Supplementary Table 3. Summary of the normalisation methods on 133 well-established single-cell studies.
