# Supplementary material for: scDenorm: a denormalization tool for integrating single-cell transcriptomics data
Source: Gigascience. 2026 Mar 31;15:giag032. doi: 10.1093/gigascience/giag032 (PMC13142155; doi:10.1093/gigascience/giag032)
Supplement: giag032_GIGA-D-25-00209_Revision_1 [file giag032_giga-d-25-00209_revision_1.pdf]

GigaScience

scDenorm: a denormalisation tool for integrating single-cell transcriptomics data

--Manuscript Draft--

**Manuscript Number:**	GIGA-D-25-00209R1
**Full Title:**	scDenorm: a denormalisation tool for integrating single-cell transcriptomics data
**Article Type:**	Research
**Funding Information:**	the National Key R&D Programs of China (2023YFF1204700) — Prof. Zhichao Miao<br>the National Key R&D Programs of China (2024YFF1206600) — Prof. Zhichao Miao<br>the Natural Science Foundation of China (32270707) — Prof. Zhichao Miao<br>the Major Project of Guangzhou National Laboratory (GZNL2024A01002) — Prof. Zhichao Miao<br>the Major Project of Guangzhou National Laboratory (GZNL2023A01006) — Prof. Zhichao Miao<br>the R&D Programs of Guangzhou National Laboratory (HWYQ23-003) — Prof. Zhichao Miao<br>the R&D Programs of Guangzhou National Laboratory (YW-YFYJ0102) — Prof. Zhichao Miao<br>Postdoctoral Research Project Funding of Guangzhou (BSHF23-049) — Dr Yin Huang
**Abstract:**	Integrating single-cell omics data at an atlas scale enhances our understanding of cell types and disease mechanisms. However, the integration of data processed by different normalisation methods can lead to biases, such as unexpected batch effects and gene expression distortion, leading to misinterpretations in downstream analysis. To address these challenges, we present scDenorm, an algorithm that reverts normalised single-cell omics data to raw counts, preserving the integrity of the original measurements and ensuring consistent data processing during integration. We evaluated scDenorm's performance on large-scale datasets and benchmarked its impact on data integration and downstream analysis across three datasets.
**Corresponding Author:**	Zhichao Miao<br>Guangzhou Laboratory<br>Guangzhou, Guangdong CHINA
**Corresponding Author Secondary Information:**	
**Corresponding Author's Institution:**	Guangzhou Laboratory
**Corresponding Author's Secondary Institution:**	
**First Author:**	Zhichao Miao
**First Author Secondary Information:**	
**Order of Authors:**	Zhichao Miao<br>Yin Huang<br>Anna Vathrakokili Pournara<br>Ying Ao<br>Ziliang Huang<br>Hui Zhang

Powered by Editorial Manager® and ProduXion Manager® from Aries Systems Corporation

	Yongjian Zhang
	Sheng Liu
	Alvis Brazma
	Irene Papatheodorou
	Xinlu Yang
	Ming Shi
**Order of Authors Secondary Information:**	
**Response to Reviewers:**	Dear Nogoy,<br><br>We gratefully appreciate your feedback and all the comments from the reviewers. The constructive feedback helped us to improve our manuscript. We have carefully addressed the concerns raised by the reviewers. Please find our point-by-point responses below:<br><br><br>0.0 Your manuscript "scDenorm: a denormalisation tool for integrating single-cell transcriptomics data" (GIGA-D-25-00209) has been assessed by our reviewers. Although it is of interest, we are unable to consider it for publication in its current form. The reviewers have raised a number of points which we believe would improve the manuscript and may allow a revised version to be published in GigaScience.<br><br>Their reports, together with any other comments, are below. Please also take a moment to check our website at https://www.editorialmanager.com/giga/ for any additional comments that were saved as attachments.<br><br>In addition, please register any new software application in the bio.tools and SciCrunch.org databases to receive RRID (Research Resource Identification Initiative ID) and biotoolsID identifiers, and include these in your manuscript. Computational workflows should be registered in workflowhub.eu and the DOIs cited in the relevant places in the manuscript. These will facilitate tracking, reproducibility and re-use of your tool.<br><br>If you are able to fully address these points, we would encourage you to submit a revised manuscript to GigaScience. Once you have made the necessary corrections, please submit online at:<br><br>https://www.editorialmanager.com/giga/<br><br>If you have forgotten your username or password please use the "Send Login Details" link to get your login information. For security reasons, your password will be reset.<br><br>Please include a point-by-point within the 'Response to Reviewers' box in the submission system. Please ensure you describe additional experiments that were carried out and include a detailed rebuttal of any criticisms or requested revisions that you disagreed with. Please also ensure that your revised manuscript conforms to the journal style, which can be found in the Instructions for Authors on the journal homepage. If the data and code has been modified in the revision process please be sure to update the public versions of this too.<br><br>The due date for submitting the revised version of your article is 05 Nov 2025.<br><br>I look forward to receiving your revised manuscript soon.<br><br><br>Thank you for the feedback! We have carefully addressed all reviewer comments and made substantial improvements to enhance clarity, reproducibility, and usability.<br>Key updates include:<br>The human prefrontal cortex (PFC) integration example, which demonstrates the utility of scDenorm across studies with different pipelines, has been promoted to main Figure 7, as suggested.

Powered by Editorial Manager® and ProduXion Manager® from Aries Systems Corporation

A ready-to-use Docker image and detailed environment files (environment.yaml for Python and installed_packages.csv for R) to ensure reproducibility.
All required input data, metadata, and pre-computed results have been made available via Zenodo (https://zenodo.org/uploads/17275776).
A new "How to install and use scDenorm" section added in the GitHub README, with installation instructions, a quick-start example, and a link to full documentation.
scDenorm has been registered in bio.tools (biotoolsID: scdenorm) and SciCrunch.org (RRID: SCR_027574), and the corresponding identifiers have been included in the manuscript.
We believe these revisions significantly improve the manuscript and its reproducibility.
Please find all these improvements below in our point-to-point answers to the reviewers' comments.

Reviewer 2

1.0 Gigascience review for Huang et al, scDenorm: a denormalisation tool for integrating single-cell transcriptomics data
The authors aim to address the important and often irritating problem of how to combine scRNAseq data from different sources and provided after processing with different normalization tools. The authors note that often times raw counts matrices are not provided, nor information on how the data was normalized. Their tools aim to estimate the parameters used for data transformation (eg. scale factor, pseudocount addition, log-transformation), and reverse those transformations to return to the raw counts matrix again.
In an ideal world where public data is well-documented and actually fully available, this type of reverse-engineering would not be necessary, but due to the current state of many data repositories, in many cases, the tools the authors provide would be indeed very useful. The paper does a good job at identifying the problem, implementing a systematic solution with useful benchmarks, and showing multiple examples of the method's utility using public datasets. However, a few additional questions arise about their method:
Thank you for your recognition of the tool we have developed. Following are the answers for reviewers' comments and suggestions one by one.
1.1 Figure 1c-d: UMAP is a distortion/projection onto a 2D space, so it is not super surprising that different scaling factors produce clouds of cells in different locations. Do the differences in scaling factors affect the identification of the cells in any way? I.e. After denormalization for datasets of different scale factors/logbases/pseudocounts, when performing renormalization on the newly obtained raw counts, is there an optimal set of normalization parameters to now use?

Thank you for the comment. As illustrated in Figures 5–7, differences in normalization methods among datasets can lead to artificial inflation or suppression of marker gene expression. This, in turn, affects the accuracy of cell type identification. For example, in Figure 5b, the confusion matrix heatmap shows that a substantial proportion of $CD14^{+}$ monocytes were misclassified as HSPCs or pDCs, which we attribute to inconsistencies in normalization across datasets.

To avoid such bias, we recommend starting from raw counts and applying a consistent normalization method across datasets. This minimizes technical biases and ensures that biological signals are preserved, thereby improving the reliability of downstream analyses such as cell type annotation and differential expression.

1.2 Figure 2: For a particular dataset, if it is not known how the data was normalized and the transformation did not follow the form of log(C/T + P), will the tool alert the user to this? Or will the tool still attempt to find the best fit log base and pseudocount? In other words, prior to performing the denormalization, it might be helpful to display some metrics that indicate that the delta method was the most likely normalization method

used, especially when ones tries to apply the tool to hundreds of datasets. (However, it seems it may not be possible to tell whether deNorm is successful until it is attempted (both empirically trying common log-base/pseudocount combinations, or solving the optimization equation), and then one assesses whether the resulting data is negative binomially distributed.)

Thank you for the suggestion. scDenorm is designed to automatically infer the most likely normalization parameters—specifically the log base and pseudocount—under the assumption that the transformation follows the form log(C/T + P). If the data conforms to this model and the resulting denormalized values follow a negative binomial distribution, the tool will report the best-fit parameters used.

However, if the data does not match the expected transformation form or fails to follow a negative binomial distribution, it will notify the user that denormalization was unsuccessful and return the original input data. This ensures transparency and prevents misleading downstream analysis.

1.3 Figure 3d does not seem sufficiently described in the text. The authors write: "For recovery error, the absolute values are below 10-6 in 27 datasets normalized with natural logarithmic transformation (Fig. 3d, Supplementary Table 1), indicating a good accuracy of scDenorm (Fig. 3f)." Fig 3f does show recovery error, and Fig 3e shows rounding error, but what does Fig 3d intend to show? Text clarification would be helpful.

Thank you for pointing this out. Figure 3d presents the distribution of success rates across the 27 datasets that were normalized using the natural logarithmic transformation.

As defined in Equation (11) method section (see P13L495), the success rate is calculated as the proportion of cells that were successfully denormalized:
success rate = Nsuccess/Ntotal (11)

Nsuccess is the number of cells for which scDenorm was able to recover valid raw counts, and Ntotal is the total number of cells in the dataset. In some cases, denormalization may fail for a subset of cells due to poor sequencing quality or low gene counts. Figure 3d visualizes this variability across datasets.

We now revise the main text to explain the purpose of Figure 3d as below:

P5L184
To further assess the robustness of scDenorm across diverse datasets, we evaluated its performance on 27 datasets that were normalised using a natural logarithmic transformation. We present the distribution of success rates (Fig. 3d), defined as the proportion of cells that were successfully denormalised (Equation 11). This metric accounts for cases where poor sequencing quality or a low number of expressed genes may result in cell-wise deviations from the expected negative binomial distribution, thereby preventing accurate recovery during denormalisation. The results demonstrate that scDenorm performs robustly across diverse datasets, even when some cells cannot be fully recovered.

P13L495
In certain cases, not all cells can be successfully denormalised due to poor sequencing quality, or a low number of expressed genes. To evaluate denormalisation in such situations, we define success rate as the percentage of successfully denormalised cells, equation (11).

success rate = Nsuccess/Ntotal (11)

Powered by Editorial Manager® and ProduXion Manager® from Aries Systems Corporation

Nsuccess is the number of successfully denormalised cells, while Ntotal is the total number of cells.

1.4 For Fig 3d, why do certain datasets have lower "success rate"? It would be helpful for the bars in Fig 3d to be in the same order as the blue bars in Fig 3b, to better compare any relationship between gene or cell number with "success rate".

Thanks for this insightful comment. Certain datasets exhibit lower success rates in Figure 3d due to technical issues such as poor sequencing quality or a low number of expressed genes (filtered datasets), which may cause individual cells to deviate from the expected negative binomial distribution. Since scDenorm relies on this distributional assumption to accurately recover raw counts, such deviations can hinder successful denormalisation.

We have a supplementary figure to match the order between Figure 4d and Figure 3b to better compare any relationship between gene or cell number with "success rate". Please see the Supplementary Figure 7 as below.

Supplementary Fig. 7 Benchmark analysis on the datasets from
Brain Cell Atlas
a, The bar plot shows the number of genes in each dataset. The x-axis is the name of datasets (same as d), and the y-axis is the log-scaled number of genes.
b, The bar plot shows the number of cells in each dataset. The x-axis is the name of datasets (same as d), and the y-axis is the log-scaled number of cells.
c, The bar plot shows the success rate for each dataset. The x-axis is the name of datasets (same as d), and the y-axis is the success rate (see Method).
d, The jitter plot shows the distribution of rounding errors observed in the denormalised datasets from the Brain Cell Atlas. The x-axis is the name of datasets, and the y-axis is the rounding error.
1.5 In addition, from Methods it appears that the determination of "Success Rate" relies on knowing the original raw count values. For those data sets where many of the cells could not be successfully denormalized to raw values, what is the impact of that on the downstream cell identification or DEG analysis?
Sorry for the confusion. The calculation of success rate does not require access to the original raw count values. Instead, we evaluate whether a cell has been successfully denormalised based on the rounding error, defined as the difference between the denormalised value and its nearest integer. If the denormalised values are very close to integers (default: the rounding error<0.01), this indicates a successful recovery of count-like data.

Cells with large rounding errors are considered unsuccessfully denormalised and are filtered out prior to downstream analysis. As these cells typically correspond to low-quality profiles, their exclusion does not affect downstream tasks such as cell type identification or differential expression analysis. This filtering step ensures that only reliably recovered data are used for biological interpretation.
1.6 Figure 4f: Not sure if this is an error in image display, but I do not see any blue points (original data) in the UMAP, as described in the legend.
Thanks. The absence of visible blue points (original data) in Figure 4f is due to the fact that they are largely overlapped by the orange points, as both sets of coordinates are nearly identical.

To clarify this, we have included a figure showing the UMAP plots for the three conditions separately as below:

Revision Fig 1. Separate UMAP plots of different conditions (Origin, Floating, and HVG)

1.7 Figures 5-6: The authors mention assessing how scDenorm helps in different scenarios where one might want to combine data: "the batch in the COVID-19 PBMCs

dataset are samples from two patients, while in the human prefrontal cortex (PFC)31,35 dataset are samples from two different studies. And the batch in the human skin36 dataset are groups of samples from young and old donors."
The most interesting scenario here are the samples from two different studies, perhaps performed by different labs, with different bioinformatic pipelines, mapped to different versions of the genome, etc. Thus, I thought the example of integrating human PFC from two different studies and how the cell type identification changed (via the river plots) was the most compelling example, and the authors could consider making that a main figure instead.
Thanks for the thoughtful feedback. We have promoted the human prefrontal cortex example to a main figure (Figure 7) in the revised manuscript. The river plots clearly demonstrate how scDenorm improves cell type identification by reducing misclassification and enhancing consistency across batches. We believe this example effectively illustrates the utility of scDenorm in real-world multi-study integration tasks.
1.8 The linked github repository, while commendable in providing code to reproduce figures, could benefit from further documentation/README showing the user how to use the tool.
Thanks. We have updated the README file to include a dedicated section titled “How to install and use scDenorm” as below. This section provides clear instructions on installation, a quick-start example for applying scDenorm to a dataset, and a link to the full documentation for further details.

How to use and install scDenorm
- Install: pip install scDenorm
- Usage: scdenorm data/pbmc3k_norm.h5ad --fout data/pbmc3k_denorm.h5ad
- Documentation: https://changebio.github.io/scDenorm

Reviewer 3

Reproducibility report for: scDenorm: a denormalisation tool for integrating single-cell transcriptomics data
Journal: GigaScience
ID number/DOI: GIGA-D-25-00209
Reviewer(s): Laura Caquelin, Department of Clinical Neuroscience, Karolinska Institutet, Sweden
----
1. Summary of the study
In order to reduce biaises caused by different normalization methods, the authors developed scDenorm, "an algorithm that reverts normalised single-cell omics data to raw counts". They evaluated its performance on large-scale datasets to assess its impact on data integration and downstream analysis.
----
2. Scope of reproducibility
According to our assessment the primary objective is: to evaluate whether scDenorm improves the biological relevance of gene expression analyses by comparing GO term enrichment from differentially expressed genes (DEGs), before and after denormalization against a gold standard.
Note: While this is not the main outcome highlighted in the article's conclusion, we chose to focus on the GO enrichment analysis because its involves statistical inference abd provides a clear and reproducible downstream result.
- Outcome: GO enrichment profiles based on hematopoietic stem and progenitor cells for each condition (gold standard, before scDenorm and after scDenorm).
- Analysis method outcome: "Downstream analysis after cell type annotation includes differential expression (DE) analysis and Gene Ontology (GO) pathway analysis. Differential gene expression analysis was conducted for each cell type (one against the rest) using the Wilcoxon test implemented in SCANPY19. As part of the dataset is used as the reference dataset, the differentially expressed genes (DEGs) derived from the reference dataset is used as 'gold standard' in our evaluation. The same approach was used for the calculation of the DEGs before and after scDenorm. The top differentially expressed genes were compared across different thresholds (top 50, 100, 200, 500, and 1000). As for GO pathway analysis, the enrichGO program was used on the top 500 differential expression genes.

	DE and GO analysis with the correct labels from Solé-Boldo et al. was conducted for each cell type (one against the rest) using the Wilcoxon test implemented in Seurat (V3.1.1), same as the version described in Solé-Boldo et al. These analyses were conducted before scDenorm and after scDenorm. The differentially expressed genes were obtained from the study's supplementary materials as 'gold standard'". (Method part, Dataset processing for data integration and downstream analysis section, page 14-<br>15)<br>- Main result: "Gene enrichment analysis of the differential genes in HSPCs indicated that the GO terms enriched after scDenorm closely aligned with those of the 'gold standard', whereas the enrichment before scDenorm showed minimal overlap (Supplementary Fig. 10f). The enriched GO terms are relevant functions associated with HSPC cells, such as hematopoietic stem cell proliferation and hematopoietic progenitor cell differentiation (Fig. 5f)." (Results part, page 7)<br>---<br>3. AvailabilityofMaterials a. Data<br>- Data availability: Open<br>- Data completeness: Some data necessary to reproduce the main results were initially missing but were provided by the authors after we requested them.<br>- Access Method: Repository<br>- Repository: https://doi.org/10.5281/zenodo.11240443<br>-Data quality: The data files have been shared and appear sufficient for running the analyses. However, no metadata is provided to describe the content, structure, or origin of the files which limits interpretability and reusability.<br>b. Code<br>- Code availability: Open<br>- Programming Language(s): Python (Jupyter Notebook)<br>- Repository link: https://github.com/rnacentre/scDenorm-<br>reproducibility<br>- License: Apache 2.0<br>- Repository status: Public<br>- Documentation: The documentation could be improved. Important<br>details about the computational environment are missing, which makes it difficult to set up and reproduce the analysis. Additionally, since the code scripts are long and complex, navigating and understanding the workflow is challenging without clearer explanations.<br><br>---<br>4. Computational environment of reproduction analysis<br>- Operating system for reproduction: MacOS 15.5<br>- Programming Language(s): Python, R<br>- Code implementation approach: Using shared code with the jupyter notebook<br>- Version environment for reproduction: Creation of an environment with Python 3.10 and R 4.5.0<br><br>---<br><br>5. Results<br><br>5.1 Original study results - Results 1: Figure 5f<br>5.2 Steps for reproduction<br>-> Installation and environment setup<br>- Issue 1: Incompatibility between SCCAF and Python.<br>-- Resolved: SCCAF, when installed via PyPI, required scanpy==1.4.6, which is incompatible with Python 3.12, causing a TypeError due to metaclass conflicts. A new conda environment was created with Python 3.10. The authors specified that the PyPI version of SCCAF is outdated and recommended using their GitHub repository (https://github.com/rnacentre/sccaf).<br><br>- Issue 2: Incompatibility with recent versions of pandas.<br>-- Resolved: An ImportError occurred due to using pandas==2.2.3.<br>Downgrading to pandas==1.5.3 solved the issue.<br><br>- Issue 3: Incompatibility with recent versions of anndata.

Powered by Editorial Manager® and ProduXion Manager® from Aries Systems Corporation

	-- Resolved: Using anndata==0.11.4 raised import errors. Downgraded to anndata==0.8.0, which resolved the issue.<br>Setting up a clean environment with Python 3.10, scanpy==1.9.1, matplotlib==3.5.3, pandas==1.5.3, anndata==0.8.0, and the GitHub SCCAF package resolved the issue.<br>Authors validated the environment configuration above.<br><br>-> Running the original notebook (fig5.ipynb)<br>- Issue 4: KeyError due to missing labels ('Neutrophil', 'RBC') in confusion matrix.<br>-- Half resolved: Modified indexing logic to handle missing labels. The authors explained this variability stems from model randomness trained by SCCAF. Authors suggested filtering the label list accordingly:<br>conf_mat = conf_mat.loc[idx, [i for i in idx if i in conf_mat.columns]]<br>The proposed fix was to filter the columns with [i for i in idx if i in conf_mat.columns], but this still raises the same error due to missing labels in the rows.<br>conf_mat = conf_mat.loc[[i for i in idx if i in conf_mat.index] , [i for i in idx if i in conf_mat.columns]]<br>This error recurs throughout the code, requiring numerous modifications to be made.<br><br>- Issue 5: KeyError when building Sankey diagram ""Bad flow - couldn't find destination node: ('CD4+ T cell ', 'CD8+ T cell', 148)"<br>-- Resolved: Authors proposed instead of hardcoding the label list :<br>for i in new_ad.obs.predict0.value_counts()[['B cell', 'CD4+ T cell', 'NK cell', 'Other T', 'CD16+ Monocyte', 'cDC', 'pDC', 'HSPC', 'Plasmablast', 'Platelet']].items():<br>Simplify it to:<br>for i in new_ad.obs.predict0.value_counts().items():<br><br>- Issue 6: KeyError: 'predict0'<br>-- Resolved: Column predict0 did not exist. Replaced with predict and predict_colors:<br>tmp = new_ad.obs.groupby(['cell', 'predict']).size()<br>right_cols = pd.Series(new_ad.uns['predict_colors'], index=new_ad.obs.predict.cat.categories)<br>Multiple predict0 en predict modifications had to be made throughout the code.<br><br>- Issue 7: ValueError no field of name Neutrophil<br>-- Unresolved: New error in this part of code:<br>df_bench=[]<br>for i in idx:<br>tmp=[]<br>for j in [50,100,200,500,1000]:<br>tmp.append(len(set(adx0.uns['ground']['names'][i][:j]).intersection(adx 0.uns['wilcoxon']['names'][i][:j])))<br>df_bench.append(tmp)<br><br>After asking authors about this error, they precised that if the goal is to reproduce the results of scDenorm, the Sankey plot part can be skip and proceed directly to the section "After scDenorm".<br><br>-> Running the original notebook (fig5.ipynb) - "After scDenorm" section<br>- Issue 8: NameError: name 'ad1' is not defined<br>-- Resolved: Replaced ad1 with ad3, which had been defined earlier. Authors confirmed this change is correct.<br><br>- Issue 9: AttributeError: 'DataFrame' object has no attribute 'feature_name'<br>-- Unresolved: The line of code "ad.var_names=list(ad.var.feature_name) not working. feature_name column not present in ad.var. This part was skipped.<br><br>- Issue 10: KeyError: 'wilcoxon' in plotting DEG results<br>-- Resolved: Clarified that 'wilcoxon' is the method, not the key. Correct usage:<br>sc.pl.rank_genes_groups(adx0, n_genes=25, sharey=False, key="ground")<br>- Issue 11: Missing data files<br>-- Resolved: The following files are required but not found in the repository: * PBMC_before_scDenorm_DEGs.pkl<br>* PBMC_after_scDenorm_DEGs.pkl

Powered by Editorial Manager® and ProduXion Manager® from Aries Systems Corporation

	* PBMC_go_analysis_result.csv<br>After request, the authors provided the script R_goanalysis.ipynb, which reproduces the PBMC_go_analysis_result.csv file, along with the necessary data files.<br>-> Running the R_goanalysis.ipynb and the "DEG and GO venn" section<br><br>5.3 Statistical comparison Original vs Reproduced results - Results:<br><br>- Comments: As the results we obtained were completely different from those reported in the manuscript, and identical before and after scDenorm, I contacted the authors to request details about the computational R environment to run the code R_goanalysis.ipynb. Although we used the environment they provided, this did not resolve the discrepancy.<br>- Errors detected: -<br>- Statistical Consistency: We obtained results that were completely different from those reported in the original study. Moreover, the outputs for the "after scDenorm" and "before scDenorm" conditions appeared graphically identical.<br>--<br><br>6. Conclusion<br>- Summary of the computational reproducibility review<br>The reproduction of the results in the scope of this computational reproducibility review was not successful. Many errors were encountered when running the original fig5.ipynb notebook, including missing or outdated variables, missing files, and misalignments in hard-coded labels. While most of these issues were resolved through direct communication with the authors and code modifications, some steps remained partially or completely irreproducible.<br>The results we obtained for the figure 5f were entirely different from those reported in the original manuscript. Despite using the R script (R_goanalysis.ipynb) and data files shared by the authors, and running them in the specified environment, the GO analysis results were inconsistent with those presented in the publication. Furthermore, the "after scDenorm" and "before scDenorm" conditions produced graphically identical results. This raises questions about whether the differences come from the code, the data, or something else in the analysis.<br><br>- Recommendations for authors<br>To make the study easier to reproduce and more transparent, we suggest the following:<br><br>-- Add a requirement file that lists all the needed packages with their exact versions and mention which Python and R versions are supported. Make it clear in the documentation that the SCCAF version on PyPI is outdated and point users to the up-to-date version on GitHub.<br>-- Make sure all data files needed to reproduce the figures are available in the repository.<br>-- Check that the analysis code does not depend on fixed labels or assumptions that might not work with other datasets or slightly different results.<br>-- Clearly explain which parts of the results may vary due to randomness in the model and how much variation users should expect.<br>-- Consider adding some pre-computed results or intermediate files so users can compare their outputs with the original study more easily.<br><br><br>We sincerely thank the reviewer for the detailed and thoughtful reproducibility assessment. We greatly appreciate the effort to test scDenorm and have carefully addressed all points raised to improve the reproducibility, usability, and transparency of our study.<br>Reproducibility of Figure 5f and GO Analysis<br>Sorry for the confusion. We have reorganized and updated the code in Fig5.ipynb and R_goanalysis.ipynb (now renamed to Fig5_R_goanalysis.ipynb) to ensure clarity and consistency.<br>A reproducible docker environment closely matching our local setup has been constructed. We re-ran the analysis and obtained results consistent with those reported in the manuscript.<br>To facilitate validation, we have provided pre-computed GO enrichment results and

Powered by Editorial Manager® and ProduXion Manager® from Aries Systems Corporation

	intermediate files at Zenodo (https://zenodo.org/uploads/17275776).<br>Docker Image for Reproducibility<br>To further simplify setup and ensure reproducibility, we have provided a ready-to-use Docker image that includes all dependencies and environment configurations. This allows users to reproduce results without manual installation.<br>Improved Documentation and Environment Setup<br>We have added a comprehensive environment.yaml file for the Python environment and an installed_packages.csv file for the R environment, specifying exact versions of Python, R, and all required packages.<br>The GitHub README now includes a tutorial for running the notebooks, with step-by-step instructions as below:<br>Clone the repository:<br>git clone https://github.com/rnacentre/scDenorm_reproducibility.git<br>Download the Docker image and data from Zenodo: https://zenodo.org/uploads/17275776.<br>Move all downloaded data to scDenorm_reproducibility/data folder.<br>Run the Docker image (How to install docker: https://docs.docker.com/engine/install/):<br>tar -xzf scdenorm_v0.tar.gz<br>docker load -i scdenorm_v0.tar<br>docker run -p 8888:8888 -v /path/to/scDenorm_reproducibility:/app scdenorm_v0 jupyter lab --ip=0.0.0.0 --no-browser --allow-root<br><br>Clarification on Model Randomness<br>We have added annotation in the notebooks which parts of the workflow may vary due to model randomness (e.g., SCCAF training) and provided pre-computed results for reproducibility.<br>Availability of Data Files and Pre-computed Results for Comparison<br>All previously missing data files required to reproduce the figures have been added to Zenodo. We have also included pre-computed outputs and intermediate files to support reproducibility and allow users to compare their results with those reported in the manuscript.<br><br>We believe these revisions fully address the reviewer's concerns and significantly improve the reproducibility and transparency of scDenorm. Thank you again for your valuable feedback and suggestion.
**Additional Information:**	
**Question**	**Response**
Are you submitting this manuscript to a special series or article collection?	No
**Experimental design and statistics**<br><br>Full details of the experimental design and statistical methods used should be given in the Methods section, as detailed in our [Minimum Standards Reporting Checklist](). Information essential to interpreting the data presented should be made available in the figure legends.<br><br>Have you included all the information requested in your manuscript?	Yes
**Resources**	Yes

A description of all resources used, including antibodies, cell lines, animals and software tools, with enough information to allow them to be uniquely identified, should be included in the Methods section. Authors are strongly encouraged to cite Research Resource Identifiers (RRIDs) for antibodies, model organisms and tools, where possible.<br><br>Have you included the information requested as detailed in our Minimum Standards Reporting Checklist?	
**Availability of data and materials**<br><br>All datasets and code on which the conclusions of the paper rely must be either included in your submission or deposited in publicly available repositories (where available and ethically appropriate), referencing such data using a unique identifier in the references and in the “Availability of Data and Materials” section of your manuscript.<br><br>Have you have met the above requirement as detailed in our Minimum Standards Reporting Checklist?	Yes
GigaScience has policies and guidelines in place for the use of generative AI-writing tools such as ChatGPT. If you have used such writing tools to assist with writing the manuscript this must be declared and cited in the text. Authors should not list AI-writing tools and other AI-assisted technologies as an author or co-author and should acknowledge that they are fully responsible for text generated or refined by AI-writing tools.<p><br><br>A summary of use (particularly in the introduction or among methods) needs to be included at the end of the paper, and the outputs should also be included as a supplementary file hosted in GigaDB or other open repositories. Please <a href=https://academic.oup.com/gigascienc	No

Powered by Editorial Manager® and ProduXion Manager® from Aries Systems Corporation

e/pages/editorial_policies_and_reporting_standards target="_new" > read our guidelines for more information. <p><br><br>By submitting to GigaScience, you are aware of the journal's AI-writing tools policy, and if you have declared use of such tools below, you have acknowledged this where appropriate in your manuscript and have made a summary of use and outputs available. <p><br><b>AI-assisted writing tools have been used in the preparation of this manuscript?	

Powered by Editorial Manager® and ProduXion Manager® from Aries Systems Corporation

Manuscript Click here to access/download;Manuscript;scDenorm_R2.pdf

scDenorm: a denormalisation tool for integrating single-cell transcriptomics data

Yin Huang[1,2], Anna Vathrakokili Pournara[4], Ying Ao[3], Ziliang Huang[2], Hui Zhang[5], Yongjian Zhang[6], Sheng Liu[7,8], Alvis Brazma[4], Irene Papatheodorou[4], Xinlu Yang[5,*], Ming Shi[9,*], Zhichao Miao[2,3,*]

[1] Translational Research Institute of Brain and Brain-Like Intelligence and Department of Anesthesiology, Shanghai Fourth People's Hospital Affiliated to Tongji University School of Medicine, Shanghai, China
[2]Guangzhou National Laboratory, Guangzhou International Bio Island, Guangzhou 510005, China
[3] GMU-GIBH Joint School of Life Sciences, Guangzhou Medical University, Guangzhou, China
[4] European Molecular Biology Laboratory, European Bioinformatics Institute, EMBL-EBI, Wellcome Genome Campus, Cambridge CB10 1SD, UK
[5] Department of Obstetrics and Gynaecology, Harbin Red Cross Central Hospital, Harbin 150001, China.
[6] Department of Surgery Oncology, Harbin Medical University Cancer Hospital, Harbin 150001, China
[7] State Key Laboratory of Ophthalmology, Zhongshan Ophthalmic Center, Sun Yat-sen University, Guangdong Provincial Key Laboratory of Ophthalmology and Visual Science, Guangzhou, China
[8] Guangdong Province Key Laboratory of Brain Function and Disease, Guangzhou, China
[9] School of Life Science and Technology, Harbin Institute of Technology, Harbin 150001, China
* correspondence should be addressed to Zhichao Miao, miao_zhichao@gzlab.ac.cn, Ming Shi, shiming@hit.edu.cn, and Xinlu Yang, 519950370@qq.com

Abstract

Integrating single-cell omics data at an atlas scale enhances our understanding of cell types and disease mechanisms. However, the integration of data processed by different normalisation methods can lead to biases, such as unexpected batch effects and gene expression distortion, leading to misinterpretations in downstream analysis. To address these challenges, we present scDenorm, an algorithm that reverts normalised single-cell omics data to raw counts, preserving the integrity of the original measurements and ensuring consistent data processing during integration. We evaluated scDenorm's performance on large-scale datasets and benchmarked its impact on data integration and downstream analysis across three datasets.

Background

Single-cell RNA sequencing (scRNA-seq) is a powerful high-throughput technology for measuring gene expression in individual cells. Integration of atlas-level single-cell transcriptomics data has

exerted a great potential for understanding how cells orchestrate in the human body, as well as complex molecular mechanisms in various diseases[1,2]. With the progress of the Human Cell Atlas (HCA)[3], an increasing number of reference atlases are available for comparison and integration[4–6]. Numerous integration methods have been developed and several studies have been performed to benchmark their performance and explore their limitations[7–9]. In order to achieve effective large-scale data integration, it is crucial to take into account the assumptions of data distribution and noise levels. For instance, scVI[10] and scANVI[11] integration methods model single-cell data using a Negative Binomial distribution (also known as Gamma-Poisson distribution), and thus both require raw counts as input. Even though some other integration methods, *e.g.,* Seurat integration methods[12] (RPCA, CCA), Harmony[13], Liger[14] etc, do not directly rely on raw counts, they inherently make assumptions about the data distribution. As a result, for most existing integration methods, it is key to ensure the consistency of the input datasets.

To address technical variations (*e.g.,* sequencing depth) and biases inherent in scRNA-seq, scaling and transformation methods are often employed to ensure comparability across cells[15,16]. Normally, scaling is used to account for sequencing depth, while transformation is used to stabilise variance of the data. The differences between variance-stabilising transformations have been benchmarked by Constantin Ahlmann-Eltze and Wolfgang Huber[17], demonstrating the effectiveness of the delta method for comparing cells with varying gene expression levels. In a delta normalisation, raw counts are scaled by total counts and target sum, followed by log-transformation with an added pseudo count (see **Methods**). It has been adopted in well-established analysis workflows (e.g., Seurat[18] and SCANPY[19]), assuming that droplet-based scRNA-seq data follows a negative binomial distribution[20–23]. In some large-scale data resources, such as the UCSC Cell Browser[24], delta method normalised matrices are deposited instead of the raw counts to facilitate reproducibility of analysis results. Thus, many datasets are available only as processed matrices rather than raw counts, hindering atlas-level data integration.

The best way to guarantee consistent data processing in large-scale data integration is to use raw counts as input. Integrating processed data with raw counts can introduce unnecessary biases, due to re-normalising of data that has already been normalised. Some downstream analysis steps[25,26] (such as selecting highly variable genes, differential gene expression analysis) also assume raw counts as input. When raw counts are not available, researchers often seek to obtain the raw sequencing data and re-analyse them, including secondary analysis of reads mapping, demultiplexing, and quantification analysis[27] to obtain the raw count matrix. However, a count matrix from the re-analysis may deviate from the original published analysis in terms of reference genome and cell barcodes. Thus, the cell type annotation or other metadata reported in the raw publication cannot be used, rendering difficulties in reproducing the analysis results. Besides, this secondary analysis can be both computationally expensive and time-consuming. Therefore, reliable conversion of normalised matrices back to raw counts can benefit large-scale data integration tasks as well as wider use of publicly deposited data. Yet, there is no tool available to meet this urgent need.

In this study, we propose scDenorm, an algorithm that converts delta method normalised gene expression data back to the raw counts. It effectively explores key implicit features of the data

distribution in scRNA-seq and recovers raw count matrices. Based on benchmarking across large-scale datasets, as well as application studies of downstream analysis, we demonstrate the capability, accuracy, scalability and efficiency of this method. Moreover, scDenorm can deal with different normalisation parameters, thereby facilitating data integration, consistent downstream analyses and the construction of atlases.

Results

Inconsistent data normalisation may generate biases in data integration

Using the 10x 3k peripheral blood mononuclear cells (PBMCs) data, which is the example data used in the well-established SCANPY[19] and Seurat[18] single-cell tutorials, as an example, we investigated the impact of normalisation parameters in the delta method. These parameters are the scaling factor, logarithmic transformation base, and pseudo counts. Matrices normalised by different parameters go through the same downstream analysis of highly variable gene selection, dimensionality reduction (e.g., Principal Component Analysis), clustering and visualisation. The Uniform Manifold Approximation and Projection (UMAP) plot shows deviations between datasets processed with different normalisation parameters, e.g., the deviations between B cells in $L=10^3$ and the same B cells in other normalisations, indicating the potential bias introduced by inconsistent data normalisation (**Fig. 1a,b**, **Supplementary Fig. 1a**). Furthermore, such a data normalisation effect cannot be removed through data integration by Harmony[13], scanorama[28], or BBKNN[29] (**Fig. 1**, **Supplementary Fig. 1**), e.g., the B cell populations in **Fig. 1c** and **1d** cluster separately. Therefore, we suggest converting the normalised matrices back to raw counts for a consistent data integration and downstream analysis.

The denormalisation process in scDenorm

We term the recovery of normalised data to raw counts as "denormalisation". Denormalising delta method normalised data requires the determination of three parameters, scaling factors, the logarithmic transformation (log-transformation) base, and the pseudo count. The first step for denormalisation is to determine if log-transformed has been applied for the whole expression matrix. It is well established that droplet-based scRNA-seq data follows a negative binomial distribution[20–23], where the variance exceeds the mean (**Fig. 1e**). Thus, the variance versus mean distribution effectively indicates whether the data has been log-transformed or not (**Supplementary Fig. 2a**). The second key step in denormalisation is to determine the scaling factor for each cell. This needs to exploit the implicit data distribution feature of scRNA-seq. Droplet-based scRNA-seq mainly probes the highly expressed genes, rendering a high dropout rate. In a sparse matrix where zeros have been removed, the frequency of counts can be ranked, with the most frequent count number being one, followed by two, and so on (**Fig. 1f**, **Supplementary Fig. 2b,c**). Using such a 'count-rank' distribution, scaling factors for cells can be measured by establishing the relationship between the top two most frequent numbers in the normalised data and numbers one and two. After exploring 105 datasets from the Brain Cell

Atlas[30], we found that over 99% of the cells follow this 'count-rank' distribution for the top three most frequent count numbers (one, two, and three), while >95% cells in Chromium and > 80% cells in Drop-seq follow the distribution for the top five count numbers. Notably, >99% cells in Smart-seq2 data follow this distribution for the top ten numbers (**Fig. 1g**, **Supplementary Fig. 2d**). Following this 'count-rank' distribution, the top most frequent count numbers can be used to determine the three parameters in delta method normalisation (**Supplementary Fig. 3a**).

The denormalisation procedure in scDenorm involves two steps: de-transformation and unscaling (**Supplementary Fig. 3b**). In the de-transformation step, a subset matrix (100 cells) is used to determine the same log-transformation base and pseudo count among cells since these two parameters keep the same for the whole expression matrix. Using a subset of data effectively accelerates the calculation. First, empirical values (*e.g.,* 2, e Euler's number, 10 for the log-transformation base, 0.01, 0.1, 1 for pseudo count), which are used in standard analysis workflows, are tried. If not successful, these two parameters can be determined by solving equations between the top two most frequent numbers (**Supplementary Fig. 3c**). In the unscaling step, each cell has a different scaling factor, which is a ratio between the total counts of the cell and the target sum (e.g., 10,000). To measure the scaling factor of a cell, we implement two methods: 1) a regression-based method (see **Methods**, equation (4) in **Supplementary Fig. 3d**) and 2) solving equations between the top two most frequent numbers (see **Methods**, equation (5) in **Supplementary Fig. 3d**), while the latter method offers the advantages of fast speed and good robustness (**Supplementary Fig. 4a,b**). As the expression matrix is processed from raw counts, which consists of integers only, a successful denormalisation should result in a small mean square error between denormalised values and their nearest integers (see **Methods**).

To elaborate the denormalisation process, we used an example dataset[31] of single-nucleus RNA sequencing (snRNA-seq) data of autism spectrum disorder, with both the normalised data and raw count matrix available in the database from https://autism.cells.ucsc.edu. According to the respective publication[31], the data was normalised with the delta method. The relationship between the top ten most frequent gene expression values and their respective frequencies in three cells in the processed data (**Fig. 2a**), suggests a logarithmic distribution, while the less frequent values after them do not follow such a distribution due to dropouts. Should any of the cells in the dataset follow the same distribution. And the mean versus variance distribution confirms a logarithmic conversion (**Fig. 2b**). The log-transformation base and the pseudo count are determined as 2 and 1 respectively, by solving equation (3) in **Supplementary Fig. 3c**. These parameters show a good fit according to the top two most frequent values (**Fig. 2c**). The normalised matrix is de-transformed by taking the exponential of the log-transformation base and subtracting the pseudo count, resulting in a 'scaled matrix'. In the scaled matrix, the top five most frequent values show a linear 'count-rank' distribution in each cell (**Fig. 2d**). The slope of the line is the reciprocal of the scaling factor. This linear distribution indicates the success of de-transformation. Additionally, the mean versus variance distribution (**Fig. 2e**) confirms this success. The summed expression values for most cells are approximately 10,000, indicating that the target sum is 10,000. Some genes may have been removed after normalisation, leading to a reduction in the summed expression values (**Fig. 2f**). In the unscaling step, scaling factors are determined by solving equation (5) in **Supplementary Fig. 3d.** Each cell is multiplied by its scaling factor resulting in a

"denormalised matrix", which is supposed to be similar to the raw count matrix of integers. As in a sparse matrix, the top two most frequent numbers should be one and two (**Fig. 2g**). The mean versus variance distribution of the denormalised matrix conforms to a negative binomial distribution (**Fig. 2h**), which is expected for the raw counts of droplet-based scRNA-seq. Comparing the denormalised matrix with the raw count matrix, the maximum error for each value was less than 0.001 (**Fig. 2i**), which may result from the digital float calculation. After taking round values, the denormalised matrix is identical to the raw count matrix, suggesting a successful denormalisation.

scDenorm recovers raw count matrices for large-scale database

To evaluate the performance of scDenorm in real scenarios, 40 processed datasets (**Supplementary Table 1**) from the UCSC Cell Browser[24] were used as test data, covering a good variety of species, tissues and sequencing techniques (**Fig. 3b**). Denormalisation performance was evaluated by two metrics: 1) rounding error, defined as the difference between a value in the denormalised matrix and its nearest integer (round value), and 2) recovery error, defined as the difference between a value in the normalised matrix and its corresponding value in the denormalised matrix after renormalisation (**Fig. 3a**, See **Methods**). 32 out of the 40 test sets were successfully denormalised (**Fig. 3c**, **Supplementary Table 1**), while the 8 unsuccessful cases were normalised as TPM (Transcripts per Million), log2(FRPM) or by scTransform[32] method rather than the delta method (**Supplementary Table 1**). The mean versus variance distribution confirms a negative binomial distribution after denormalisation, indicating the successful denormalisation (**Supplementary Fig. 5**). To further assess the robustness of scDenorm across diverse datasets, we evaluated its performance on 27 datasets that were normalised using a natural logarithmic transformation. We present the distribution of success rates (**Fig. 3d**), defined as the proportion of cells that were successfully denormalised (**Equation 11**). This metric accounts for cases where poor sequencing quality or a low number of expressed genes may result in cell-wise deviations from the expected negative binomial distribution, thereby preventing accurate recovery during denormalisation. The results demonstrate that scDenorm performs robustly across diverse datasets, even when some cells cannot be fully recovered. The rounding errors, which are positively correlated to the expression value (**Supplementary Fig. 6**), are consistently below 0.005 (**Fig. 3e**). For recovery error, the absolute values are below 10^{-6} in 27 datasets normalised with natural logarithmic transformation (**Supplementary Table 1**), indicating a good accuracy of scDenorm (**Fig. 3f**). Further benchmarking of the denormalisation on 60 datasets (**Supplementary Table 2**) from the Brain Cell Atlas[30] show similar results (**Supplementary Fig. 7**). In addition, scDenorm shows a linear computational time complexity and memory usage with increasing number of cells and genes, demonstrating a high computational efficiency and scalability (**Supplementary Fig. 4c,d**).

scDenorm accurately recovers raw counts in different scenarios

In realistic scenarios, denormalising the normalised matrix deposited in the database can be affected by several aspects, including: 1) the parameters used in delta normalisation method; 2) the digital precision kept in the deposited data; and 3) the genes filtered after data normalisation

(**Fig. 4a**), (e.g., some lowly expressed genes could be removed). Using the 10x 3k PBMC single-cell dataset as a showcase, we benchmark these aspects.

We examined the effect of normalisation parameters (target sum, log-transformation base, and pseudo count) by simulating the normalisation process with eight sets of hierarchical parameters. The dataset was normalised using these parameters, and denormalised by scDenorm. As shown in **Fig. 4b**, the errors between the denormalised value and its raw count in all denormalised matrices are consistently low, as < 5×10^{-4}, indicating a minimal impact from normalisation parameters.

The digital precision of the normalised data, which can vary depending on the data processing tools and the saved file format, can also affect computational memory consumption. By default, the normalised data is saved as float32 (single-precision floating-point) format, with a precision of 6 to 9 decimal digits[33]. We simulated data with lower precision and denormalised it with scDenorm. The recovery error was less than 0.5 for count values less than 100, and less than one for count values greater than 100 (**Fig. 4c**). The errors are less than 0.01 when the digit precisions are more than 4 digits. The precision achieved with 3 to 4 decimal digits was consistent with the results of float16 conversion (**Fig. 4d**). Yet two decimal precision shows larger errors in highly expressing genes but keeps the cell identities (**Supplementary Fig. 8**).

In scRNA-seq data analysis, some genes expressed in few cells need to be removed or only selected genes may be kept in the normalised matrix. We simulated a gradient of the number of selected genes and tested the impact on denormalisation. No detectable error was found when more than 300 genes are kept in the normalised matrix, with the error increasing as the number of genes decreases (**Fig. 4e, Supplementary Fig. 9**). However, downstream data visualisation demonstrates that the denormalised matrices from float16 precision and a selection of 2000 highly variable genes successfully recovered the UMAP representation derived from raw counts (**Fig. 4f,g**), in spite of minor differences in the values.

scDenorm facilitates downstream analysis

We further evaluated the impact of denormalisation on downstream analysis tasks, including data integration, cell type annotation, differential expression (DE) analysis, and gene ontology (GO) analysis. As data from different batches may go through different normalisation, three datasets were prepared to cover different batch types. The batch in the COVID-19 PBMCs[34] dataset are samples from two patients, while in the human prefrontal cortex (PFC)[31,35] dataset are samples from two different studies. And the batch in the human skin[36] dataset are groups of samples from young and old donors.

The two patient samples in the COVID-19 PBMCs[34] dataset are first normalised with different target sums (1000 and 10,000) before going through downstream analysis (**Supplementary Fig. 10**). Firstly, if without denormalisation, the UMAP visualisation after harmony integration shows cells of the same cell type exist in multiple clusters, e.g., plasmablast (**Fig. 5a**). Subsequently, SCCAF[37], a well-established reference-based machine learning algorithm, was used to annotate

the cell types. The first sample was used as a reference to annotate the cell types in the second sample, resulting in an accuracy of 66% (the consistency between the original cell type labels and those assigned by SCCAF). Notably, CD14+ monocytes were misclassified as plasmacytoid dendritic cells (pDCs) and hematopoietic stem and progenitor cells (HSPCs), while CD8+ T cells were misclassified as natural killer (NK) cells and CD4+ T cells (**Fig. 5b**). Fortunately, with the help of scDenorm denormalisation, the two patient samples can be integrated, with each cell type cluster forming distinct clusters (**Fig. 5c**). And the accuracy of cell type annotation using SCCAF increased to 92%, indicating effective correction of denormalisation. Furthermore, mis-annotated cell type labels may result in biases in differential gene expression analysis (**Fig. 5d, Supplementary Fig. 10d**) and gene ontology analysis (**Supplementary Fig. 10e**). For instance, 350 differential genes in HSPCs matched the 'gold standard' after scDenorm, compared to only 81 before scDenorm (**Fig. 5e**). Gene enrichment analysis of the differential genes in HSPCs indicated that the GO terms enriched after scDenorm closely aligned with those of the 'gold standard', whereas the enrichment before scDenorm showed minimal overlap (**Supplementary Fig. 10f**). The enriched GO terms are relevant functions associated with HSPC cells, such as hematopoietic stem cell proliferation and hematopoietic progenitor cell differentiation (**Fig. 5f**).

Similarly, the same analysis of data integration and cell type annotation was performed on two other datasets, the human prefrontal cortex dataset and the human skin dataset. Both study-wise batch (the former dataset) and condition-wise batch (the latter dataset) demonstrated that data processed by scDenorm yielded superior integration results (**Fig. 6**, **Fig. 7**) and improved annotation results from SCCAF (**Fig. 7g,h**, **Supplementary Fig. 11d,e**). Yet, the mislabelled cell types lead to biased differentially expressed genes (DEGs) (**Fig. 7i**) and Gene Ontology (GO) terms (**Fig. 7j**).

However, the correct cell type annotation can also be achieved according to marker genes. We evaluate the impact of normalisation parameters on downstream DE and GO analysis. Taking the human skin data[36] as an example, differential gene expression analysis was performed before and after scDenorm using the cell type labels derived from the original publication, while the published differential expression genes (see **Methods**) and their resulted GO results were taken as 'gold standard'. The DE and GO results after scDenorm show higher consistency with the 'gold standard' than the results before scDenorm (**Fig. 6g,h**). In addition, the DEGs identified before scDenorm include more false positive genes (**Fig. 6i**), resulting in the enrichment of unrelated GO terms (**Fig. 6j**), such as the nuclear transport function for keratinocyte cells (**Supplementary Fig. 11f**).

Discussion

In our survey of 133 well-established single-cell studies, delta method normalisation takes up >83% (110) of the datasets (**Supplementary Table 3**), since it is implemented in widely-used SCANPY and Seurat analysis workflows. We demonstrate the capability of scDenorm on an example dataset and large-scale test sets from UCSC database[24] and the Brain Cell Atlas[30]. Different parameter sets in the delta method normalisation as well as the digital precision kept in the normalised data have minimal effect on denormalisation. Moreover, the number of genes kept

after normalisation does not significantly affect denormalisation, unless the number of genes used is too small (less than 300) (**Fig. 4e**). In the 40 datasets from UCSC database (**Supplementary Table 1**) and 60 datasets from Brain Cell Atlas (**Supplementary Table 2**), scDenorm successfully restored count values in the majority (88%) of the cases, with minimal rounding errors and recovery errors. Therefore, scDenorm may robustly recover matrices for the majority (estimated to be 80-90%) of the datasets, which are delta method normalised, while maintaining efficient computational speed (**Supplementary Fig. 4c**).

The limitations of scDenorm relies on specific prerequisites of the delta method normalisation. Datasets normalised using alternative methods may not be compatible with scDenorm. For example, GLM residual methods (such as SCTransfrom[32]) and latent expression (such as Sanity[38] and Dino[39]) cannot be denormalised by scDenorm. Fortunately, other denormalisation methods besides the delta method only constitute 10-20% of the datasets. And the raw counts of these datasets can be obtained from reads mapping. Additionally, the performance of scDenorm may be influenced by the choice of normalisation parameters and the quality of the input data. Cells whose gene expression distribution deviates from the assumptions of negative binomial distribution may lead to the failure of the denormalisation process.

Several case studies show that different normalisations can result in unnecessary deviations in downstream analysis, including data integration, cell type annotation, differential gene expression, gene ontology and pathway analysis. In particular, biased differential expression or gene ontology results can be generated due to different normalisation parameters when the cell type annotation is correct. Therefore, denormalising the expression matrix to raw counts can be a good choice to mitigate biases in downstream analysis. It could be a key question for large-scale data integration, where study-wise batch effects need to be minimised while biology should be kept. Batch correction as well as data integration methods have already been extensively discussed and benchmarked[8]. Here we highlight the consistency in data processing, which is nontrivial when data from tens or hundreds of studies need to be combined together. Consistent single-cell data analysis workflows which can both keep the raw conclusions from the publications and get integrated with data from other studies would greatly help. Therefore, the availability and reproducibility of the raw published analysis code would be important.

Conclusions

Here, we demonstrate that inconsistent data normalisation can generate unexpected bias in data integration, potentially obstructing atlas-level single-cell data integration. Fortunately, denormalising processed data back to raw counts could standardise analysis, thereby facilitating the creation of comprehensive cell atlases. We present scDenorm, a tool designed to denormalise data from the delta method normalisation, which are widely used by 80% of the 40 datasets in the UCSC database and 93% of the 60 datasets in Brain Cell Atlas. It employs both equation solving and regression methods to determine the parameters in the delta method. Benchmarks on 32 UCSC cell browser datasets and 56 Brain Cell Atlas datasets demonstrate the efficacy of scDenorm for delta method normalisation data, with further applications on COVID-19 PBMCs, prefrontal cortex, and human skin datasets revealing its ability to mitigate biases in downstream

analysis. scDenorm can be a useful tool in atlas level single-cell data processing and integration, such as the Human Cell Atlas[40], The Human Developmental Cell Atlas[41], the Brain Cell Atlas[30], and HuBMAP[42].

Methods

Assumption and algorithm design

In scRNA-seq, the data is in the form of a count matrix, where most entries are zeros due to the sparsity of gene expression. Our assumption is that the scRNA-seq data follows negative binomial distribution, which is theoretically and empirically well-supported for the unique molecular identifier data[17]. This means that probabilistically speaking, in the count matrix zero is the most frequently observed count, followed by one and two and so on. The sequential pattern of these values has a probabilistic one-to-one correspondence with the rank of their frequency by descending order (**Fig. 1f**). The smaller the values, the higher the probability of the correspondence (**Fig. 1g**). For example, without considering 0, the probability that the values 1 and 2 are equal to the rank of their frequency is almost 100%. Based on this assumption, we design an algorithm to normalise scRNA-seq data that has been normalised by the most commonly used delta methods, which scale the raw counts by the total number of counts (library size) and target sum (the summed value of the cell after scaling), and then log-transformed after adding a pseudo count (**Supplementary Fig. 3a**). Specifically, we consider a scaled expression matrix from a count matrix which has been transformed to adjust for differences in the scale of the features (e.g., genes) in the data. In scRNA-seq data, a scaled expression matrix typically refers to a count matrix that has been normalised and transformed to have a similar distribution of gene expression values across cells. For example, scRNA-seq data can be normalised to account for differences in sequencing depth and other technical factors that can affect the distribution of counts across cells and genes, such as total count normalisation. And it can be transformed to adjust for the distribution of gene expression values across cells such as log-transformation, and variance-stabilising transformation. The normalised gene expression matrix is derived from the count matrix to adjust for differences in gene expression across cells, which usually involves scaling and transformation techniques such as total count scaling and log-transformation. This normalisation process does not change the one-to-one correspondence between the gene expression value and its rank of the value's frequency in a cell.

Using the probabilistic one-to-one correspondence property, we can extract a cell vector from a normalised expression matrix and sort the values based on their frequency in the vector. This allows us to establish that the most frequently occurring non-zero value corresponds to one and the second most frequent represents two and so forth, which means the rank number and the count number are theoretically the same and this is normally true for the top ranks. By following this procedure, we were able to obtain the rank and normalised value pairs (C, N) (where C is the rank and N is the normalised count) for the equation $N = log_b^{(\frac{C}{s}+p)}$, (s is the scaling factor, b is the base of log-transformation, and p is the pseudo count). First, we try reversing log

transformation of natural base(e), base 2 and base 10, and solve the equation on the pairs of values (1, N_1) and (2, N_2), N_1 and N_2 are the values of the two most frequent numbers. Normally, we think the pseudo count C is given as 1. Otherwise, we need to check whether the variance of the solved C from different cells is sufficiently small, since each vector from the gene expression matrix has been augmented with the same pseudo count. If the unscaling process is unsuccessful for all of the above cases, we conclude that the matrix has not been pre-processed according to the workflow. The following shows the complete workflow of scDenorm algorithm.

The denormalisation algorithm can be divided into two steps, de-transformation and unscaling. In de-transformation, there are two sequential steps. Firstly, (a) we search for empirical values for the log-transformation bases and the pseudo count. It searches for empirical bases such as 2, e (natural base), and 10, as well as common pseudo counts like 0, 0.01, 0.1, and 1. If the pseudo count is 0, it indicates that the normalisation process has not added the pseudo count. A fraction of cells is used to evaluate if any of these bases or pseudo counts meet the criteria in step (c). If passing the criteria, skip to step 2. Otherwise, it goes to step (b) to determine the parameters. Step (b) uses equation solving method to determine the parameters: This method uses the two values (N_1, N_2) occurring most frequently in a cell to construct the following equation. For each cell i:

$$\frac{1}{s_i} + p = b^{N_1^i} \quad (1)$$

$$\frac{2}{s_i} + p = b^{N_2^i} \quad (2)$$

s_i is the scaling factor for the cell i. The p and b are pseudo count and base. From equation (1) and (2), we can get equation (3).

$$p = 2 \times b^{N_1^i} - b^{N_2^i} \quad (3)$$

Randomly select a group of cells (e.g., n=100) to generate a corresponding set of data points (N_1, N_2), and solve p and b by equation (4) with optimization methods.

$$argmin \sum_{i=1}^{n} \quad (p - 2 \times b^{N_1^i} + b^{N_2^i})^2 \quad (4)$$

The L-BFGS-B method from the sklearn[43] package is used to find the best base (b) and pseudo count (p). This method is based on the limited-memory Broyden-Fletcher-Goldfarb-Shanno (BFGS) algorithm, which is capable of large-scale optimization. L-BFGS-B allows for box constraints, ensuring that the parameters stay within specified bounds during optimization.

After the de-transformation, the sum of each cell should be the same or very similar. Step (c). checks if the sum of each cell is the same. For example, let X as the vector of the sums, x is a number in it. And if abs(x-mean(X)) is always smaller than the small number (*e.g.*, mean(X)=10000, x=9999.7, small number is 0.5), then the de-transformation is successful. However, this is an ideal situation. Often, we encounter that after normalisation, the data filters out some genes for the quality control in downstream analysis. In addition, some normalisation methods do not scale the total expression values to the same for all cells. To address these complex cases, we also added the following criteria. If it is the automatic detection method, we only need to make sure that the mean square error (MSE) is small enough, such as 10^{-5}. In general, we just need to unscale a cell to see if it is successful.

In unscaling, we have two approaches implemented in the same function, while a parameter can be used to select the option. The first approach (a) is based on regression to determine the scaling factors for all cells. The scaling factor is derived from fitting a regression model to the relationship between the de-transformed values and their ranks, providing an estimate of the scaling factor for each cell. For each cell:

$$\arg\min_{s} \sum_{i=1}^{n} \left(\frac{c_i}{s} + p - x_i\right)^2 \quad (5)$$

c_i is the rank, and x_i is the de-transformed value.
To ensure a more accurate one-to-one correspondence, only the first 5 pairs of values (c_i, x_i) are used. We can get the scaling factor s by optimising the equation (5) using the same L-BFGS-B method as in solving equation (4).

The second approach (b) is solving equations between the top two most frequent values: This method only used the first 2 pairs of values (c_i, x_i). We can get a closed form of solution by solving the following equation. For each cell:

$$\frac{c_1}{s} + p = x_1 \quad (6)$$

$$\frac{c_2}{s} + p = x_2 \quad (7)$$

From equation (6) and (7), we can get equation (8).

$$s = \frac{c_2 - c_1}{x_2 - x_1} \quad (8)$$

To evaluate the success of the denormalisation process, we measure the error between the denormalised value and its rounded value. Ideally, after denormalisation, the values should closely approximate integers. We can calculate abs(round(x)-x) and check if it is smaller than a certain number. Of note, in some cases, the same top value (e.g., 1) can be normalised into more than 1 different values due to some improper data processing, and the ranks of these numbers are thus lower than expected. These numbers with tiny differences are merged as one value by decreasing their digital precision.

scDenorm is publicly available as an open-source Python package and provides a user-friendly python function interface, which can be combined in the use of SCANPY analysis. It can be used both at the command line and interactively in Jupyter notebook. Description of the function details are provided in **Supplementary Materials**. Considering that different samples in a dataset may be normalised with different parameter sets, scDenorm also implements a per sample denormalisation function overloading the original ‘scdenorm’ function with a ‘by=sample’ parameter as input.

Integration of scRNA-seq data from different normalisation parameters

We downloaded a PBMCs scRNA-seq data from the 10x genomics datasets (http://cf.10xgenomics.com/samples/cell-exp/1.1.0/pbmc3k/pbmc3k_filtered_gene_bc_matrices.tar.gz), and preprocessed and annotated the data according to this tutorial (https://scanpy-tutorials.readthedocs.io/en/latest/pbmc3k.html). Then, we used different parameter combinations (including 1e3, 1e4, 1e5, 1e6 as target sums, 2, e, 10 as base, and 1,0.1, 0.001 as pseudo counts) to normalise the data separately and merge all the data together. Principal Component Analysis (PCA) of 50 components was derived from the expression matrix. Three single-cell data integration tools (Harmony, BBKNN, scanorama) were tested to integrate the combined data with the normalisation parameters as the batch key. For data visualisation, Uniform Manifold Approximation and Projection (UMAP)[44] is calculated in the integrated latent space or the PCA space.

Consistency of count-rank relationship across sequencing platforms

The consistency of count-rank relationship refers to the percentage of the correct one-to-one correspondence between the gene expression value and its rank of the value's frequency in a cell. For example, given 100 cells, we first calculate the frequency of the raw count values (the raw count value called as count) in each cell, and order the frequencies from highest to lowest. The order is called as rank, which ranges from 1,2, …, n. If count is the same as rank, we consider this to be a correct one-to-one correspondence. Finally, for the count from 1 to 10, we respectively calculate what percentage of cells have the correct one-to-one correspondence as the consistency of the count-rank relationship. To compare different sequencing platforms, we calculated the consistency of the count-rank relationship in 105 datasets obtained from the Brain Cell Atlas (http://braincellatlas.org). Among these datasets, 81 are from Chromium, 15 from Drop-seq, and 9 from Smart-seq2.

Evaluation metrics

When benchmarking denormalisation for scRNA-seq data, two measures can be used: rounding error and recovery error. Rounding error measures the discrepancy between the denormalised values and their rounded counterparts. After denormalisation, the expected outcome is that the denormalised values approximate integers. Rounding error quantifies the extent to which the denormalised values deviate from integers. To calculate rounding error, the difference between each denormalised value and its rounded value is computed, see equation (9) below. Recovery error evaluates the difference before denormalisation and after re-normalising the denormalised values (values after scDenorm, **Fig. 3a**). To calculate recovery error, the difference between each normalised value and its re-normalised value is computed, see equation (10) below.

Specifically, we assume x is the normalised value (a single value for one gene in one cell), y is the denormalised value after scDenorm, and z is the re-normalised value from denormalised value (y). The rounding error is calculated as the difference between the denormalised value (y) and its rounded value, equation (9):

$$rounding\_error = round(y) - y \quad (9)$$

The recovery error is calculated as the difference between the normalised value (x) and the re-normalised value (z), equation (10):

$$recovery\_error = x - z \quad (10)$$

In certain cases, not all cells can be successfully denormalised due to poor sequencing quality, or a low number of expressed genes. To evaluate denormalisation in such situations, we define success rate as the percentage of successfully denormalised cells, equation (11).

$$success\ rate = Nsuccess/Ntotal \quad (11)$$

Nsuccess is the number of successfully denormalised cells, while Ntotal is the total number of cells.

Benchmark scDenorm based on digital precision and gene filtering

To assess the impact of different digital precision of normalised data on the denormalisation process, we performed the following steps on the PBMC data: First, we applied total-count normalisation (the normalize_total function in SCANPY[19]) to the data matrix with target sum as 10,000, and log-transform (natural base, e) the data with 1 as pseudo count. Next, we used the round function to retain the data at different levels of precision, ranging from 2 to 8. Float16 corresponds to 3 to 4 decimal places of precision, while float32 corresponds to 6 to 9 decimal places of precision. Finally, we denormalise the data separately for each precision level and compare the results with rounding errors to evaluate their effects.

To test our algorithm for gene filtering on extreme cases, we selected a series of highly variable genes, ranging from 100, 200, 300, 400, 500, 1000, 2000 to 5000. Specifically, First, we normalise the data by sc.pp.normalise_total with target_sum as 10,000 and logarithmic the data with sc.pp.log1p. The high-variable genes are then selected using sc.pp.highly_variable_genes with layer as ‘count’ and flavour as ‘seruat_v3’. Finally, we use scDenorm to denormalise the data and calculate the recovery errors.

Benchmark on large-scale datasets

To perform the usage of our algorithm on atlas data, we downloaded 40 datasets from the UCSC Cell Browser and 60 datasets from the Brain Cell Atlas, ensuring that they encompass a diverse range of species, sequencing platforms, and normalisation methods. First, we used scDenorm to

denormalise each dataset. If successful, we calculate the rounding errors for the dataset, which quantifies the difference between the denormalised values before and after rounding. Additionally, if all total values (the sum of all the denormalised values in each cell) of the dataset are close to a fixed value (target sum, e.g., 10,000) after de-transformation, the recovery errors are calculated.

Dataset processing for data integration and downstream analysis

The COVID-19 PBMC dataset from Arunachalam et al.[34] (**Fig. 5**) was downloaded from GEO[45] under the accession code GSE155673. Two samples Arunachalam_cov11 (S1) and Arunachalam_cov11 (S2) were processed with different delta normalisation parameters: S1 was normalised by target sum 1e3, while S2 was normalised by target sum 1e4. And both were log-transformed. For data visualisation, we performed Harmony[13] data integration of these two samples after PCA of 50 components. For cell type annotation, SCCAF[37] was used. S2 was used as the reference for annotating S1.

The human skin dataset from Solé-Boldo et al., was downloaded from GEO under the accession code GSE130973 (**Fig. 6**), including two young (25 and 27 years old) and three old (53, 69 and 70 years old) donors. The young samples were normalised to target sum 1e3, while the old samples were normalised to target sum 1e4. And both samples were logarithmic transformed after normalisation. For cell type annotation, the old samples were used as the reference for annotating the young sample.

The human prefrontal cortex data includes datasets from two studies, Ma et al.[35] (170,000 cells) and Velmeshev et al.[31] (100, 000 cells) (**Fig. 7**). The Velmeshev's dataset was normalised to target sum 1e3 and logarithmic transformation, while the Ma's dataset was not normalised. Harmony was used for data integration. For cell type annotation, the Ma's dataset was used as the reference for annotating Velmeshev's dataset.

For data processing after denormalisation with scDenorm, we follow a standard workflow of data normalisation and dimension reduction. Specifically, the expression matrix is normalised to target sum of 10,000 and log-transformed. And the default dimension reduction process in the SCANPY workflow was used, including PCA, Harmony integration and UMAP visualisation. SCCAF was used to predict the cell types as described above.

Downstream analysis after cell type annotation includes differential expression (DE) analysis and Gene Ontology (GO) pathway analysis. Differential gene expression analysis was conducted for each cell type (one against the rest) using the Wilcoxon test implemented in SCANPY[19]. As part of the dataset is used as the reference dataset, the differentially expressed genes (DEGs) derived from the reference dataset is used as 'gold standard' in our evaluation. The same approach was used for the calculation of the DEGs before and after scDenorm. The top differentially expressed genes were compared across different thresholds (top 50, 100, 200, 500, and 1000). As for GO pathway analysis, the enrichGO program was used on the top 500 differential expression genes.

DE and GO analysis with the correct labels from Solé-Boldo et al. was conducted for each cell type (one against the rest) using the Wilcoxon test implemented in Seurat (V3.1.1), same as the version described in Solé-Boldo et al. These analyses were conducted before scDenorm and after scDenorm. The differentially expressed genes were obtained from the study's supplementary materials as 'gold standard'.

Availability of Data and Materials

The 10x 3k PBMC data was downloaded from the 10x genomics website https://support.10xgenomics.com/single-cell-gene-expression/datasets/1.1.0/pbmc3k. The dataset with both the normalised expression and the raw count matrix was downloaded from https://autism.cells.ucsc.edu. 40 processed datasets (**Supplementary Table 1**) were downloaded from https://cells.ucsc.edu. 60 processed datasets (**Supplementary Table 2**) were downloaded from https://www.braincellatlas.org. Datasets used in the manuscript have been deposited at zenodo: https://doi.org/10.5281/zenodo.11240443. Codes for reproducing this work are available via GitHub at: https://github.com/rnacentre/scDenorm_reproducibility.

The source codes and documentation of scDenorm are available at https://github.com/rnacentre/scDenorm. It can also be installed via pip or anaconda, at: https://pypi.org/project/scDenorm/ and https://anaconda.org/changebio/scdenorm, respectively. This software is licensed under the Apache License 2.0, an open source licence compliant with Open Source Initiative. scDenorm has been registered in bio.tools (biotoolsID: scdenorm) and SciCrunch.org (RRID: SCR_027574).

Declarations

Ethics approval and consent to participate

Not applicable.

Consent for publication

Not applicable.

Competing interests

The author declares no competing interests.

Author's contributions

Z.M. and A.B. designed and conceived the study. Y.H., and Z.M. implemented the scDenorm algorithm, Y.H. conceived and performed most of the bioinformatics analyses. Y.A. performed

part of the analysis. H.Z., Y.Z., S.L., X.Y., and M.S. provided some datasets. Y.H., Z.M., A.V.P., Y.A., I.P. wrote the manuscript. Z.M., M.S., X.Y. supervise the study.

Acknowledgements

The authors thank Ziliang Huang for help with the datasets.

Funding

This work was supported by the National Key R&D Programs of China (2023YFF1204700, 2024YFF1206600), the Natural Science Foundation of China (32270707), the Major Project of Guangzhou National Laboratory (grant nos GZNL2024A01002 and GZNL2023A01006), the R&D Programs of Guangzhou National Laboratory (grant nos HWYQ23-003 and YW-YFYJ0102), and Postdoctoral Research Project Funding of Guangzhou, BSHF23-049.

References

1. Butler, A., Hoffman, P., Smibert, P., Papalexi, E. & Satija, R. Integrating single-cell transcriptomic data across different conditions, technologies, and species. *Nat. Biotechnol.* **36**, 411–420 (2018).
2. Ren, X. *et al.* COVID-19 immune features revealed by a large-scale single-cell transcriptome atlas. *Cell* **184**, 1895–1913.e19 (2021).
3. Regev, A. *et al.* The Human Cell Atlas. *Elife* **6**, (2017).
4. Travaglini, K. J. *et al.* A molecular cell atlas of the human lung from single-cell RNA sequencing. *Nature* **587**, 619–625 (2020).
5. Elmentaite, R. *et al.* Single-Cell Sequencing of Developing Human Gut Reveals Transcriptional Links to Childhood Crohn's Disease. *Dev. Cell* **55**, 771–783.e5 (2020).
6. Sikkema, L. *et al.* An integrated cell atlas of the lung in health and disease. *Nat. Med.* **29**, 1563–1577 (2023).
7. Song, Y., Miao, Z., Brazma, A. & Papatheodorou, I. Benchmarking strategies for cross-species integration of single-cell RNA sequencing data. *Nat. Commun.* **14**, 6495 (2023).

8. Luecken, M. D. *et al.* Benchmarking atlas-level data integration in single-cell genomics. *Nat. Methods* **19**, 41–50 (2022).

9. Büttner, M., Miao, Z., Wolf, F. A., Teichmann, S. A. & Theis, F. J. A test metric for assessing single-cell RNA-seq batch correction. *Nat. Methods* **16**, 43–49 (2019).

10. Lopez, R., Regier, J., Cole, M. B., Jordan, M. I. & Yosef, N. Deep generative modeling for single-cell transcriptomics. *Nat. Methods* **15**, 1053–1058 (2018).

11. Xu, C. *et al.* Probabilistic harmonization and annotation of single-cell transcriptomics data with deep generative models. *Mol. Syst. Biol.* **17**, e9620 (2021).

12. Stuart, T. *et al.* Comprehensive Integration of Single-Cell Data. *Cell* **177**, 1888–1902.e21 (2019).

13. Korsunsky, I. *et al.* Fast, sensitive and accurate integration of single-cell data with Harmony. *Nat. Methods* **16**, 1289–1296 (2019).

14. Liu, J. *et al.* Jointly defining cell types from multiple single-cell datasets using LIGER. *Nat. Protoc.* **15**, 3632–3662 (2020).

15. Vallejos, C. A., Risso, D., Scialdone, A., Dudoit, S. & Marioni, J. C. Normalizing single-cell RNA sequencing data: challenges and opportunities. *Nat. Methods* **14**, 565–571 (2017).

16. Bacher, R. *et al.* SCnorm: robust normalization of single-cell RNA-seq data. *Nat. Methods* **14**, 584–586 (2017).

17. Ahlmann-Eltze, C. & Huber, W. Comparison of transformations for single-cell RNA-seq data. *Nat. Methods* **20**, 665–672 (2023).

18. Satija, R., Farrell, J. A., Gennert, D., Schier, A. F. & Regev, A. Spatial reconstruction of single-cell gene expression data. *Nat. Biotechnol.* **33**, 495–502 (2015).

19. Wolf, F. A., Angerer, P. & Theis, F. J. SCANPY: large-scale single-cell gene expression data analysis. *Genome Biol.* **19**, 15 (2018).

20. Grün, D., Kester, L. & van Oudenaarden, A. Validation of noise models for single-cell transcriptomics. *Nat. Methods* **11**, 637–640 (2014).

21. Cao, Y., Kitanovski, S., Küppers, R. & Hoffmann, D. UMI or not UMI, that is the question for scRNA-seq zero-inflation. *Nat. Biotechnol.* **39**, 158–159 (2021).

22. Kharchenko, P. V. The triumphs and limitations of computational methods for scRNA-seq. *Nat. Methods* **18**, 723–732 (2021).

23. Svensson, V. Droplet scRNA-seq is not zero-inflated. *Nat. Biotechnol.* **38**, 147–150 (2020).

24. Speir, M. L. *et al.* UCSC Cell Browser: visualize your single-cell data. *Bioinformatics* **37**, 4578–4580 (2021).

25. Chen, W. *et al.* UMI-count modeling and differential expression analysis for single-cell RNA sequencing. *Genome Biol.* **19**, 70 (2018).

26. Townes, F. W., Hicks, S. C., Aryee, M. J. & Irizarry, R. A. Feature selection and dimension reduction for single-cell RNA-Seq based on a multinomial model. *Genome Biol.* **20**, 295 (2019).

27. Li, M. *et al.* DISCO: a database of Deeply Integrated human Single-Cell Omics data. *Nucleic Acids Res.* **50**, D596–D602 (2022).

28. Hie, B., Bryson, B. & Berger, B. Efficient integration of heterogeneous single-cell transcriptomes using Scanorama. *Nat. Biotechnol.* **37**, 685–691 (2019).

29. Polański, K. *et al.* BBKNN: fast batch alignment of single cell transcriptomes. *Bioinformatics* **36**, 964–965 (2020).

30. Chen, X. *et al.* A brain cell atlas integrating single-cell transcriptomes across human brain regions. *Nat. Med.* (2024) doi:10.1038/s41591-024-03150-z.

31. Velmeshev, D. *et al.* Single-cell genomics identifies cell type-specific molecular changes in autism. *Science* **364**, 685–689 (2019).

32. Hafemeister, C. & Satija, R. Normalization and variance stabilization of single-cell RNA-seq data using regularized negative binomial regression. *Genome Biol.* **20**, 296 (2019).

33. Muller, J.-M. *et al. Handbook of Floating-Point Arithmetic*. (Springer International Publishing).

34. Arunachalam, P. S. *et al.* Systems biological assessment of immunity to mild versus severe COVID-19 infection in humans. *Science* **369**, 1210–1220 (2020).

35. Ma, S. *et al.* Molecular and cellular evolution of the primate dorsolateral prefrontal cortex. *Science* **377**, eabo7257 (2022).

36. Solé-Boldo, L. *et al.* Single-cell transcriptomes of the human skin reveal age-related loss of fibroblast priming. *Commun Biol* **3**, 188 (2020).

37. Miao, Z. *et al.* Putative cell type discovery from single-cell gene expression data. *Nat. Methods* **17**, 621–628 (2020).

38. Breda, J., Zavolan, M. & van Nimwegen, E. Bayesian inference of gene expression states from single-cell RNA-seq data. *Nat. Biotechnol.* **39**, 1008–1016 (2021).

39. Brown, J., Ni, Z., Mohanty, C., Bacher, R. & Kendziorski, C. Normalization by distributional resampling of high throughput single-cell RNA-sequencing data. *Bioinformatics* **37**, 4123–4128 (2021).

40. Rozenblatt-Rosen, O., Stubbington, M. J. T., Regev, A. & Teichmann, S. A. The Human Cell Atlas: from vision to reality. *Nature* **550**, 451–453 (2017).

41. Haniffa, M. *et al.* A roadmap for the Human Developmental Cell Atlas. *Nature* **597**, 196–205 (2021).

42. HuBMAP Consortium. The human body at cellular resolution: the NIH Human Biomolecular Atlas Program. *Nature* **574**, 187–192 (2019).

43. Pedregosa, F. *et al.* Scikit-learn: Machine Learning in Python. *J. Mach. Learn. Res.* **abs/1201.0490**, (2011).

44. McInnes, L., Healy, J., Saul, N. & Großberger, L. UMAP: Uniform Manifold Approximation and Projection. *J. Open Source Softw.* **3**, 861 (2018).

45. Barrett, T. *et al.* NCBI GEO: archive for functional genomics data sets--update. *Nucleic Acids Res.* **41**, D991–5 (2013).

Figure legends

Fig. 1 The data distribution of droplet-based single-cell data

a, UMAP plot of PBMC 3k datasets, without data integration, normalised with different delta normalisation parameters, including target sum (L), logarithmic base (b) and pseudo count (p). The plot is coloured by different parameter sets.
b, The same UMAP plot as panel (**a**) coloured according to cell type annotation.
c, UMAP plot after Harmony integration of data normalised by different parameters, coloured by cell types.
d, The UMAP plots are the same as panel (**c**) (after data integration by Harmony), displaying four different normalisation parameter sets.
e, Scatter plot demonstrating the mean (x-axis) against the variance (y-axis) for each gene in the count matrix of the PBMC 3k datasets. Each dot shows the mean and variance value of a gene. The diagonal line is shown in blue. The orange curve is the fitted curve of negative binomial distribution with variance v, mean μ and dispersion φ.
f, Histogram depicting the frequencies of count values and their ranks in a single cell, showing the 'count-rank' distribution in a cell selected from the count matrix of the PBMC 3k datasets.
g, The percentage of cells that follow the 'count-rank' distribution (the value of count equal to its rank from 1 to 5) in three scRNA-seq technologies (Chromium, Smart-seq2, and Drop-seq).

Fig. 2 Evaluation of scDenorm on normalised scRNA-seq data with known raw counts

a, The scatter plot shows the distribution between expression values and their ranks of frequencies in three example cells. Each dot is an expression value and its rank of frequency in the cell, different cells are shown in different shape and colour.
b, the scatter plot shows the distribution between the log-transformed mean expression (x-axis) and the log-transformed variance (y-axis) for each gene in the gene expression matrix from Velmeshev et al dataset. The diagonal line (x=y) is shown in blue.
c, The scatter plot shows the distribution between the most and second most frequent values in different cells, displaying each cell as a dot. The blue curve shows the fitted equation derived from the equation (4) in **Methods**, with base value (b) equal to 2 and pseudo count (p) equal to 1. N_1 and N_2 are the most and second most frequent values in cells.
d, The scatter plot shows the distribution between expression values and their ranks of frequencies in the three example cells after de-transformation, coloured in the same manner as panel (**a**).
e, The scatter plot shows the relationship between the mean expression (x-axis) and variance (y-axis) for each gene after de-transformation.

f, The scatter plot shows the distribution between the number of genes and the target sum (sum of all expression values) in the cell after de-transformation.

g, The scatter plot shows the distribution between expression values and their ranks of frequencies in the three example cells after unscaling, displaying the 'count-rank' distribution.

h, The dot plot shows the distribution between the mean expression (x-axis) with the variance (y-axis) for each gene in the count matrix.

i, The scatter plot shows the distribution between the count values and the rounding errors after denormalisation. Each dot represents a count value in a cell and its rounding error.

Fig. 3 Performance of scDenorm on normalised scRNA-seq data from UCSC Cell Browser

a, The diagram illustrates the workflow of scDenorm to evaluate denormalisation using the rounding and recovery error. The processed matrix (left) deposited in the UCSC database is first denormalised (middle) with scDenorm to calculate the rounding error (see **Methods**). Subsequently, the denormalised matrix (middle) is re-normalised (right) to measure the recovery error (see **Methods**). The values of target sum and pseudo count normalisation parameters are 1e4 and 1, respectively.

b, Two barplots show the number of genes (top) and number of cells (bottom) for the collected UCSC datasets. The x-axis shows the datasets by name, while the y-axis shows the log-scaled number of genes (top) and the log-scaled number of cells (bottom). The colours represent the different parameters of the delta normalisation. Blue and orange are natural base (e) and base=2 respectively, green represents data without log-transformation. Red shows non-delta method normalisation cases, which could not be denormalised by scDenorm.

c, The pie chart shows the distribution of the number of datasets classified by different base values, detected by scDenorm. The colours are the same as shown in panel (**b**). Unknown represents non-delta method normalisation cases.

d, The bar plot shows the distribution of the success rate (see **Methods**) across the 27 datasets that were normalised with natural logarithmic transformation.

e, The jitter plot shows the distribution of rounding errors observed in the denormalised datasets. The x-axis is the rounding error, while the y-axis shows the same datasets as panel (**d**).

f, The box plot shows the distribution of recovery errors after re-normalization with the parameters of target sum, pseudo count and logarithm base as 1e4, 1 and natural base (e), respectively. The x-axis is the recovery error, while the y-axis shows the datasets in the same order as in panel (**d**).

Fig. 4 Benchmark of scDenorm on different normalisation parameters, digital precision and gene filtering

a, The diagram shows the workflow of evaluating the recovery errors of denormalisation in three scenarios, different normalisation parameters, different digit precision, and gene filtering, on the PBMC 3k dataset. The raw count matrix (top) is first normalised with different parameter sets (middle), and then denormalised (bottom) with scDenorm giving different digit precisions and filtered genes. In delta method normalisation: C is the count value; T is the total count value of a

cell; L is the target sum; p is the pseudo count; b is the base of logarithmic function; and N is the normalised gene expression values.

b, The line plot shows the distribution of recovery errors from using different parameter sets in delta normalisation.

c, The dot plot shows the distribution between raw count values and their recovery errors after the conversion of normalised data from float32 to float16.

d, The line plot shows the distribution of recovery errors from normalised float32 data while preserving different levels of digit precision (2-8 digit precisions).

e, The histogram shows the mean square error of regression loss of equation (4) (see **Methods**) from normalised data with different numbers of highly variable genes (from 100 to 5,000).

f, The UMAP plot shows the distribution of data processed using different approaches, including original processed data (blue), denormalised data after converting to float16 (orange), and denormalised data after selecting 2,000 highly variable genes (green).

g, The UMAP plot shows the cell type distribution of panel (**f**), colour-coded by cell types.

Fig. 5 Different normalisations impact cell type annotation of COVID-19 PBMCs

a, The UMAP plot shows the distribution of cells before denormalisation by scDenorm, coloured by predicted cell type annotation (see **Methods**).

b, The UMAP plot shows the distribution of cells after Harmony data integration followed by scDenorm. Cells are coloured by predicted cell type annotation.

c. The heatmap shows the confusion matrix between the published cell type labels and the predicted cell types based on the Harmony-integrated latent space before scDenorm denormalisation. The x-axis represents predicted cell types, while the y-axis denotes original cell type annotation published in the study.

d. The histogram shows the percentage of DEGs overlap between the ‘gold standard’ (DEGs derived according to the original published cell type labels) and the ones derived from re-analysis before (blue) and after (orange) scDenorm across cell types. The DEGs are calculated with a two-sided Wilcoxon test using the predicted cell types as clusters.

e. The Venn diagram shows the overlap of the top 500 DEGs for HSPCs derived from the 'gold standard' (blue) from original study, as well as before (orange) and after (red) scDenorm.

f. The heatmap shows the enriched Gene Ontology (GO) terms of HSPCs derived from DEGs of the 'gold standard' (blue) from original study, as well as before (orange) and after (red) scDenorm.

Fig. 6 Different normalisations impact differential expression analysis

a, The UMAP plot shows the cell distribution of the two age groups of the human skin dataset (Solé-Boldo et al.) before data integration without the scDenorm denormalisation, coloured by age groups.

b, The UMAP plot shows the Harmony-integrated result without the scDenorm denormalisation, coloured by age groups.

c, The UMAP plot shows the Harmony-integrated result after running scDenorm, coloured by age groups.
d, The UMAP plots show the same distribution as panels (**a**), coloured by original cell type labels from Solé-Boldo et al.
e-f, The UMAP plots show the same distribution as panels (**b-c**), but coloured by predicted cell type labels before and after denormalisation by scDenorm.
g-h, The histograms show the percentages of DEGs (**g**) and GO terms (**h**) overlap between the 'gold standard' (DEGs extracted from the data in the original study, GO terms derived from these DEGs) and data before (purple) and after (blue) scDenorm across cell types, using the 'gold standard' cell type labels reported in the original publication. The DEGs are calculated with a two-sided Wilcoxon test based on the original cell type from the human skin dataset (Solé-Boldo et al.), while the GO analysis shows Benjamini-Hochberg-adjusted P value <0.05.
i-j, The Venn diagrams show the overlaps of the DEGs (**i**) (Benjamini-Hochberg-adjusted P value <0.05 and logFC >0.25) and GO terms (**j**) for differentiated keratinocytes derived from data of the 'gold standard' (red), as well as before (purle) and after (blue) scDenorm denormalisation.

Fig. 7 scDenorm helps in cell type annotation and differential expression analysis on prefrontal cortex datasets

a, The UMAP plot shows the distribution of cells of the two prefrontal cortex datasets before integration.
b, The UMAP plot shows Harmony-integrated results without the scDenorm denormalisation, coloured by studies.
c, The UMAP plot shows the Harmony-integrated result after running scDenorm, coloured by studies.
d, The figure legend of cell type annotation for panel (**e**) and (**f**).
e, The UMAP plot is the same as (**b**) coloured by predicted cell type annotation.
f, The UMAP plot is the same as (**c**) coloured by predicted cell type annotation.
g-h, River plot illustrates the transition between original and predicted cell types before scDenorm (**g**) and after scDenorm (**h**). The left side represents the original cell types from Velmeshev et al., while the right side displays the predicted cell types.
i, Bar plot showing the overlapping percentage of DEGs between the gold standard with before and after scDenorm across cell types. The DEGs are calculated with a two-sided Wilcoxon test based on the predicted cell types.
j, Bar plot showing the overlapping percentage of GO terms between the gold standard with before and after scDenorm across cell types.

Figure

Click here to access/download;Figure;Fig7.pdf

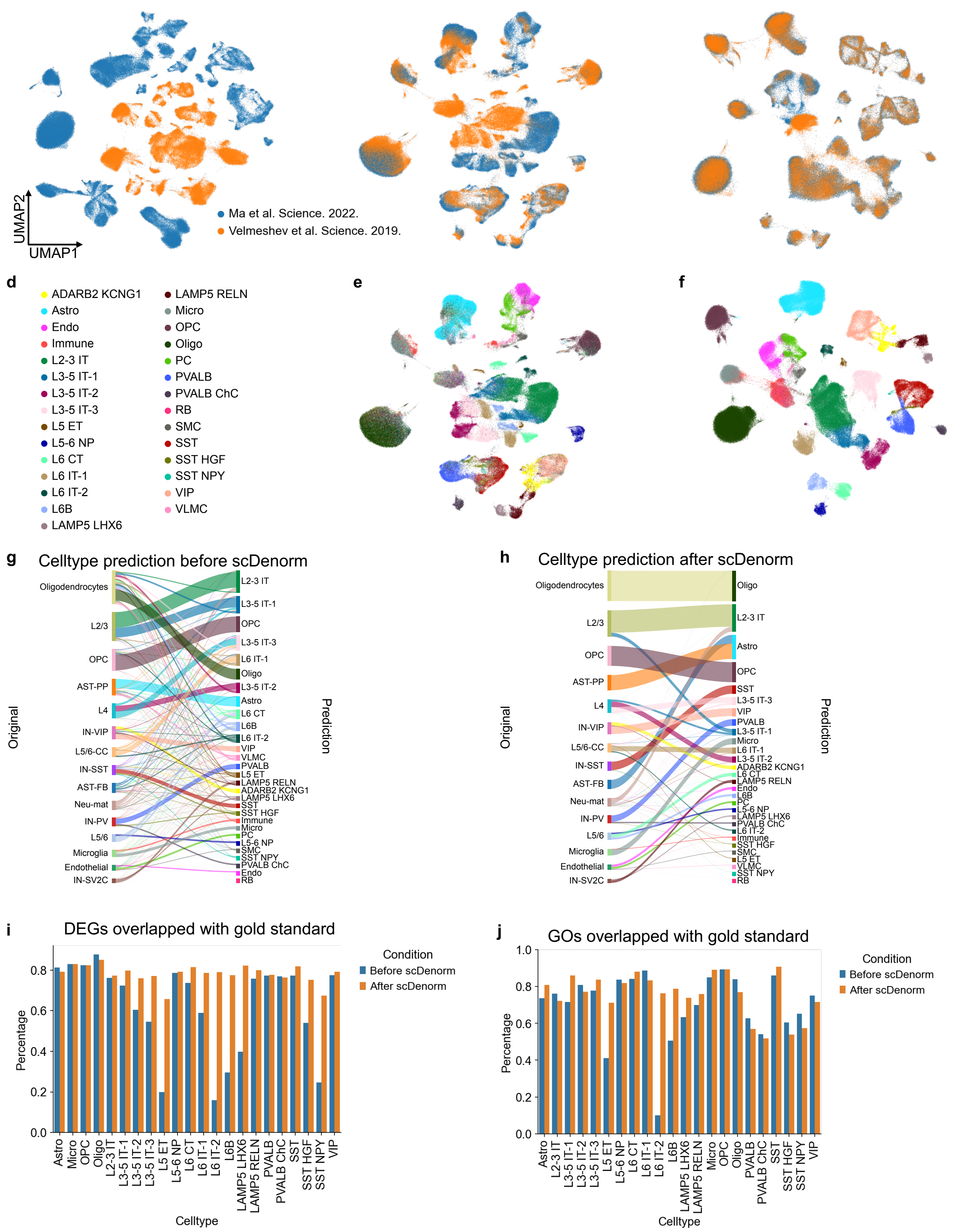

Click here to access/download
Supplementary Material
SupplementaryInformation_R2.pdf

Personal Cover
Click here to access/download;Personal Cover;CoverLetter (1).pdf

Chichau Miao, PhD Principal Investigator
Guangzhou National Laboratory, Guangzhou, China
Guangzhou Medical University, Guangzhou, China
miao_zhichao@gzlab.ac.cn
Lab page: www.rnacentre.org
+86 (0)13205837785

Nov 1, 2025

Dear Nogoy,

We would like provide you our revised manuscript:
scDenorm: a denormalisation tool for integrating single-cell transcriptomics data
for your consideration for publication at ***GigaScience***.

Thank you for the valuable feedback on our manuscript. We have carefully addressed all reviewer comments and made substantial improvements to enhance the clarity, reproducibility, and usability of our work.

Key updates include:

1. We have clarified the reviewers' concerns regarding the application of our methods and the related figures in the manuscript. As suggested, the human prefrontal cortex (PFC) integration example has been promoted to main Figure 7, demonstrating the utility of scDenorm across studies with different pipelines.

2. We have provided a ready-to-use Docker image along with detailed environment files (environment.yaml for Python and installed_packages.csv for R) to ensure reproducibility. All required input data, metadata, and pre-computed results are now available via Zenodo (https://zenodo.org/uploads/17275776).

3. Additionally, scDenorm has been registered in bio.tools (biotoolsID: scdenorm) and SciCrunch.org (RRID: SCR_027574), with the corresponding identifiers included in the manuscript.

We look forward to hearing from you. Thank you in advance for sharing our enthusiasm!

Sincerely,

Zhichao (Chichau) Miao
Guangzhou Laboratory

 Click here to access/download;Personal Cover;Rebuttal_v1.pdf

Dear Nogoy,

We gratefully appreciate your feedback and all the comments from the reviewers. The constructive feedback helped us to improve our manuscript. We have carefully addressed the concerns raised by the reviewers. Please find our point-by-point responses below:

**Comments**	**Answers**
Editor	
**0.0** Your manuscript "scDenorm: a denormalisation tool for integrating single-cell transcriptomics data" (GIGA-D-25-00209) has been assessed by our reviewers. Although it is of interest, we are unable to consider it for publication in its current form. The reviewers have raised a number of points which we believe would improve the manuscript and may allow a revised version to be published in GigaScience.<br><br>Their reports, together with any other comments, are below. Please also take a moment to check our website at https://www.editorialmanager.com/giga/ for any additional comments that were saved as attachments.<br><br>In addition, please register any new software application in the bio.tools and SciCrunch.org databases to receive RRID (Research Resource Identification Initiative ID) and biotoolsID identifiers, and include these in your manuscript. Computational workflows should be registered in workflowhub.eu and the DOIs cited in the relevant places in the manuscript. These will facilitate tracking, reproducibility and re-use of your tool.	Thank you for the feedback! We have carefully addressed all reviewer comments and made substantial improvements to enhance clarity, reproducibility, and usability.<br><br>Key updates include:<br><br>• The **human prefrontal cortex (PFC)** integration example, which demonstrates the utility of *scDenorm* across studies with different pipelines, has been promoted to **main Figure 7**, as suggested.<br>• A **ready-to-use Docker image** and detailed environment files (`environment.yaml` for Python and `installed_packages.csv` for R) to ensure reproducibility.<br>• All **required input data**, **metadata**, and **pre-computed results** have been made available via Zenodo (https://zenodo.org/uploads/17275776).<br>• A new **"How to install and use scDenorm"** section added in the GitHub README, with installation instructions, a quick-start example, and a link to full documentation.<br>• *scDenorm* has been registered in **bio.tools (biotoolsID: scdenorm)** and **SciCrunch.org (RRID: SCR_027574)**, and the corresponding identifiers have been included in the

If you are able to fully address these points, we would encourage you to submit a revised manuscript to GigaScience. Once you have made the necessary corrections, please submit online at:<br><br>https://www.editorialmanager.com/giga/<br><br>If you have forgotten your username or password please use the "Send Login Details" link to get your login information. For security reasons, your password will be reset.<br><br>Please include a point-by-point within the 'Response to Reviewers' box in the submission system. Please ensure you describe additional experiments that were carried out and include a detailed rebuttal of any criticisms or requested revisions that you disagreed with. Please also ensure that your revised manuscript conforms to the journal style, which can be found in the Instructions for Authors on the journal homepage. If the data and code has been modified in the revision process please be sure to update the public versions of this too.<br><br>The due date for submitting the revised version of your article is 05 Nov 2025.<br><br>I look forward to receiving your revised manuscript soon.	manuscript.<br><br>We believe these revisions significantly improve the manuscript and its reproducibility. Please find all these improvements below in our point-to-point answers to the reviewers' comments.

Reviewer 2	
**1.0** Gigascience review for Huang et al, scDenorm: a denormalisation tool for integrating single-cell transcriptomics data<br>The authors aim to address the important and often irritating problem of how to combine scRNAseq data from different sources and provided after processing with different normalization tools. The authors note that often times raw counts matrices are not provided, nor information on how the data was normalized. Their tools aim to estimate the parameters used for data transformation (eg. scale factor, pseudocount addition, log-transformation), and reverse those transformations to return to the raw counts matrix again.<br>In an ideal world where public data is well-documented and actually fully available, this type of reverse-engineering would not be necessary, but due to the current state of many data repositories, in many cases, the tools the authors provide would be indeed very useful. The paper does a good job at identifying the problem, implementing a systematic solution with useful benchmarks, and showing multiple examples of the method's utility using public datasets. However, a few additional questions arise about their method:	Thank you for your recognition of the tool we have developed. Following are the answers for reviewers' comments and suggestions one by one.
**1.1** Figure 1c-d: UMAP is a distortion/projection onto a 2D space, so it is not super surprising that different scaling factors produce clouds of cells in different locations. Do the differences in scaling factors affect the identification of the cells in any way? I.e. After denormalization for	Thank you for the comment. As illustrated in Figures 5–7, differences in normalization methods among datasets can lead to artificial inflation or suppression of marker gene expression. This, in turn, affects the accuracy of cell type identification. For example, in Figure 5b, the confusion matrix heatmap shows that a substantial proportion of

datasets of different scale factors/logbases/pseudocounts, when performing renormalization on the newly obtained raw counts, is there an optimal set of normalization parameters to now use?	CD14⁺ monocytes were misclassified as HSPCs or pDCs, which we attribute to inconsistencies in normalization across datasets.<br><br>To avoid such bias, we recommend starting from raw counts and applying a consistent normalization method across datasets. This minimizes technical biases and ensures that biological signals are preserved, thereby improving the reliability of downstream analyses such as cell type annotation and differential expression.
**1.2** Figure 2: For a particular dataset, if it is not known how the data was normalized and the transformation did not follow the form of log(C/T + P), will the tool alert the user to this? Or will the tool still attempt to find the best fit log base and pseudocount? In other words, prior to performing the denormalization, it might be helpful to display some metrics that indicate that the delta method was the most likely normalization method used, especially when ones tries to apply the tool to hundreds of datasets. (However, it seems it may not be possible to tell whether deNorm is successful until it is attempted (both empirically trying common log-base/pseudocount combinations, or solving the optimization equation), and then one assesses whether the resulting data is negative binomially distributed.)	Thank you for the suggestion. scDenorm is designed to automatically infer the most likely normalization parameters—specifically the log base and pseudocount—under the assumption that the transformation follows the form log(C/T + P). If the data conforms to this model and the resulting denormalized values follow a negative binomial distribution, the tool will report the best-fit parameters used.<br><br>However, if the data does not match the expected transformation form or fails to follow a negative binomial distribution, it will notify the user that denormalization was unsuccessful and return the original input data. This ensures transparency and prevents misleading downstream analysis.

**1.3** Figure 3d does not seem sufficiently described in the text. The authors write: "For recovery error, the absolute values are below 10-6 in 27 datasets normalized with natural logarithmic transformation (Fig. 3d, Supplementary Table 1), indicating a good accuracy of scDenorm (Fig. 3f)." Fig 3f does show recovery error, and Fig 3e shows rounding error, but what does Fig 3d intend to show? Text clarification would be helpful.	Thank you for pointing this out. Figure 3d presents the distribution of success rates across the 27 datasets that were normalized using the natural logarithmic transformation.<br><br>As defined in Equation (11) method section (see P13L495), the success rate is calculated as the proportion of cells that were successfully denormalized:<br>success rate = Nsuccess/Ntotal (11)<br><br>Nsuccess is the number of cells for which scDenorm was able to recover valid raw counts, and Ntotal is the total number of cells in the dataset. In some cases, denormalization may fail for a subset of cells due to poor sequencing quality or low gene counts. Figure 3d visualizes this variability across datasets.<br><br>We now revise the main text to explain the purpose of Figure 3d as below:<br><br>P5L184<br>To further assess the robustness of scDenorm across diverse datasets, we evaluated its performance on 27 datasets that were normalised using a natural logarithmic transformation. We present the distribution of success rates (Fig. 3d), defined as the proportion of cells that were successfully denormalised (**Equation 11**). This metric accounts for cases where poor sequencing quality or a low number of expressed genes may result in cell-wise deviations from the expected negative binomial distribution, thereby preventing accurate recovery during denormalisation. The results demonstrate that scDenorm performs robustly across diverse datasets, even when some cells cannot be fully recovered.

	P13L495<br>In certain cases, not all cells can be successfully denormalised due to poor sequencing quality, or a low number of expressed genes. To evaluate denormalisation in such situations, we define success rate as the percentage of successfully denormalised cells, equation (11).<br><br>$$\text{success rate} = \text{Nsuccess}/\text{Ntotal} \quad (11)$$<br><br>Nsuccess is the number of successfully denormalised cells, while Ntotal is the total number of cells.
**1.4** For Fig 3d, why do certain datasets have lower "success rate"? It would be helpful for the bars in Fig 3d to be in the same order as the blue bars in Fig 3b, to better compare any relationship between gene or cell number with "success rate".	Thanks for this insightful comment. Certain datasets exhibit lower success rates in Figure 3d due to technical issues such as poor sequencing quality or a low number of expressed genes (filtered datasets), which may cause individual cells to deviate from the expected negative binomial distribution. Since scDenorm relies on this distributional assumption to accurately recover raw counts, such deviations can hinder successful denormalisation.<br><br>We have a supplementary figure to match the order between Figure 4d and Figure 3b to better compare any relationship between gene or cell number with "success rate". Please see the **Supplementary Figure 7** as below.

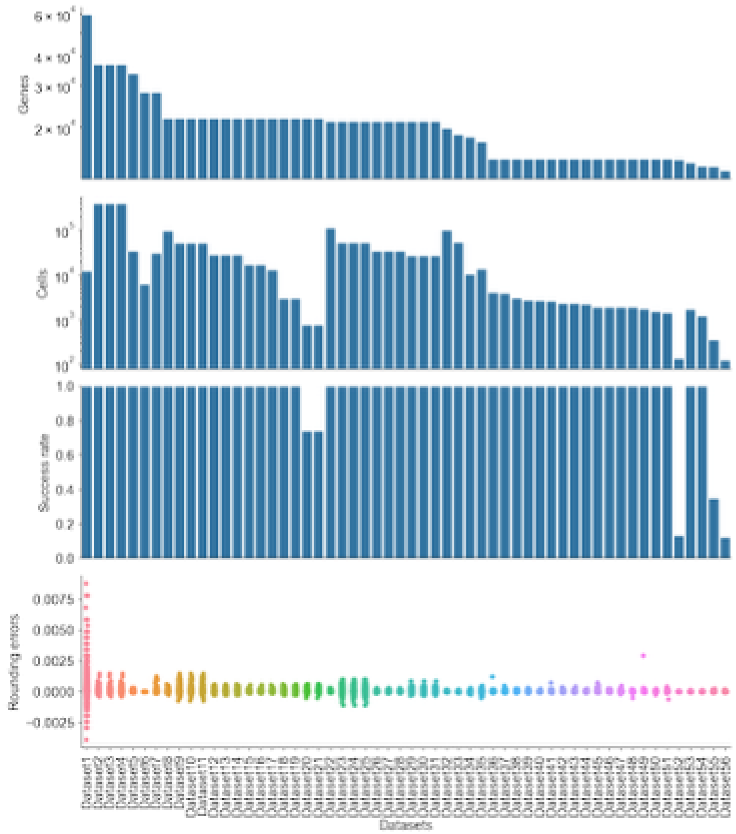

Supplementary Fig. 7 Benchmark analysis on the datasets from Brain Cell Atlas

a, The bar plot shows the number of genes in each dataset. The x-axis is the name of datasets (same as d), and the y-axis is the log-scaled number of genes.

b, The bar plot shows the number of cells in each dataset. The x-axis is the name of datasets (same as d), and the y-axis is the log-scaled number of cells.

	c, The bar plot shows the success rate for each dataset. The x-axis is the name of datasets (same as d), and the y-axis is the success rate (see Method).<br>d, The jitter plot shows the distribution of rounding errors observed in the denormalised datasets from the Brain Cell Atlas. The x-axis is the name of datasets, and the y-axis is the rounding error.
**1.5** In addition, from Methods it appears that the determination of "Success Rate" relies on knowing the original raw count values. For those data sets where many of the cells could not be successfully denormalized to raw values, what is the impact of that on the downstream cell identification or DEG analysis?	Sorry for the confusion. The calculation of success rate does not require access to the original raw count values. Instead, we evaluate whether a cell has been successfully denormalised based on the rounding error, defined as the difference between the denormalised value and its nearest integer. If the denormalised values are very close to integers (default: the rounding error<0.01), this indicates a successful recovery of count-like data.<br><br>Cells with large rounding errors are considered unsuccessfully denormalised and are filtered out prior to downstream analysis. As these cells typically correspond to low-quality profiles, their exclusion does not affect downstream tasks such as cell type identification or differential expression analysis. This filtering step ensures that only reliably recovered data are used for biological interpretation.
**1.6** Figure 4f: Not sure if this is an error in image display, but I do not see any blue points (original data) in the UMAP, as described in the legend.	Thanks. The absence of visible blue points (original data) in Figure 4f is due to the fact that they are largely overlapped by the orange points, as both sets of coordinates are nearly identical.<br><br>To clarify this, we have included a figure showing the UMAP plots for the three conditions separately as below:

<table>
<tr><td></td><td>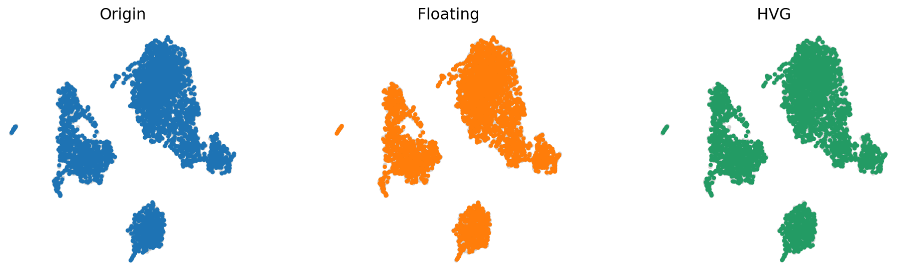

Revision Fig 1. Separate UMAP plots of different conditions (Origin, Floating, and HVG)</td></tr>
<tr><td>1.7 Figures 5-6: The authors mention assessing how scDenorm helps in different scenarios where one might want to combine data: "the batch in the COVID-19 PBMCs dataset are samples from two patients, while in the human prefrontal cortex (PFC)31,35 dataset are samples from two different studies. And the batch in the human skin36 dataset are groups of samples from young and old donors."
The most interesting scenario here are the samples from two different studies, perhaps performed by different labs, with different bioinformatic pipelines, mapped to different versions of the genome, etc. Thus, I thought the example of integrating human PFC from two different studies and how the cell type identification changed (via the river plots) was the most compelling example, and the authors could consider making that a main figure instead.</td><td>Thanks for the thoughtful feedback. We have promoted the human prefrontal cortex example to a main figure (Figure 7) in the revised manuscript. The river plots clearly demonstrate how scDenorm improves cell type identification by reducing misclassification and enhancing consistency across batches. We believe this example effectively illustrates the utility of scDenorm in real-world multi-study integration tasks.</td></tr>
<tr><td>1.8 The linked github repository, while commendable in providing code to reproduce figures, could benefit from further documentation/README showing the user how to</td><td>Thanks. We have updated the README file to include a dedicated section titled “How to install and use scDenorm” as below. This section provides clear instructions on installation, a quick-start</td></tr>
</table>

use the tool.	example for applying scDenorm to a dataset, and a link to the full documentation for further details.<br><br>**How to use and install scDenorm**<br>- Install: pip install scDenorm<br>- Usage: scdenorm data/pbmc3k_norm.h5ad --fout data/pbmc3k_denorm.h5ad<br>- Documentation: https://changebio.github.io/scDenorm
Reviewer 3	
Reproducibility report for: scDenorm: a denormalisation tool for integrating single-cell transcriptomics data<br><br>Journal: GigaScience<br><br>ID number/DOI: GIGA-D-25-00209<br><br>Reviewer(s): Laura Caquelin, Department of Clinical Neuroscience, Karolinska Institutet, Sweden<br><br>--<br><br>1. Summary of the study<br><br>In order to reduce biaises caused by different normalization methods, the authors developed scDenorm, "an algorithm that reverts normalised single-cell omics data to raw counts". They evaluated its performance on large-scale datasets to assess its impact on data integration and downstream analysis.	We sincerely thank the reviewer for the detailed and thoughtful reproducibility assessment. We greatly appreciate the effort to test *scDenorm* and have carefully addressed all points raised to improve the reproducibility, usability, and transparency of our study.<br><br>1. **Reproducibility of Figure 5f and GO Analysis**<br>Sorry for the confusion. We have reorganized and updated the code in Fig5.ipynb and R_goanalysis.ipynb (now renamed to Fig5_R_goanalysis.ipynb) to ensure clarity and consistency.<br><br>A reproducible docker environment closely matching our local setup has been constructed. We re-ran the analysis and obtained results consistent with those reported in the manuscript.<br><br>To facilitate validation, we have provided pre-computed GO enrichment results and intermediate files at Zenodo

-- <br>2. Scope of reproducibility<br><br>According to our assessment the primary objective is: to evaluate whether scDenorm improves the biological relevance of gene expression analyses by comparing GO term enrichment from differentially expressed genes (DEGs), before and after denormalization against a gold standard.<br><br>Note: While this is not the main outcome highlighted in the article's conclusion, we chose to focus on the GO enrichment analysis because its involves statistical inference abd provides a clear and reproducible downstream result.<br><br>- Outcome: GO enrichment profiles based on hematopoietic stem and progenitor cells for each condition (gold standard, before scDenorm and after scDenorm).<br><br>- Analysis method outcome: "Downstream analysis after cell type annotation includes differential expression (DE) analysis and Gene Ontology (GO) pathway analysis. Differential gene expression analysis was conducted for each cell type (one against the rest) using the Wilcoxon test implemented in SCANPY19. As part of the dataset is used as the reference dataset, the differentially expressed genes (DEGs) derived from the reference dataset is used as 'gold standard' in our evaluation. The same approach was used for the calculation of the DEGs	(https://zenodo.org/uploads/17275776).<br><br>2. **Docker Image for Reproducibility**<br>To further simplify setup and ensure reproducibility, we have provided a **ready-to-use Docker image** that includes all dependencies and environment configurations. This allows users to reproduce results without manual installation.<br>3. **Improved Documentation and Environment Setup**<br>We have added a comprehensive `environment.yaml` file for the Python environment and an `installed_packages.csv` file for the R environment, specifying exact versions of Python, R, and all required packages.<br><br>The GitHub README now includes a **tutorial for running the notebooks**, with step-by-step instructions as below:<br><br>○ Clone the repository:<br><br>`git clone https://github.com/rnacentre/scDenorm_reproducibility.git`<br><br>○ Download the Docker image and data from Zenodo: https://zenodo.org/uploads/17275776.<br>○ Move all downloaded data to `scDenorm_reproducibility/data folder.`<br>○ Run the Docker image (How to install docker: https://docs.docker.com/engine/install/):<br><br>`tar -xzf scdenorm_v0.tar.gz`

before and after scDenorm. The top differentially expressed genes were compared across different thresholds (top 50, 100, 200, 500, and 1000). As for GO pathway analysis, the enrichGO program was used on the top 500 differential expression genes.<br><br>DE and GO analysis with the correct labels from Solé-Boldo et al. was conducted for each cell type (one against the rest) using the Wilcoxon test implemented in Seurat (V3.1.1), same as the version described in Solé-Boldo et al. These analyses were conducted before scDenorm and after scDenorm. The differentially expressed genes were obtained from the study's supplementary materials as 'gold standard'". (Method<br><br>part, Dataset processing for data integration and downstream analysis section, page 14-<br><br>15)<br><br>- Main result: "Gene enrichment analysis of the differential genes in HSPCs indicated<br><br>that the GO terms enriched after scDenorm closely aligned with those of the 'gold standard', whereas the enrichment before scDenorm showed minimal overlap (Supplementary Fig. 10f). The enriched GO terms are relevant functions associated with HSPC cells, such as hematopoietic stem cell proliferation and hematopoietic progenitor cell differentiation (Fig. 5f)." (Results part, page 7)<br><br>---	`docker load -i scdenorm_v0.tar`<br><br>`docker run -p 8888:8888 -v /path/to/scDenorm_reproducibility:/app scdenorm_v0 jupyter lab --ip=0.0.0.0 --no-browser --allow-root`<br><br>4. **Clarification on Model Randomness**<br><br>We have added annotation in the notebooks which parts of the workflow may vary due to model randomness (e.g., SCCAF training) and provided pre-computed results for reproducibility.<br><br>5. **Availability of Data Files and Pre-computed Results for Comparison**<br>All previously missing data files required to reproduce the figures have been added to Zenodo. We have also included pre-computed outputs and intermediate files to support reproducibility and allow users to compare their results with those reported in the manuscript.<br><br>We believe these revisions fully address the reviewer's concerns and significantly improve the reproducibility and transparency of *scDenorm*. Thank you again for your valuable feedback and suggestion.

------------<br><br>3. AvailabilityofMaterials a. Data<br><br>- Data availability: Open<br><br>- Data completeness: Some data necessary to reproduce the main results were initially missing but were provided by the authors after we requested them.<br><br>- Access Method: Repository<br><br>- Repository: https://doi.org/10.5281/zenodo.11240443<br><br>-Data quality: The data files have been shared and appear sufficient for running the analyses. However, no metadata is provided to describe the content, structure, or origin of the files which limits interpretability and reusability.<br><br>b. Code<br><br>- Code availability: Open<br><br>- Programming Language(s): Python (Jupyter Notebook)<br><br>- Repository link: https://github.com/rnacentre/scDenorm-<br><br>reproducibility<br><br>- License: Apache 2.0<br><br>- Repository status: Public<br><br>- Documentation: The documentation could be improved. Important<br><br>details about the computational environment are missing,	

which makes it difficult to set up and reproduce the analysis. Additionally, since the code scripts are long and complex, navigating and understanding the workflow is challenging without clearer explanations.<br><br>--<br><br>4. Computational environment of reproduction analysis<br><br>- Operating system for reproduction: MacOS 15.5<br><br>- Programming Language(s): Python, R<br><br>- Code implementation approach: Using shared code with the jupyter notebook<br><br>- Version environment for reproduction: Creation of an environment with Python 3.10 and R 4.5.0<br><br>--<br><br>5. Results<br><br>5.1 Original study results - Results 1: Figure 5f<br><br>5.2 Steps for reproduction	

-> Installation and environment setup<br><br>- Issue 1: Incompatibility between SCCAF and Python.<br><br>-- Resolved: SCCAF, when installed via PyPI, required scanpy==1.4.6, which is incompatible with Python 3.12, causing a TypeError due to metaclass conflicts. A new conda environment was created with Python 3.10. The authors specified that the PyPI version of SCCAF is outdated and recommended using their GitHub repository (https://github.com/rnacentre/sccaf).<br><br>- Issue 2: Incompatibility with recent versions of pandas.<br><br>-- Resolved: An ImportError occurred due to using pandas==2.2.3.<br><br>Downgrading to pandas==1.5.3 solved the issue.<br><br>- Issue 3: Incompatibility with recent versions of anndata.<br><br>-- Resolved: Using anndata==0.11.4 raised import errors. Downgraded to anndata==0.8.0, which resolved the issue.<br><br>Setting up a clean environment with Python 3.10, scanpy==1.9.1, matplotlib==3.5.3, pandas==1.5.3, anndata==0.8.0, and the GitHub SCCAF package resolved the issue. Authors validated the environment configuration above.	

-> Running the original notebook (fig5.ipynb)<br><br>- Issue 4: KeyError due to missing labels ('Neutrophil', 'RBC') in confusion matrix.<br><br>-- Half resolved: Modified indexing logic to handle missing labels. The authors explained this variability stems from model randomness trained by SCCAF. Authors suggested filtering the label list accordingly:<br><br>conf_mat = conf_mat.loc[idx, [i for i in idx if i in conf_mat.columns]]<br><br>The proposed fix was to filter the columns with [i for i in idx if i in conf_mat.columns], but this still raises the same error due to missing labels in the rows.<br><br>conf_mat = conf_mat.loc[[i for i in idx if i in conf_mat.index] , [i for i in idx if i in conf_mat.columns]]<br><br>This error recurs throughout the code, requiring numerous modifications to be made.<br><br><br>- Issue 5: KeyError when building Sankey diagram ""Bad flow - couldn't find destination node: ('CD4+ T cell ', 'CD8+ T cell', 148)"<br><br>-- Resolved: Authors proposed instead of hardcoding the label list :<br><br>for i in new_ad.obs.predict0.value_counts()[['B cell', 'CD4+ T cell', 'NK cell', 'Other T', 'CD16+ Monocyte',	

'cDC', 'pDC', 'HSPC', 'Plasmablast', 'Platelet']].items():<br><br>Simplify it to:<br><br>for i in new_ad.obs.predict0.value_counts().items():<br><br><br>- Issue 6: KeyError: 'predict0'<br><br>-- Resolved: Column predict0 did not exist. Replaced with predict and<br><br>predict_colors:<br><br>tmp = new_ad.obs.groupby(['cell', 'predict']).size()<br><br>right_cols = pd.Series(new_ad.uns['predict_colors'], index=new_ad.obs.predict.cat.categories)<br><br>Multiple predict0 en predict modifications had to be made throughout the code.<br><br><br>- Issue 7: ValueError no field of name Neutrophil<br><br>-- Unresolved: New error in this part of code:<br><br>df_bench=[]<br><br>for i in idx:<br><br>tmp=[]<br><br>for j in [50,100,200,500,1000]: tmp.append(len(set(adx0.uns['ground']['names'][i][:j]).inter	

section(adx 0.uns['wilcoxon']['names'][i][:j])))<br><br>df_bench.append(tmp)<br><br>After asking authors about this error, they precised that if the goal is to reproduce the results of scDenorm, the Sankey plot part can be skip and proceed directly to the section "After scDenorm".<br><br>-> Running the original notebook (fig5.ipynb) - "After scDenorm" section<br><br>- Issue 8: NameError: name 'ad1' is not defined<br><br>-- Resolved: Replaced ad1 with ad3, which had been defined earlier. Authors confirmed this change is correct.<br><br>- Issue 9: AttributeError: 'DataFrame' object has no attribute 'feature_name'<br><br>-- Unresolved: The line of code "ad.var_names=list(ad.var.feature_name)<br><br>not working. feature_name column not present in ad.var. This part was skipped.<br><br>- Issue 10: KeyError: 'wilcoxon' in plotting DEG results<br><br>-- Resolved: Clarified that 'wilcoxon' is the method, not	

the key. Correct usage:<br><br>sc.pl.rank_genes_groups(adx0, n_genes=25, sharey=False, key="ground")<br><br>- Issue 11: Missing data files<br><br>-- Resolved: The following files are required but not found in the repository: * PBMC_before_scDenorm_DEGs.pkl<br><br>* PBMC_after_scDenorm_DEGs.pkl<br><br>* PBMC_go_analysis_result.csv<br><br>After request, the authors provided the script R_goanalysis.ipynb, which reproduces the PBMC_go_analysis_result.csv file, along with the necessary data files.<br><br>-> Running the R_goanalysis.ipynb and the "DEG and GO venn" section<br><br>5.3 Statistical comparison Original vs Reproduced results<br>- Results:<br><br>- Comments: As the results we obtained were completely different from those reported in the manuscript, and identical before and after scDenorm, I contacted the authors to request details about the computational R environment to run the code R_goanalysis.ipynb. Although we used the environment they provided, this did	

not resolve the discrepancy.<br><br>- Errors detected: -<br><br>- Statistical Consistency: We obtained results that were completely different from those reported in the original study. Moreover, the outputs for the "after scDenorm" and "before scDenorm" conditions appeared graphically identical.<br><br>---<br><br>6. Conclusion<br><br>- Summary of the computational reproducibility review<br><br>The reproduction of the results in the scope of this computational reproducibility review was not successful. Many errors were encountered when running the original fig5.ipynb notebook, including missing or outdated variables, missing files, and misalignments in hard-coded labels. While most of these issues were resolved through direct communication with the authors and code modifications, some steps remained partially or completely irreproducible.<br><br>The results we obtained for the figure 5f were entirely different from those reported in the original manuscript. Despite using the R script (R_goanalysis.ipynb) and data files shared by the authors, and running them in the specified environment, the GO analysis results were	

inconsistent with those presented in the publication. Furthermore, the "after scDenorm" and "before scDenorm" conditions produced graphically identical results. This raises questions about whether the differences come from the code, the data, or something else in the analysis.<br><br>- Recommendations for authors<br><br>To make the study easier to reproduce and more transparent, we suggest the following:<br><br>-- Add a requirement file that lists all the needed packages with their exact versions and mention which Python and R versions are supported. Make it clear in the documentation that the SCCAF version on PyPI is outdated and point users to the up-to-date version on GitHub.<br><br>-- Make sure all data files needed to reproduce the figures are available in the repository.<br><br>-- Check that the analysis code does not depend on fixed labels or assumptions that might not work with other datasets or slightly different results.<br><br>-- Clearly explain which parts of the results may vary due to randomness in the model and how much variation users should expect.<br><br>-- Consider adding some pre-computed results or	

intermediate files so users can compare their outputs with the original study more easily.	
