# Supplementary material for: scDenorm: a denormalization tool for integrating single-cell transcriptomics data
Source: Gigascience. 2026 Mar 31;15:giag032. doi: 10.1093/gigascience/giag032 (PMC13142155; doi:10.1093/gigascience/giag032)
Supplement: giag032_GIGA-D-25-00209_Revision_2 [file giag032_giga-d-25-00209_revision_2.pdf]

GigaScience

scDenorm: a denormalisation tool for integrating single-cell transcriptomics data
--Manuscript Draft--

**Manuscript Number:**	GIGA-D-25-00209R2
**Full Title:**	scDenorm: a denormalisation tool for integrating single-cell transcriptomics data
**Article Type:**	Research
**Funding Information:**	the Major Project of Guangzhou National Laboratory (GZNL2024A01002) — Prof. Zhichao Miao<br>the Major Project of Guangzhou National Laboratory (GZNL2023A01006) — Prof. Zhichao Miao<br>the National Key R&D Programs of China (2023YFF1204700) — Prof. Zhichao Miao<br>the National Key R&D Programs of China (2024YFF1206600) — Prof. Zhichao Miao<br>the Natural Science Foundation of China (32270707) — Prof. Zhichao Miao<br>the R&D Programs of Guangzhou National Laboratory (HWYQ23-003) — Prof. Zhichao Miao<br>the R&D Programs of Guangzhou National Laboratory (YW-YFYJ0102) — Prof. Zhichao Miao<br>Postdoctoral Research Project Funding of Guangzhou (BSHF23-049) — Dr Yin Huang
**Abstract:**	Integrating single-cell omics data at an atlas scale enhances our understanding of cell types and disease mechanisms. However, the integration of data processed by different normalisation methods can lead to biases, such as unexpected batch effects and gene expression distortion, leading to misinterpretations in downstream analysis. To address these challenges, we present scDenorm, an algorithm that reverts normalised single-cell omics data to raw counts, preserving the integrity of the original measurements and ensuring consistent data processing during integration. We evaluated scDenorm's performance on large-scale datasets and benchmarked its impact on data integration and downstream analysis across three datasets.
**Corresponding Author:**	Zhichao Miao<br>Guangzhou Laboratory<br>Guangzhou, Guangdong CHINA
**Corresponding Author Secondary Information:**	
**Corresponding Author's Institution:**	Guangzhou Laboratory
**Corresponding Author's Secondary Institution:**	
**First Author:**	Zhichao Miao
**First Author Secondary Information:**	
**Order of Authors:**	Zhichao Miao<br>Yin Huang<br>Anna Vathrakokili Pournara<br>Ying Ao<br>Ziliang Huang<br>Hui Zhang

Powered by Editorial Manager® and ProduXion Manager® from Aries Systems Corporation

	Yongjian Zhang
	Sheng Liu
	Alvis Brazma
	Irene Papatheodorou
	Xinlu Yang
	Ming Shi
**Order of Authors Secondary Information:**	
**Response to Reviewers:**	Dear Dongni,<br><br>Thanks very much for the positive feedback! We have now carefully checked the reviewers' comments. I think the only one left is comment 5 from reviewer #3, which is about the reproducibility and automation of our code. We would like to completely solve this problem in two ways: 1. We have now provided a Docker to include all the necessary environment required by our program; 2. We have now provided a README with details on running our programs. We have now run this workflow on our computers several times. If the reviewer still has a problem running the program, we would be happy to Zoom chat to solve the problem. We greatly appreciate the reviewer's check of the program, as we know the robustness and user-friendliness of a program affect its popularity. We are providing a point-to-point answer to all these comments in attached rebuttal file.<br><br>We look forward to hearing from you. Thank you in advance for sharing our enthusiasm!<br><br>Sincerely,<br><br>Zhichao (Chichau) Miao<br>Guangzhou Laboratory
**Additional Information:**	
**Question**	**Response**
Are you submitting this manuscript to a special series or article collection?	No
**Experimental design and statistics**<br><br>Full details of the experimental design and statistical methods used should be given in the Methods section, as detailed in our Minimum Standards Reporting Checklist. Information essential to interpreting the data presented should be made available in the figure legends.<br><br>Have you included all the information requested in your manuscript?	Yes
**Resources**<br><br>A description of all resources used, including antibodies, cell lines, animals	Yes

and software tools, with enough information to allow them to be uniquely identified, should be included in the Methods section. Authors are strongly encouraged to cite Research Resource Identifiers (RRIDs) for antibodies, model organisms and tools, where possible.<br><br>Have you included the information requested as detailed in our Minimum Standards Reporting Checklist?	
**Availability of data and materials**<br><br>All datasets and code on which the conclusions of the paper rely must be either included in your submission or deposited in publicly available repositories (where available and ethically appropriate), referencing such data using a unique identifier in the references and in the "Availability of Data and Materials" section of your manuscript.<br><br>Have you have met the above requirement as detailed in our Minimum Standards Reporting Checklist?	Yes
GigaScience has policies and guidelines in place for the use of generative AI-writing tools such as ChatGPT. If you have used such writing tools to assist with writing the manuscript this must be declared and cited in the text. Authors should not list AI-writing tools and other AI-assisted technologies as an author or co-author and should acknowledge that they are fully responsible for text generated or refined by AI-writing tools.<p><br><br>A summary of use (particularly in the introduction or among methods) needs to be included at the end of the paper, and the outputs should also be included as a supplementary file hosted in GigaDB or other open repositories. Please read our guidelines for more information. <p><br><br>By submitting to GigaScience, you are	No

aware of the journal's AI-writing tools policy, and if you have declared use of such tools below, you have acknowledged this where appropriate in your manuscript and have made a summary of use and outputs available. <p> <b>AI-assisted writing tools have been used in the preparation of this manuscript?	

Powered by Editorial Manager® and ProduXion Manager® from Aries Systems Corporation

Manuscript
Click here to access/download;Manuscript;scDenorm_R10320.docx

scDenorm: a denormalisation tool for integrating single-cell transcriptomics data

Yin Huang[1,2], Anna Vathrakokili Pournara[4], Ying Ao[3], Ziliang Huang[2], Hui Zhang[5], Yongjian Zhang[6], Sheng Liu[7,8], Alvis Brazma[4], Irene Papatheodorou[4], Xinlu Yang[5,*], Ming Shi[9,*], Zhichao Miao[2,3,*]

[1] Translational Research Institute of Brain and Brain-Like Intelligence and Department of Anesthesiology, Shanghai Fourth People's Hospital Affiliated to Tongji University School of Medicine, Shanghai, China
[2]Guangzhou National Laboratory, Guangzhou International Bio Island, Guangzhou 510005, China
[3] GMU-GIBH Joint School of Life Sciences, Guangzhou Medical University, Guangzhou, China
[4] European Molecular Biology Laboratory, European Bioinformatics Institute, EMBL-EBI, Wellcome Genome Campus, Cambridge CB10 1SD, UK
[5] Department of Obstetrics and Gynaecology, Harbin Red Cross Central Hospital, Harbin 150001, China.
[6] Department of Surgery Oncology, Harbin Medical University Cancer Hospital, Harbin 150001, China
[7] State Key Laboratory of Ophthalmology, Zhongshan Ophthalmic Center, Sun Yat-sen University, Guangdong Provincial Key Laboratory of Ophthalmology and Visual Science, Guangzhou, China
[8] Guangdong Province Key Laboratory of Brain Function and Disease, Guangzhou, China
[9] School of Life Science and Technology, Harbin Institute of Technology, Harbin 150001, China
* correspondence should be addressed to Zhichao Miao, miao_zhichao@gzlab.ac.cn, Ming Shi, shiming@hit.edu.cn, and Xinlu Yang, 519950370@qq.com

Yin Huang [0000-0003-1055-2602]; Anna Vathrakokili Pournara [0000-0002-2701-1987]; Ying Ao; Lirong Yang; Hui Zhang; Yongjian Zhang; Sheng Liu [0000-0002-2497-8666]; Alvis Brazma; Irene Papatheodorou [0000-0001-7270-5470]; Xinlu Yang [0009-0003-7111-3969]; Ming Shi[0000-0003-0527-0061]; Zhichao Miao [0000-0002-5777-9815]

Abstract

Integrating single-cell omics data at an atlas scale enhances our understanding of cell types and disease mechanisms. However, the integration of data processed by different normalisation methods can lead to biases, such as unexpected batch effects and gene expression distortion, leading to misinterpretations in downstream analysis. To address these challenges, we present scDenorm, an algorithm that reverts delta-method normalised single-cell omics data to raw counts, preserving the integrity of the original measurements and ensuring consistent data processing during integration. We evaluated scDenorm's performance on large-scale datasets and benchmarked its impact on data integration and downstream analysis across three datasets.

Background

Single-cell RNA sequencing (scRNA-seq) is a powerful high-throughput technology for measuring gene expression in individual cells. Integration of atlas-level single-cell transcriptomics data has exerted a great potential for understanding how cells orchestrate in the human body, as well as complex molecular mechanisms in various diseases[1,2]. With the progress of the Human Cell Atlas (HCA)[3], an increasing number of reference atlases are available for comparison and integration[4–6]. Numerous integration methods have been developed and several studies have been performed to benchmark their performance and explore their limitations[7–9]. In order to achieve effective large-scale data integration, it is crucial to take into account the assumptions of data distribution and noise levels. For instance, scVI[10] and scANVI[11] integration methods model single-cell data using a Negative Binomial distribution (also known as Gamma-Poisson distribution), and thus both require raw counts as input. Even though some other integration methods, *e.g.,* Seurat integration methods[12] (RPCA, CCA), Harmony[13], Liger[14] etc, do not directly rely on raw counts, they inherently make assumptions about the data distribution. As a result, for most existing integration methods, it is key to ensure the consistency of the input datasets.

To address technical variations (*e.g.,* sequencing depth) and biases inherent in scRNA-seq, scaling and transformation methods are often employed to ensure comparability across cells[15,16]. Normally, scaling is used to account for sequencing depth, while transformation is used to stabilise variance of the data. The differences between variance-stabilising transformations have been benchmarked by Constantin Ahlmann-Eltze and Wolfgang Huber[17], demonstrating the effectiveness of the delta method for comparing cells with varying gene expression levels. In a delta normalisation, raw counts are scaled by total counts and target sum, followed by log-transformation with an added pseudo count (see **Methods**). It has been adopted in well-established analysis workflows (e.g., Seurat[18] and SCANPY[19]), assuming that droplet-based scRNA-seq data follows a negative binomial distribution[20–23]. In some large-scale data resources, such as the UCSC Cell Browser[24], delta method normalised matrices are deposited instead of the raw counts to facilitate reproducibility of analysis results. Thus, many datasets are available only as processed matrices rather than raw counts, hindering atlas-level data integration.

The best way to guarantee consistent data processing in large-scale data integration is to use raw counts as input. If we integrate normalised data with raw counts, processed data can be renormalised again while the raw counts are being normalised, thus introducing unnecessary biases. Some downstream analysis steps[25–27] (such as multinomial model based highly variable gene selection[28], differential gene expression analysis by statistical modeling of read counts[29]) also assume raw counts as input. When raw counts are not available, researchers often seek to obtain the raw sequencing data and re-analyse them, including secondary analysis of reads mapping, demultiplexing, and quantification analysis[30] to obtain the raw count matrix. However, a count matrix from the re-analysis may deviate from the original published analysis in terms of reference genome and cell barcodes. Thus, the cell type annotation or other metadata reported in the raw publication cannot be used, rendering difficulties in reproducing the analysis results. Besides, this secondary analysis can be both computationally expensive and time-

consuming. Therefore, reliable conversion of normalised matrices back to raw counts can benefit large-scale data integration tasks as well as wider use of publicly deposited data. Yet, there is no tool available to meet this urgent need.

In this study, we propose scDenorm, an algorithm that converts delta method normalised gene expression data back to the raw counts. It effectively explores key implicit features of the data distribution in scRNA-seq and recovers raw count matrices. Based on benchmarking across large-scale datasets, as well as application studies of downstream analysis, we demonstrate the capability, accuracy, scalability and efficiency of this method. Moreover, scDenorm can deal with different normalisation parameters, thereby facilitating data integration, consistent downstream analyses and the construction of atlases.

Results

Inconsistent data normalisation may generate biases in data integration

Using the 10x 3k peripheral blood mononuclear cells (PBMCs) data, which is the example data used in the well-established SCANPY[19] and Seurat[18] single-cell tutorials, as an example, we investigated the impact of normalisation parameters in the delta method. These parameters are the scaling factor, logarithmic transformation base, and pseudo counts. Matrices normalised by different parameters go through the same downstream analysis of highly variable gene selection, dimensionality reduction (e.g., Principal Component Analysis), clustering and visualisation. The Uniform Manifold Approximation and Projection (UMAP) plot shows deviations between datasets processed with different normalisation parameters, e.g., the deviations between B cells in $L=10^3$ and the same B cells in other normalisations, indicating the potential bias introduced by inconsistent data normalisation (**Fig. 1a,b**, **Supplementary Fig. 1a**). Furthermore, such a data normalisation effect cannot be removed through data integration by Harmony[13], scanorama[31], or BBKNN[32] (**Fig. 1**, **Supplementary Fig. 1**), e.g., the B cell populations in **Fig. 1c** and **1d** cluster separately. Therefore, we suggest converting the normalised matrices back to raw counts for a consistent data integration and downstream analysis.

The denormalisation process in scDenorm

We term the recovery of normalised data to raw counts as “denormalisation”. Denormalising delta method normalised data requires the determination of three parameters, scaling factors, the logarithmic transformation (log-transformation) base, and the pseudo count. The first step for denormalisation is to determine if log-transformed has been applied for the whole expression matrix. It is well established that droplet-based scRNA-seq data follows a negative binomial distribution[20–23], where the variance exceeds the mean (**Fig. 1e**). Thus, the variance versus mean distribution effectively indicates whether the data has been log-transformed or not (**Supplementary Fig. 2a**). The second key step in denormalisation is to determine the scaling factor for each cell. This needs to exploit the implicit data distribution feature of scRNA-seq.

Droplet-based scRNA-seq mainly probes the highly expressed genes, rendering a high dropout rate. In a sparse matrix where zeros have been removed, the frequency of counts can be ranked, with the most frequent count number being one, followed by two, and so on (**Fig. 1f**, **Supplementary Fig. 2b,c**). Using such a 'count-rank' distribution, scaling factors for cells can be measured by establishing the relationship between the top two most frequent numbers in the normalised data and numbers one and two. After exploring 105 datasets from the Brain Cell Atlas[33], we found that over 99% of the cells follow this 'count-rank' distribution for the top three most frequent count numbers (one, two, and three), while >95% cells in Chromium and > 80% cells in Drop-seq follow the distribution for the top five count numbers. Notably, >99% cells in Smart-seq2 data follow this distribution for the top ten numbers (**Fig. 1g**, **Supplementary Fig. 2d**). Following this 'count-rank' distribution, the top most frequent count numbers can be used to determine the three parameters in delta method normalisation **(Supplementary Fig. 3a**).

The denormalisation procedure in scDenorm involves two steps: de-transformation and unscaling (**Supplementary Fig. 3b**). In the de-transformation step, a subset matrix (100 cells) is used to determine the same log-transformation base and pseudo count among cells since these two parameters keep the same for the whole expression matrix. Using a subset of data effectively accelerates the calculation. First, empirical values (*e.g.,* 2, e Euler's number, 10 for the log-transformation base, 0.01, 0.1, 1 for pseudo count), which are used in standard analysis workflows, are tried. If not successful, these two parameters can be determined by solving equations between the top two most frequent numbers (**Supplementary Fig. 3c**). In the unscaling step, each cell has a different scaling factor, which is a ratio between the total counts of the cell and the target sum (e.g., 10,000). To measure the scaling factor of a cell, we implement two methods: 1) a regression-based method (see **Methods**, equation (4) in **Supplementary Fig. 3d**) and 2) solving equations between the top two most frequent numbers (see **Methods**, equation (5) in **Supplementary Fig. 3d**), while the latter method offers the advantages of fast speed and good robustness (**Supplementary Fig. 4a,b**). As the expression matrix is processed from raw counts, which consists of integers only, a successful denormalisation should result in a small mean square error between denormalised values and their nearest integers (see **Methods**).

To elaborate the denormalisation process, we used an example dataset[34] of single-nucleus RNA sequencing (snRNA-seq) data of autism spectrum disorder, with both the normalised data and raw count matrix available in the autism database (see **Data availability**). According to the respective publication[34], the data was normalised with the delta method. The relationship between the top ten most frequent gene expression values and their respective frequencies in three cells in the processed data (Fig. 2a), suggests a logarithmic distribution, while the less frequent values after them do not follow such a distribution due to dropouts. Should any of the cells in the dataset follow the same distribution. And the mean versus variance distribution confirms a logarithmic conversion (Fig. 2b). The log-transformation base and the pseudo count are determined as 2 and 1 respectively, by solving equation (3) in Supplementary Fig. 3c. These parameters show a good fit according to the top two most frequent values (Fig. 2c). The normalised matrix is de-transformed by taking the exponential of the log-transformation base and subtracting the pseudo count, resulting in a 'scaled matrix'. In the scaled matrix, the top five most frequent values show a linear 'count-rank' distribution in each cell (Fig. 2d). The slope of

the line is the reciprocal of the scaling factor. This linear distribution indicates the success of de-transformation. Additionally, the mean versus variance distribution (Fig. 2e) confirms this success. The summed expression values for most cells are approximately 10,000, indicating that the target sum is 10,000. Some genes may have been removed after normalisation, leading to a reduction in the summed expression values (Fig. 2f). In the unscaling step, scaling factors are determined by solving equation (5) in Supplementary Fig. 3d. Each cell is multiplied by its scaling factor resulting in a "denormalised matrix", which is supposed to be similar to the raw count matrix of integers. As in a sparse matrix, the top two most frequent numbers should be one and two (Fig. 2g). The mean versus variance distribution of the denormalised matrix conforms to a negative binomial distribution (Fig. 2h), which is expected for the raw counts of droplet-based scRNA-seq. Comparing the denormalised matrix with the raw count matrix, the maximum error for each value was less than 0.001 (Fig. 2i), which may result from the digital float calculation. After taking round values, the denormalised matrix is identical to the raw count matrix, suggesting a successful denormalisation.

scDenorm recovers raw count matrices for large-scale database

To evaluate the performance of scDenorm in realistic scenarios, 40 processed datasets (**Supplementary Table 1**) from the UCSC Cell Browser[24] were used as test data, covering a good variety of species, tissues and sequencing techniques (**Fig. 3b**). Denormalisation performance was evaluated by two metrics: 1) rounding error, defined as the difference between a value in the denormalised matrix and its nearest integer (round value), and 2) recovery error, defined as the difference between a value in the normalised matrix and its corresponding value in the denormalised matrix after renormalisation (**Fig. 3a**, See **Methods**). 32 out of the 40 test sets were successfully denormalised (**Fig. 3c**, **Supplementary Table 1**), while the 8 unsuccessful cases were normalised as TPM (Transcripts per Million), log2(FRPM) or by scTransform[35] method rather than the delta method (**Supplementary Table 1**). The mean versus variance distribution confirms a negative binomial distribution after denormalisation, indicating the successful denormalisation (**Supplementary Fig. 5**). To further assess the robustness of scDenorm across diverse datasets, we evaluated its performance on 27 datasets that were normalised using a natural logarithmic transformation. We present the distribution of success rates (**Fig. 3d**), defined as the proportion of cells that were successfully denormalised (**Equation 11**). This metric accounts for cases where poor sequencing quality or a low number of expressed genes may result in cell-wise deviations from the expected negative binomial distribution, thereby preventing accurate recovery during denormalisation. The results demonstrate that scDenorm performs robustly across diverse datasets, even when some cells cannot be fully recovered. The rounding errors, which are positively correlated to the expression value (**Supplementary Fig. 6**), are consistently below 0.005 (**Fig. 3e**). For recovery error, the absolute values are below 10^{-6} in 27 datasets normalised with natural logarithmic transformation (**Supplementary Table 1**), indicating a good accuracy of scDenorm (**Fig. 3f**). Further benchmarking of the denormalisation on 60 datasets (**Supplementary Table 2**) from the Brain Cell Atlas[33] show similar results (**Supplementary Fig. 7**). In addition, scDenorm shows a linear computational time complexity and memory usage with increasing number of cells and genes, demonstrating a high computational efficiency and scalability (**Supplementary Fig. 4c,d**).

scDenorm accurately recovers raw counts in different scenarios

In realistic scenarios, denormalising the normalised matrix deposited in the database can be affected by several aspects, including: 1) the parameters used in delta normalisation method; 2) the digital precision kept in the deposited data; and 3) the genes filtered after data normalisation (**Fig. 4a**), (e.g., some lowly expressed genes could be removed). Using the 10x 3k PBMC single-cell dataset as a showcase, we benchmark these aspects.

We examined the effect of normalisation parameters (target sum, log-transformation base, and pseudo count) by simulating the normalisation process with eight sets of hierarchical parameters. The dataset was normalised using these parameters, and denormalised by scDenorm. As shown in **Fig. 4b**, the errors between the denormalised value and its raw count in all denormalised matrices are consistently low, as < $5x10^{-4}$, indicating a minimal impact from normalisation parameters.

The digital precision of the normalised data, which can vary depending on the data processing tools and the saved file format, can also affect computational memory consumption. By default, the normalised data is saved as float32 (single-precision floating-point) format, with a precision of 6 to 9 decimal digits[36]. We simulated data with lower precision and denormalised it with scDenorm. The recovery error was less than 0.5 for count values less than 100, and less than one for count values greater than 100 (**Fig. 4c**). The errors are less than 0.01 when the digit precisions are more than 4 digits. The precision achieved with 3 to 4 decimal digits was consistent with the results of float16 conversion (**Fig. 4d**). Yet two decimal precision shows larger errors in highly expressing genes but keeps the cell identities (**Supplementary Fig. 8**).

In scRNA-seq data analysis, some genes expressed in few cells need to be removed or only selected genes may be kept in the normalised matrix. We simulated a gradient of the number of selected genes and tested the impact on denormalisation. No detectable error was found when more than 300 genes are kept in the normalised matrix, with the error increasing as the number of genes decreases (**Fig. 4e, Supplementary Fig. 9**). However, downstream data visualisation demonstrates that the denormalised matrices from float16 precision and a selection of 2000 highly variable genes successfully recovered the UMAP representation derived from raw counts (**Fig. 4f,g**), in spite of minor differences in the values.

scDenorm facilitates downstream analysis

We further evaluated the impact of denormalisation on downstream analysis tasks, including data integration, cell type annotation, differential expression (DE) analysis, and gene ontology (GO) analysis. As data from different batches may go through different normalisation, three datasets were prepared to cover different batch types. The batch in the COVID-19 PBMCs[37] dataset are samples from two patients, while in the human prefrontal cortex (PFC)[34,38] dataset are samples from two different studies. And the batch in the human skin[39] dataset are groups of samples from young and old donors.

The two patient samples in the COVID-19 PBMCs[37] dataset are first normalised with different target sums (1000 and 10,000) before going through downstream analysis (**Supplementary Fig. 10**). Firstly, if without denormalisation, the UMAP visualisation after harmony integration shows cells of the same cell type exist in multiple clusters, e.g., plasmablast (**Fig. 5a**). Subsequently, SCCAF[40], a well-established reference-based machine learning algorithm, was used to annotate the cell types. The first sample was used as a reference to annotate the cell types in the second sample, resulting in an accuracy of 66% (the consistency between the original cell type labels and those assigned by SCCAF). Notably, CD14+ monocytes were misclassified as plasmacytoid dendritic cells (pDCs) and hematopoietic stem and progenitor cells (HSPCs), while CD8+ T cells were misclassified as natural killer (NK) cells and CD4+ T cells (**Fig. 5b**). Fortunately, with the help of scDenorm denormalisation, the two patient samples can be integrated, with each cell type cluster forming distinct clusters (**Fig. 5c**). And the accuracy of cell type annotation using SCCAF increased to 92%, indicating effective correction of denormalisation. Furthermore, mis-annotated cell type labels may result in biases in differential gene expression analysis (**Fig. 5d, Supplementary Fig. 10d**) and gene ontology analysis (**Supplementary Fig. 10e**). For instance, 350 differential genes in HSPCs matched the 'gold standard' after scDenorm, compared to only 81 before scDenorm (**Fig. 5e**). Gene enrichment analysis of the differential genes in HSPCs indicated that the GO terms enriched after scDenorm closely aligned with those of the 'gold standard', whereas the enrichment before scDenorm showed minimal overlap (**Supplementary Fig. 10f**). The enriched GO terms are relevant functions associated with HSPC cells, such as hematopoietic stem cell proliferation and hematopoietic progenitor cell differentiation (**Fig. 5f**).

Similarly, the same analysis of data integration and cell type annotation was performed on two other datasets, the human prefrontal cortex dataset and the human skin dataset. Both study-wise batch (the former dataset) and condition-wise batch (the latter dataset) demonstrated that data processed by scDenorm yielded superior integration results (**Fig. 6, Fig. 7**) and improved annotation results from SCCAF (**Fig. 7g,h, Supplementary Fig. 11d,e**). Yet, the mislabelled cell types lead to biased differentially expressed genes (DEGs) (**Fig. 7i**) and Gene Ontology (GO) terms (**Fig. 7j**).

Furthermore, we evaluate the impact of normalisation parameters on downstream DE and GO analysis. Taking the human skin data[39] as an example, differential gene expression analysis was performed before and after scDenorm using the cell type labels derived from the original publication, while the published differential expression genes (see **Methods**) and their resulted GO results were taken as 'gold standard'. The DE and GO results after scDenorm show higher consistency with the 'gold standard' than the results before scDenorm (**Fig. 6g,h**). In addition, the DEGs identified before scDenorm include more false positive genes (**Fig. 6i**), resulting in the enrichment of unrelated GO terms (**Fig. 6j**), such as the nuclear transport function for keratinocyte cells (**Supplementary Fig. 11f**).

Discussion

In our survey of 133 well-established single-cell studies, delta method normalisation takes up >83% (110) of the datasets (**Supplementary Table 3**), since it is implemented in widely-used SCANPY

and Seurat analysis workflows. We demonstrate the capability of scDenorm on an example dataset and large-scale test sets from UCSC database[24] and the Brain Cell Atlas[33]. Different parameter sets in the delta method normalisation as well as the digital precision kept in the normalised data have minimal effect on denormalisation. Moreover, the number of genes kept after normalisation does not significantly affect denormalisation, unless the number of genes used is too small (less than 300) (**Fig. 4e**). In the 40 datasets from UCSC database (**Supplementary Table 1**) and 60 datasets from Brain Cell Atlas (**Supplementary Table 2**), scDenorm successfully restored count values in the majority (88%) of the cases, with minimal rounding errors and recovery errors. Therefore, scDenorm may robustly recover matrices for the majority (estimated to be 80-90%) of the datasets, which are delta method normalised, while maintaining efficient computational speed (**Supplementary Fig. 4c**).

The limitations of scDenorm relies on specific prerequisites of the delta method normalisation. Datasets normalised using alternative methods may not be compatible with scDenorm. For example, GLM residual methods (such as SCTransfrom[35]) and latent expression (such as Sanity[41] and Dino[42]) cannot be denormalised by scDenorm. Fortunately, other denormalisation methods besides the delta method only constitute 10-20% of the datasets. And the raw counts of these datasets can be obtained from reads mapping. Additionally, the performance of scDenorm may be influenced by the choice of normalisation parameters and the quality of the input data. Cells whose gene expression distribution deviates from the assumptions of negative binomial distribution may lead to the failure of the denormalisation process.

Several case studies show that different normalisations can result in unnecessary deviations in downstream analysis, including data integration, cell type annotation, differential gene expression, gene ontology and pathway analysis. In particular, biased differential expression or gene ontology results can be generated due to different normalisation parameters when the cell type annotation is correct. Therefore, denormalising the expression matrix to raw counts can be a good choice to mitigate biases in downstream analysis. It could be a key question for large-scale data integration, where study-wise batch effects need to be minimised while biology should be kept. Batch correction as well as data integration methods have already been extensively discussed and benchmarked[8]. Here we highlight the consistency in data processing, which is nontrivial when data from tens or hundreds of studies need to be combined together. Consistent single-cell data analysis workflows which can both keep the raw conclusions from the publications and get integrated with data from other studies would greatly help. Therefore, the availability and reproducibility of the raw published analysis code would be important.

Conclusions

Here, we demonstrate that inconsistent data normalisation can generate unexpected bias in data integration, potentially obstructing atlas-level single-cell data integration. Fortunately, denormalising processed data back to raw counts could standardise analysis, thereby facilitating the creation of comprehensive cell atlases. We present scDenorm, a tool designed to denormalise data from the delta method normalisation, which are widely used by 80% of the 40 datasets in the UCSC database and 93% of the 60 datasets in Brain Cell Atlas. It employs both equation solving

and regression methods to determine the parameters in the delta method. Benchmarks on 32 UCSC cell browser datasets and 56 Brain Cell Atlas datasets demonstrate the efficacy of scDenorm for delta method normalisation data, with further applications on COVID-19 PBMCs, prefrontal cortex, and human skin datasets revealing its ability to mitigate biases in downstream analysis. scDenorm can be a useful tool in atlas level single-cell data processing and integration, such as the Human Cell Atlas[43], The Human Developmental Cell Atlas[44], the Brain Cell Atlas[33], and HuBMAP[45].

Methods

Assumption and algorithm design

In scRNA-seq, the data is in the form of a count matrix, where most entries are zeros due to the sparsity of gene expression. Our assumption is that the scRNA-seq data follows negative binomial distribution, which is theoretically and empirically well-supported for the unique molecular identifier data[17]. This means that probabilistically speaking, in the count matrix zero is the most frequently observed count, followed by one and two and so on. The sequential pattern of these values has a probabilistic one-to-one correspondence with the rank of their frequency by descending order (**Fig. 1f**). The smaller the values, the higher the probability of the correspondence (**Fig. 1g**). For example, without considering 0, the probability that the values 1 and 2 are equal to the rank of their frequency is almost 100%. Based on this assumption, we design an algorithm to normalise scRNA-seq data that has been normalised by the most commonly used delta methods, which scale the raw counts by the total number of counts (library size) and target sum (the summed value of the cell after scaling), and then log-transformed after adding a pseudo count (**Supplementary Fig. 3a**). Specifically, we consider a scaled expression matrix from a count matrix which has been transformed to adjust for differences in the scale of the features (e.g., genes) in the data. In scRNA-seq data, a scaled expression matrix typically refers to a count matrix that has been normalised and transformed to have a similar distribution of gene expression values across cells. For example, scRNA-seq data can be normalised to account for differences in sequencing depth and other technical factors that can affect the distribution of counts across cells and genes, such as total count normalisation. And it can be transformed to adjust for the distribution of gene expression values across cells such as log-transformation, and variance-stabilising transformation. The normalised gene expression matrix is derived from the count matrix to adjust for differences in gene expression across cells, which usually involves scaling and transformation techniques such as total count scaling and log-transformation. This normalisation process does not change the one-to-one correspondence between the gene expression value and its rank of the value's frequency in a cell.

Using the probabilistic one-to-one correspondence property, we can extract a cell vector from a normalised expression matrix and sort the values based on their frequency in the vector. This allows us to establish that the most frequently occurring non-zero value corresponds to one and the second most frequent represents two and so forth, which means the rank number and the count number are theoretically the same and this is normally true for the top ranks. By following

this procedure, we were able to obtain the rank and normalised value pairs (C, N) (where C is the rank and N is the normalised count) for the equation $N = log_b^{(\frac{C}{s}+p)}$, (s is the scaling factor, b is the base of log-transformation, and p is the pseudo count). First, we try reversing log transformation of natural base(e), base 2 and base 10, and solve the equation on the pairs of values (1, N_1) and (2, N_2), N_1 and N_2 are the values of the two most frequent numbers. Normally, we think the pseudo count C is given as 1. Otherwise, we need to check whether the variance of the solved C from different cells is sufficiently small, since each vector from the gene expression matrix has been augmented with the same pseudo count. If the unscaling process is unsuccessful for all of the above cases, we conclude that the matrix has not been pre-processed according to the workflow. The following shows the complete workflow of scDenorm algorithm.

The denormalisation algorithm can be divided into two steps, de-transformation and unscaling. In de-transformation, there are two sequential steps. Firstly, (a) we search for empirical values for the log-transformation bases and the pseudo count. It searches for empirical bases such as 2, e (natural base), and 10, as well as common pseudo counts like 0, 0.01, 0.1, and 1. If the pseudo count is 0, it indicates that the normalisation process has not added the pseudo count. A fraction of cells is used to evaluate if any of these bases or pseudo counts meet the criteria in step (c). If passing the criteria, skip to step 2. Otherwise, it goes to step (b) to determine the parameters. Step (b) uses equation solving method to determine the parameters: This method uses the two values (N_1, N_2) occurring most frequently in a cell to construct the following equation. For each cell i:

$$\frac{1}{s_i} + p = b^{N_1^i} \quad (1)$$

$$\frac{2}{s_i} + p = b^{N_2^i} \quad (2)$$

s_i is the scaling factor for the cell i. The p and b are pseudo count and base. From equation (1) and (2), we can get equation (3).

$$p = 2 \times b^{N_1^i} - b^{N_2^i} \quad (3)$$

Randomly select a group of cells (e.g., n=100) to generate a corresponding set of data points (N_1, N_2), and solve p and b by equation (4) with optimization methods.

$$argmin \sum_{i=1}^{n} \quad (p - 2 \times b^{N_1^i} + b^{N_2^i})^2 \quad (4)$$

The L-BFGS-B method from the sklearn[46] package is used to find the best base (b) and pseudo count (p). This method is based on the limited-memory Broyden-Fletcher-Goldfarb-Shanno (BFGS) algorithm, which is capable of large-scale optimization. L-BFGS-B allows for box constraints, ensuring that the parameters stay within specified bounds during optimization.

After the de-transformation, the sum of each cell should be the same or very similar. Step (c). checks if the sum of each cell is the same. For example, let X as the vector of the sums, x is a number in it. And if abs(x-mean(X)) is always smaller than the small number (*e.g.*, mean(X)=10000, x=9999.7, small number is 0.5), then the de-transformation is successful. However, this is an ideal situation. Often, we encounter that after normalisation, the data filters out some genes for the quality control in downstream analysis. In addition, some normalisation methods do not scale the total expression values to the same for all cells. To address these

complex cases, we also added the following criteria. If it is the automatic detection method, we only need to make sure that the mean square error (MSE) is small enough, such as 10^{-5}. In general, we just need to unscale a cell to see if it is successful.

In unscaling, we have two approaches implemented in the same function, while a parameter can be used to select the option. The first approach (a) is based on regression to determine the scaling factors for all cells. The scaling factor is derived from fitting a regression model to the relationship between the de-transformed values and their ranks, providing an estimate of the scaling factor for each cell. For each cell:

$$\arg\min_{s} \sum_{i=1}^{n} \left(\frac{c_i}{s} + p - x_i\right)^2 \quad (5)$$

c_i is the rank, and x_i is the de-transformed value.
To ensure a more accurate one-to-one correspondence, only the first 5 pairs of values (c_i, x_i) are used. We can get the scaling factor s by optimising the equation (5) using the same L-BFGS-B method as in solving equation (4).

The second approach (b) is solving equations between the top two most frequent values: This method only used the first 2 pairs of values (c_i, x_i). We can get a closed form of solution by solving the following equation. For each cell:

$$\frac{c_1}{s} + p = x_1 \quad (6)$$

$$\frac{c_2}{s} + p = x_2 \quad (7)$$

From equation (6) and (7), we can get equation (8).

$$s = \frac{c_2 - c_1}{x_2 - x_1} \quad (8)$$

To evaluate the success of the denormalisation process, we quantify the error between the denormalised values and their rounded counterparts. Ideally, denormalised values should closely approximate integers. We therefore compute the mean absolute error (MAE) between the denormalised matrix and its rounded count matrix, and assess whether the MAE falls below a predefined threshold (default cutoff: 0.05). If the MAE exceeds this cutoff, the denormalisation is considered unsuccessful. Of note, in some cases, the same top value (e.g., 1) can be normalised into more than 1 different values due to some improper data processing, and the ranks of these numbers are thus lower than expected. These numbers with tiny differences are merged as one value by decreasing their digital precision.

scDenorm is publicly available as an open-source Python package and provides a user-friendly python function interface, which can be combined in the use of SCANPY analysis. It can be used both at the command line and interactively in Jupyter notebook. Description of the function details are provided in **Supplementary Materials**. Considering that different samples in a dataset may be normalised with different parameter sets, scDenorm also implements a per sample denormalisation function overloading the original 'scdenorm' function with a 'by=sample' parameter as input.

Integration of scRNA-seq data from different normalisation parameters

We downloaded a PBMCs scRNA-seq data from the 10x genomics datasets, and preprocessed and annotated the data according to the pbmc3k Scanpy tutorial. Then, we used different parameter combinations (including 1e3, 1e4, 1e5, 1e6 as target sums, 2, e, 10 as base, and 1,0.1, 0.001 as pseudo counts) to normalise the data separately and merge all the data together. Principal Component Analysis (PCA) of 50 components was derived from the expression matrix. Three single-cell data integration tools (Harmony, BBKNN, scanorama) were tested to integrate the combined data with the normalisation parameters as the batch key. For data visualisation, Uniform Manifold Approximation and Projection (UMAP)[47] is calculated in the integrated latent space or the PCA space.

Consistency of count-rank relationship across sequencing platforms

The consistency of count-rank relationship refers to the percentage of the correct one-to-one correspondence between the gene expression value and its rank of the value's frequency in a cell. For example, given 100 cells, we first calculate the frequency of the raw count values (the raw count value called as count) in each cell, and order the frequencies from highest to lowest. The order is called as rank, which ranges from 1,2, …, n. If count is the same as rank, we consider this to be a correct one-to-one correspondence. Finally, for the count from 1 to 10, we respectively calculate what percentage of cells have the correct one-to-one correspondence as the consistency of the count-rank relationship. To compare different sequencing platforms, we calculated the consistency of the count-rank relationship in 105 datasets obtained from the Brain Cell Atlas. Among these datasets, 81 are from Chromium, 15 from Drop-seq, and 9 from Smart-seq2.

Evaluation metrics

When benchmarking denormalisation for scRNA-seq data, two measures can be used: rounding error and recovery error. Rounding error measures the discrepancy between the denormalised values and their rounded counterparts. After denormalisation, the expected outcome is that the denormalised values approximate integers. Rounding error quantifies the extent to which the denormalised values deviate from integers. To calculate rounding error, the difference between each denormalised value and its rounded value is computed, see equation (9) below. Recovery error evaluates the difference before denormalisation and after re-normalising the denormalised values (values after scDenorm, **Fig. 3a**). To calculate recovery error, the difference between each normalised value and its re-normalised value is computed, see equation (10) below.

Specifically, we assume x is the normalised value (a single value for one gene in one cell), y is the denormalised value after scDenorm, and z is the re-normalised value from denormalised value

(y). The rounding error is calculated as the difference between the denormalised value (y) and its rounded value, equation (9):

$$rounding\_error = round(y) - y \quad (9)$$

The recovery error is calculated as the difference between the normalised value (x) and the re-normalised value (z), equation (10):

$$recovery\_error = x - z \quad (10)$$

In certain cases, not all cells can be successfully denormalised due to poor sequencing quality, or a low number of expressed genes. To evaluate denormalisation in such situations, we define success rate as the percentage of successfully denormalised cells, equation (11).

$$success\ rate = Nsuccess/Ntotal \quad (11)$$

Nsuccess is the number of successfully denormalised cells, while Ntotal is the total number of cells.

Benchmark scDenorm based on digital precision and gene filtering

To assess the impact of different digital precision of normalised data on the denormalisation process, we performed the following steps on the PBMC data: First, we applied total-count normalisation (the normalize_total function in SCANPY[19]) to the data matrix with target sum as 10,000, and log-transform (natural base, e) the data with 1 as pseudo count. Next, we used the round function to retain the data at different levels of precision, ranging from 2 to 8. Float16 corresponds to 3 to 4 decimal places of precision, while float32 corresponds to 6 to 9 decimal places of precision. Finally, we denormalise the data separately for each precision level and compare the results with rounding errors to evaluate their effects.

To test our algorithm for gene filtering on extreme cases, we selected a series of highly variable genes, ranging from 100, 200, 300, 400, 500, 1000, 2000 to 5000. Specifically, First, we normalise the data by sc.pp.normalise_total with target_sum as 10,000 and logarithmic the data with sc.pp.log1p. The high-variable genes are then selected using sc.pp.highly_variable_genes with layer as ‘count’ and flavour as ‘seruat_v3’. Finally, we use scDenorm to denormalise the data and calculate the recovery errors.

Benchmark on large-scale datasets

To perform the usage of our tool on atlas data, we downloaded 40 datasets from the UCSC Cell Browser and 60 datasets from the Brain Cell Atlas, ensuring that they encompass a diverse range of species, sequencing platforms, and normalisation methods. First, we used scDenorm to denormalise each dataset. If successful, we calculate the rounding errors for the dataset, which quantifies the difference between the denormalised values before and after rounding. In addition,

when the total expression values (i.e., the sum of all denormalised values within each cell) were close to a fixed target sum (e.g., 10,000) after de-transformation, we further calculated the recovery error. Specifically, the datasets were re-normalised using a target sum of 1e4 , a pseudo-count of 1, and the natural logarithm base (e). The recovery error was calculated as the difference between the original normalised matrix and the re-normalised matrix obtained after denormalisation and re-normalisation.

Dataset processing for data integration and downstream analysis

The COVID-19 PBMC dataset from Arunachalam et al.[37] (**Fig. 5**) was downloaded from GEO[48] under the accession code GSE155673. Two samples Arunachalam_cov11 (S1) and Arunachalam_cov11 (S2) were processed with different delta normalisation parameters: S1 was normalised by target sum 1e3, while S2 was normalised by target sum 1e4. And both were log-transformed. For data visualisation, we performed Harmony[13] data integration of these two samples after PCA of 50 components. For cell type annotation, SCCAF[40] was used. S2 was used as the reference for annotating S1.

The human skin dataset from Solé-Boldo et al., was downloaded from GEO under the accession code GSE130973 (**Fig. 6**), including two young (25 and 27 years old) and three old (53, 69 and 70 years old) donors. The young samples were normalised to target sum 1e3, while the old samples were normalised to target sum 1e4. And both samples were logarithmic transformed after normalisation. For cell type annotation, the old samples were used as the reference for annotating the young sample.

The human prefrontal cortex data includes datasets from two studies, Ma et al.[38] (170,000 cells) and Velmeshev et al.[34] (100, 000 cells) (**Fig. 7**). The Velmeshev's dataset was normalised to target sum 1e3 and logarithmic transformation, while the Ma's dataset was not normalised. Harmony was used for data integration. For cell type annotation, the Ma's dataset was used as the reference for annotating Velmeshev's dataset.

For data processing after denormalisation with scDenorm, we follow a standard workflow of data normalisation and dimension reduction. Specifically, the expression matrix is normalised to target sum of 10,000 and log-transformed. And the default dimension reduction process in the SCANPY workflow was used, including PCA, Harmony integration and UMAP visualisation. SCCAF was used to predict the cell types as described above.

Downstream analysis after cell type annotation includes differential expression (DE) analysis and Gene Ontology (GO) pathway analysis. Differential gene expression analysis was conducted for each cell type (one against the rest) using the Wilcoxon test implemented in SCANPY[19]. As part of the dataset is used as the reference dataset, the differentially expressed genes (DEGs) derived from the reference dataset is used as 'gold standard' in our evaluation. The same approach was used for the calculation of the DEGs before and after scDenorm. The top differentially expressed genes were compared across different thresholds (top 50, 100, 200, 500,

and 1000). As for GO pathway analysis, the enrichGO program was used on the top 500 differential expression genes.

DE and GO analysis with the correct labels from Solé-Boldo et al. was conducted for each cell type (one against the rest) using the Wilcoxon test implemented in Seurat (V3.1.1), same as the version described in Solé-Boldo et al. These analyses were conducted before scDenorm and after scDenorm. The differentially expressed genes were obtained from the study’s supplementary materials as 'gold standard'.

Data availability

The 10x 3k PBMC data was downloaded from the 10x genomics website [49]. The dataset with both the normalised expression and the raw count matrix was downloaded from the UCSC Cell Browser autism dataset [50]. 40 processed datasets (**Supplementary Table 1**) were downloaded from the UCSC Cell Browser [51]. 60 processed datasets (**Supplementary Table 2**) were downloaded from the Brain Cell Atlas [52]. Datasets used in the manuscript have been deposited at Zenodo [53].

Availability of source code and requirements

Project name: scDenorm
Project homepage: https://github.com/rnacentre/scDenorm
License: Apache-2.0 license
Operating system: Linux
Programming language: Python
Package management: pip-https://pypi.org/project/scDenorm/; anaconda-https://anaconda.org/changebio/scdenorm
Hardware requirements: No requirements
biotools: scdenorm
RRID: SCR_027574
Codes for reproducing this work: https://github.com/rnacentre/scDenorm_reproducibility

Declarations

Ethics approval and consent to participate

Not applicable.

Consent for publication

Not applicable.

Competing interests

The author declares no competing interests.

Author's contributions

Z.M. and A.B. designed and conceived the study. Y.H., and Z.M. implemented the scDenorm algorithm, Y.H. conceived and performed most of the bioinformatics analyses. Y.A. performed part of the analysis. H.Z., Y.Z., S.L., X.Y., and M.S. provided some datasets. Y.H., Z.M., A.V.P., Y.A., I.P. wrote the manuscript. Z.M., M.S., X.Y. supervise the study.

Acknowledgements

The authors thank Ziliang Huang for help with the datasets.

Funding

This work was supported by the Major Project of Guangzhou National Laboratory (grant nos GZNL2024A01002 and GZNL2023A01006), the National Key R&D Programs of China (2023YFF1204700, 2024YFF1206600), the Natural Science Foundation of China (32270707), the R&D Programs of Guangzhou National Laboratory (grant nos HWYQ23-003 and YW-YFYJ0102), and Postdoctoral Research Project Funding of Guangzhou, BSHF23-049.

References

1. Butler A, Hoffman P, Smibert P, Papalexi E, Satija R. Integrating single-cell transcriptomic data across different conditions, technologies, and species. *Nat Biotechnol*. 2018; doi: 10.1038/nbt.4096.

2. Ren X, Wen W, Fan X, Hou W, Su B, Cai P, et al.. COVID-19 immune features revealed by a large-scale single-cell transcriptome atlas. *Cell*. 2021; doi: 10.1016/j.cell.2021.01.053.

3. Regev A, Teichmann SA, Lander ES, Amit I, Benoist C, Birney E, et al.. The Human Cell Atlas. *Elife*. 2017; doi: 10.7554/eLife.27041.

4. Travaglini KJ, Nabhan AN, Penland L, Sinha R, Gillich A, Sit RV, et al.. A molecular cell atlas of the human lung from single-cell RNA sequencing. *Nature*. 2020; doi: 10.1038/s41586-020-2922-4.

5. Elmentaite R, Ross ADB, Roberts K, James KR, Ortmann D, Gomes T, et al.. Single-Cell Sequencing of Developing Human Gut Reveals Transcriptional Links to Childhood Crohn's Disease. *Dev Cell*. 2020; doi: 10.1016/j.devcel.2020.11.010.

6. Sikkema L, Ramírez-Suástegui C, Strobl DC, Gillett TE, Zappia L, Madissoon E, et al.. An integrated cell atlas of the lung in health and disease. *Nat Med*. 2023; doi: 10.1038/s41591-023-02327-2.

7. Song Y, Miao Z, Brazma A, Papatheodorou I. Benchmarking strategies for cross-species integration of single-cell RNA sequencing data. *Nat Commun*. 2023; doi: 10.1038/s41467-023-41855-w.

8. Luecken MD, Büttner M, Chaichoompu K, Danese A, Interlandi M, Mueller MF, et al.. Benchmarking atlas-level data integration in single-cell genomics. *Nat Methods*. 2022; doi: 10.1038/s41592-021-01336-8.

9. Büttner M, Miao Z, Wolf FA, Teichmann SA, Theis FJ. A test metric for assessing single-cell RNA-seq batch correction. *Nat Methods*. 2019; doi: 10.1038/s41592-018-0254-1.

10. Lopez R, Regier J, Cole MB, Jordan MI, Yosef N. Deep generative modeling for single-cell transcriptomics. *Nat Methods*. 2018; doi: 10.1038/s41592-018-0229-2.

11. Xu C, Lopez R, Mehlman E, Regier J, Jordan MI, Yosef N. Probabilistic harmonization and annotation of single-cell transcriptomics data with deep generative models. *Mol Syst Biol*. 2021; doi: 10.15252/msb.20209620.

12. Stuart T, Butler A, Hoffman P, Hafemeister C, Papalexi E, Mauck WM 3rd, et al.. Comprehensive Integration of Single-Cell Data. *Cell*. 2019; doi: 10.1016/j.cell.2019.05.031.

13. Korsunsky I, Millard N, Fan J, Slowikowski K, Zhang F, Wei K, et al.. Fast, sensitive and accurate integration of single-cell data with Harmony. *Nat Methods*. 2019; doi: 10.1038/s41592-019-0619-0.

14. Liu J, Gao C, Sodicoff J, Kozareva V, Macosko EZ, Welch JD. Jointly defining cell types from multiple single-cell datasets using LIGER. *Nat Protoc*. 2020; doi: 10.1038/s41596-020-0391-8.

15. Vallejos CA, Risso D, Scialdone A, Dudoit S, Marioni JC. Normalizing single-cell RNA sequencing data: challenges and opportunities. *Nat Methods*. 2017; doi: 10.1038/nmeth.4292.

16. Bacher R, Chu L-F, Leng N, Gasch AP, Thomson JA, Stewart RM, et al.. SCnorm: robust normalization of single-cell RNA-seq data. *Nat Methods*. 2017; doi: 10.1038/nmeth.4263.

17. Ahlmann-Eltze C, Huber W. Comparison of transformations for single-cell RNA-seq data. *Nat Methods*. 2023; doi: 10.1038/s41592-023-01814-1.

18. Satija R, Farrell JA, Gennert D, Schier AF, Regev A. Spatial reconstruction of single-cell gene expression data. *Nat Biotechnol*. 2015; doi: 10.1038/nbt.3192.

19. Wolf FA, Angerer P, Theis FJ. SCANPY: large-scale single-cell gene expression data analysis. *Genome Biol*. 2018; doi: 10.1186/s13059-017-1382-0.

20. Grün D, Kester L, van Oudenaarden A. Validation of noise models for single-cell

transcriptomics. *Nat Methods*. 2014; doi: 10.1038/nmeth.2930.

21. Cao Y, Kitanovski S, Küppers R, Hoffmann D. UMI or not UMI, that is the question for scRNA-seq zero-inflation. *Nat Biotechnol*. 2021; doi: 10.1038/s41587-020-00810-6.

22. Kharchenko PV. The triumphs and limitations of computational methods for scRNA-seq. *Nat Methods*. 2021; doi: 10.1038/s41592-021-01171-x.

23. Svensson V. Droplet scRNA-seq is not zero-inflated. *Nat Biotechnol*. 2020; doi: 10.1038/s41587-019-0379-5.

24. Speir ML, Bhaduri A, Markov NS, Moreno P, Nowakowski TJ, Papatheodorou I, et al.. UCSC Cell Browser: visualize your single-cell data. *Bioinformatics*. 2021; doi: 10.1093/bioinformatics/btab503.

25. Fahrenberger M, Esk C, Knoblich JA, von Haeseler A. GTestimate: improving relative gene expression estimation in scRNA-seq using the Good-Turing estimator. *Gigascience*. Oxford University Press (OUP); 2025; doi: 10.1093/gigascience/giaf084.

26. Hua Y, Weng L, Zhao F, Rambow F. SeuratExtend: streamlining single-cell RNA-seq analysis through an integrated and intuitive framework. *Gigascience*. 2025; doi: 10.1093/gigascience/giaf076.

27. Song W-M, Ming C, Forst CV, Zhang B. Unsupervised multiscale clustering of single-cell transcriptomes to identify hierarchical structures of cell subtypes. *Gigascience*. Oxford University Press (OUP); 2025; doi: 10.1093/gigascience/giaf111.

28. Townes FW, Hicks SC, Aryee MJ, Irizarry RA. Feature selection and dimension reduction for single-cell RNA-Seq based on a multinomial model. *Genome Biol*. 2019; doi: 10.1186/s13059-019-1861-6.

29. Chen W, Li Y, Easton J, Finkelstein D, Wu G, Chen X. UMI-count modeling and differential expression analysis for single-cell RNA sequencing. *Genome Biol*. 2018; doi: 10.1186/s13059-018-1438-9.

30. Li M, Zhang X, Ang KS, Ling J, Sethi R, Lee NYS, et al.. DISCO: a database of Deeply Integrated human Single-Cell Omics data. *Nucleic Acids Res*. 2022; doi: 10.1093/nar/gkab1020.

31. Hie B, Bryson B, Berger B. Efficient integration of heterogeneous single-cell transcriptomes using Scanorama. *Nat Biotechnol*. 2019; doi: 10.1038/s41587-019-0113-3.

32. Polański K, Young MD, Miao Z, Meyer KB, Teichmann SA, Park J-E. BBKNN: fast batch alignment of single cell transcriptomes. *Bioinformatics*. 2020; doi: 10.1093/bioinformatics/btz625.

33. Chen X, Huang Y, Huang L, Huang Z, Hao Z-Z, Xu L, et al.. A brain cell atlas integrating single-cell transcriptomes across human brain regions. *Nat Med*. 2024; doi: 10.1038/s41591-024-03150-z.

34. Velmeshev D, Schirmer L, Jung D, Haeussler M, Perez Y, Mayer S, et al.. Single-cell genomics identifies cell type-specific molecular changes in autism. *Science*. 2019; doi: 10.1126/science.aav8130.

35. Hafemeister C, Satija R. Normalization and variance stabilization of single-cell RNA-seq data using regularized negative binomial regression. *Genome Biol*. 2019; doi: 10.1186/s13059-019-1874-1.

36. Muller J-M, Brunie N, de Dinechin F, Jeannerod C-P, Joldes M, Lefèvre V, et al.. Handbook of Floating-Point Arithmetic. Springer International Publishing;

37. Arunachalam PS, Wimmers F, Mok CKP, Perera RAPM, Scott M, Hagan T, et al.. Systems biological assessment of immunity to mild versus severe COVID-19 infection in humans. *Science*. 2020; doi: 10.1126/science.abc6261.

38. Ma S, Skarica M, Li Q, Xu C, Risgaard RD, Tebbenkamp ATN, et al.. Molecular and cellular evolution of the primate dorsolateral prefrontal cortex. *Science*. 2022; doi: 10.1126/science.abo7257.

39. Solé-Boldo L, Raddatz G, Schütz S, Mallm J-P, Rippe K, Lonsdorf AS, et al.. Single-cell transcriptomes of the human skin reveal age-related loss of fibroblast priming. *Commun Biol*. 2020; doi: 10.1038/s42003-020-0922-4.

40. Miao Z, Moreno P, Huang N, Papatheodorou I, Brazma A, Teichmann SA. Putative cell type discovery from single-cell gene expression data. *Nat Methods*. Springer Science and Business Media LLC; 2020; doi: 10.1038/s41592-020-0825-9.

41. Breda J, Zavolan M, van Nimwegen E. Bayesian inference of gene expression states from single-cell RNA-seq data. *Nat Biotechnol*. 2021; doi: 10.1038/s41587-021-00875-x.

42. Brown J, Ni Z, Mohanty C, Bacher R, Kendziorski C. Normalization by distributional resampling of high throughput single-cell RNA-sequencing data. *Bioinformatics*. 2021; doi: 10.1093/bioinformatics/btab450.

43. Rozenblatt-Rosen O, Stubbington MJT, Regev A, Teichmann SA. The Human Cell Atlas: from vision to reality. *Nature*. 2017; doi: 10.1038/550451a.

44. Haniffa M, Taylor D, Linnarsson S, Aronow BJ, Bader GD, Barker RA, et al.. A roadmap for the Human Developmental Cell Atlas. *Nature*. 2021; doi: 10.1038/s41586-021-03620-1.

45. HuBMAP Consortium. The human body at cellular resolution: the NIH Human Biomolecular Atlas Program. *Nature*. 2019; doi: 10.1038/s41586-019-1629-x.

46. Pedregosa F, Varoquaux G, Gramfort A, Michel V, Thirion B, Grisel O, et al.. Scikit-learn: Machine Learning in Python. *J Mach Learn Res*. 2011; doi: 10.5555/1953048.2078195.

47. McInnes L, Healy J, Saul N, Großberger L. UMAP: Uniform Manifold Approximation and Projection. *J Open Source Softw*. The Open Journal; 2018; doi: 10.21105/joss.00861.

48. Barrett T, Wilhite SE, Ledoux P, Evangelista C, Kim IF, Tomashevsky M, et al.. NCBI GEO: archive for functional genomics data sets--update. *Nucleic Acids Res*. 2013; doi: 10.1093/nar/gks1193.

49. 10x Genomics. 10x 3k PBMC Dataset. https://www.10xgenomics.com/datasets/3-k-pbm-cs-from-a-healthy-donor-1-standard-1-1-0. Accessed 20 Feb 2026.

50. UCSC Cell Browser. Autism Cell Dataset. https://autism.cells.ucsc.edu. Accessed 20 Feb

2026.

51. UCSC Cell Browser. Processed scRNA-seq datasets. https://cells.ucsc.edu. Accessed 20 Feb 2026.

52. Brain Cell Atlas. Processed scRNA-seq datasets. https://www.braincellatlas.org. Accessed 20 Feb 2026.

53. Huang, Y. (2025). scDenorm: a denormalisation tool for Integrating Single-cell Transcriptomics Data. Zenodo. https://doi.org/10.1101/2025.05.10.653289

Figure legends

Fig. 1 The data distribution of droplet-based single-cell data

a, UMAP plot of PBMC 3k datasets, without data integration, normalised with different delta normalisation parameters, including target sum (L), logarithmic base (b) and pseudo count (p). The plot is coloured by different parameter sets.
b, The same UMAP plot as panel (**a**) coloured according to cell type annotation.
c, UMAP plot after Harmony integration of data normalised by different parameters, coloured by cell types.
d, The UMAP plots are the same as panel (**c**) (after data integration by Harmony), displaying four different normalisation parameter sets.
e, Scatter plot demonstrating the mean (x-axis) against the variance (y-axis) for each gene in the count matrix of the PBMC 3k datasets. Each dot shows the mean and variance value of a gene. The diagonal line is shown in blue. The orange curve is the fitted curve of negative binomial distribution with variance [illegible], mean μ and dispersion φ.
f, Histogram depicting the frequencies of count values and their ranks in a single cell, showing the ‘count-rank’ distribution in a cell selected from the count matrix of the PBMC 3k datasets.
g, The percentage of cells that follow the ‘count-rank’ distribution (the value of count equal to its rank from 1 to 5) in three scRNA-seq technologies (Chromium, Smart-seq2, and Drop-seq).

Fig. 2 Evaluation of scDenorm on normalised scRNA-seq data with known raw counts

a, The scatter plot shows the distribution between expression values and their ranks of frequencies in three example cells. Each dot is an expression value and its rank of frequency in the cell, different cells are shown in different shape and colour.

b, the scatter plot shows the distribution between the log-transformed mean expression (x-axis) and the log-transformed variance (y-axis) for each gene in the gene expression matrix from Velmeshev et al dataset. The diagonal line (x=y) is shown in blue.
c, The scatter plot shows the distribution between the most and second most frequent values in different cells, displaying each cell as a dot. The blue curve shows the fitted equation derived from the equation (4) in **Methods**, with base value (b) equal to 2 and pseudo count (p) equal to 1. N_1 and N_2 are the most and second most frequent values in cells.
d, The scatter plot shows the distribution between expression values and their ranks of frequencies in the three example cells after de-transformation, coloured in the same manner as panel (**a**).
e, The scatter plot shows the relationship between the mean expression (x-axis) and variance (y-axis) for each gene after de-transformation.
f, The scatter plot shows the distribution between the number of genes and the target sum (sum of all expression values) in the cell after de-transformation.
g, The scatter plot shows the distribution between expression values and their ranks of frequencies in the three example cells after unscaling, displaying the 'count-rank' distribution.
h, The dot plot shows the distribution between the mean expression (x-axis) with the variance (y-axis) for each gene in the count matrix.
i, The scatter plot shows the distribution between the count values and the errors between the denormalised matrix with the raw count matrix after denormalisation. Each dot represents a count value in a cell and its rounding error.

Fig. 3 Performance of scDenorm on normalised scRNA-seq data from UCSC Cell Browser

a, The diagram illustrates the workflow of scDenorm to evaluate denormalisation using the rounding and recovery error. The normalised matrix (left) deposited in the UCSC database is first denormalised (middle) with scDenorm to calculate the rounding error (see **Methods**). Subsequently, the denormalised matrix (middle) is re-normalised (right) to measure the recovery error (see **Methods**). These matrices are used to calculate the rounding errors (the difference between the denormalised matrix and its rounding matrix) and recovery errors (the difference between the normalised matrix and the re-normalised matrix). The values of target sum and pseudo count normalisation parameters are 1e4 and 1, respectively.
b, Two barplots show the number of genes (top) and number of cells (bottom) for the collected UCSC datasets. The x-axis shows the datasets by name, while the y-axis shows the log-scaled number of genes (top) and the log-scaled number of cells (bottom). The colours represent the different parameters of the delta normalisation. Blue and orange are natural base (e) and base=2 respectively, green represents data without log-transformation. Red shows non-delta method normalisation cases, which could not be denormalised by scDenorm.
c, The pie chart shows the distribution of the number of datasets classified by different base values, detected by scDenorm. The colours are the same as shown in panel (**b**). 'Unknown' represents unsuccessful cases that were not normalized with the delta method, while all other 32 cases were successful.
d, The bar plot shows the distribution of the success rate (see **Methods**) across the 27 datasets

that were normalised with natural logarithmic transformation.
e, The jitter plot shows the distribution of rounding errors observed in the denormalised datasets. The x-axis is the rounding error, while the y-axis shows the same datasets as panel (**d**).
f, The box plot shows the distribution of recovery errors after re-normalization with the parameters of target sum, pseudo count and logarithm base as 1e4, 1 and natural base (e), respectively. The x-axis is the recovery error, while the y-axis shows the datasets in the same order as in panel (**d**).

Fig. 4 Benchmark of scDenorm on different normalisation parameters, digital precision and gene filtering

a, The diagram shows the workflow of evaluating the recovery errors of denormalisation in three scenarios, different normalisation parameters, different digit precision, and gene filtering, on the PBMC 3k dataset. The raw count matrix (top) is first normalised with different parameter sets (middle), and then denormalised (bottom) with scDenorm giving different digit precisions and filtered genes. In delta method normalisation: C is the count value; T is the total count value of a cell; L is the target sum; p is the pseudo count; b is the base of logarithmic function; and N is the normalised gene expression values.
b, The line plot shows the distribution of recovery errors from using different parameter sets in delta normalisation.
c, The dot plot shows the distribution between raw count values and their recovery errors after the conversion of normalised data from float32 to float16.
d, The line plot shows the distribution of recovery errors from normalised float32 data while preserving different levels of digit precision (2-8 digit precisions).
e, The histogram shows the mean square error of regression loss of equation (4) (see **Methods**) from normalised data with different numbers of highly variable genes (from 100 to 5,000).
f, The UMAP plot shows the distribution of data processed using different approaches, including original processed data (blue), denormalised data after converting to float16 (orange), and denormalised data after selecting 2,000 highly variable genes (green). Both original processed data and denormalised data show similar UMAP visualisations.
g, The UMAP plot shows the cell type distribution of panel (**f**), colour-coded by cell types. Both original processed data and denormalised data capture the same cell types.

Fig. 5 Different normalisations impact cell type annotation of COVID-19 PBMCs

a, The UMAP plot shows the distribution of cells before denormalisation by scDenorm, coloured by predicted cell type annotation (see **Methods**). The two patient samples were normalised to different target sums (1,000 and 10,000). The UMAP shows that some cells of the same type exist in multiple clusters and mix with other cell types as highlighted black circle.
b, The heatmap shows the confusion matrix between the published cell type labels and the predicted cell types based on the Harmony-integrated latent space before scDenorm denormalisation. The x-axis represents predicted cell types, while the y-axis denotes original cell

type annotation published in the study. The confusion matrix was derived from SCCAF, based on logistic regression learning of the data.

c, The UMAP plot shows the distribution of cells after Harmony data integration followed by scDenorm. Cells are coloured by predicted cell type annotation. The UMAP shows that cells of the same type are clustered together.

d. The histogram shows the percentage of DEGs overlap between the ‘gold standard’ (DEGs derived according to the original published cell type labels) and the ones derived from re-analysis before (blue) and after (orange) scDenorm across cell types. The DEGs are calculated with a two-sided Wilcoxon test using the predicted cell types as clusters.

e. The Venn diagram shows the overlap of the top 500 DEGs for HSPCs derived from the 'gold standard' (blue) from original study, as well as before (orange) and after (red) scDenorm.

f. The heatmap shows the enriched Gene Ontology (GO) terms of HSPCs derived from DEGs of the 'gold standard' (blue) from original study, as well as before (orange) and after (red) scDenorm.

Fig. 6 Different normalisations impact differential expression analysis

a, The UMAP plot shows the cell distribution of the two age groups of the human skin dataset (Solé-Boldo et al.) before data integration without the scDenorm denormalisation, coloured by age groups.

b, The UMAP plot shows the Harmony-integrated result without the scDenorm denormalisation, coloured by age groups.

c, The UMAP plot shows the Harmony-integrated result after running scDenorm, coloured by age groups.

d, The UMAP plots show the same distribution as panels (**a**), coloured by original cell type labels from Solé-Boldo et al.

e-f, The UMAP plots show the same distribution as panels (**b-c**), but coloured by predicted cell type labels before and after denormalisation by scDenorm.

g-h, The histograms show the percentages of DEGs (**g**) and GO terms (**h**) overlap between the 'gold standard' (DEGs extracted from the data in the original study, GO terms derived from these DEGs) and data before (purple) and after (blue) scDenorm across cell types, using the ‘gold standard’ cell type labels reported in the original publication. The DEGs are calculated with a two-sided Wilcoxon test based on the original cell type from the human skin dataset (Solé-Boldo et al.), while the GO analysis shows Benjamini-Hochberg-adjusted P value <0.05.

i-j, The Venn diagrams show the overlaps of the DEGs (**i**) (Benjamini-Hochberg-adjusted P value <0.05 and logFC >0.25) and GO terms (**j**) for differentiated keratinocytes derived from data of the 'gold standard' (red), as well as before (purle) and after (blue) scDenorm denormalisation.

Fig. 7 scDenorm helps in cell type annotation and differential expression analysis on prefrontal cortex datasets

a, The UMAP plot shows the distribution of cells of the two prefrontal cortex datasets before

integration.

b, The UMAP plot shows Harmony-integrated results without the scDenorm denormalisation, coloured by studies.

c, The UMAP plot shows the Harmony-integrated result after running scDenorm, coloured by studies.

d, The figure legend of cell type annotation for panel (**e**) and (**f**).

e, The UMAP plot is the same as (**b**) coloured by predicted cell type annotation.

f, The UMAP plot is the same as (**c**) coloured by predicted cell type annotation.

g-h, River plot illustrates the transition between original and predicted cell types before scDenorm (**g**) and after scDenorm (**h**). The left side represents the original cell types from Velmeshev et al., while the right side displays the predicted cell types.

i, Bar plot showing the overlapping percentage of DEGs between the gold standard with before and after scDenorm across cell types. The DEGs are calculated with a two-sided Wilcoxon test based on the predicted cell types.

j, Bar plot showing the overlapping percentage of GO terms between the gold standard with before and after scDenorm across cell types.

Click here to access/download;Figure;Fig7.pdf

Figure

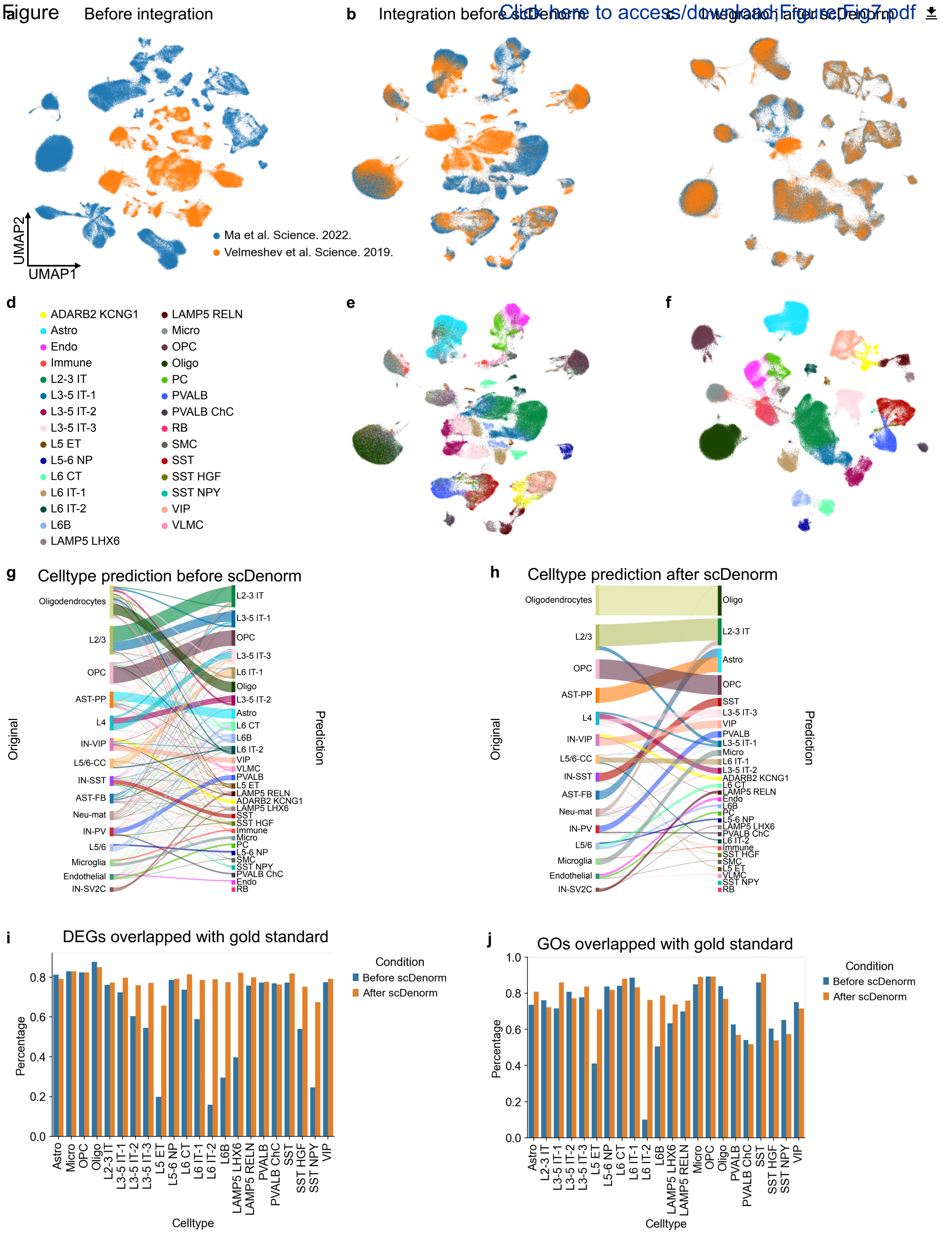

Fig1

Click here to access/download;Figure;Fig1.pdf

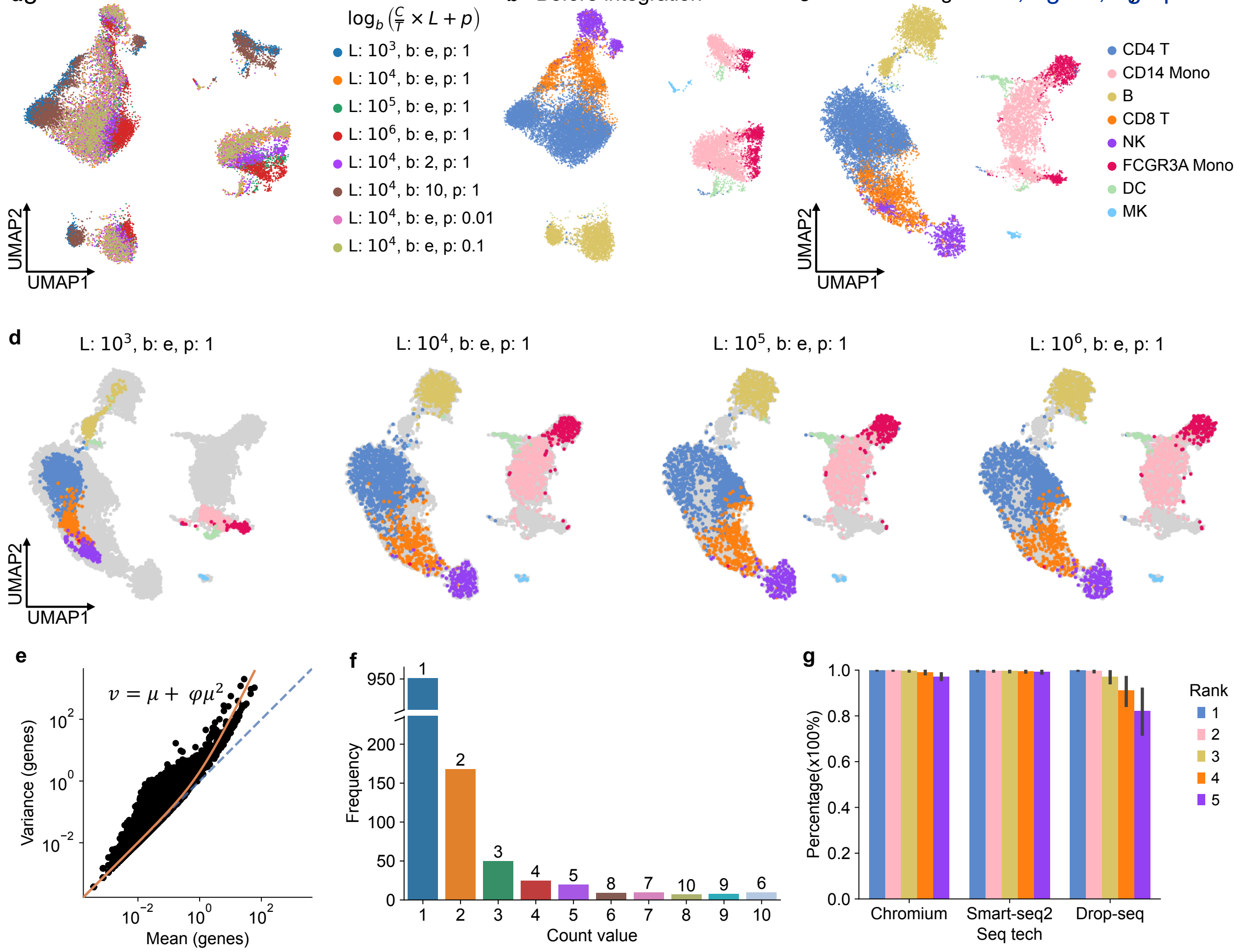

Fig2

Click here to access/download;Figure;Fig2.pdf

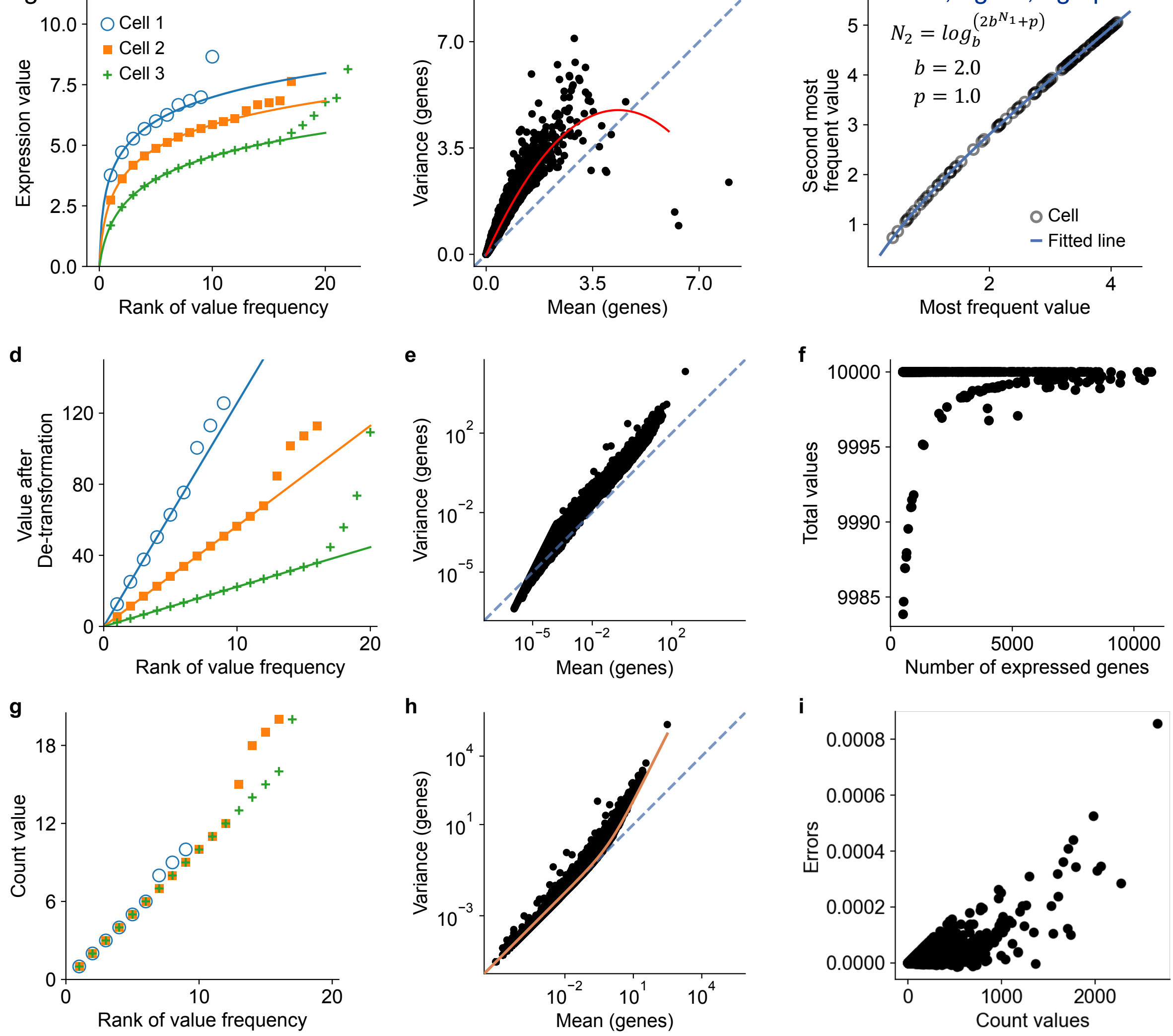

Fig3

Click here to access/download;Figure;Fig3.pdf

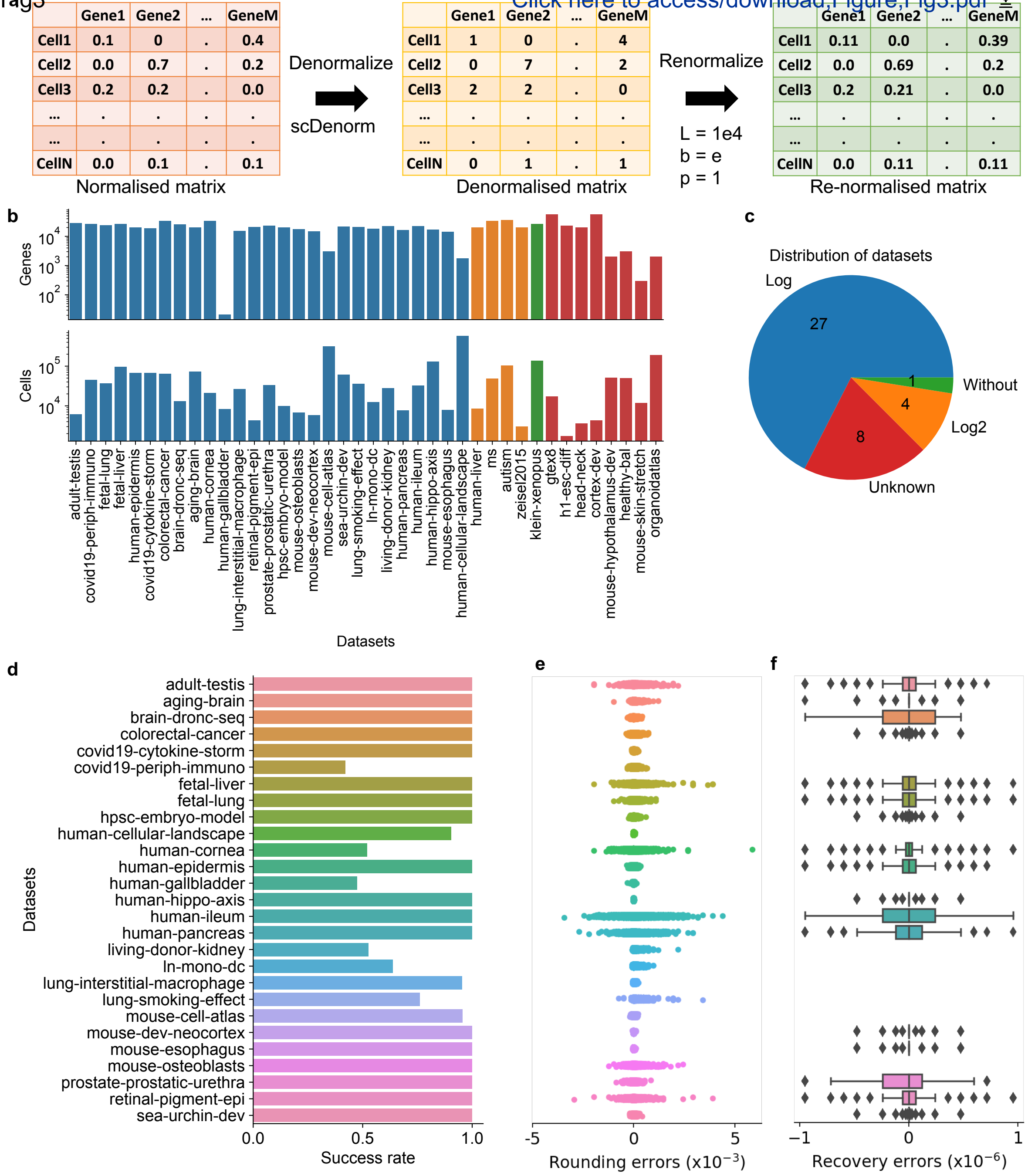

Fig4

Click here to access/download;Figure;Fig4.jpg

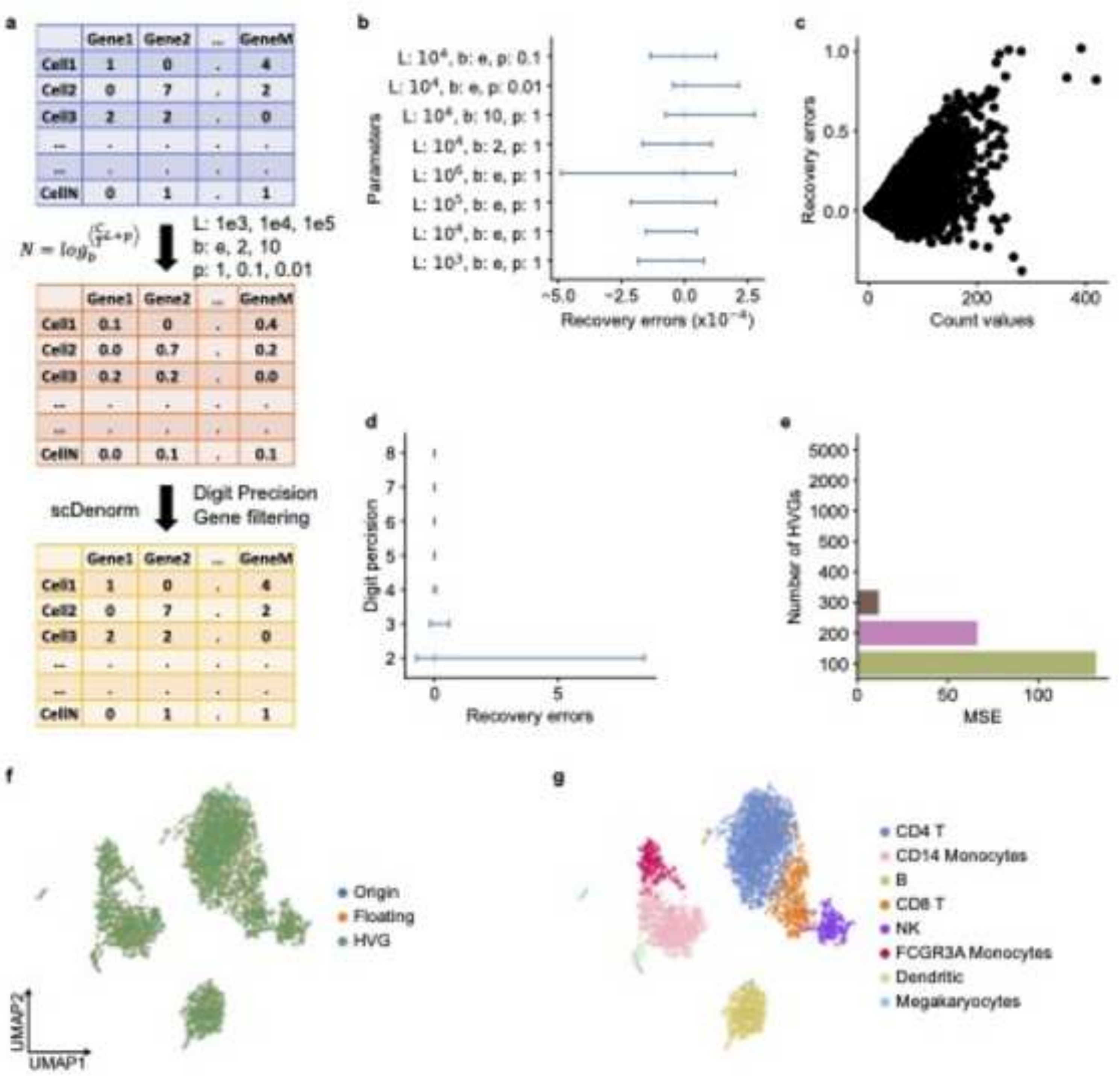

Click here to access/download;Figure;Fig4.pdf

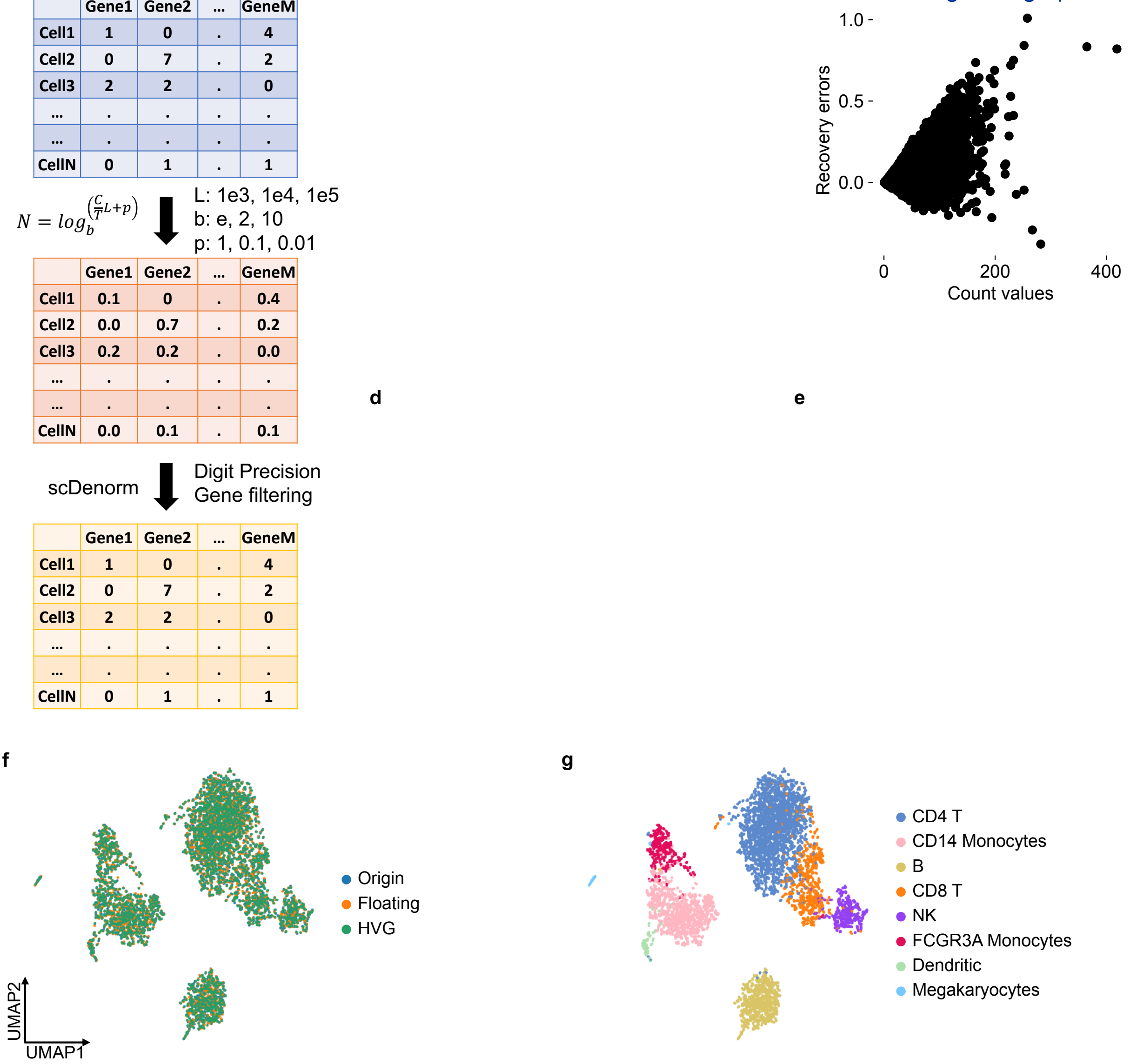

Fig5

Click here to access/download;Figure;Fig5.pdf

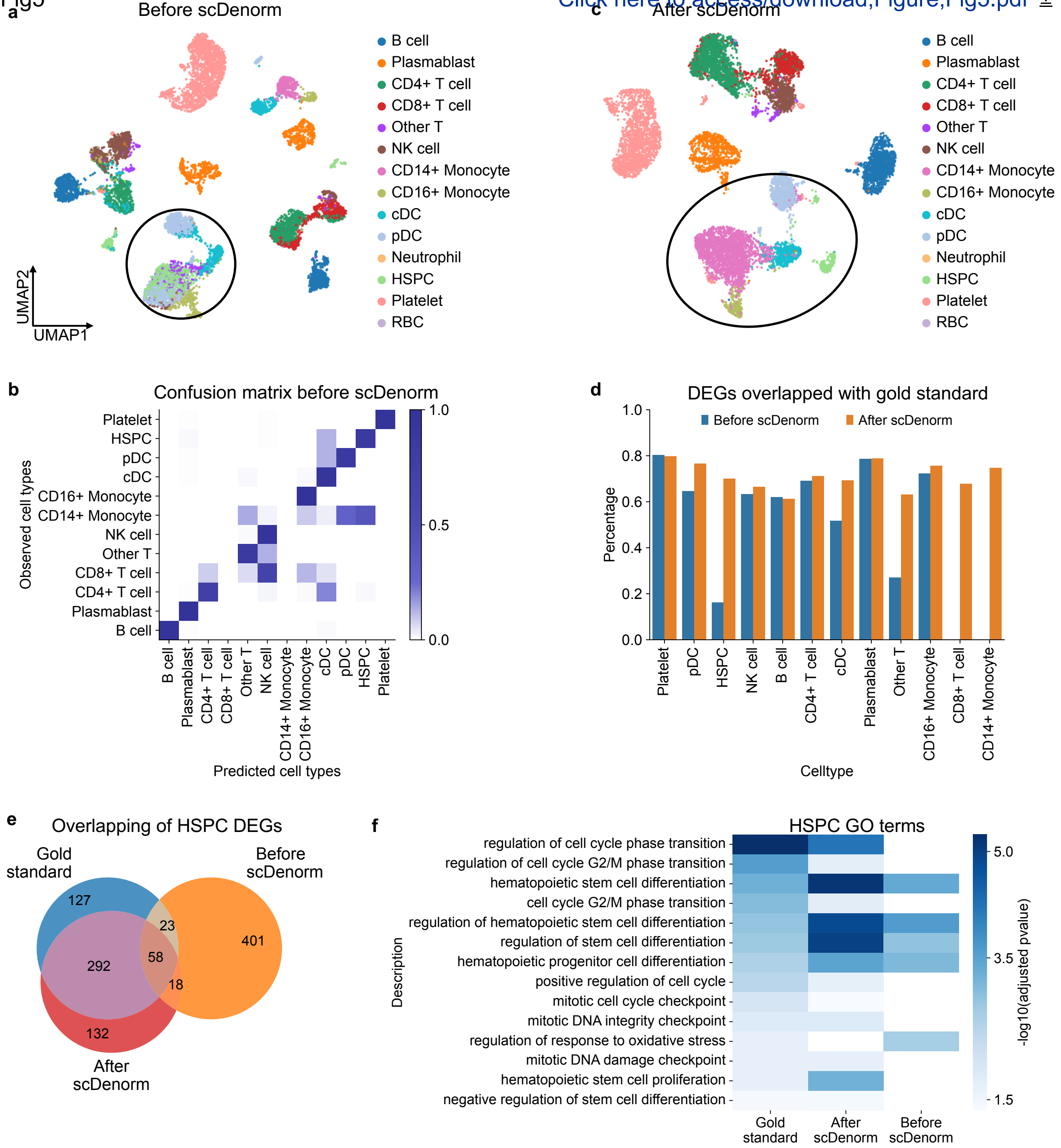

Click here to access/download;Figure;Fig6.pdf

Fig6

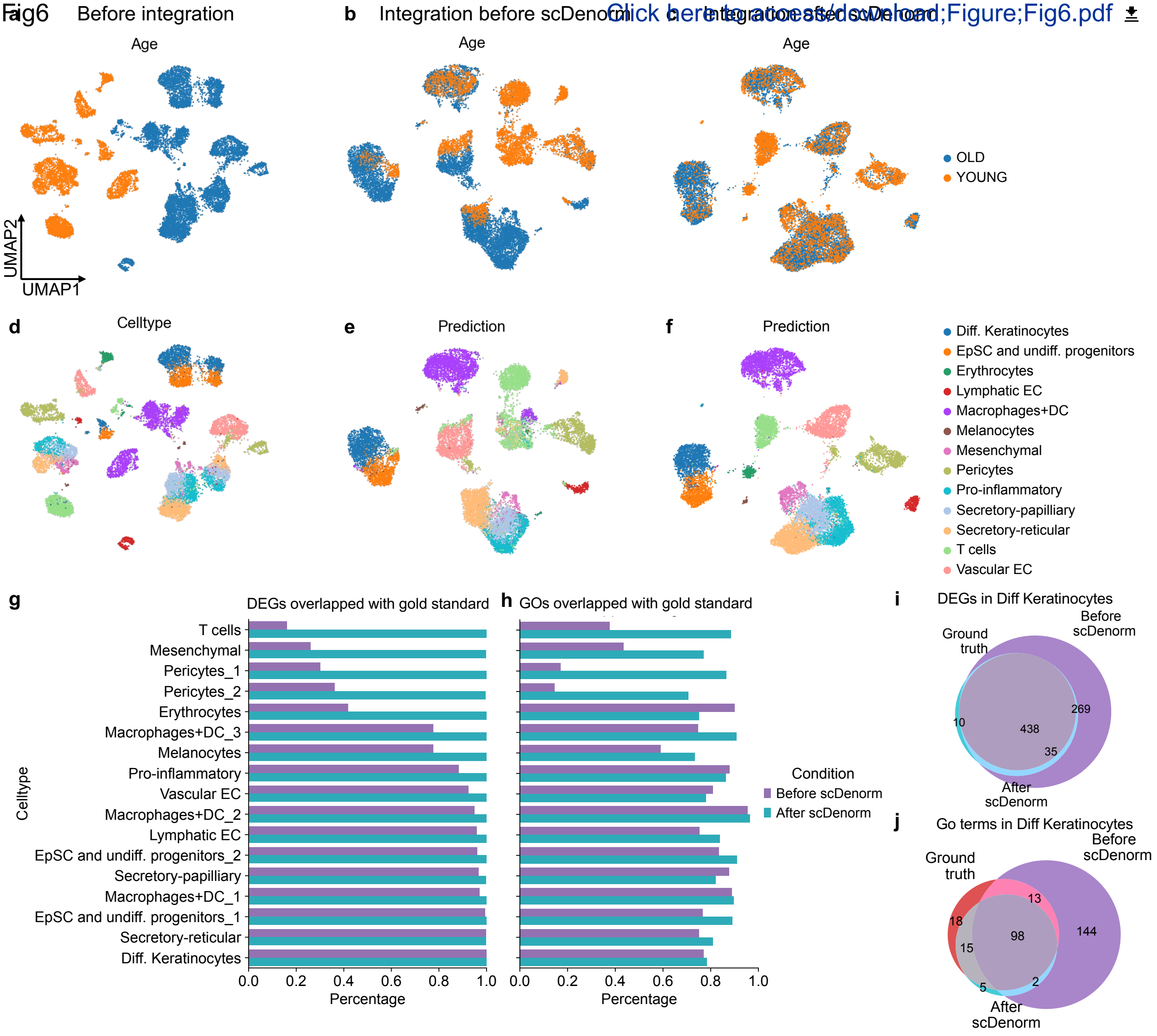

Click here to access/download
Supplementary Material
SupplementaryInformation_R1.docx

Dear Dongni,
Thanks very much for the positive feedback! We have now carefully checked the reviewers' comments. I think the only one left is comment 5 from reviewer #3, which is about the reproducibility and automation of our code. We would like to completely solve this problem in two ways: 1. We have now provided a Docker to include all the necessary environment required by our program; 2. We have now provided a README with details on running our programs. We have now run this workflow on our computers several times. If the reviewer still has a problem running the program, we would be happy to Zoom chat to solve the problem. We greatly appreciate the reviewer's check of the program, as we know the robustness and user-friendliness of a program affect its popularity. We are providing a point-to-point answer to all these comments as below:

**Comments**	**Answers**
Dear Prof. Miao,<br><br>Your manuscript "scDenorm: a denormalisation tool for integrating single-cell transcriptomics data" (GIGA-D-25-00209R1) has been assessed by our reviewers. Based on these reports, and my own assessment as Editor, I am pleased to inform you that it is potentially acceptable for publication in GigaScience, once you have carried out some essential revisions suggested by our reviewers.<br><br>Their reports, together with any other comments, are below. Please also take a moment to check our website at https://www.editorialmanager.com/giga/ for any additional comments that were saved as attachments.<br><br>In addition, please register any new software application in the bio.tools and SciCrunch.org databases to receive RRID (Research Resource Identification Initiative ID) and biotoolsID	We would like to thank you and the referees for supporting our manuscript.

identifiers, and include these in your manuscript. Computational workflows should be registered in workflowhub.eu and the DOIs cited in the relevant places in the manuscript. These will facilitate tracking, reproducibility and re-use of your tool.<br><br>Once you have made the necessary corrections, please submit a revised manuscript online at:<br><br>https://www.editorialmanager.com/giga/<br><br>If you have forgotten your username or password please use the "Send Login Details" link to get your login information. For security reasons, your password will be reset.<br><br>Please include a point-by-point within the 'Response to Reviewers' box in the submission system. Please ensure you describe additional experiments that were carried out and include a detailed rebuttal of any criticisms or requested revisions that you disagreed with. Please also ensure that your revised manuscript conforms to the journal style, which can be found in the Instructions for Authors on the journal homepage. If the data and code has been modified in the revision process please be sure to update the public versions of this too.<br><br>The due date for submitting the revised version of your article is 22 Apr 2026.<br><br>We look forward to receiving your revised manuscript soon.<br><br>Best wishes,	

Dongni Ma, Ph.D.<br>GigaScience<br>www.gigasciencejournal.com	
Editor comment:<br><br>As you revise the manuscript, you might find these recent GigaScience articles on single-cell RNA-seq normalization and integration informative, as they discuss related approaches and tools that could provide useful context for readers.<br><br>(1) Martin Fahrenberger, Christopher Esk, Jürgen A Knoblich, Arndt von Haeseler, GTestimate: improving relative gene expression estimation in scRNA-seq using the Good–Turing estimator, GigaScience, Volume 14, 2025, giaf084, https://doi.org/10.1093/gigascience/giaf084<br><br>(2) Yichao Hua, Linqian Weng, Fang Zhao, Florian Rambow, SeuratExtend: streamlining single-cell RNA-seq analysis through an integrated and intuitive framework, GigaScience, Volume 14, 2025, giaf076, https://doi.org/10.1093/gigascience/giaf076<br><br>(3) Won-Min Song, Chen Ming, Christian V Forst, Bin Zhang, Unsupervised multiscale clustering of single-cell transcriptomes to identify hierarchical structures of cell subtypes, GigaScience, Volume 14, 2025, giaf111, https://doi.org/10.1093/gigascience/giaf111<br><br>--<br>Please also take a moment to check our website at https://www.editorialmanager.com/giga/l.asp?i=240594&l=AMCD	Thanks for the suggestion! We have now included these necessary references in the introduction section.

WVYI for any additional comments that were saved as attachments. Please note that as GigaScience has a policy of open peer review, you will be able to see the names of the reviewers.<br><br>____<br>In compliance with data protection regulations, you may request that we remove your personal registration details at any time. (Use the following URL: https://www.editorialmanager.com/giga/login.asp?a=r). Please contact the publication office if you have any questions.	
Reviewer #1: The authors have addressed all of my comments. The manuscript is suitable for publication after formatting in accordance with the journal's regulations.	Many Thanks!
Reviewer #2: The authors have done a commendable job in addressing the concerns and making the tool accessible. The manuscript is now improved and I recommend it for publication.	Many Thanks!
Reviewer #3:<br><br>1. Context<br><br>This report corresponds to a second assessment of the computational reproducibility of the article GIGA-D-25-00209, following a revision by the authors after the first round of review.	Thank you for the rigorous assessment and valuable feedback regarding our manuscript and the reproducibility of our results! We have now addressed the remaining issues following your recommendations, including improving the README file, and providing a functional environment.yaml. See details in answer to **comment 5**.

The scope of the computational reproducibility review is to reproduce the results in figure 5f related to the evaluation of whether scDenorm improves the biological relevance of gene expression analyses by comparing GO term enrichment from differentially expressed genes (DEGs), before and after denormalization against a gold standard.	
2. Changes since the first review<br><br>The authors made several changes based on comments from the initial computational<br>reproducibility review:<br>- Reorganized and updated the code in Fig5.ipynb and R_goanalysis.ipynb,<br>- Created a docker environment,<br>- Provided pre-computed GO enrichment results and intermediate files in Zenodo,<br>- Added an environment.yaml file for python and installed_packages.csv file for R,<br>- Improved the Readme file.	Thanks!
3. Availability of Materials<br>a. Data<br>- Data availability: Open<br>- Data completeness: Complete = all data necessary to reproduce main results are available	Thanks!

- Access Method: Repository<br>- Repository: https://zenodo.org/records/17275776 (new link)<br>-Data quality: Completed, no metadata was shared.<br><br>b. Code<br>- Code availability: Open<br>- Programming Language(s): R and Python<br>- Repository link: https://github.com/rnacentre/scDenorm_reproducibility<br>- License: -<br>- Repository status: Public<br>- Documentation: A Readme file is provided, but some improvements are needed.	
4. Computational environment of reproduction analysis<br><br>- Operating system for reproduction: MacOS 15.6.1<br>- Programming Language(s): R (jupyter notebook), Python (jupyter notebook)<br>- Code implementation approach: Using shared code<br>- Version environment for reproduction: Docker version 28.5.1, R version 4.5.1 (2025-06-13), Python 3.13.9	Thanks!
5. Results<br><br>5.1 Original study results<br>- Results 1: In the revised version 1 of the paper , Figure 5 does not appear in the PDF. Therefore, we assumed that the figure is identical to the one in the original submission, especially based	Thank you for your valuable feedback and for highlighting the issues encountered during the reproduction of Figure 5f.<br><br>We apologize for any confusion and have updated our README file to include detailed instructions on how to run our scripts, which can be found here

on the authors' comment stating that "We re-ran the analysis and obtained results consistent with those reported in the manuscript."<br>Below is Figure 5f from the original paper:<br><br>(See screenshot)<br><br>The intermediate file "PBMC_go_analysis_result.csv" shared in Zenodo was used to run the authors' code and extract the numerical values of this graph, enabling direct comparison:<br><br>(See screenshot)<br><br>5.2 Steps for reproduction<br><br>-> Follow the readme guidelines to set up the environnement:<br>--> Download the notebooks from Github.<br>Note: notebook list in readme is not updated.<br>--> Install docker and jupyter.<br>Note: the jupyter installation is not precised in the readme file.<br>--> Download data.<br>--- Issue 1: To download the data, no link was provided in the readme file in the Github repository. The zenodo link in the manuscript was not updated in the "Availability of Data and Materials" section.<br>---- Resolved: The new link was provided in the authors' response to the reviewer but needs to be added in the manuscript and the readme file. The link is https://zenodo.org/records/17275776.<br>--- Issue 2: Guidelines in the README file do not	(https://github.com/rnacentre/scDenorm_reproducibility). We have also revised the environment.yaml and installed_packages.csv files to facilitate the setup of the environment locally, along with clear installation steps.<br><br>Regarding the discrepancies in the p-values and the GO enrichment analysis, we ensure that additional clarifications are provided in the README file to address these concerns.<br><br>The Readme details to reproduce figure 5 are as follows:<br><br>**Tutorial for Running Notebooks**<br><br>Download the Notebooks: Clone or download this repository from GitHub: scDenorm GitHub Repository or from Zenodo: scDenorm Data.<br><br>Download and install Docker and Jupyter: Follow the instructions for installation: Docker Get Started.<br><br>**Download Data:**<br><br>Download the data file from Zenodo: scDenorm Data.<br><br>Unzip the downloaded data and place the relevant files into the scDenorm_reproducibility/data folder.<br><br>Run Docker Image:<br><br>Ensure Docker is running.<br><br>Load the Docker image directly from the .tar.gz file:

correspond to the actual procedure.<br>---- Resolved: From the Zenodo archive, download scDenorm_reproducibility.tar.gz, unzip it, and place the data into the data folder. It would be clearer if the authors explicitly specified which files should be placed in the data directory to avoid confusion.<br>--> Run the docker image.<br>--- Issue 3: The following Docker instructions provided by the authors do not work as written:<br>tar -xzf scdenorm_v0.tar.gz<br>docker load -i scdenorm_v0.tar<br>docker run -p 8888:8888 -v /path/to/scDenorm_reproducibility:/app scdenorm_v0 \ jupyter lab --ip=0.0.0.0 --no-browser --allow-root<br>scdenorm_v0.tar.gz does not contain a standard Docker .tar image. After extraction, the result is a directory named scdenorm_v0, not a .tar file. docker load -i scdenorm_v0.tar fails because scdenorm_v0.tar does not exist. Docker must be running before executing docker load. The extraction step is sensitive to the current directory, but this is not documented.<br>---- Resolved: The image can be successfully loaded directly from the .tar.gz file using:<br>docker load < scdenorm_v0.tar.gz<br>After this, the image scdenorm_v0:latest is available.<br><br>--- Issue 4: Two main issues appeared when running the docker run command:<br>----- "WARNING: The requested image's platform (linux/amd64) does not match the detected host platform (linux/arm64/v8)"<br>----- "mounts denied: The path	docker load < scdenorm_v0.tar.gz<br><br>or<br><br>tar -xzf scdenorm_v0.tar.gz<br><br>docker load -i scdenorm_v0.tar<br><br>Run the Docker container with the following command (update the local path accordingly):<br><br>docker run --platform linux/amd64 \\<br><br>-p 8888:8888 \\<br><br>-v /path/to/scDenorm_reproducibility/data:/app \\<br><br>scdenorm_v0 \\<br><br>jupyter lab --ip=0.0.0.0 --no-browser --allow-root<br><br>Note: Ensure to share the project folder with Docker. Go to Docker → Preferences → Resources → File Sharing and add the local project path.<br><br>**Example running Fig5.ipynb**<br><br>Open Fig5.ipynb.<br><br>Select Kernel > Change Kernel > Python [conda env: sc].<br><br>Data Import: Copy the data from Zenodo into scDenorm_reproducibility/data, including: fig5_input.h5ad,

/path/to/scDenorm_reproducibility is not shared from the host".<br>---- Resolved: To be able to use the docker run command, two steps were needed:<br>----- Share the project folder with docker manually: Docker → Preferences → Resources → File Sharing → add the local project path<br>----- Update the docker run command with the local path and add linux/amd64:<br><br>docker run --platform linux/amd64\\<br>-p 8888:8888\\<br>-v /path/to /scDenorm_reproducibility:/app\\<br>scdenorm_v0\\<br>jupyter lab --ip=0.0.0.0 --no-browser --allow-root<br><br>--- Issue 5: R was not connected to Jupyter.<br>---- Resolved: In the terminal, this made the R kernel available:<br><br>R<br>install.packages("IRkernel")<br>IRkernel::installspec()<br><br>-> Run the Fig5_R__goanalysis.ipynb script<br>--- Issue 6: Docker image does not install the R packages. The file installed_packages.csv lists all required R packages, but they are not installed automatically.<br>---- Resolved: A solution was to install all required packages at the start of the notebook using the csv file:<br>pkg_list <- read.csv("installed_packages.csv", stringsAsFactors = FALSE)	PBMC_before_scDenorm.h5ad, PBMC_after_scDenorm.h5ad, PBMC_groundgo.csv, PBMC_beforego.csv, PBMC_aftergo.csv<br><br>**Run the notebook cells in order.**<br><br>Example running Fig5_R_goanalysis.ipynb<br><br>Open Fig5_R_goanalysis.ipynb.<br><br>Select Kernel > Change Kernel > R [conda env: sc].<br><br>Data Import: Copy the data from Zenodo into scDenorm_reproducibility/data, including: PBMC_raw_count_b0_deg.csv, PBMC_raw_count_b1_deg.csv, PBMC_normlized_data_1e3_b1_deg.csv<br><br>Run the notebook cells in order.

for (pkg in pkg_list$Package) {<br>if (!requireNamespace(pkg, quietly = TRUE)) {<br>message(" Installing the package: ", pkg)<br>tryCatch(<br>{<br>install.packages(pkg, dependencies = TRUE)<br>},<br>error = function(e) {<br>message("Failed to install package: ", pkg)<br>}<br>)<br>} else {<br>message(" Already installed: ", pkg)<br>}<br>}<br>Additional required packages from Bioconductor:<br><br>if (!require("BiocManager", quietly = TRUE))<br>install.packages("BiocManager")<br>if (!requireNamespace("enrichplot", quietly = TRUE)) {<br>BiocManager::install("enrichplot", ask = FALSE)}<br>if (!requireNamespace(c("enrichplot","org.Hs.eg.db"), quietly = TRUE)) {<br>BiocManager::install(c("clusterProfiler", "org.Hs.eg.db"), ask = FALSE)}<br><br>After these steps, the R script ran without errors.<br><br>-> Run the Fig5.ipynb script<br>--- Issue 7: The same issue as no. 3 occurred again, the	

docker image did not provide a working python environment. Attempt to create the python environment with environment.yaml file.<br>conda env create -f environment.yaml<br>Failed because many packages do not exist for the system, for exemple:<br>"ipyw_jlab_nb_ext_conf ==0.1.0 py39h06a4308_1 does not exist (perhaps a typo or a missing channel);"<br>These errors seem to happen because the environment file contains many Linux-specific packages.<br>---- Unresolved: Authors should provide an environment file working in all systems. A temporary solution was used: create a minimal clean environment:<br>conda env create -f environment.yaml<br>Environment.yaml:<br>name: scdenorm_clean<br>channels:<br>- conda-forge<br>- bioconda<br>- defaults<br><br>dependencies:<br>- python=3.9<br>- numpy<br>- pandas<br>- scipy<br>- matplotlib<br>- seaborn<br>- tqdm<br>- scanpy<br>- anndata	

- tables<br>- pip<br><br>- pip:<br>- scdenorm<br>- SCCAF<br><br>Then:<br><br>conda activate scdenorm_clean<br>conda install ipykernel<br>python -m ipykernel install --user --name=scdenorm_clean --display-name "Python (scdenorm)"<br><br>Select this kernel in Jupyter Notebook to run the python files.<br><br>An additional issue was the conflict between matplotlib and scapy. Resolved with:<br><br>conda install matplotlib=3.6.3<br>conda install -c conda-forge scanpy (Successfully installed scanpy-1.10.3)<br><br>--> The script was executed only by starting from HSPC section.<br>--- Issue 8: A specific issue appeared after filtering the dataframe tmp1 by go_terms, only two cell types remained (b0 and b1), and b1n disappeared. This was because no row corresponding to b1n matched the selected GO terms.<br>---- Unresolved: Fig5_R__goanalysis.ipynb was re-run	

multiple times to obtain a new version of the PBMC_go_analysis_result.csv. However, the error persists.<br><br>5.3 Statistical comparison Original vs Reproduced results<br>- Reproduced results: Figure 5f<br><br>(see screenshots)<br><br>- Comments: The figure obtained does not show all go_terms nor all categories. Only categories b1 and b0 are shown.<br>- Errors detected: -<br>- Statistical Consistency: If there is no error, b0 would correspond to the gold standard and b1 to the before_scDenorm cell type. The -log10(adjusted p-value) values reproduced do not match the reported values.	
6. Conclusion<br>- Follow-up on previous recommendations:<br>In the first round of review, we noted the following points:<br>-- Add a requirement file that lists all the needed packages with their exact versions. Authors provided an installed_packages.csv which allowed to manually reconstruct the R environment. However, a functional environment.yaml is required.<br>-- Make sure all data files needed to reproduce the figures are available in the repository. The authors updated the Zenodo link and uploaded all relevant intermediate files.<br>-- Clearly explain which parts of the results may vary due to randomness in the model and how much variation users should expect. This point remains insufficiently addressed.	Thanks very much for the nice suggestions to improve and automate the reproducibility! Following them, we have now revised our codes and scripts. Details have been listed in answers to **comment 5**.

- Summary of the second computational reproducibility review<br><br>Both scripts used to reproduce the figure 5f were executed, but several issues were encountered. The results obtained differ from the ones reported in the manuscript. In particular:<br>-- Several p-values could not be reproduced,<br>-- Some discrepancies appeared in the GO enrichment analysis.<br>Some clarifications are required for the GO analysis about why some cell types are not present after filtering.<br><br>Significant manual intervention was required, to improve the reproducibility, here is some new recommendations:<br>-- Improve the readme file. The readme does not reflect the real procedure needed to reproduce the results (incorrect docker instructions, missing steps, outdated notebook list). Clear instructions should be added regarding:<br>--- the required jupyter installation,<br>--- file paths and folder structure,<br>--- link to the zenodo<br>--- how to run each notebook<br>-- Provide a functional environment.yaml. The provided docker image fails to create the required Python and R environments.	
