# Supplementary material for: scDenorm: a denormalization tool for integrating single-cell transcriptomics data
Source: Gigascience. 2026 Mar 31;15:giag032. doi: 10.1093/gigascience/giag032 (PMC13142155; doi:10.1093/gigascience/giag032)
Supplement: giag032_Authors_Response_To_Reviewer_Comments_original_submission [file giag032_authors_response_to_reviewer_comments_original_submission.pdf]

Author's Response To Reviewer Comments

Dear Nogoy,

We gratefully appreciate your feedback and all the comments from the reviewers. The constructive feedback helped us to improve our manuscript. We have carefully addressed the concerns raised by the reviewers. Please find our point-by-point responses below:

0.0 Your manuscript "scDenorm: a denormalisation tool for integrating single-cell transcriptomics data" (GIGA-D-25-00209) has been assessed by our reviewers. Although it is of interest, we are unable to consider it for publication in its current form. The reviewers have raised a number of points which we believe would improve the manuscript and may allow a revised version to be published in GigaScience.

Their reports, together with any other comments, are below. Please also take a moment to check our website at https://www.editorialmanager.com/giga/ for any additional comments that were saved as attachments.

In addition, please register any new software application in the bio.tools and SciCrunch.org databases to receive RRID (Research Resource Identification Initiative ID) and biotoolsID identifiers, and include these in your manuscript. Computational workflows should be registered in workflowhub.eu and the DOIs cited in the relevant places in the manuscript. These will facilitate tracking, reproducibility and re-use of your tool.

If you are able to fully address these points, we would encourage you to submit a revised manuscript to GigaScience. Once you have made the necessary corrections, please submit online at:

https://www.editorialmanager.com/giga/

If you have forgotten your username or password please use the "Send Login Details" link to get your login information. For security reasons, your password will be reset.

Please include a point-by-point within the 'Response to Reviewers' box in the submission system. Please ensure you describe additional experiments that were carried out and include a detailed rebuttal of any criticisms or requested revisions that you disagreed with. Please also ensure that your revised manuscript conforms to the journal style, which can be found in the Instructions for Authors on the journal homepage. If the data and code has been modified in the revision process please be sure to update the public versions of this too.

The due date for submitting the revised version of your article is 05 Nov 2025.

I look forward to receiving your revised manuscript soon.

Thank you for the feedback! We have carefully addressed all reviewer comments and made substantial improvements to enhance clarity, reproducibility, and usability.
Key updates include:
The human prefrontal cortex (PFC) integration example, which demonstrates the utility of scDenorm across studies with different pipelines, has been promoted to main Figure 7, as suggested.
A ready-to-use Docker image and detailed environment files (environment.yaml for Python and installed_packages.csv for R) to ensure reproducibility.
All required input data, metadata, and pre-computed results have been made available via Zenodo (https://zenodo.org/uploads/17275776).
A new “How to install and use scDenorm” section added in the GitHub README, with installation instructions, a quick-start example, and a link to full documentation.
scDenorm has been registered in bio.tools (biotoolsID: scdenorm) and SciCrunch.org (RRID:

SCR_027574), and the corresponding identifiers have been included in the manuscript.
We believe these revisions significantly improve the manuscript and its reproducibility. Please find all these improvements below in our point-to-point answers to the reviewers' comments.

Reviewer 2

1.0 Gigascience review for Huang et al, scDenorm: a denormalisation tool for integrating single-cell transcriptomics data
The authors aim to address the important and often irritating problem of how to combine scRNAseq data from different sources and provided after processing with different normalization tools. The authors note that often times raw counts matrices are not provided, nor information on how the data was normalized. Their tools aim to estimate the parameters used for data transformation (eg. scale factor, pseudocount addition, log-transformation), and reverse those transformations to return to the raw counts matrix again. In an ideal world where public data is well-documented and actually fully available, this type of reverse-engineering would not be necessary, but due to the current state of many data repositories, in many cases, the tools the authors provide would be indeed very useful. The paper does a good job at identifying the problem, implementing a systematic solution with useful benchmarks, and showing multiple examples of the method's utility using public datasets. However, a few additional questions arise about their method:
Thank you for your recognition of the tool we have developed. Following are the answers for reviewers' comments and suggestions one by one.
1.1 Figure 1c-d: UMAP is a distortion/projection onto a 2D space, so it is not super surprising that different scaling factors produce clouds of cells in different locations. Do the differences in scaling factors affect the identification of the cells in any way? I.e. After denormalization for datasets of different scale factors/logbases/pseudocounts, when performing renormalization on the newly obtained raw counts, is there an optimal set of normalization parameters to now use?

Thank you for the comment. As illustrated in Figures 5–7, differences in normalization methods among datasets can lead to artificial inflation or suppression of marker gene expression. This, in turn, affects the accuracy of cell type identification. For example, in Figure 5b, the confusion matrix heatmap shows that a substantial proportion of $CD14^{+}$ monocytes were misclassified as HSPCs or pDCs, which we attribute to inconsistencies in normalization across datasets.

To avoid such bias, we recommend starting from raw counts and applying a consistent normalization method across datasets. This minimizes technical biases and ensures that biological signals are preserved, thereby improving the reliability of downstream analyses such as cell type annotation and differential expression.

1.2 Figure 2: For a particular dataset, if it is not known how the data was normalized and the transformation did not follow the form of log(C/T + P), will the tool alert the user to this? Or will the tool still attempt to find the best fit log base and pseudocount? In other words, prior to performing the denormalization, it might be helpful to display some metrics that indicate that the delta method was the most likely normalization method used, especially when ones tries to apply the tool to hundreds of datasets. (However, it seems it may not be possible to tell whether deNorm is successful until it is attempted (both empirically trying common log-base/pseudocount combinations, or solving the optimization equation), and then one assesses whether the resulting data is negative binomially distributed.)

Thank you for the suggestion. scDenorm is designed to automatically infer the most likely normalization

parameters—specifically the log base and pseudocount—under the assumption that the transformation follows the form log(C/T + P). If the data conforms to this model and the resulting denormalized values follow a negative binomial distribution, the tool will report the best-fit parameters used.

However, if the data does not match the expected transformation form or fails to follow a negative binomial distribution, it will notify the user that denormalization was unsuccessful and return the original input data. This ensures transparency and prevents misleading downstream analysis.

1.3 Figure 3d does not seem sufficiently described in the text. The authors write: "For recovery error, the absolute values are below 10-6 in 27 datasets normalized with natural logarithmic transformation (Fig. 3d, Supplementary Table 1), indicating a good accuracy of scDenorm (Fig. 3f)." Fig 3f does show recovery error, and Fig 3e shows rounding error, but what does Fig 3d intend to show? Text clarification would be helpful.

Thank you for pointing this out. Figure 3d presents the distribution of success rates across the 27 datasets that were normalized using the natural logarithmic transformation.

As defined in Equation (11) method section (see P13L495), the success rate is calculated as the proportion of cells that were successfully denormalized:
success rate = Nsuccess/Ntotal (11)

Nsuccess is the number of cells for which scDenorm was able to recover valid raw counts, and Ntotal is the total number of cells in the dataset. In some cases, denormalization may fail for a subset of cells due to poor sequencing quality or low gene counts. Figure 3d visualizes this variability across datasets.

We now revise the main text to explain the purpose of Figure 3d as below:

P5L184
To further assess the robustness of scDenorm across diverse datasets, we evaluated its performance on 27 datasets that were normalised using a natural logarithmic transformation. We present the distribution of success rates (Fig. 3d), defined as the proportion of cells that were successfully denormalised (Equation 11). This metric accounts for cases where poor sequencing quality or a low number of expressed genes may result in cell-wise deviations from the expected negative binomial distribution, thereby preventing accurate recovery during denormalisation. The results demonstrate that scDenorm performs robustly across diverse datasets, even when some cells cannot be fully recovered.

P13L495
In certain cases, not all cells can be successfully denormalised due to poor sequencing quality, or a low number of expressed genes. To evaluate denormalisation in such situations, we define success rate as the percentage of successfully denormalised cells, equation (11).

success rate = Nsuccess/Ntotal (11)

Nsuccess is the number of successfully denormalised cells, while Ntotal is the total number of cells.

1.4 For Fig 3d, why do certain datasets have lower "success rate"? It would be helpful for the bars in Fig 3d to be in the same order as the blue bars in Fig 3b, to better compare any relationship between gene or cell number with "success rate".

Thanks for this insightful comment. Certain datasets exhibit lower success rates in Figure 3d due to technical issues such as poor sequencing quality or a low number of expressed genes (filtered datasets),

which may cause individual cells to deviate from the expected negative binomial distribution. Since scDenorm relies on this distributional assumption to accurately recover raw counts, such deviations can hinder successful denormalisation.

We have a supplementary figure to match the order between Figure 4d and Figure 3b to better compare any relationship between gene or cell number with "success rate". Please see the Supplementary Figure 7 as below.

Supplementary Fig. 7 Benchmark analysis on the datasets from
Brain Cell Atlas
a, The bar plot shows the number of genes in each dataset. The x-axis is the name of datasets (same as d), and the y-axis is the log-scaled number of genes.
b, The bar plot shows the number of cells in each dataset. The x-axis is the name of datasets (same as d), and the y-axis is the log-scaled number of cells.
c, The bar plot shows the success rate for each dataset. The x-axis is the name of datasets (same as d), and the y-axis is the success rate (see Method).
d, The jitter plot shows the distribution of rounding errors observed in the denormalised datasets from the Brain Cell Atlas. The x-axis is the name of datasets, and the y-axis is the rounding error.
1.5 In addition, from Methods it appears that the determination of "Success Rate" relies on knowing the original raw count values. For those data sets where many of the cells could not be successfully denormalized to raw values, what is the impact of that on the downstream cell identification or DEG analysis?
Sorry for the confusion. The calculation of success rate does not require access to the original raw count values. Instead, we evaluate whether a cell has been successfully denormalised based on the rounding error, defined as the difference between the denormalised value and its nearest integer. If the denormalised values are very close to integers (default: the rounding error<0.01), this indicates a successful recovery of count-like data.

Cells with large rounding errors are considered unsuccessfully denormalised and are filtered out prior to downstream analysis. As these cells typically correspond to low-quality profiles, their exclusion does not affect downstream tasks such as cell type identification or differential expression analysis. This filtering step ensures that only reliably recovered data are used for biological interpretation.
1.6 Figure 4f: Not sure if this is an error in image display, but I do not see any blue points (original data) in the UMAP, as described in the legend.
Thanks. The absence of visible blue points (original data) in Figure 4f is due to the fact that they are largely overlapped by the orange points, as both sets of coordinates are nearly identical.

To clarify this, we have included a figure showing the UMAP plots for the three conditions separately as below:

Revision Fig 1. Separate UMAP plots of different conditions (Origin, Floating, and HVG)

1.7 Figures 5-6: The authors mention assessing how scDenorm helps in different scenarios where one might want to combine data: "the batch in the COVID-19 PBMCs dataset are samples from two patients, while in the human prefrontal cortex (PFC)31,35 dataset are samples from two different studies. And the batch in the human skin36 dataset are groups of samples from young and old donors."
The most interesting scenario here are the samples from two different studies, perhaps performed by different labs, with different bioinformatic pipelines, mapped to different versions of the genome, etc. Thus, I thought the example of integrating human PFC from two different studies and how the cell type identification changed (via the river plots) was the most compelling example, and the authors could consider making that a main figure instead.
Thanks for the thoughtful feedback. We have promoted the human prefrontal cortex example to a main figure (Figure 7) in the revised manuscript. The river plots clearly demonstrate how scDenorm improves cell type identification by reducing misclassification and enhancing consistency across batches. We believe

this example effectively illustrates the utility of scDenorm in real-world multi-study integration tasks.
1.8 The linked github repository, while commendable in providing code to reproduce figures, could benefit from further documentation/README showing the user how to use the tool.
Thanks. We have updated the README file to include a dedicated section titled "How to install and use scDenorm" as below. This section provides clear instructions on installation, a quick-start example for applying scDenorm to a dataset, and a link to the full documentation for further details.

How to use and install scDenorm
- Install: pip install scDenorm
- Usage: scdenorm data/pbmc3k_norm.h5ad --fout data/pbmc3k_denorm.h5ad
- Documentation: https://changebio.github.io/scDenorm

Reviewer 3

Reproducibility report for: scDenorm: a denormalisation tool for integrating single-cell transcriptomics data
Journal: GigaScience
ID number/DOI: GIGA-D-25-00209
Reviewer(s): Laura Caquelin, Department of Clinical Neuroscience, Karolinska Institutet, Sweden
----
1. Summary of the study
In order to reduce biaises caused by different normalization methods, the authors developed scDenorm, "an algorithm that reverts normalised single-cell omics data to raw counts". They evaluated its performance on large-scale datasets to assess its impact on data integration and downstream analysis.
----
2. Scope of reproducibility
According to our assessment the primary objective is: to evaluate whether scDenorm improves the biological relevance of gene expression analyses by comparing GO term enrichment from differentially expressed genes (DEGs), before and after denormalization against a gold standard.
Note: While this is not the main outcome highlighted in the article's conclusion, we chose to focus on the GO enrichment analysis because its involves statistical inference abd provides a clear and reproducible downstream result.
- Outcome: GO enrichment profiles based on hematopoietic stem and progenitor cells for each condition (gold standard, before scDenorm and after scDenorm).
- Analysis method outcome: "Downstream analysis after cell type annotation includes differential expression (DE) analysis and Gene Ontology (GO) pathway analysis. Differential gene expression analysis was conducted for each cell type (one against the rest) using the Wilcoxon test implemented in SCANPY19. As part of the dataset is used as the reference dataset, the differentially expressed genes (DEGs) derived from the reference dataset is used as 'gold standard' in our evaluation. The same approach was used for the calculation of the DEGs before and after scDenorm. The top differentially expressed genes were compared across different thresholds (top 50, 100, 200, 500, and 1000). As for GO pathway analysis, the enrichGO program was used on the top 500 differential expression genes.
DE and GO analysis with the correct labels from Solé-Boldo et al. was conducted for each cell type (one against the rest) using the Wilcoxon test implemented in Seurat (V3.1.1), same as the version described in Solé-Boldo et al. These analyses were conducted before scDenorm and after scDenorm. The differentially expressed genes were obtained from the study's supplementary materials as 'gold standard'". (Method part, Dataset processing for data integration and downstream analysis section, page 14-
15)
- Main result: "Gene enrichment analysis of the differential genes in HSPCs indicated
that the GO terms enriched after scDenorm closely aligned with those of the 'gold standard', whereas the enrichment before scDenorm showed minimal overlap (Supplementary Fig. 10f). The enriched GO terms are relevant functions associated with HSPC cells, such as hematopoietic stem cell proliferation and hematopoietic progenitor cell differentiation (Fig. 5f)." (Results part, page 7)
----
3. AvailabilityofMaterials a. Data
- Data availability: Open

- Data completeness: Some data necessary to reproduce the main results were initially missing but were provided by the authors after we requested them.
- Access Method: Repository
- Repository: https://doi.org/10.5281/zenodo.11240443
-Data quality: The data files have been shared and appear sufficient for running the analyses. However, no metadata is provided to describe the content, structure, or origin of the files which limits interpretability and reusability.
b. Code
- Code availability: Open
- Programming Language(s): Python (Jupyter Notebook)
- Repository link: https://github.com/rnacentre/scDenorm-reproducibility
- License: Apache 2.0
- Repository status: Public
- Documentation: The documentation could be improved. Important details about the computational environment are missing, which makes it difficult to set up and reproduce the analysis. Additionally, since the code scripts are long and complex, navigating and understanding the workflow is challenging without clearer explanations.

----
4. Computational environment of reproduction analysis
- Operating system for reproduction: MacOS 15.5
- Programming Language(s): Python, R
- Code implementation approach: Using shared code with the jupyter notebook
- Version environment for reproduction: Creation of an environment with Python 3.10 and R 4.5.0

----

5. Results

5.1 Original study results - Results 1: Figure 5f
5.2 Steps for reproduction
-> Installation and environment setup
- Issue 1: Incompatibility between SCCAF and Python.
-- Resolved: SCCAF, when installed via PyPI, required scanpy==1.4.6, which is incompatible with Python 3.12, causing a TypeError due to metaclass conflicts. A new conda environment was created with Python 3.10. The authors specified that the PyPI version of SCCAF is outdated and recommended using their GitHub repository (https://github.com/rnacentre/sccaf).

- Issue 2: Incompatibility with recent versions of pandas.
-- Resolved: An ImportError occurred due to using pandas==2.2.3.
Downgrading to pandas==1.5.3 solved the issue.

- Issue 3: Incompatibility with recent versions of anndata.
-- Resolved: Using anndata==0.11.4 raised import errors. Downgraded to anndata==0.8.0, which resolved the issue.
Setting up a clean environment with Python 3.10, scanpy==1.9.1, matplotlib==3.5.3, pandas==1.5.3, anndata==0.8.0, and the GitHub SCCAF package resolved the issue. Authors validated the environment configuration above.

-> Running the original notebook (fig5.ipynb)
- Issue 4: KeyError due to missing labels ('Neutrophil', 'RBC') in confusion matrix.
-- Half resolved: Modified indexing logic to handle missing labels. The authors explained this variability stems from model randomness trained by SCCAF. Authors suggested filtering the label list accordingly:
conf_mat = conf_mat.loc[idx, [i for i in idx if i in conf_mat.columns]]
The proposed fix was to filter the columns with [i for i in idx if i in conf_mat.columns], but this still raises the same error due to missing labels in the rows.

conf_mat = conf_mat.loc[[i for i in idx if i in conf_mat.index] , [i for i in idx if i in conf_mat.columns]]
This error recurs throughout the code, requiring numerous modifications to be made.

- Issue 5: KeyError when building Sankey diagram ""Bad flow - couldn't find destination node: ('CD4+ T cell ', 'CD8+ T cell', 148)"
-- Resolved: Authors proposed instead of hardcoding the label list :
for i in new_ad.obs.predict0.value_counts()[['B cell', 'CD4+ T cell', 'NK cell', 'Other T', 'CD16+ Monocyte', 'cDC', 'pDC', 'HSPC', 'Plasmablast', 'Platelet']].items():
Simplify it to:
for i in new_ad.obs.predict0.value_counts().items():

- Issue 6: KeyError: 'predict0'
-- Resolved: Column predict0 did not exist. Replaced with predict and
predict_colors:
tmp = new_ad.obs.groupby(['cell', 'predict']).size()
right_cols = pd.Series(new_ad.uns['predict_colors'], index=new_ad.obs.predict.cat.categories)
Multiple predict0 en predict modifications had to be made throughout the code.

- Issue 7: ValueError no field of name Neutrophil
-- Unresolved: New error in this part of code:
df_bench=[]
for i in idx:
tmp=[]
for j in [50,100,200,500,1000]: tmp.append(len(set(adx0.uns['ground']['names'][i][:j]).intersection(adx0.uns['wilcoxon']['names'][i][:j])))
df_bench.append(tmp)

After asking authors about this error, they precised that if the goal is to reproduce the results of scDenorm, the Sankey plot part can be skip and proceed directly to the section "After scDenorm".

-> Running the original notebook (fig5.ipynb) - "After scDenorm" section
- Issue 8: NameError: name 'ad1' is not defined
-- Resolved: Replaced ad1 with ad3, which had been defined earlier. Authors confirmed this change is correct.

- Issue 9: AttributeError: 'DataFrame' object has no attribute 'feature_name'
-- Unresolved: The line of code "ad.var_names=list(ad.var.feature_name)
not working. feature_name column not present in ad.var. This part was skipped.

- Issue 10: KeyError: 'wilcoxon' in plotting DEG results
-- Resolved: Clarified that 'wilcoxon' is the method, not the key. Correct usage:
sc.pl.rank_genes_groups(adx0, n_genes=25, sharey=False, key="ground")
- Issue 11: Missing data files
-- Resolved: The following files are required but not found in the repository: *
PBMC_before_scDenorm_DEGs.pkl
* PBMC_after_scDenorm_DEGs.pkl
* PBMC_go_analysis_result.csv
After request, the authors provided the script R_goanalysis.ipynb, which reproduces the PBMC_go_analysis_result.csv file, along with the necessary data files.
-> Running the R_goanalysis.ipynb and the "DEG and GO venn" section

5.3 Statistical comparison Original vs Reproduced results - Results:

- Comments: As the results we obtained were completely different from those reported in the manuscript, and identical before and after scDenorm, I contacted the authors to request details about the computational R environment to run the code R_goanalysis.ipynb. Although we used the environment they provided, this did not resolve the discrepancy.

- Errors detected: -
- Statistical Consistency: We obtained results that were completely different from those reported in the original study. Moreover, the outputs for the "after scDenorm" and "before scDenorm" conditions appeared graphically identical.
----

6. Conclusion
- Summary of the computational reproducibility review
The reproduction of the results in the scope of this computational reproducibility review was not successful. Many errors were encountered when running the original fig5.ipynb notebook, including missing or outdated variables, missing files, and misalignments in hard-coded labels. While most of these issues were resolved through direct communication with the authors and code modifications, some steps remained partially or completely irreproducible.
The results we obtained for the figure 5f were entirely different from those reported in the original manuscript. Despite using the R script (R_goanalysis.ipynb) and data files shared by the authors, and running them in the specified environment, the GO analysis results were inconsistent with those presented in the publication. Furthermore, the "after scDenorm" and "before scDenorm" conditions produced graphically identical results. This raises questions about whether the differences come from the code, the data, or something else in the analysis.

- Recommendations for authors
To make the study easier to reproduce and more transparent, we suggest the following:

-- Add a requirement file that lists all the needed packages with their exact versions and mention which Python and R versions are supported. Make it clear in the documentation that the SCCAF version on PyPI is outdated and point users to the up-to-date version on GitHub.
-- Make sure all data files needed to reproduce the figures are available in the repository.
-- Check that the analysis code does not depend on fixed labels or assumptions that might not work with other datasets or slightly different results.
-- Clearly explain which parts of the results may vary due to randomness in the model and how much variation users should expect.
-- Consider adding some pre-computed results or intermediate files so users can compare their outputs with the original study more easily.

We sincerely thank the reviewer for the detailed and thoughtful reproducibility assessment. We greatly appreciate the effort to test scDenorm and have carefully addressed all points raised to improve the reproducibility, usability, and transparency of our study.
Reproducibility of Figure 5f and GO Analysis
Sorry for the confusion. We have reorganized and updated the code in Fig5.ipynb and R_goanalysis.ipynb (now renamed to Fig5_R_goanalysis.ipynb) to ensure clarity and consistency.
A reproducible docker environment closely matching our local setup has been constructed. We re-ran the analysis and obtained results consistent with those reported in the manuscript.
To facilitate validation, we have provided pre-computed GO enrichment results and intermediate files at Zenodo (https://zenodo.org/uploads/17275776).
Docker Image for Reproducibility
To further simplify setup and ensure reproducibility, we have provided a ready-to-use Docker image that includes all dependencies and environment configurations. This allows users to reproduce results without manual installation.
Improved Documentation and Environment Setup
We have added a comprehensive environment.yaml file for the Python environment and an installed_packages.csv file for the R environment, specifying exact versions of Python, R, and all required packages.
The GitHub README now includes a tutorial for running the notebooks, with step-by-step instructions as below:
Clone the repository:
git clone https://github.com/rnacentre/scDenorm_reproducibility.git

Download the Docker image and data from Zenodo: https://zenodo.org/uploads/17275776.
Move all downloaded data to scDenorm_reproducibility/data folder.
Run the Docker image (How to install docker: https://docs.docker.com/engine/install/):
tar -xzf scdenorm_v0.tar.gz
docker load -i scdenorm_v0.tar
docker run -p 8888:8888 -v /path/to/scDenorm_reproducibility:/app scdenorm_v0 jupyter lab --ip=0.0.0.0 --no-browser --allow-root

Clarification on Model Randomness
We have added annotation in the notebooks which parts of the workflow may vary due to model randomness (e.g., SCCAF training) and provided pre-computed results for reproducibility.
Availability of Data Files and Pre-computed Results for Comparison
All previously missing data files required to reproduce the figures have been added to Zenodo. We have also included pre-computed outputs and intermediate files to support reproducibility and allow users to compare their results with those reported in the manuscript.

We believe these revisions fully address the reviewer’s concerns and significantly improve the reproducibility and transparency of scDenorm. Thank you again for your valuable feedback and suggestion.

Editorial Manager®

Cookie Preference Center

We use cookies which are necessary to make our site work. We may also use additional cookies to analyze, improve and personalize our content and your digital experience. For more information, see our Cookie Policy.

You may choose not to allow some types of cookies. However, blocking some types may impact your experience of our site and the services we are able to offer. See the different category headings below to find out more or change your settings.

Allow all

Manage Consent Preferences

Strictly Necessary Cookies

Always active

These cookies are necessary for the website to function and cannot be switched off in our systems. They are usually only set in response to actions made by you which amount to a request for services, such as setting your privacy preferences, logging in or filling in forms. You can set your browser to block or alert you about these cookies, but some parts of the site will not then work.

Functional Cookies

These cookies enable the website to provide enhanced functionality and personalisation. They may be set by us or by third party providers whose services we have added to our pages. If you do not allow these cookies then some or all of these services may not function properly.

Performance Cookies

These cookies allow us to count visits and traffic sources so we can measure and improve the performance of our site. They help us to know which pages are the most and least popular and see how visitors move around the site.

Targeting Cookies

These cookies may be set through our site by our advertising partners. They may be used by those companies to build a profile of your interests and show you relevant adverts on other sites. If you do not allow these cookies, you will experience less targeted advertising.

Cookie List

Clear

Apply Cancel

Consent Leg.Interest

Confirm my choices

Power
