# Supplementary material for: scDenorm: a denormalization tool for integrating single-cell transcriptomics data
Source: Gigascience. 2026 Mar 31;15:giag032. doi: 10.1093/gigascience/giag032 (PMC13142155; doi:10.1093/gigascience/giag032)
Supplement: giag032_Authors_Response_To_Reviewer_Comments_revision_1 [file giag032_authors_response_to_reviewer_comments_revision_1.pdf]

Author's Response To Reviewer Comments

Dear Dongni,

Thanks very much for the positive feedback! We have now carefully checked the reviewers' comments. I think the only one left is comment 5 from reviewer #3, which is about the reproducibility and automation of our code. We would like to completely solve this problem in two ways: 1. We have now provided a Docker to include all the necessary environment required by our program; 2. We have now provided a README with details on running our programs. We have now run this workflow on our computers several times. If the reviewer still has a problem running the program, we would be happy to Zoom chat to solve the problem. We greatly appreciate the reviewer's check of the program, as we know the robustness and user-friendliness of a program affect its popularity. We are providing a point-to-point answer to all these comments in attached rebuttal file.

We look forward to hearing from you. Thank you in advance for sharing our enthusiasm!

Sincerely,

Zhichao (Chichau) Miao
Guangzhou Laboratory

Editorial Manager®

Cookie Preference Center

We use cookies which are necessary to make our site work. We may also use additional cookies to analyze, improve and personalize our content and your digital experience. For more information, see our Cookie Policy.

You may choose not to allow some types of cookies. However, blocking some types may impact your experience of our site and the services we are able to offer. See the different category headings below to find out more or change your settings.

Allow all

Manage Consent Preferences

Strictly Necessary Cookies

Always active

These cookies are necessary for the website to function and cannot be switched off in our systems. They are usually only set in response to actions made by you which amount to a request for services, such as setting your privacy preferences, logging in or filling in forms. You can set your browser to block or alert you about these cookies, but some parts of the site will not then work.

Functional Cookies

These cookies enable the website to provide enhanced functionality and personalisation. They may be set by us or by third party providers whose services we have added to our pages. If you do not allow these cookies then some or all of these services may not function properly.

Performance Cookies

These cookies allow us to count visits and traffic sources so we can measure and improve the performance of our site. They help us to know which pages are the most and least popular and see how visitors move around the site.

Targeting Cookies

These cookies may be set through our site by our advertising partners. They may be used by those companies to build a profile of your interests and show you relevant adverts on other sites. If you do not allow these cookies, you will experience less targeted advertising.

Cookie List

Clear

Apply Cancel

Consent Leg.Interest

Confirm my choices

Power
