# Supplementary material for: scDenorm: a denormalization tool for integrating single-cell transcriptomics data
Source: Gigascience. 2026 Mar 31;15:giag032. doi: 10.1093/gigascience/giag032 (PMC13142155; doi:10.1093/gigascience/giag032)
Supplement: giag032_Reviewer_2_Report_original_submission — Reviewer 2 -- 7/19/2025 [file giag032_reviewer_2_report_original_submission.pdf]

Reviewer Report

Title: scDenorm: a denormalisation tool for integrating single-cell transcriptomics data

Version: Original Submission **Date:** 7/19/2025

Reviewer name: Reviewer 2

Reviewer Comments to Author:

Gigascience review for Huang et al, scDenorm: a denormalisation tool for integrating single-cell transcriptomics data
The authors aim to address the important and often irritating problem of how to combine scRNAseq data from different sources and provided after processing with different normalization tools. The authors note that often times raw counts matrices are not provided, nor information on how the data was normalized. Their tools aim to estimate the parameters used for data transformation (eg. scale factor, pseudocount addition, log-transformation), and reverse those transformations to return to the raw counts matrix again.
In an ideal world where public data is well-documented and actually fully available, this type of reverse-engineering would not be necessary, but due to the current state of many data repositories, in many cases, the tools the authors provide would be indeed very useful. The paper does a good job at identifying the problem, implementing a systematic solution with useful benchmarks, and showing multiple examples of the method's utility using public datasets. However, a few additional questions arise about their method:
1. Figure 1c-d: UMAP is a distortion/projection onto a 2D space, so it is not super surprising that different scaling factors produce clouds of cells in different locations. Do the differences in scaling factors affect the identification of the cells in any way? I.e. After denormalization for datasets of different scale factors/logbases/pseudocounts, when performing renormalization on the newly obtained raw counts, is there an optimal set of normalization parameters to now use?
2. Figure 2: For a particular dataset, if it is not known how the data was normalized and the transformation did not follow the form of log(C/T + P), will the tool alert the user to this? Or will the tool still attempt to find the best fit log base and pseudocount? In other words, prior to performing the denormalization, it might be helpful to display some metrics that indicate that the delta method was the most likely normalization method used, especially when ones tries to apply the tool to hundreds of datasets. (However, it seems it may not be possible to tell whether deNorm is successful until it is attempted (both empirically trying common log-base/pseudocount combinations, or solving the optimization equation), and then one assesses whether the resulting data is negative binomially distributed.)
3. Figure 3d does not seem sufficiently described in the text. The authors write: "For recovery error, the absolute values are below 10-6 in 27 datasets normalized with natural logarithmic transformation (Fig. 3d, Supplementary Table 1), indicating a good accuracy of scDenorm (Fig. 3f)." Fig 3f does show recovery error, and Fig 3e shows rounding error, but what does Fig 3d intend to show? Text clarification would be helpful.

4. For Fig 3d, why do certain datasets have lower "success rate"? It would be helpful for the bars in Fig 3d to be in the same order as the blue bars in Fig 3b, to better compare any relationship between gene or cell number with "success rate".
5. In addition, from Methods it appears that the determination of "Success Rate" relies on knowing the original raw count values. For those data sets where many of the cells could not be successfully denormalized to raw values, what is the impact of that on the downstream cell identification or DEG analysis?
6. Figure 4f: Not sure if this is an error in image display, but I do not see any blue points (original data) in the UMAP, as described in the legend.
7. Figures 5-6: The authors mention assessing how scDenorm helps in different scenarios where one might want to combine data: "the batch in the COVID-19 PBMCs dataset are samples from two patients, while in the human prefrontal cortex (PFC)31,35 dataset are samples from two different studies. And the batch in the human skin36 dataset are groups of samples from young and old donors."
The most interesting scenario here are the samples from two different studies, perhaps performed by different labs, with different bioinformatic pipelines, mapped to different versions of the genome, etc. Thus, I thought the example of integrating human PFC from two different studies and how the cell type identification changed (via the river plots) was the most compelling example, and the authors could consider making that a main figure instead.
8. The linked github repository, while commendable in providing code to reproduce figures, could benefit from further documentation/README showing the user how to use the tool.

Methods

Are the methods appropriate to the aims of the study, are they well described, and are necessary controls included? Choose an item.

Conclusions

Are the conclusions adequately supported by the data shown? Choose an item.

Reporting Standards

Does the manuscript adhere to the journal's guidelines on minimum standards of reporting? Choose an item.

Choose an item.

Statistics

Are you able to assess all statistics in the manuscript, including the appropriateness of statistical tests used? Choose an item.

Quality of Written English

Please indicate the quality of language in the manuscript: Choose an item.

Declaration of Competing Interests

Please complete a declaration of competing interests, considering the following questions:

- Have you in the past five years received reimbursements, fees, funding, or salary from an organisation that may in any way gain or lose financially from the publication of this manuscript, either now or in the future?
- Do you hold any stocks or shares in an organisation that may in any way gain or lose financially from the publication of this manuscript, either now or in the future?
- Do you hold or are you currently applying for any patents relating to the content of the manuscript?
- Have you received reimbursements, fees, funding, or salary from an organization that holds or has applied for patents relating to the content of the manuscript?
- Do you have any other financial competing interests?
- Do you have any non-financial competing interests in relation to this paper?

If you can answer no to all of the above, write 'I declare that I have no competing interests' below. If your reply is yes to any, please give details below.

I declare that I have no competing interests

I agree to the open peer review policy of the journal. I understand that my name will be included on my report to the authors and, if the manuscript is accepted for publication, my named report including any attachments I upload will be posted on the website along with the authors' responses. I agree for my report to be made available under an Open Access Creative Commons CC-BY license (http://creativecommons.org/licenses/by/4.0/). I understand that any comments which I do not wish to be included in my named report can be included as confidential comments to the editors, which will not be published.

Choose an item.

To further support our reviewers, we have joined with Publons, where you can gain additional credit to further highlight your hard work (see: https://publons.com/journal/530/gigascience). On publication of this paper, your review will be automatically added to Publons, you can then choose whether or not to claim your Publons credit. I understand this statement.

Yes Choose an item.
