# Supplementary material for: scDenorm: a denormalization tool for integrating single-cell transcriptomics data
Source: Gigascience. 2026 Mar 31;15:giag032. doi: 10.1093/gigascience/giag032 (PMC13142155; doi:10.1093/gigascience/giag032)
Supplement: giag032_Reviewer_3_Report_original_submission — Reviewer 3 -- 8/7/2025 [file giag032_reviewer_3_report_original_submission.pdf]

Reviewer Report

Title: scDenorm: a denormalisation tool for integrating single-cell transcriptomics data

Version: Original Submission **Date:** 8/7/2025

Reviewer name: Reviewer 3

Reviewer Comments to Author:

Reproducibility report for: scDenorm: a denormalisation tool for integrating single-cell transcriptomics data
Journal: GigaScience
ID number/DOI: GIGA-D-25-00209
Reviewer(s): Laura Caquelin, Department of Clinical Neuroscience, Karolinska Institutet, Sweden
----
1. Summary of the study
In order to reduce biaises caused by different normalization methods, the authors developed scDenorm, "an algorithm that reverts normalised single-cell omics data to raw counts". They evaluated its performance on large-scale datasets to assess its impact on data integration and downstream analysis.
----
2. Scope of reproducibility
According to our assessment the primary objective is: to evaluate whether scDenorm improves the biological relevance of gene expression analyses by comparing GO term enrichment from differentially expressed genes (DEGs), before and after denormalization against a gold standard.
Note: While this is not the main outcome highlighted in the article's conclusion, we chose to focus on the GO enrichment analysis because its involves statistical inference abd provides a clear and reproducible downstream result.
- Outcome: GO enrichment profiles based on hematopoietic stem and progenitor cells for each condition (gold standard, before scDenorm and after scDenorm).
- Analysis method outcome: "Downstream analysis after cell type annotation includes differential expression (DE) analysis and Gene Ontology (GO) pathway analysis. Differential gene expression analysis was conducted for each cell type (one against the rest) using the Wilcoxon test implemented in SCANPY19. As part of the dataset is used as the reference dataset, the differentially expressed genes (DEGs) derived from the reference dataset is used as 'gold standard' in our evaluation. The same approach was used for the calculation of the DEGs before and after scDenorm. The top differentially expressed genes were compared across different thresholds (top 50, 100, 200, 500, and 1000). As for GO pathway analysis, the enrichGO program was used on the top 500 differential expression genes.
DE and GO analysis with the correct labels from SolÃ©-Boldo et al. was conducted for each cell type (one against the rest) using the Wilcoxon test implemented in Seurat (V3.1.1), same as the version described in SolÃ©-Boldo et al. These analyses were conducted before scDenorm and after scDenorm. The differentially expressed genes were obtained from the study's supplementary materials as 'gold standard'". (Method

part, Dataset processing for data integration and downstream analysis section, page 14-
15)
- Main result: "Gene enrichment analysis of the differential genes in HSPCs indicated
that the GO terms enriched after scDenorm closely aligned with those of the 'gold standard', whereas the enrichment before scDenorm showed minimal overlap (Supplementary Fig. 10f). The enriched GO terms are relevant functions associated with HSPC cells, such as hematopoietic stem cell proliferation and hematopoietic progenitor cell differentiation (Fig. 5f)." (Results part, page 7)
----
3. AvailabilityofMaterials a. Data
- Data availability: Open
- Data completeness: Some data necessary to reproduce the main results were initially missing but were provided by the authors after we requested them.
- Access Method: Repository
- Repository: https://doi.org/10.5281/zenodo.11240443
-Data quality: The data files have been shared and appear sufficient for running the analyses. However, no metadata is provided to describe the content, structure, or origin of the files which limits interpretability and reusability.
b. Code
- Code availability: Open
- Programming Language(s): Python (Jupyter Notebook)
- Repository link: https://github.com/rnacentre/scDenorm-
reproducibility
- License: Apache 2.0
- Repository status: Public
- Documentation: The documentation could be improved. Important
details about the computational environment are missing, which makes it difficult to set up and reproduce the analysis. Additionally, since the code scripts are long and complex, navigating and understanding the workflow is challenging without clearer explanations.
----
4. Computational environment of reproduction analysis
- Operating system for reproduction: MacOS 15.5
- Programming Language(s): Python, R
- Code implementation approach: Using shared code with the jupyter notebook
- Version environment for reproduction: Creation of an environment with Python 3.10 and R 4.5.0
----
5. Results
5.1 Original study results - Results 1: Figure 5f
5.2 Steps for reproduction
-> Installation and environment setup
- Issue 1: Incompatibility between SCCAF and Python.
-- Resolved: SCCAF, when installed via PyPI, required scanpy==1.4.6, which is incompatible with Python 3.12, causing a TypeError due to metaclass conflicts. A new conda environment was created with Python

3.10. The authors specified that the PyPI version of SCCAF is outdated and recommended using their GitHub repository (https://github.com/rnacentre/sccaf).
- Issue 2: Incompatibility with recent versions of pandas.
-- Resolved: An ImportError occurred due to using pandas==2.2.3.
Downgrading to pandas==1.5.3 solved the issue.
- Issue 3: Incompatibility with recent versions of anndata.
-- Resolved: Using anndata==0.11.4 raised import errors. Downgraded to anndata==0.8.0, which resolved the issue.
Setting up a clean environment with Python 3.10, scanpy==1.9.1, matplotlib==3.5.3, pandas==1.5.3, anndata==0.8.0, and the GitHub SCCAF package resolved the issue. Authors validated the environment configuration above.
-> Running the original notebook (fig5.ipynb)
- Issue 4: KeyError due to missing labels ('Neutrophil', 'RBC') in confusion matrix.
-- Half resolved: Modified indexing logic to handle missing labels. The authors explained this variability stems from model randomness trained by SCCAF. Authors suggested filtering the label list accordingly:
conf_mat = conf_mat.loc[idx, [i for i in idx if i in conf_mat.columns]]
The proposed fix was to filter the columns with [i for i in idx if i in conf_mat.columns], but this still raises the same error due to missing labels in the rows.
conf_mat = conf_mat.loc[[i for i in idx if i in conf_mat.index] , [i for i in idx if i in conf_mat.columns]]
This error recurs throughout the code, requiring numerous modifications to be made.
- Issue 5: KeyError when building Sankey diagram ""Bad flow - couldn't find destination node: ('CD4+ T cell ', 'CD8+ T cell', 148)"
-- Resolved: Authors proposed instead of hardcoding the label list :
for i in new_ad.obs.predict0.value_counts()[['B cell', 'CD4+ T cell', 'NK cell', 'Other T', 'CD16+ Monocyte', 'cDC', 'pDC', 'HSPC', 'Plasmablast', 'Platelet']].items():
Simplify it to:
for i in new_ad.obs.predict0.value_counts().items():
- Issue 6: KeyError: 'predict0'
-- Resolved: Column predict0 did not exist. Replaced with predict and
predict_colors:
tmp = new_ad.obs.groupby(['cell', 'predict']).size()
right_cols = pd.Series(new_ad.uns['predict_colors'], index=new_ad.obs.predict.cat.categories)
Multiple predict0 en predict modifications had to be made throughout the code.
- Issue 7: ValueError no field of name Neutrophil
-- Unresolved: New error in this part of code:
df_bench=[]
for i in idx:
tmp=[]
for j in [50,100,200,500,1000]: tmp.append(len(set(adx0.uns['ground']['names'][i][:j]).intersection(adx0.uns['wilcoxon']['names'][i][:j])))
df_bench.append(tmp)
After asking authors about this error, they precised that if the goal is to reproduce the results of

scDenorm, the Sankey plot part can be skip and proceed directly to the section "After scDenorm".
-> Running the original notebook (fig5.ipynb) - "After scDenorm" section
- Issue 8: NameError: name 'ad1' is not defined
-- Resolved: Replaced ad1 with ad3, which had been defined earlier. Authors confirmed this change is correct.
- Issue 9: AttributeError: 'DataFrame' object has no attribute 'feature_name'
-- Unresolved: The line of code "ad.var_names=list(ad.var.feature_name)
not working. feature_name column not present in ad.var. This part was skipped.
- Issue 10: KeyError: 'wilcoxon' in plotting DEG results
-- Resolved: Clarified that 'wilcoxon' is the method, not the key. Correct usage:
sc.pl.rank_genes_groups(adx0, n_genes=25, sharey=False, key="ground")
- Issue 11: Missing data files
-- Resolved: The following files are required but not found in the repository: *
PBMC_before_scDenorm_DEGs.pkl
* PBMC_after_scDenorm_DEGs.pkl
* PBMC_go_analysis_result.csv
After request, the authors provided the script R_goanalysis.ipynb, which reproduces the PBMC_go_analysis_result.csv file, along with the necessary data files.
-> Running the R_goanalysis.ipynb and the "DEG and GO venn" section
5.3 Statistical comparison Original vs Reproduced results - Results:
- Comments: As the results we obtained were completely different from those reported in the manuscript, and identical before and after scDenorm, I contacted the authors to request details about the computational R environment to run the code R_goanalysis.ipynb. Although we used the environment they provided, this did not resolve the discrepancy.
- Errors detected: -
- Statistical Consistency: We obtained results that were completely different from those reported in the original study. Moreover, the outputs for the "after scDenorm" and "before scDenorm" conditions appeared graphically identical.
----
6. Conclusion
- Summary of the computational reproducibility review
The reproduction of the results in the scope of this computational reproducibility review was not successful. Many errors were encountered when running the original fig5.ipynb notebook, including missing or outdated variables, missing files, and misalignments in hard-coded labels. While most of these issues were resolved through direct communication with the authors and code modifications, some steps remained partially or completely irreproducible.
The results we obtained for the figure 5f were entirely different from those reported in the original manuscript. Despite using the R script (R_goanalysis.ipynb) and data files shared by the authors, and running them in the specified environment, the GO analysis results were inconsistent with those presented in the publication. Furthermore, the "after scDenorm" and "before scDenorm" conditions produced graphically identical results. This raises questions about whether the differences come from the code, the data, or something else in the analysis.

- Recommendations for authors
To make the study easier to reproduce and more transparent, we suggest the following:
-- Add a requirement file that lists all the needed packages with their exact versions and mention which Python and R versions are supported. Make it clear in the documentation that the SCCAF version on PyPI is outdated and point users to the up-to-date version on GitHub.
-- Make sure all data files needed to reproduce the figures are available in the repository.
-- Check that the analysis code does not depend on fixed labels or assumptions that might not work with other datasets or slightly different results.
-- Clearly explain which parts of the results may vary due to randomness in the model and how much variation users should expect.
-- Consider adding some pre-computed results or intermediate files so users can compare their outputs with the original study more easily.

Methods

Are the methods appropriate to the aims of the study, are they well described, and are necessary controls included? Choose an item.

Conclusions

Are the conclusions adequately supported by the data shown? Choose an item.

Reporting Standards

Does the manuscript adhere to the journal's guidelines on minimum standards of reporting? Choose an item.

Choose an item.

Statistics

Are you able to assess all statistics in the manuscript, including the appropriateness of statistical tests used? Choose an item.

Quality of Written English

Please indicate the quality of language in the manuscript: Choose an item.

Declaration of Competing Interests

Please complete a declaration of competing interests, considering the following questions:

- Have you in the past five years received reimbursements, fees, funding, or salary from an organisation that may in any way gain or lose financially from the publication of this manuscript, either now or in the future?
- Do you hold any stocks or shares in an organisation that may in any way gain or lose financially from the publication of this manuscript, either now or in the future?
- Do you hold or are you currently applying for any patents relating to the content of the manuscript?
- Have you received reimbursements, fees, funding, or salary from an organization that holds or has applied for patents relating to the content of the manuscript?
- Do you have any other financial competing interests?
- Do you have any non-financial competing interests in relation to this paper?

If you can answer no to all of the above, write 'I declare that I have no competing interests' below. If your reply is yes to any, please give details below.

1. No 2. No 3. No 4. No 5. No 6. No

I agree to the open peer review policy of the journal. I understand that my name will be included on my report to the authors and, if the manuscript is accepted for publication, my named report including any attachments I upload will be posted on the website along with the authors' responses. I agree for my report to be made available under an Open Access Creative Commons CC-BY license (http://creativecommons.org/licenses/by/4.0/). I understand that any comments which I do not wish to be included in my named report can be included as confidential comments to the editors, which will not be published.

Choose an item.

To further support our reviewers, we have joined with Publons, where you can gain additional credit to further highlight your hard work (see: https://publons.com/journal/530/gigascience). On publication of this paper, your review will be automatically added to Publons, you can then choose whether or not to claim your Publons credit. I understand this statement.

Yes Choose an item.
