# Supplementary material for: scDenorm: a denormalization tool for integrating single-cell transcriptomics data
Source: Gigascience. 2026 Mar 31;15:giag032. doi: 10.1093/gigascience/giag032 (PMC13142155; doi:10.1093/gigascience/giag032)
Supplement: giag032_Reviewer_3_Report_revision_1 — Reviewer 3 -- 11/18/2025 [file giag032_reviewer_3_report_revision_1.pdf]

Reviewer Report

Title: scDenorm: a denormalisation tool for integrating single-cell transcriptomics data

Version: Revision 1 **Date:** 11/18/2025

Reviewer name: Reviewer 3

Reviewer Comments to Author:

Reproducibility report for: scDenorm: a denormalisation tool for integrating single-cell transcriptomics data
Journal: Gigascience
ID number/DOI: GIGA-D-25-00209
Reviewer(s): Laura Caquelin, Department of Clinical Neuroscience, Karolinska Institutet, Sweden
----
1. Context
This report corresponds to a second assessment of the computational reproducibility of the article GIGA-D-25-00209, following a revision by the authors after the first round of review.
The scope of the computational reproducibility review is to reproduce the results in figure 5f related to the evaluation of whether scDenorm improves the biological relevance of gene expression analyses by comparing GO term enrichment from differentially expressed genes (DEGs), before and after denormalization against a gold standard.
----
2. Changes since the first review
The authors made several changes based on comments from the initial computational reproducibility review:
- Reorganized and updated the code in Fig5.ipynb and R_goanalysis.ipynb,
- Created a docker environment,
- Provided pre-computed GO enrichment results and intermediate files in Zenodo,
- Added an environment.yaml file for python and installed_packages.csv file for R,
- Improved the Readme file.
----
3. Availability of Materials
a. Data
- Data availability: Open
- Data completeness: Complete = all data necessary to reproduce main results are available
- Access Method: Repository
- Repository: https://zenodo.org/records/17275776 (new link)
-Data quality: Completed, no metadata was shared.
b. Code
- Code availability: Open
- Programming Language(s): R and Python

- Repository link:
https://github.com/rnacentre/scDenorm_reproducibility
- License: -
- Repository status: Public
- Documentation: A Readme file is provided, but some improvements are needed.
----
4. Computational environment of reproduction analysis
- Operating system for reproduction: MacOS 15.6.1
- Programming Language(s): R (jupyter notebook), Python (jupyter notebook)
- Code implementation approach: Using shared code
- Version environment for reproduction: Docker version 28.5.1, R version 4.5.1 (2025-06-13), Python 3.13.9
----
5. Results
5.1 Original study results
- Results 1: In the revised version 1 of the paper , Figure 5 does not appear in the PDF. Therefore, we assumed that the figure is identical to the one in the original submission, especially based on the authors' comment stating that "We re-ran the analysis and obtained results consistent with those reported in the manuscript."
Below is Figure 5f from the original paper:
(See screenshot)
The intermediate file "PBMC_go_analysis_result.csv" shared in Zenodo was used to run the authors' code and extract the numerical values of this graph, enabling direct comparison:
(See screenshot)
5.2 Steps for reproduction
-> Follow the readme guidelines to set up the environnement:
--> Download the notebooks from Github.
Note: notebook list in readme is not updated.
--> Install docker and jupyter.
Note: the jupyter installation is not precised in the readme file.
--> Download data.
--- Issue 1: To download the data, no link was provided in the readme file in the Github repository. The zenodo link in the manuscript was not updated in the "Availability of Data and Materials" section.
---- Resolved: The new link was provided in the authors' response to the reviewer but needs to be added in the manuscript and the readme file. The link is https://zenodo.org/records/17275776.
--- Issue 2: Guidelines in the README file do not correspond to the actual procedure.
---- Resolved: From the Zenodo archive, download scDenorm_reproducibility.tar.gz, unzip it, and place the data into the data folder. It would be clearer if the authors explicitly specified which files should be placed in the data directory to avoid confusion.
--> Run the docker image.
--- Issue 3: The following Docker instructions provided by the authors do not work as written:
tar -xzf scdenorm_v0.tar.gz

docker load -i scdenorm_v0.tar
docker run -p 8888:8888 -v /path/to/scDenorm_reproducibility:/app scdenorm_v0 \ jupyter lab --ip=0.0.0.0 --no-browser --allow-root
scdenorm_v0.tar.gz does not contain a standard Docker .tar image. After extraction, the result is a directory named scdenorm_v0, not a .tar file. docker load -i scdenorm_v0.tar fails because scdenorm_v0.tar does not exist. Docker must be running before executing docker load. The extraction step is sensitive to the current directory, but this is not documented.
---- Resolved: The image can be successfully loaded directly from the .tar.gz file using:
docker load < scdenorm_v0.tar.gz
After this, the image scdenorm_v0:latest is available.
--- Issue 4: Two main issues appeared when running the docker run command:
----- "WARNING: The requested image's platform (linux/amd64) does not match the detected host platform (linux/arm64/v8)"
----- "mounts denied: The path /path/to/scDenorm_reproducibility is not shared from the host".
---- Resolved: To be able to use the docker run command, two steps were needed:
----- Share the project folder with docker manually: Docker â†’ Preferences â†’ Resources â†’ File Sharing â†’ add the local project path
----- Update the docker run command with the local path and add linux/amd64:
docker run --platform linux/amd64\
-p 8888:8888\
-v /path/to /scDenorm_reproducibility:/app\
scdenorm_v0\
jupyter lab --ip=0.0.0.0 --no-browser --allow-root
--- Issue 5: R was not connected to Jupyter.
---- Resolved: In the terminal, this made the R kernel available:
R
install.packages("IRkernel")
IRkernel::installspec()
-> Run the Fig5_R__goanalysis.ipynb script
--- Issue 6: Docker image does not install the R packages. The file installed_packages.csv lists all required R packages, but they are not installed automatically.
---- Resolved: A solution was to install all required packages at the start of the notebook using the csv file:
pkg_list <- read.csv("installed_packages.csv", stringsAsFactors = FALSE)
for (pkg in pkg_list$Package) {
if (!requireNamespace(pkg, quietly = TRUE)) {
message(" Installing the package: ", pkg)
tryCatch(
{
install.packages(pkg, dependencies = TRUE)
},
error = function(e) {

message("Failed to install package: ", pkg)
}
)
} else {
message(" Already installed: ", pkg)
}
}
Additional required packages from Bioconductor:
if (!require("BiocManager", quietly = TRUE))
install.packages("BiocManager")
if (!requireNamespace("enrichplot", quietly = TRUE)) {
BiocManager::install("enrichplot", ask = FALSE)}
if (!requireNamespace(c("enrichplot","org.Hs.eg.db"), quietly = TRUE)) {
BiocManager::install(c("clusterProfiler", "org.Hs.eg.db"), ask = FALSE)}
After these steps, the R script ran without errors.
-> Run the Fig5.ipynb script
--- Issue 7: The same issue as no. 3 occurred again, the docker image did not provide a working python environment. Attempt to create the python environment with environment.yaml file.
conda env create -f environment.yaml
Failed because many packages do not exist for the system, for exemple:
"ipyw_jlab_nb_ext_conf ==0.1.0 py39h06a4308_1 does not exist (perhaps a typo or a missing channel);"
These errors seem to happen because the environment file contains many Linux-specific packages.
---- Unresolved: Authors should provide an environment file working in all systems. A temporary solution was used: create a minimal clean environment:
conda env create -f environment.yaml
Environment.yaml:
name: scdenorm_clean
channels:
- conda-forge
- bioconda
- defaults
dependencies:
- python=3.9
- numpy
- pandas
- scipy
- matplotlib
- seaborn
- tqdm
- scanpy
- anndata
- tables

- pip
- pip:
- scdenorm
- SCCAF
Then:
conda activate scdenorm_clean
conda install ipykernel
python -m ipykernel install --user --name=scdenorm_clean --display-name "Python (scdenorm)"
Select this kernel in Jupyter Notebook to run the python files.
An additional issue was the conflict between matplotlib and scapy. Resolved with:
conda install matplotlib=3.6.3
conda install -c conda-forge scanpy (Successfully installed scanpy-1.10.3)
--> The script was executed only by starting from HSPC section.
--- Issue 8: A specific issue appeared after filtering the dataframe tmp1 by go_terms, only two cell types remained (b0 and b1), and b1n disappeared. This was because no row corresponding to b1n matched the selected GO terms.
---- Unresolved: Fig5_R__goanalysis.ipynb was re-run multiple times to obtain a new version of the PBMC_go_analysis_result.csv. However, the error persists.
5.3 Statistical comparison Original vs Reproduced results
- Reproduced results: Figure 5f
(see screenshots)
- Comments: The figure obtained does not show all go_terms nor all categories. Only categories b1 and b0 are shown.
- Errors detected: -
- Statistical Consistency: If there is no error, b0 would correspond to the gold standard and b1 to the before_scDenorm cell type. The -log10(adjusted p-value) values reproduced do not match the reported values.
----
6. Conclusion
- Follow-up on previous recommendations:
In the first round of review, we noted the following points:
-- Add a requirement file that lists all the needed packages with their exact versions. Authors provided an installed_packages.csv which allowed to manually reconstruct the R environment. However, a functional environment.yaml is required.
-- Make sure all data files needed to reproduce the figures are available in the repository. The authors updated the Zenodo link and uploaded all relevant intermediate files.
-- Clearly explain which parts of the results may vary due to randomness in the model and how much variation users should expect. This point remains insufficiently addressed.
- Summary of the second computational reproducibility review
Both scripts used to reproduce the figure 5f were executed, but several issues were encountered. The results obtained differ from the ones reported in the manuscript. In particular:
-- Several p-values could not be reproduced,

-- Some discrepancies appeared in the GO enrichment analysis.
Some clarifications are required for the GO analysis about why some cell types are not present after filtering.
Significant manual intervention was required, to improve the reproducibility, here is some new recommendations:
-- Improve the readme file. The readme does not reflect the real procedure needed to reproduce the results (incorrect docker instructions, missing steps, outdated notebook list). Clear instructions should be added regarding:
--- the required jupyter installation,
--- file paths and folder structure,
--- link to the zenodo
--- how to run each notebook
-- Provide a functional environment.yaml. The provided docker image fails to create the required Python and R environments.

Methods

Are the methods appropriate to the aims of the study, are they well described, and are necessary controls included? Choose an item.

Conclusions

Are the conclusions adequately supported by the data shown? Choose an item.

Reporting Standards

Does the manuscript adhere to the journal's guidelines on minimum standards of reporting? Choose an item.

Choose an item.

Statistics

Are you able to assess all statistics in the manuscript, including the appropriateness of statistical tests used? Choose an item.

Quality of Written English

Please indicate the quality of language in the manuscript: Choose an item.

Declaration of Competing Interests

Please complete a declaration of competing interests, considering the following questions:

- Have you in the past five years received reimbursements, fees, funding, or salary from an organisation that may in any way gain or lose financially from the publication of this manuscript, either now or in the future?
- Do you hold any stocks or shares in an organisation that may in any way gain or lose financially from the publication of this manuscript, either now or in the future?
- Do you hold or are you currently applying for any patents relating to the content of the manuscript?
- Have you received reimbursements, fees, funding, or salary from an organization that holds or has applied for patents relating to the content of the manuscript?
- Do you have any other financial competing interests?
- Do you have any non-financial competing interests in relation to this paper?

If you can answer no to all of the above, write 'I declare that I have no competing interests' below. If your reply is yes to any, please give details below.

1. No 2. No 3. No 4. No 5. No 6. No

I agree to the open peer review policy of the journal. I understand that my name will be included on my report to the authors and, if the manuscript is accepted for publication, my named report including any attachments I upload will be posted on the website along with the authors' responses. I agree for my report to be made available under an Open Access Creative Commons CC-BY license (http://creativecommons.org/licenses/by/4.0/). I understand that any comments which I do not wish to be included in my named report can be included as confidential comments to the editors, which will not be published.

Choose an item.

To further support our reviewers, we have joined with Publons, where you can gain additional credit to further highlight your hard work (see: https://publons.com/journal/530/gigascience). On publication of this paper, your review will be automatically added to Publons, you can then choose whether or not to claim your Publons credit. I understand this statement.

Yes Choose an item.
