# Supplementary material for: scDenorm: a denormalization tool for integrating single-cell transcriptomics data
Source: Gigascience. 2026 Mar 31;15:giag032. doi: 10.1093/gigascience/giag032 (PMC13142155; doi:10.1093/gigascience/giag032)
Supplement: giag032_Reviewer_3_Report_revision_2 — Reviewer 3 -- 3/9/2026 [file giag032_reviewer_3_report_revision_2.pdf]

Reviewer Report

Title: scDenorm: a denormalisation tool for integrating single-cell transcriptomics data

Version: Revision 2 **Date:** 3/9/2026

Reviewer name: Reviewer 3

Reviewer Comments to Author:

Reproducibility report for: scDenorm: a denormalisation tool for integrating single-cell transcriptomics data
Journal: Gigascience
ID number/DOI: GIGA-D-25-00209
â€‹â€‹â€‹Reviewer(s): Laura Caquelin, Department of Clinical Neuroscience, Karolinska Institutet, Sweden
----
Context
This report corresponds to the third assessment of the computational reproducibility of the article GIGA-D-25-00209, following a revision by the authors after the second round of review.
The scope of the computational reproducibility review is to reproduce the results in figure 5f related to the evaluation of whether scDenorm improves the biological relevance of gene expression analyses by comparing GO term enrichment from differentially expressed genes (DEGs), before and after denormalization against a gold standard.

Changes since the first review
The authors made several changes based on comments from the second computational reproducibility review:
- Improve the Docker environment,
- And improve the README file.

Availability of Materials
Data
- Data availability: Open
- Data completeness: Complete = all data necessary to reproduce main results are available
- Access Method: Repository
- Repository: https://doi.org/10.5281/zenodo.11240442
-Data quality: Structured
Code
- Code availability: Open
- Programming Language(s): R, Python
- Repository link: https://github.com/rnacentre/scDenorm_reproducibility
- License: -

- Repository status: Public
- Documentation: Clear Readme file

----

4. Computational environment of reproduction analysis
- Operating system for reproduction: MacOS 15.6.1 (ARM64)
- Programming Language(s): R, Python
- Code implementation approach: Using shared code
- Version environment for reproduction: Râ€¯4.0.2, IRkernelâ€¯1.3, Pythonâ€¯3.9, Docker used to containerize environment (ARM build for Python notebook, Intel build via GitHub Actions for R notebook).

----

5. Results

Original study results

- Results 1: In the revised version 2 of the paper (as in the revised version 1), Figure 5 does not appear in the PDF. Therefore, we assumed that the figure is identical to the one in the original submission. Below is Figure 5f from the original submission: (screenshot)

Steps for reproduction

-> First attempt to reproduce the analyses

-- > Download the notebooks from Github

The following step was added from the guidelines provided by the authors in the readme:

-- Rename the folder from "scDenorm_reproducibility-main" to "scDenorm_reproducibility"

-- Create a sub-folder named "data" inside "scDenorm_reproducibility"

-->Download the required data from Zenodo and place it in the data subfolder

--> Load the provided Docker Image

--> Run the container and lauch Jupyter lab

--> Run the Fig5_R__goanalysis.ipynb script

Issue 1: R kernel not correctly detected in Jupyter.

-- Resolved: The R kernel was registered under /root/.local/share/jupyter/kernels/sc, which was not properly linked to the active conda environment. As a result, Jupyter either did not detect the kernel correctly or failed to start it. Reinstalling and registering IRkernel inside the sc conda environment using IRkernel::installspec(user = FALSE) resolved the issue. The ir kernel then appeared correctly in jupyter kernelspec list. Below the steps followed:

```
rm -rf /root/.local/share/jupyter/kernels/*
conda activate sc
install.packages('IRkernel')
conda install -c conda-forge r-irkernel -y
R -e "IRkernel::installspec(user = FALSE)"
```

Issue 2: Extremely slow execution on Apple Silicon (ARM-based Mac)

-- Resolved: The Docker image provided via Zenodo is built for Intel (amd64) architecture, while the system uses Apple Silicon (arm64) which significantly reduces performance. Large Python libraries (e.g., Scanpy) take several minutes to import, and Jupyter frequently remains in a prolonged "busy" state. The authors provided the dockerfile to rebuild the image for arm64 by email.

-> Second attempt to reproduce the analyses with the Dockerfile

-- > Build the docker image

Issue 3: During the Docker image build, the installation of Python dependencies failed due to version incompatibilities. Some packages required Python 3.10 or higher while the environment was set to Python 3.9, and the requested version of pyqtchart (5.12) was not available for the current architecture. After upgrading, another error occurred because PyQt-related packages required additional Qt build tools that were not available in the container, which caused the build to stop.

-- Resolved: The issue was resolved by upgrading the environment to Python 3.10 and by updating pyqtchart to a compatible and available version (5.15.7) in the Dockerfile. NumPy (1.26.4) was installed only with Conda to avoid conflicts. Additionally, PyQt-related packages were installed using Conda instead of pip to prevent build errors related to missing Qt tools, ensuring a stable and successful Docker build.

--> Run the container and lauch Jupyter lab

--> Run the Fig5_R__goanalysis.ipynb script with R [conda env:sc] kernel

--> Run the Fig5.ipynb script with Python [conda env:sc] kernel

Issue 4: When running the analysis in Jupyter, the import of SCCAF and running sc.tl.umap() failed. SCCAF initially failed because the pkg_resources module was missing, and UMAP failed due to a version incompatibility between Scanpy (1.9.6) and umap-learn.

-- Resolved: The problem was fixed by installing the missing packages setuptools<70 (providing pkg_resources) in the environment used for the analysis. Downgrading Scanpy to 1.9.3 and setting umap-learn to 0.5.3 to ensure UMAP works correctly. These changes were applied in the Dockerfile.

Issue 5: When running, loading the dataset with: sc.read('data/PBMC_before_scDenorm.h5ad'), sc.read('data/PBMC_after_scDenorm.h5ad') failed because the files were not found. The actual file in the folder was named "fig5_PBMC_before_scDenorm.h5ad", "fig5_PBMC_after_scDenorm.h5ad" causing a FileNotFoundError.

-- Resolved: The problem was fixed by updating the code to use the correct filename.

Issue 6: Running sc.tl.rank_genes_groups on the dataset initially failed with a KeyError: 'base', because the input data was not log-normalized for adx0 and adx1.

-- Pending: Solved by applying a log-transformation (sc.pp.log1p) to the dataset before running the function but scanpy issues a warning suggesting that the data should be logarithmized, even though the data are already log-transformed.

Issue 7: For tmp2=tmp2.loc in HSPC part, selecting specific gene sets from the result table failed because some of the requested entries were not present in the dataset.

-- Unresolved: This issue should be addressed. To be able to run the code:

```
desired = [ 'regulation of cell cycle phase transition',
'hematopoietic stem cell differentiation',
'regulation of stem cell differentiation',
'regulation of hematopoietic stem cell differentiation',
'regulation of cell cycle G2/M phase transition',
'hematopoietic progenitor cell differentiation',
'hematopoietic stem cell proliferation',
'cell cycle G2/M phase transition',
```

'regulation of response to oxidative stress',
'positive regulation of cell cycle',
'mitotic cell cycle checkpoint',
'mitotic DNA integrity checkpoint',
'mitotic DNA damage checkpoint',
'negative regulation of stem cell differentiation']
existing = [x for x in desired if x in tmp2.index]
tmp2 = tmp2.loc[existing, :]

We observed that the resulting GO terms differed from the ones reported in the original study, even when using the same input gene lists:

Issue 8: Bioconductor annotation packages versions (org.Hs.eg.db, clusterProfiler) are not precised in the dockerfile and may have been updated since the original analysis. Their contents (gene mappings) can change between versions.

-- Resolved: After requesting, the authors sent a new version of the Docker file with the exact release and package versions as in the original study for Bioconductor.

-> Third attempt to reproduce the analyses with the modified Dockerfile

-- > Build the docker image

I encountered the same issue as the one decribed in the second try and an additional issue described below. The docker file was modified accordingly.

Issue 9: The fixed versions of Python, PyQt, pyqtchart, and pyqtwebengine were incompatible with each other and with the ARM architecture, causing the environment to fail. Same with the exact versions of R (r-base=4.0.2=he766273_1) and r-irkernel (1.3=r40hc72bb7e_0) specified that were not available for the ARM architecture, so Conda could not find them and the build failed. Not available in ARM.

-- Resolved: As in the previous Dockerfile, no PyQt, pyqtchart, and pyqtwebengine, R and R-irkernel versions were specified allowing the Docker to build successfully.

--> Run the container and launch Jupyter lab

--> Run the Fig5_R__goanalysis.ipynb script with R [conda env:sc] kernel

Even after trying to resolve the compatibility issues, it was not possible to run the R Jupyter notebook. The kernel remained stuck in "busy" mode and never executed.

Solution: To overcome this, the notebook Fig5_R__goanalysis.ipynb was run using GitHub Actions in a GitHub repository on an Intel (x86) environment. This allowed the notebook to be executed successfully, and the output files were obtained, using a run.yml file:

name: Run Notebook
on: push: branches: [main]
jobs: execute-notebook: runs-on: ubuntu-latest
steps:
- uses: actions/checkout@v3
- name: Build Docker image
run: docker build -t repro_image .
- name: Execute notebook
run: |
docker run --rm -v ${{ github.workspace }}:/app repro_image \

```
conda run -n sc jupyter nbconvert \
--to notebook \
--execute \
--inplace \
--ExecutePreprocessor.kernel_name=ir \
--ExecutePreprocessor.allow_errors=True \
Fig5_R_goanalysis.ipynb
- name: Upload notebook and data as artifact
uses: actions/upload-artifact@v4
with:
name: executed-notebook
path: |
Fig5_R_goanalysis.ipynb
data/**
```

Running the Python notebook Fig5 in the same way was not possible because the input files were too large to upload to GitHub. To address this, an ARM Docker image was built by modifying the Dockerfile (attached with the report). Only the HSPC section was executed in this ARM environment to generate Figure 5f.

5.3 Statistical comparison Original vs Reproduced results

- Reproduced results: (screenshots)
- Comments: -
- Errors detected: -
- Statistical Consistency: The statistical outputs were consistent with the original study. The -log10(adjusted p-values) aligned with the reported results. Due to the partial execution of the Python notebook, statistical consistency could only be confirmed for the reproduced HSPC section, which matches the original figure.

----

6. â€‹â€‹Conclusion

- Summary of the third computational reproducibility review

The reproduction of Figure 5f was successful by using the following strategy: the R notebook could be executed fully only using GitHub Actions in an Intel environment, producing the same outputs as reported. The Python notebook could not be fully executed locally due to input file size constraints, but the HSPC section was successfully reproduced, generating Figure 5f. Overall, the main findings are reproducible.

- Follow-up on previous recommendations:

To further facilitate reproducibility for future users, it would be beneficial if the authors could:

1. Allow the R notebook to be executed directly through GitHub Actions in an Intel environment, ensuring users on ARM machines can reproduce the results without manual work.
2. Share the updated docker file and updated instructions in the Github repository.

Methods

Are the methods appropriate to the aims of the study, are they well described, and are necessary controls included? Choose an item.

Conclusions

Are the conclusions adequately supported by the data shown? Choose an item.

Reporting Standards

Does the manuscript adhere to the journal's guidelines on minimum standards of reporting? Choose an item.

Choose an item.

Statistics

Are you able to assess all statistics in the manuscript, including the appropriateness of statistical tests used? Choose an item.

Quality of Written English

Please indicate the quality of language in the manuscript: Choose an item.

Declaration of Competing Interests

Please complete a declaration of competing interests, considering the following questions:

- Have you in the past five years received reimbursements, fees, funding, or salary from an organisation that may in any way gain or lose financially from the publication of this manuscript, either now or in the future?
- Do you hold any stocks or shares in an organisation that may in any way gain or lose financially from the publication of this manuscript, either now or in the future?
- Do you hold or are you currently applying for any patents relating to the content of the manuscript?
- Have you received reimbursements, fees, funding, or salary from an organization that holds or has applied for patents relating to the content of the manuscript?
- Do you have any other financial competing interests?
- Do you have any non-financial competing interests in relation to this paper?

If you can answer no to all of the above, write 'I declare that I have no competing interests' below. If your reply is yes to any, please give details below.

1. no 2. no 3. no 4. no 5. no 6. no

I agree to the open peer review policy of the journal. I understand that my name will be included on my report to the authors and, if the manuscript is accepted for publication, my named report including any attachments I upload will be posted on the website along with the authors' responses. I agree for my report to be made available under an Open Access Creative Commons CC-BY license (http://creativecommons.org/licenses/by/4.0/). I understand that any comments which I do not wish to be included in my named report can be included as confidential comments to the editors, which will not be published.

I agree to the open peer review policy of the journal

To further support our reviewers, we have joined with Publons, where you can gain additional credit to further highlight your hard work (see: https://publons.com/journal/530/gigascience). On publication of this paper, your review will be automatically added to Publons, you can then choose whether or not to claim your Publons credit. I understand this statement.

Yes Choose an item.
